# Structural and computational supported development of 2,5-disubstituted-1,3,4-oxadiazole analogues as active LOX, urease, and α-glucosidase inhibitors

**DOI:** 10.1038/s41598-026-35499-1

**Published:** 2026-01-21

**Authors:** Jamila Javid, Aziz-ur-Rehman, Javed Iqbal, Ijaz Ahmed, Nadia Bhatti, Aleksey Kuznetsov, Fatiqa Zafar, Muhammad Adnan Ayub, Osama A. Mohammed, Samiah H. Al-Mijalli, Munawar Iqbal, Syed A. Ali Shah

**Affiliations:** 1 Department of Chemistry, Government College University, Lahore, 54000 Pakistan; 2 Department of Chemistry, University of Sahiwal, Sahiwal, 57000 Pakistan; 3 School of Chemistry, University of the Punjab, Lahore, 54590 Pakistan; 4 Departamento de Química, Campus Santiago Vitacura, Universidad Técnica Federico Santa María, Vitacura, 7660251 Santiago Chile; 5 Department of Pharmacology, College of Medicine, University of Bisha, Bisha, 61922 Saudi Arabia; 6 Department of Biology, College of Sciences, Princess Nourah bint Abdulrahman University, P.O. Box 84428, Riyadh, 11671 Saudi Arabia; 7 Renewable Energy and Environmental Technology Center, University of Tabuk, Tabuk, 47913 Saudi Arabia; 8 School of Chemistry, University of the Punjab, Lahore , 54590 Pakistan; 9 Faculty of Pharmacy, Universiti Teknologi MARA Cawangan Selangor Kampus Puncak Alam, Bandar Puncak Alam, Puncak Alam, 42300 Selangor Malaysia; 10 Atta-ur-Rahman Institute for Natural Product Discovery (AuRIns), Universiti Teknologi MARA Cawangan Selangor Kampus Puncak Alam, Bandar Puncak Alam, Puncak Alam, 42300 Selangor Malaysia

**Keywords:** 1,3,4-Oxadiazole, Lipoxygenase, Urease, Acetyl cholinesterase, Inhibition, DFT study, Biochemistry, Drug discovery, Diseases, Molecular medicine, Chemistry

## Abstract

A series of *N*-substituted analogues of 1,3,4-oxadiazole were synthesized and screened for their enzyme inhibitory activity against lipoxygenase, α-glucosidase, and urease enzymes. Spectroscopy studies including IR, ^13^C-NMR, and ^1^H-NMR techniques were used to confirm structures of the novel compounds obtained. Compounds **7a**, **7f**, **7i**, **7l**, and **7n** were found to be the best inhibitor candidates with the highest % of inhibition (89.42 ± 1.33, 78.79 ± 1.29, 84.87 ± 1.53, 75.67 ± 1.62, and 89.54 ± 1.48, respectively) and the least IC_50_ values (7.15 ± 1.34, 14.26 ± 1.07, 9.12 ± 1.17, 14.31 ± 1.19, and 7.03 ± 1.08 µM, respectively) against α-glucosidase compared to acarbose used as a standard. Compounds **7b**, **7h**, and **7n** were found to be the most potent with % of inhibition 92.2 ± 0.5, 94.5 ± 0.6, and 92.8 ± 0.8, respectively, and with the IC_50_ values 4 ± 1.05, 1.0 ± 0.3, and 1.5 ± 0.5 µM, respectively, against the lipoxygenase. With the IC_50_ value of 21.85 ± 1.43 µM and the percentage inhibition of 98.45 ± 1.69%, compound **7l** was found to be more active in comparison with the standard drug thiourea. Docking analysis further supported these biological outcomes by demonstrating strong, well-oriented binding poses and favorable interaction profiles for the most active inhibitors.

**Supplementary Information:**

The online version contains supplementary material available at 10.1038/s41598-026-35499-1.

## Introduction

Oxadiazoles possess vast medicinal potential and have received significant attention in pharmaceutical research. 1,3,4-oxadiazole derivatives have demonstrated broad spectrum activities in agrochemical and pharmaceutical fields. They have been reported to demonstrate versatile biomedical characteristics, e.g., diuretic^1^, anti-convulsant^2^, anti-analgesic^3^, antimicrobial^4^, lipid peroxidation inhibitor^5^, muscle relaxants^6^, antimalarial^7^, anti-inflammatory^8^, herbicidal^9^, anti-HIV^10^, anti-tubercular^11^, insecticidal^12^, genotoxic^13^, virucidal^14^ etc. So, researchers from all over the world have been attracted to explore this moiety to extend their search for novel drug candidates.

Urease is a well-known enzyme that converts urea into carbon dioxide and ammonia by the hydrolysis reaction. It is present in vertebrates, plants, fungi, and bacteria^15^. Pathological disorders such as pyelonephritis, peptic and gastric ulceration, kidney stones, hepatic coma etc. are caused by ammonia produced by hyperactivity of urease^16^. Thus, regulation of urease activity through inhibitors is of high importance because of the vital role of urease in the above-mentioned clinical complications^17^.

Next, α-glucosidase is a very important hydrolase enzyme found in human intestine on cellular brush boarder surfaces. Hydrolysis of carbohydrates to glucose by α-glucosidase is very essential because only monosaccharides can be absorbed by human intestine^18^. α-Glycosidase is an oral drug used to treat diabetes mellitus type 2^19^.

Finally, lipoxygenase (LOX) is a widely distributed enzyme in plant and animal kingdoms; it represents a class of non-heam dioxygenases containing iron which take part in metabolism of arachidonic acid and are responsible for synthesis of many lipids which cause inflammation. They play a vital role in organizing newfangled capillary vessels, processes of tumor angiogenesis-thrombosis, and development of various pathological conditions, e.g., arthritis and cancer, therefore lipoxygenases are the focus of interest for researchers to study inhibitor-based mechanisms and to design medicines to treat various disorders such as cancer, autoimmune diseases, bronchial asthma, and inflammations^20–26^.

**Fig. 1:**
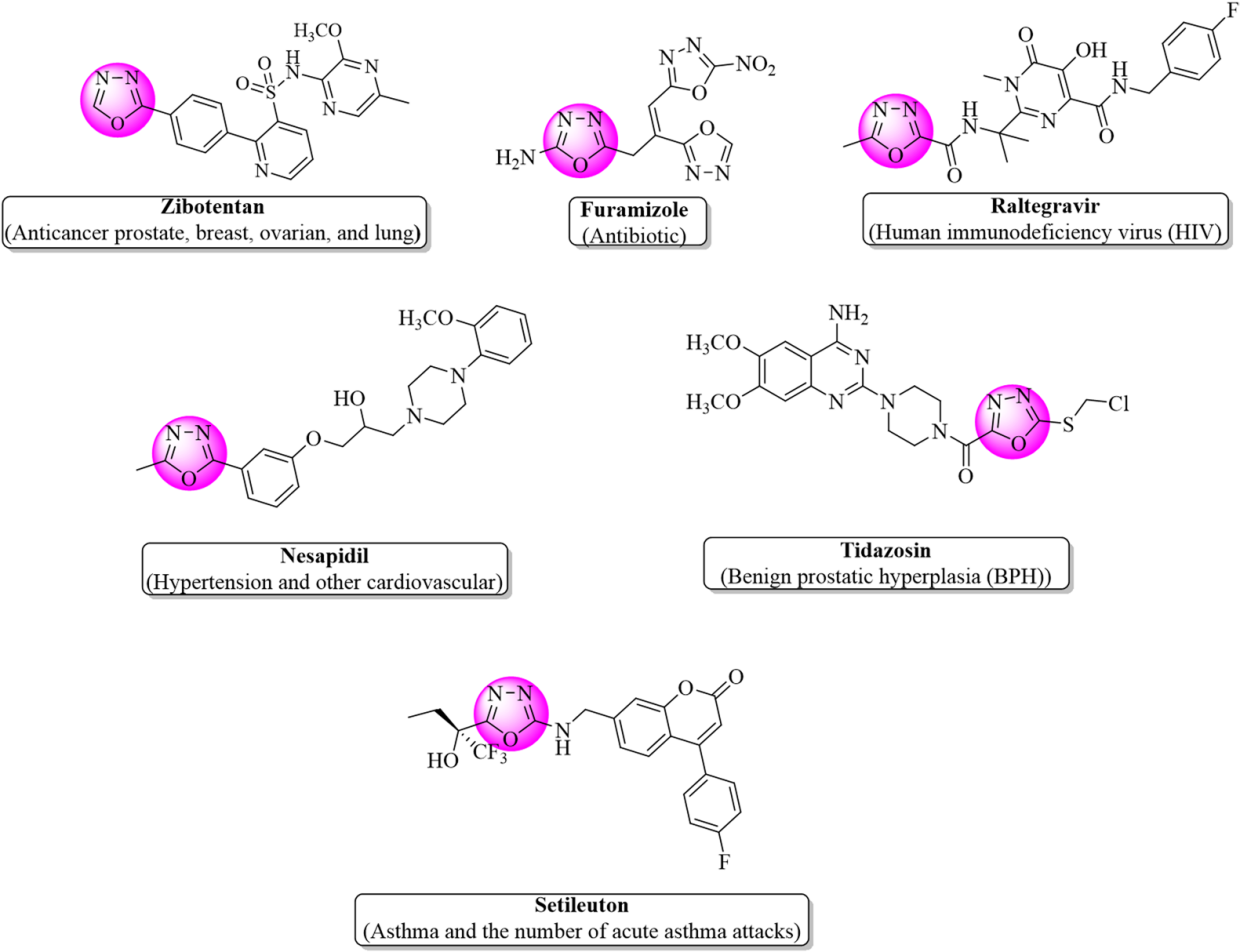
Pharmaceutically recommended oxadiazole-based compounds used as drugs against different diseases.

**Fig. 2:**
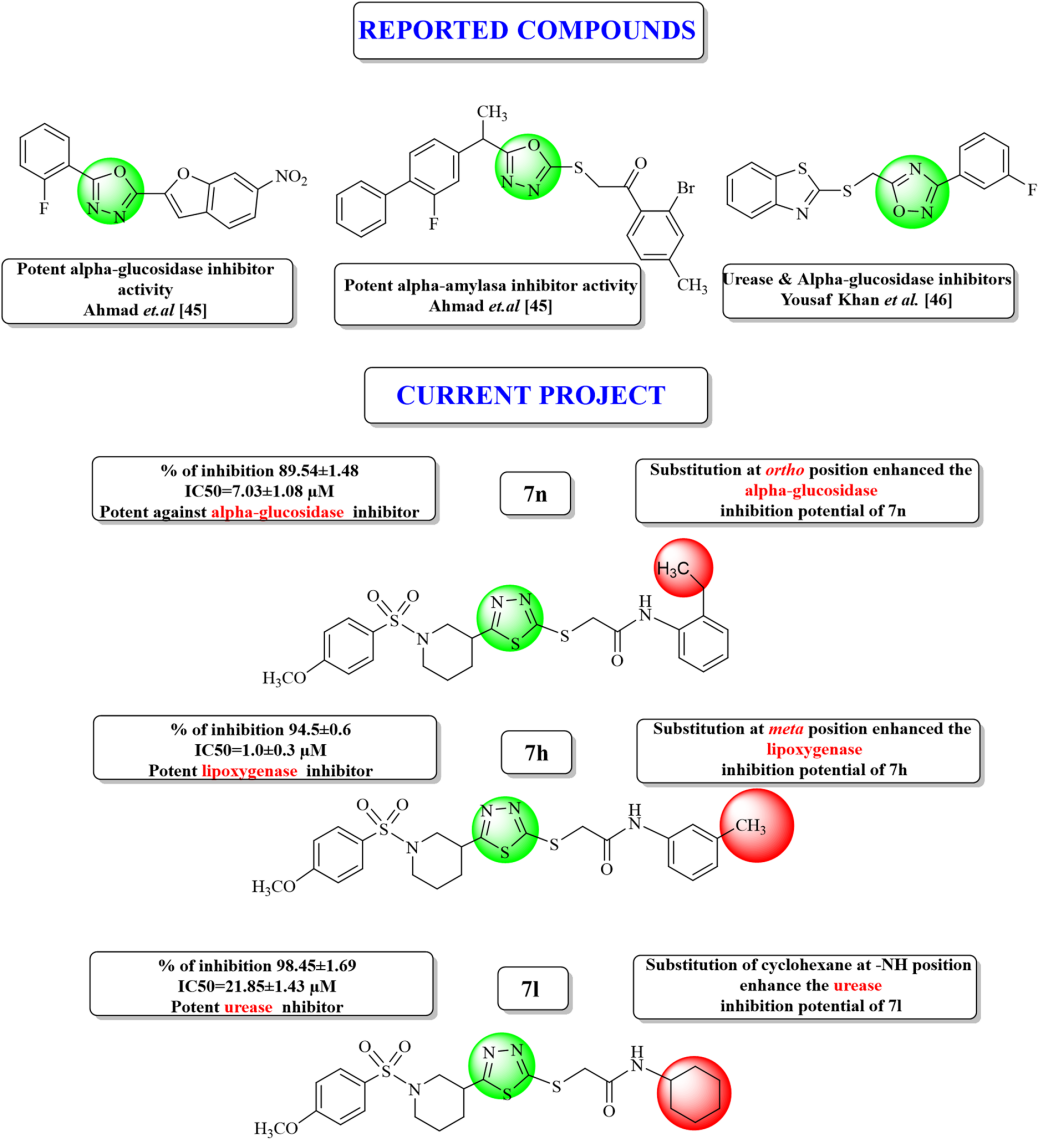
Rationale of the current study with reported compounds and best compounds in the current research.

Heterocyclic compounds containing oxygen and nitrogen are widely used in a variety of medicines to treat different diseases, e.g., cancer, gastric ulcer, bacterial and fungal infections^27^. All the medications for variety of disorders are based on heterocyclic compounds, and the oxadiazole moiety (a heterocyclic nucleus) is the most encountered one. Several important medicines containing oxadiazole moiety as a main core currently marketed against different diseases are anti-hypertension nesapidil, furamizole acting as a strong PDF inhibitor, raltegravir acting as a good inhibitor of HIV integrase, tidazosin (BPH) and the anti-cancer agent zibotentan^28,29^ as given in Fig. 1. It is also well known that several oxadiazoles are found to have good inhibition potential against monoamine oxidase and against lipoxygenase at the nanomolar level^30,31^. Oxadiazole-containing compounds have demonstrated a wide range of biological applications and served humanity as antitumor^32^, antioxidant^33^, anti-inflammatory^34^, antimalarial^35^, anticonvulsant^36^, antifungal^37^, antibacterial^38^, antidiabetic^39^, antileishmanial^40^ and HIV^41^ (see Figs. 1 and 2 for examples of pharmaceutically recommended oxadiazole-based compounds). Compounds containing piperidine nucleus (another heterocyclic core) possess important biological potential as antibacterial^42^, anti-inflammatory, and anti-convulsant drugs^43^. Low toxicity of piperidine helps in manifestation of biological properties of designed drugs^1^.

The current research was designed and conducted based on the above-considered literature review. Our present research is an effort to design novel oxadiazole-based drug candidates possessing enhanced pharmacological activities and fewer side effects to treat deadly diseases and to enhance our drug discovery program. Thus, a series of 1,3,4-oxadiazole derivatives have been synthesized with subtle modifications in their molecular structures to assess their enzyme inhibition potential against LOX, urease, and α-glucosidase enzymes which cause different diseases (see discussion above). The library of the synthesized compounds was characterized experimentally using various spectroscopic techniques, screened for their anti-enzyme activity, and studied computationally to discuss the structure–activity relationship (SAR) in drug development and designing pathway.

## Results and discussion

### Synthetic procedures and conditions

**Scheme 1:**
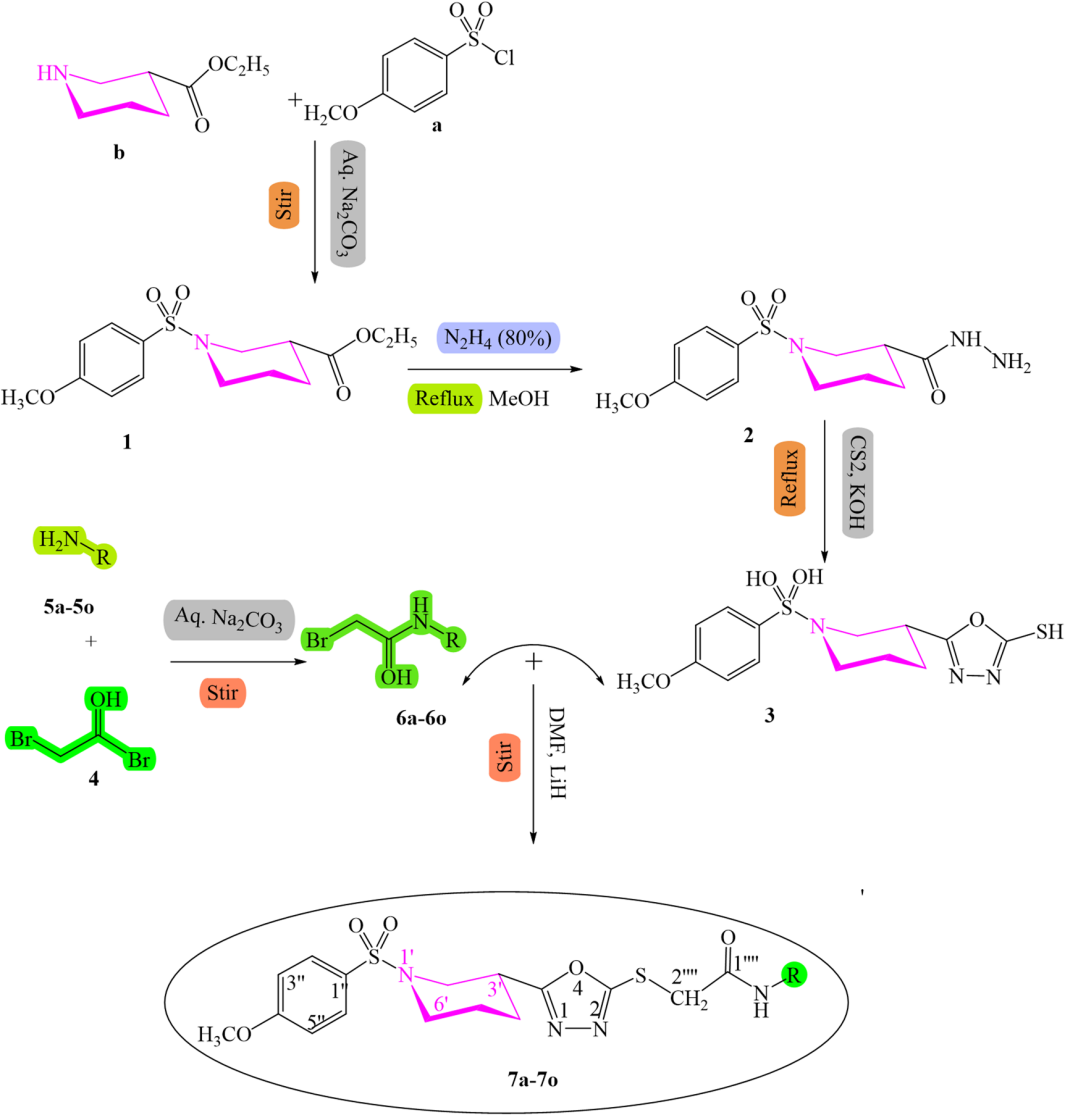
Synthetic route for the synthesis of *N*-alkyl/aralkyl/aryl acetamide analogues of 1,3,4-oxadiazole; **reaction conditions and reagents**: (I) 5% Na_2_CO_3_ soln., pH = 8–10, stirring for 12 h. (II) N_2_H_4_, CH_3_OH, reflux for 2 h. (III) CS_2_, KOH, reflux for 2 h. (IV) 5% Na_2_CO_3_ soln., pH = 8–10, stirring for 1 h. (V) LiH, DMF, stirring for 6 h.

The synthetic procedures and conditions designed to produce the desired novel compounds are outlined in Scheme 1. In the first phase, the synthetic process commenced by the synthesis of ethyl-1-[(3-methoxyphenyl sulfonyl]piperidine-3-carboxylate **(1)** through mixing of 4-methoxy benzene sulfonyl chloride **(a)** and ethyl nipecotate **(b)** in ethanol under reflux; the reaction product **1** was further solubilized in the methanolic solution of hydrazine hydrate under constant stirring to obtain respective carbohydrazide **(2)**. Compound **2** was further refluxed with methanolic solution of the equimolar amount of CS_2_ and KOH to produce an intermediate which was cyclized in the acidic medium to obtain 5-[1-(4-methoxyphenyl)sulfonyl)piperidine-3-yl]-1,3,4-oxadiazole-2-thiol **(3).**

In the second phase, the substituted aromatic amines (**5a-5o)** were treated with 2-bromoacetyl bromide (**4**) under vigorous shaking in alkaline solution (Na_2_CO_3_, 10%) for 30 to 40 min to produce a series of electrophiles, *N*-(substitutedphenyl)-2-bromoacetamides (**6a**-**6o)**.

Finally, the novel compounds, *N*-alkyl/aralkyl/aryl acetamide derivatives of 1,3,4-oxadiazole (**7a-7o)** given in Table 1 were obtained by reacting **(3)** in DMF (amount required to just solubilize) with different substituted acetamide electrophiles (**6a-6o)** using LiH as a base catalyst. The structural elucidation of the compounds obtained was performed by the ^1^H NMR, ^13^C NMR, EI-MS, and IR spectral techniques.

**Table 1:**
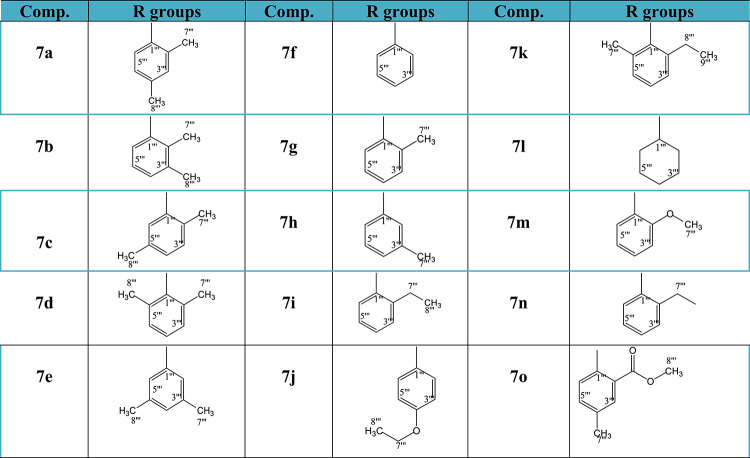
Various substituents (alkyl, aralkyl/aryl groups) in the compounds 7a-7o.

**Fig. 3:**
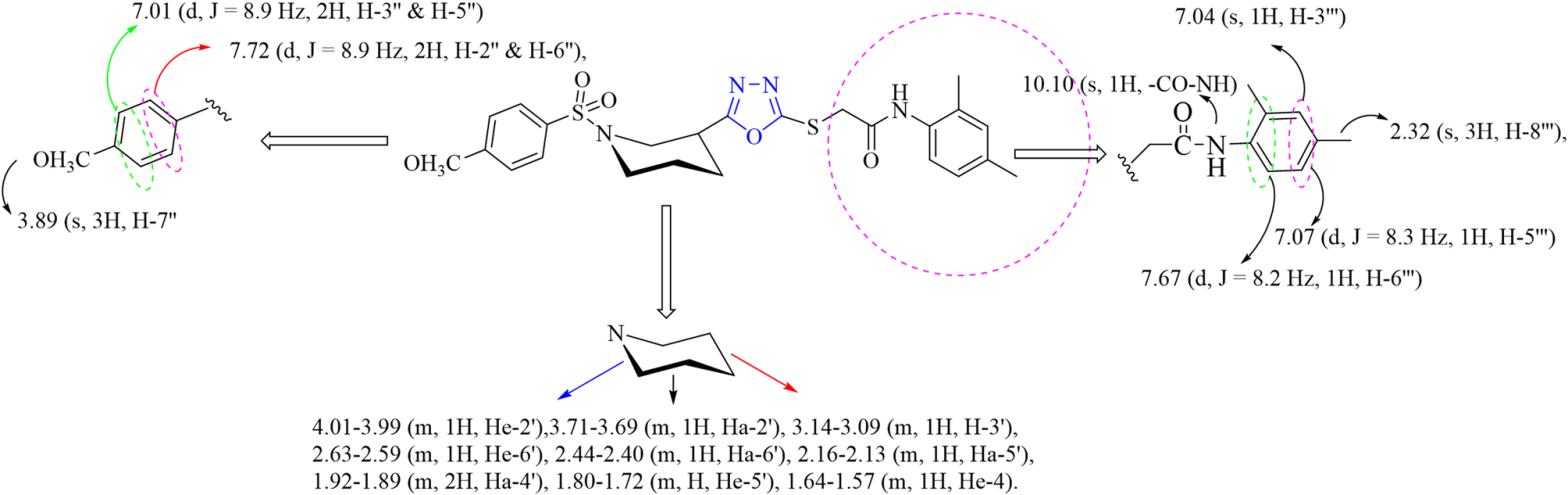
Chemical shifts of compound **7a** with elaboration of detailed structural elucidation.

One of the compounds (**7a**) of the novel series is considered hereby in detail for the rationality of the research scholars. It was acquired in solid form as light orange powder with 74% yield and melting point range 185–187°C. The molecular mass of the compound was accounted by molecular ion peak at *m/z* 509 in EI-MS spectrum and molecular formula (C_24_H_28_N_4_O_5_S_2_) was corroborated by counting protons in its ^1^H NMR spectrum. IR spectrum demonstrated the presence of major functional groups. Thus, peaks were observed at 1348 (-SO_2_ str.), 1515 (Ar C = C str.), 1572 (C = N str.), 3044 (Ar-H str.), 1234, 1076 (C-O-C str.), 3346 (N-H str.), and 1666 (C = O str.) confirming the different functionalities present in the compound. ^1^H NMR spectrum revealed two doublets at 7.72 (d, *J* = 8.9Hz, 2H, H-2’’ and H-6’’) and 7.01 ppm (d, *J* = 8.9 Hz, 2H, H-3’’ and H-5’’) which are allocated to four protons of 4-methoxy benzene ring presented in Fig. 3. Signal resonating at 3.89 ppm (s, 3H, H-7’’) was assigned to proton of methoxy group (Fig. 3). Nine multiplet signals appearing at 4.01–3.99 (m, 1H, H*e*-2’), 3.71–3.69 (m, 1H, H*a*-2’), 3.14–3.09 (m, 1H, H-3’), 2.63–2.59 (m, 1H, H*e*-6’), 2.44–2.40 (m, 1H, H*a*-6’), 2.16–2.13 (m, 1H, H*a*-5’), 1.92–1.89 (m,2H, H*a*-4’), 1.80–1.72 (m, H, H*e-*5’), and 1.64–1.57 ppm (m, 1H, H*e*-4) were allocated to protons of the piperidine ring (Fig. 3). Signals resonating at 2.32 (s, 3H, H-8’’’) and 2.31 ppm (s, 3H, H-7’’’) represented the presence of methyl groups attached to the pyridine ring. Three signals appearing at 7.67 (d, *J* = 8.2 Hz, 1H, H-6’’’), 7.07 (d, *J* = 8.3 Hz, 1H, H-5’’’), and 7.04 ppm (s, 1H, H-3’’’) were allocated to protons of the pyridine ring. ^13^C NMR spectra showed the highest peak at 163.11 ppm (C-1’’’’) which is assigned to the quaternary carbon of the carbonyl group. The second highest peak, resonating at 161.56 ppm (C-5), was allocated to the quaternary carbon of the oxadiazole group. The peak appearing at 55.61 ppm (C-7’’) confirmed the presence of the methoxy group. Peaks at 160.43 (C-4’’), 131.38 (C-1’’), 129.74 (C-2’’ and C-6’’), and 114.34 ppm (C-3’’ and C-5’’) were ascribed to carbons of the 4-methoxy benzene moiety. The quaternary carbon of the oxadiazole moiety attached to sulfur gave a signal at 160.83 ppm (C-2). Two methyl groups attached to the pyridine moiety gave signals at 20.71 (C-8’’’) and 17.63 ppm (C-7’’’). Carbons of the piperidine ring gave signals at 52.83 (C-2’), 48.61 (C-6’), 33.60 (C-3’), 27.28 (C-4’), and 23.87 ppm (C-5’). Carbons belonging to the pyridine resonate at 133.65 (C-4’’’), 133.29 (C-2’’’), 127.69 (C-1’’’), 126.97 (C-3’’’), 126.22 (C-5’’’), and 119.66 ppm (C-6’’’). The peak appearing at 46.27 ppm (C-2’’’’) was assigned to the carbon attached to the carbonyl group and sulfur atom. So, based on the aforementioned evidence, the structure of **7a** was named as *N*-(2,4-dimethylphenyl)-2-((5-(1-((4-methoxyphenyl)sulfonyl)piperidin-3-yl)-1,3,4-oxadiazol-2-yl)thio)acetamide. Similar strategies were employed for the structural verification of all the synthesized molecules.

### Biological studies

#### Enzyme Inhibition structure-activity relationship (SAR)

Minor changes in the structure of a molecule lead to great modifications in its pharmacological activities^50^, therefore a library of compounds was designed and synthesized with differences in positions and nature of substituents attached to the parent compound **3**. Tables 2 and 3 demonstrate the results for newly synthesized compounds regarding their inhibition of lipoxygenase, α-glucosidase, and urease enzymes in terms of % of inhibition and IC_50_ (concentration for 50% inhibition).

#### Lipoxygenase Inhibition structure-activity relationship (LI-SAR)

**Fig. 4:**
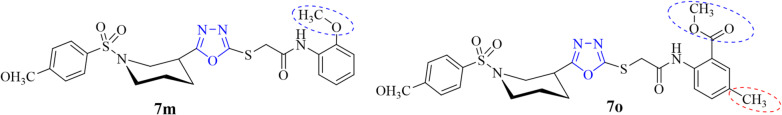
SAR of compounds **7m** and **7o** with least inhibition activity against lipoxygenase enzyme.

Iron-containing enzymes called lipoxygenases (LOXs) catalyze the oxygenation of fatty acids that are polyunsaturated, including arachidonic acid and linoleic acid, which results in the production of hydroperoxides. These enzymes are essential for the development of neurological disorders, inflammation, and malignancy. LOXs are categorized depending on their suitability for oxygenation locations; 5-LOX, 12-LOX, and 15-LOX enzymes have been extensively investigated. The potential of the entire synthesized collection of compounds was assessed using the IC_50_ values and percentage of inhibition compared to quercetin employed as a standard. The results are collected in Tables 2 and 3. Among the synthetically produced series of compounds, compounds **7m** and **7o** showed the lowest potential, with respective IC_50_ values of 105 ± 0.05 and 103.26 ± 1.09 and inhibitions of 35.6 ± 0.7 and 37.5 ± 0.4%, respectively. The presence of *N*-(2-methoxylphenyl)acetamide group in **7m** and *N*-(2,5-dimethylbenzoate)acetamide group in **7o** may be the cause of this lowered potential (see Fig. 4). A few interactions of **7m** with amino acid residues are classified as hydrophobic, Pi-Sigma, Alkyl, and Pi-Alkyl types (Figure S58 given in SI file). Interaction are weak (not well engaging the lipoxygenase enzyme) distances range from 3.59 Å to 4.97 Å with binding affinity was 7.1 kcal/mol making **7m** least inhibitor. The docking studies reveal that compound **7o** with the moderate binding affinity shows interactions with enzyme (Fig. 14C), but these interactions might not be in the specific active site which makes this compound the least active. It is important to note that the interaction of a substrate with enzyme in right orientation is specific in finding the potential of any compound.

**Fig. 5:**
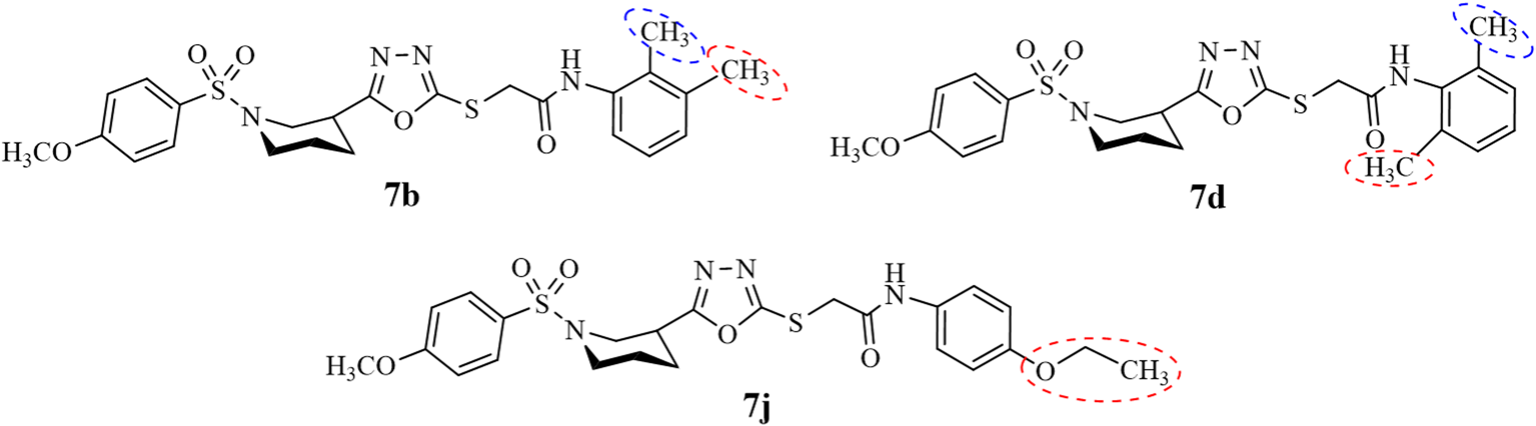
SAR of compounds **7a**,** 7d**, and **7j** with moderate inhibition activity against lipoxygenase enzyme.

Compounds **7b**,** 7d**, and **7j** showed comparable potential against the lipoxygenase enzyme as given in Tables 2 and 3. The IC_50_ values of Compounds **7b**,** 7d**, and **7j** are 4 ± 1.05, 5 ± 0.05 and 3.3 ± 0.3 respectively. Compounds **7b** and **7d** both have double substitutions at *ortho* and *meta* position for **7b** while at *ortho* position at amidic aromatic ring as presented in Fig. 5. But compound **7j** showed little better potential than **7b** and **7d**, the difference might be due to electron donating substitution at **7b** and **7d** while electron withdrawing substitution at **7j**. The overall bonding pattern and bonding orientation of **7b** are moderate, number of hydrogen bonds observed as shown in Figure S47 given in SI file. Conventional hydrogen bond observed with THR364 at 3.25 Å. Carbon hydrogen bond with ILE673 at 3.43 Å and Pi-sigma interaction with LEU607 at 3.70 Å. The overall these interaction leads to its moderate potential against lipoxygenase enzyme. Similarly, **7d** showed intermediate level of interaction with substrate as Pi-Donor Hydrogen interactions with GLN611 at 3.59 Aͦ, Pi-Pi Stacked and Alkyl interactions with PHE169 and PRO621 at 3.65 and 4.22 Aͦ respectively presented in Figure S49, which suggest its comparable potential to inhibit lipoxygenase enzyme. **7j** on the same pattern have very few interactive sites to inhibit the action of lipoxygenase. **7j** showing very few interactions with large bond distance confirming it to be moderate inhibitor of lipoxygenase as Pi-Sigma interaction LEU368 at 3.86 Å, Pi-Pi Stacked, Pi sulfur and Pi-Alkyl with PHE177, TYR181 and given in Figure S55.

**Fig. 6:**
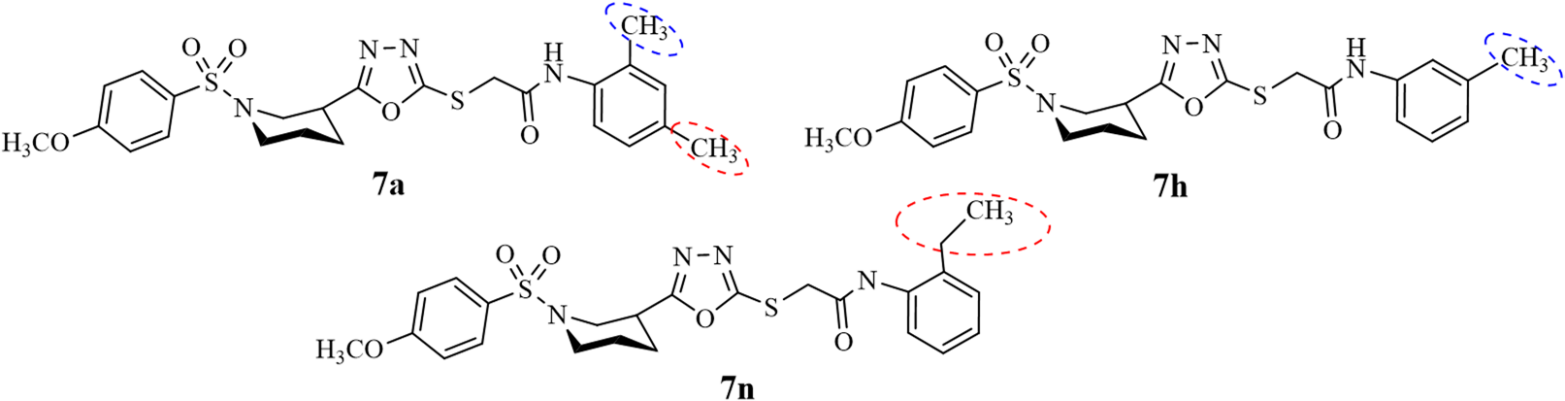
SAR of compounds **7a**,** 7h**, and **7n** with highest inhibition activity against lipoxygenase enzyme.

Compounds **7a**,** 7h**, and **7n** were found to be most potent among the whole library of synthesized compounds with % of inhibition 92.2 ± 0.5, 94.5 ± 0.6, and 92.8 ± 0.8, respectively, and IC_50_ values 3 ± 0.24, 1.0 ± 0.3, and 1.5 ± 0.5, respectively. Additional research on these substances could validate their variable potential and make compounds **7a**,** 7h**, and **7n** with variety of structural variabilities (Fig. 6) effective medications that will compete with quercetin. Conventional hydrogen bond interaction of compound **7a** observed with GLN363 and THR364 at 3.36Å and 3.25 Å while carbon-hydrogen bond with ILE673 at 3.45 Å. Pi-sigma interaction with LEU607 at 3.62 Å and Pi-pi T shaped interaction observed with HIS432 at 4.71 Å justify the better interaction of **7a** with substrate which in turns make it good inhibitor of lipoxygenase as shown in Figure S46 given in SI file. Similarly, **7n** showed the carbon-hydrogen and Pi-Donor hydrogen with GLN15 and ASN613 at 3.63 and 3.89 Aͦ respectively. The hydrophobic interactions in Pi-Sigma and Pi-Alkyl with SER171 and PHE402 at 3.99 and 4.96 Aͦ are for **7n**. At a bond distance of 4.02 Aͦ, one Pi-Sulfur interaction with PHE169 makes **7n** better inhibitor of lipoxygenase as given in Figure S59 given in SI file. On the other hand, the docking studies suggest that the binding affinity value (7.5 kcal/mol) of the synthesized compound **7 h** is a little higher. The greater number of hydrophobic interactions with amino acids (Fig. 14) were observed with smaller bond distances compared to the standard thus making **7h** more potent among the synthesized series of compounds and standard quercetin. Variety of hydrophobic interactions were including alkyl-alkyl and π–alkyl with amino acid residues LEU373, ARG411, TRP147, PHE151, TYR181, ALA603, and TRP599, were observed with distances ranging from 3.85 to 5.45 Å, indicating a strongly hydrophobic environment that supports ligand binding. Based on interaction of ligand-substrate and binding affinity the results strongly support its better potential against lipoxygenase enzyme.

#### α-Glucosidase Inhibition structure-activity relationship (α-GI-SAR)

**Fig. 7:**
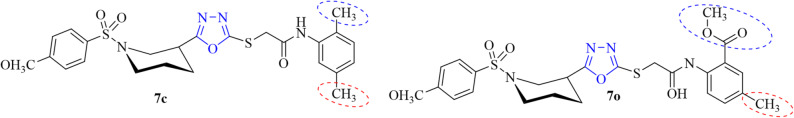
SAR of least active compounds **7c** and **7o** against alpha glucosidase enzyme.

The digestion of complex starch and series of disaccharides to monosaccharides may be achieved through the action of α-glucosidase while its enhanced activity is the source of high blood sugar level which is the ultimate reason of diabetes. The potential of the whole synthesized library of compounds for inhibition of α-glucosidase was investigated in terms of % of inhibition and IC_50_ values with reference to the acarbose used as a standard as presented in Tables 2 and 3. Two compounds, **7c** and **7o**, showed the lowest potential among the synthesized series of compounds having 45.42 ± 1.59 and 52.65 ± 1.78% of inhibition with 92.08 ± 1.14 and 86.16 ± 1.18 IC_50_ values, respectively. This decreased potential might be due to the presence of electron-donating substituents in *ortho*,* meta* and *para* positions in both compounds as shown in Fig. 7. Compound **7c** showing the least binding energy among the synthesized series of compounds was found to be the least active. The binding energy value justifies that its interaction with α-glucosidase is not appropriate thus making it the least efficient. Similarly, the potential of compound **7o** against alpha glucosidase enzyme was also very reduced with reduced binding interaction (binding affinity of -7.3 kcal/mol) and with increased bond distance between **7o**-substarte as given in Figure S89.

**Fig. 8:**
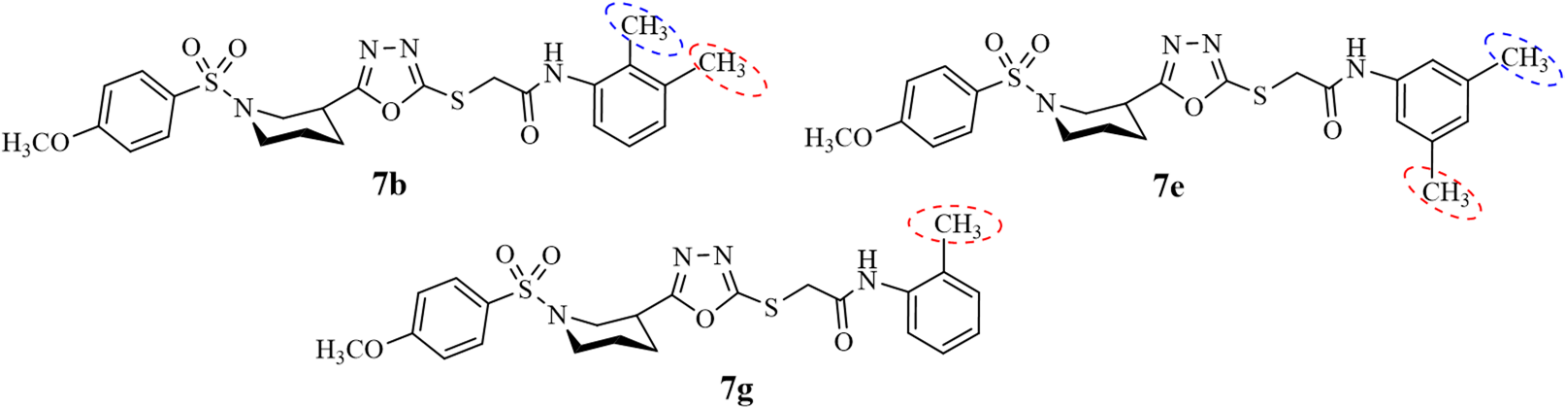
SAR of compounds **7b**,** 7e** and **7g** with moderate inhibition activity against alpha glucosidase enzyme.

Compounds **7b**,** 7e**,** 7g**,** 7h**,** 7j**, and **7m** are found to be comparable with the standard in terms of % inhibition and IC_50_ values. The most common thing is the substitution of methyl group to the amidic aromatic ring at ortho position making all the compounds moderate inhibitor of lipoxygenase. The further variation in their potential might be due to the second methyl substitution as presented in Fig. 8. The binding interaction of compounds **7b**,** 7e**,** 7 g**,** 7h**,** 7j**, and **7m** are better with binding affinities − 8.0, -7.1, -6.4, -7.9 and − 7.7 kcal/mol contributing to stabilizing the ligand orientation and interaction with amino acid residues as compared with **7c** and **7o** and making them better inhibitor of alpha glucosidase enzyme.

**Fig. 9:**
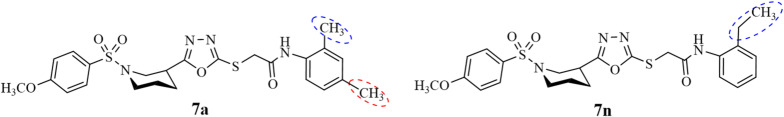
SAR of compounds **7a** and **7n** with best inhibition activity against alpha-glucosidase enzyme.

On the contrary, **7a**,** 7f**,** 7i**,** 7l**, and **7n** have the highest % of inhibition (89.42 ± 1.33, 78.79 ± 1.29, 84.87 ± 1.53, 75.67 ± 1.62, and 89.54 ± 1.48, respectively) and the least IC_50_ values (7.15 ± 1.34, 14.26 ± 1.07, 9.12 ± 1.17, 14.31 ± 1.19, and 7.03 ± 1.08, respectively) against α-glucosidase enzyme. The enhanced potential of both **7a** and **7n** might be due to the substitution of methyl and ethyl groups at *ortho* position, respectively, while the least active compounds have methyl group substitution at *meta* and ester at *ortho* positions (Fig. 9). This might be the source of potential difference among the least active compounds and the highest potential compounds. Further studies on these compounds may confirm their enhanced potential and might bring compounds **7a** and **7n** as active drugs in the market against α-glucosidase. The highest binding affinity and greater number of π-alkyl interactions making them more active against α-glucosidase. The binding orientation of compound **7n** might be with more active site of the enzyme with the least bond distances which enhances selectivity, specificity of inhibition, minimizing the off-target effect and blocking the enzyme mechanism of action that might results in the best potential of compound **7n**.

#### Urease Inhibition activity structure-activity relationship (UI-SAR)

The hydrolysis of urea results in production of carbon dioxide and ammonia. Urea is found in blood serum, in the stomach, and perspiration. As urease activity increases it results in stomach and urinary tract ulcers which might ultimately change into cancer. The increased activity of the urease enzyme might be the source of high blood pressure, which has detrimental effects on human health. The inhibition potential of all compounds was evaluated in terms of percentage of inhibition and IC_50_ values relative to thiourea used as a standard presented in Tables 2 and 3. The whole library of compounds was found to be active with variable potentials with percentage of inhibition ranging from 51.26 ± 1.39 to 98.45 ± 1.69 and IC_50_ values ranging from 120.14 ± 1.43 to 21.85 ± 1.43, from minimum to maximum potential, respectively.

**Fig. 10:**
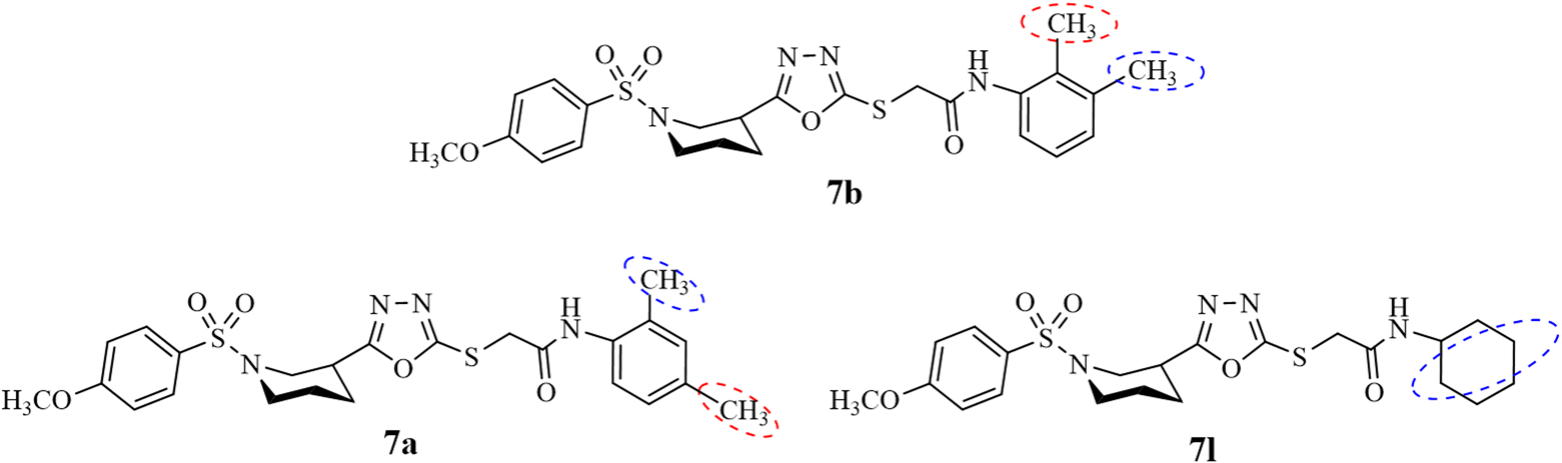
SAR of compounds **7b** (least active) **7a** and **7l** (most active) against urease enzyme.

Compound **7b** (% of inhibition of 51.26 ± 1.39% and 123.15 ± 1.24 IC_50_) shows the lowest potential with because of substitution at *ortho* and *meta* position of aromatic amidic ring as shown in Fig. 10 which might have least interaction or may not well accommodated ligand-substrate orientation. Due to the weakest hydrophobic interactions (Fig. 14I), the interactions might occur to the non-active sight, to an allosteric or non-functional site have made **7b** least active against urease enzyme. Other factors may be the metabolic instability and ligand suboptimal orientation to the active site which results in its lowest potential.

On the other hand, compounds **7a** and **7l** having electron-rich functionalities attached to the phenyl ring of acetamide in **7a** and cyclohexane in **7l**, respectively (Fig. 10), revealed excellent inhibitory activity with % of inhibition 92.35 ± 1.65 and 98.45 ± 1.69, respectively. With the IC_50_ value of 21.85 ± 1.43 µM and the percentage inhibition of 98.45 ± 1.69, compound **7l** was found to be more active in comparison to the standard drug thiourea which has the IC_50_ value 21.25 ± 0.15µM and the % of inhibition of 96.24 ± 0.16. Compound **7l** made interactions with active site of protein (PDB ID: 3la4). Three carbon-hydrogen bonds were observed with ASP633, ASP633, and ALA636 at 3.52 Å, 3.61 Å, and 3.56 Å, respectively. A π-donor hydrogen bond involving MET637 at of 3.88 Å. Hydrophobic interactions include ALA636, ARG639, HIS409, HIS492, and HIS519 at 4.30 Å, 5.32 Å, 4.84 Å, 5.26 Å, and 5.35 Å, respectively. Several π-alkyl interactions with MET588, ALA636, MET637 were observed at 4.76 Å, 4.86 Å, and 4.58 Å, respectively contributed to stabilizing the ligand orientation. The compound occupied a deep catalytic cleft, demonstrating robust interactions comparable to known inhibitors. High binding affinity of compound **7l** confirms its better potential to block the active site of the urease enzyme (Fig. 14H) which also enhances selectivity, specificity of inhibition, and minimizes the off-target effect.

**Table 2:** Enzyme inhibitor activities of the 1,3,4-oxadiazole derivatives expressed as % of inhibition.

Compounds	LOX Inhibition (%) at 0.25 mM	α-Glucosidase Inhibition (%) at 0.5 Mm	Urease Inhibition (%) at 0.25 mM
**7a**	92.2 ± 0.5	89.42 ± 1.33	92.35 ± 1.65
**7b**	85.3 ± 0.3	67.35 ± 1.21	51.26 ± 1.39
**7c**	47.5 ± 0.8	45.42 ± 1.59	75.35 ± 1.52
**7d**	82.4 ± 0.6	72.35 ± 1.45	76.28 ± 1.43
**7e**	75.4 ± 0.6	66.38 ± 1.72	62.41 ± 1.57
**7f**	78.4 ± 0.7	78.79 ± 1.29	58.26 ± 1.45
**7g**	53.2 ± 0.4	67.54 ± 1.67	68.72 ± 1.58
**7h**	94.5 ± 0.6	62.35 ± 1.79	53.26 ± 1.35
**7i**	45.3 ± 0.6	84.87 ± 1.53	72.54 ± 1.45
**7j**	85.4 ± 0.7	63.25 ± 1.34	77.63 ± 1.23
**7k**	62.1 ± 0.6	56.49 ± 1.64	86.42 ± 1.55
**7l**	75.2 ± 0.4	75.67 ± 1.62	98.45 ± 1.69
**7m**	35.6 ± 0.7	63.84 ± 1.27	72.16 ± 1.45
**7n**	92.8 ± 0.8	89.54 ± 1.48	87.12 ± 1.76
**7o**	37.5 ± 0.4	52.65 ± 1.78	73.52 ± 1.63
**Quercetin**	89.2 ± 0.6	-	-
**Acarbose**	-	65.73 ± 1.93	-
**Thiourea**	-	-	98.21 ± 0.78

**Table 3:** IC_50_ values of the 1,3,4-oxadiazole analogues studied for LOX, urease and α-Glucosidase potential.

Compound	IC_50_ (µM)		
	LOX	α-Glucosidase	Urease
**7a**	3 ± 0.24	7.15 ± 1.34	26.64 ± 1.32
**7b**	4 ± 1.05	36.54 ± 1.13	123.15 ± 1.24
**7c**	28.01 ± 0.03	92.08 ± 1.14	38.06 ± 1.28
**7d**	5 ± 0.05	17.21 ± 1.35	36.09 ± 1.35
**7e**	42.5 ± 0.3	37.18 ± 1.24	98.16 ± 1.72
**7f**	8.2 ± 0.4	14.26 ± 1.07	108.50 ± 1.07
**7g**	26 ± 1.16	36.21 ± 1.04	40.41 ± 1.36
**7h**	1.0 ± 0.3	39.56 ± 1.37	120.14 ± 1.43
**7i**	30 ± 1.02	9.12 ± 1.17	39.05 ± 1.31
**7j**	3.3 ± 0.3	38.01 ± 1.25	33.09 ± 1.02
**7k**	1.02 ± 0.04	45.56 ± 1.36	28.12 ± 1.04
**7l**	16 ± 0.01	14.31 ± 1.19	21.85 ± 1.43
**7m**	105 ± 0.05	38.05 ± 1.26	39.03 ± 1.78
**7n**	1.5 ± 0.5	7.03 ± 1.08	29.25 ± 1.52
**7o**	103.26 ± 1.09	86.16 ± 1.18	39.42 ± 1.43
**Quercetin**	2.3 ± 0.3	-	-
**Acarbose**	-	37.58 ± 1.76	-
**Thiourea**	-		21.25 ± 0.35

### Computational studies

#### Energetic features

**Fig. 11:**
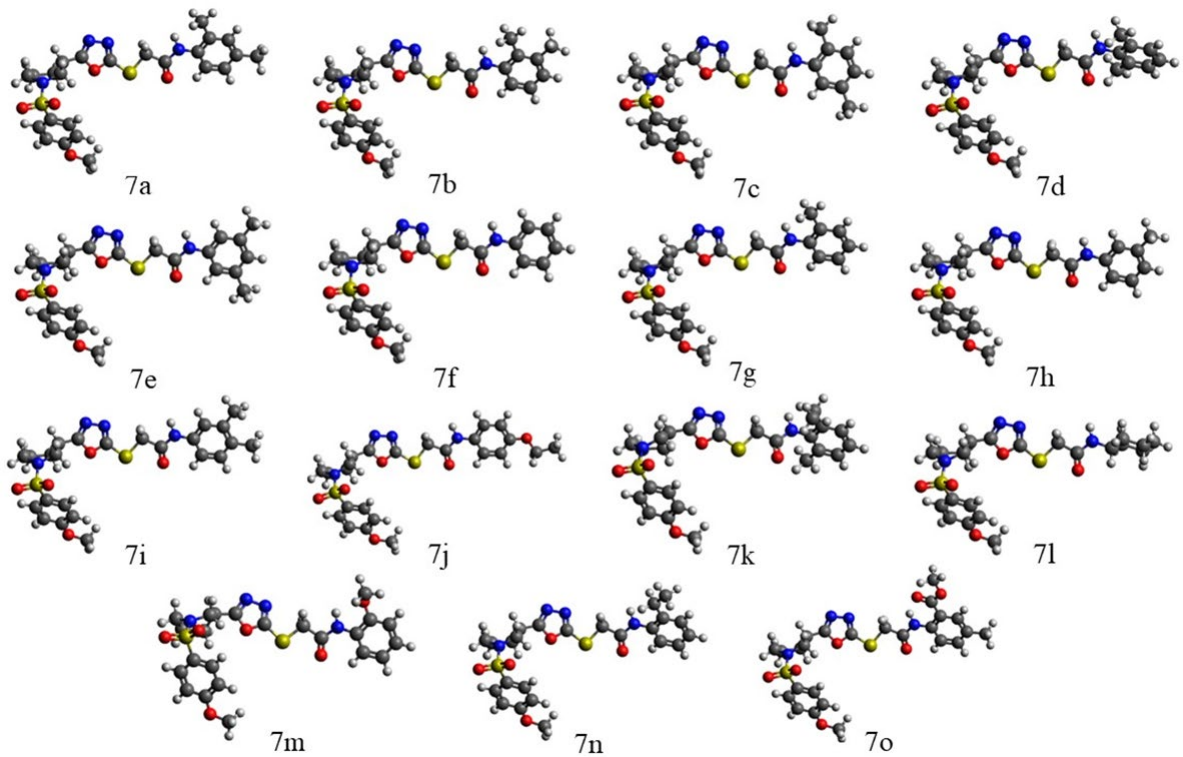
B3LYP/6-311G(d, p) optimized structures of the compounds **7a-7o** calculated with the implicit effects of DMSO.

In Table S1 the energetics parameters (electronic energies, electronic energies with zero-point corrections, HOMO/LUMO energies, and HOMO/LUMO gaps) are provided for the compounds **7a-7o** obtained using the B3LYP/6-311G(d, p) approach, both in the gas phase and with the implicit solvent effects from DMSO. Figure 11 shows the optimized structures of the compounds **7a-7o** calculated with the implicit effects of DMSO. Consideration of the optimized structures shows their noticeable similarity as well as it implies their significant flexibility. This in turn suggests that molecules of the compounds investigated can easily accommodate themselves to enzyme active sites (see also below discussion of MEP plots).

#### FMO analysis

Analysis of the data in Table S1 shows that almost all compounds studied have significant HOMO/LUMO gaps in the implicit DMSO, within 5.15ࣧ5.50 eV, only the compounds **7j**, with the 4-ethoxyphenyl group, and **7o**, with the methylbenzoate group, have smaller HOMO/LUMO gaps, 4.87 and 4.66 eV, respectively. These results show that all these compounds should have quite high thermodynamic stability.

**Fig. 12:**
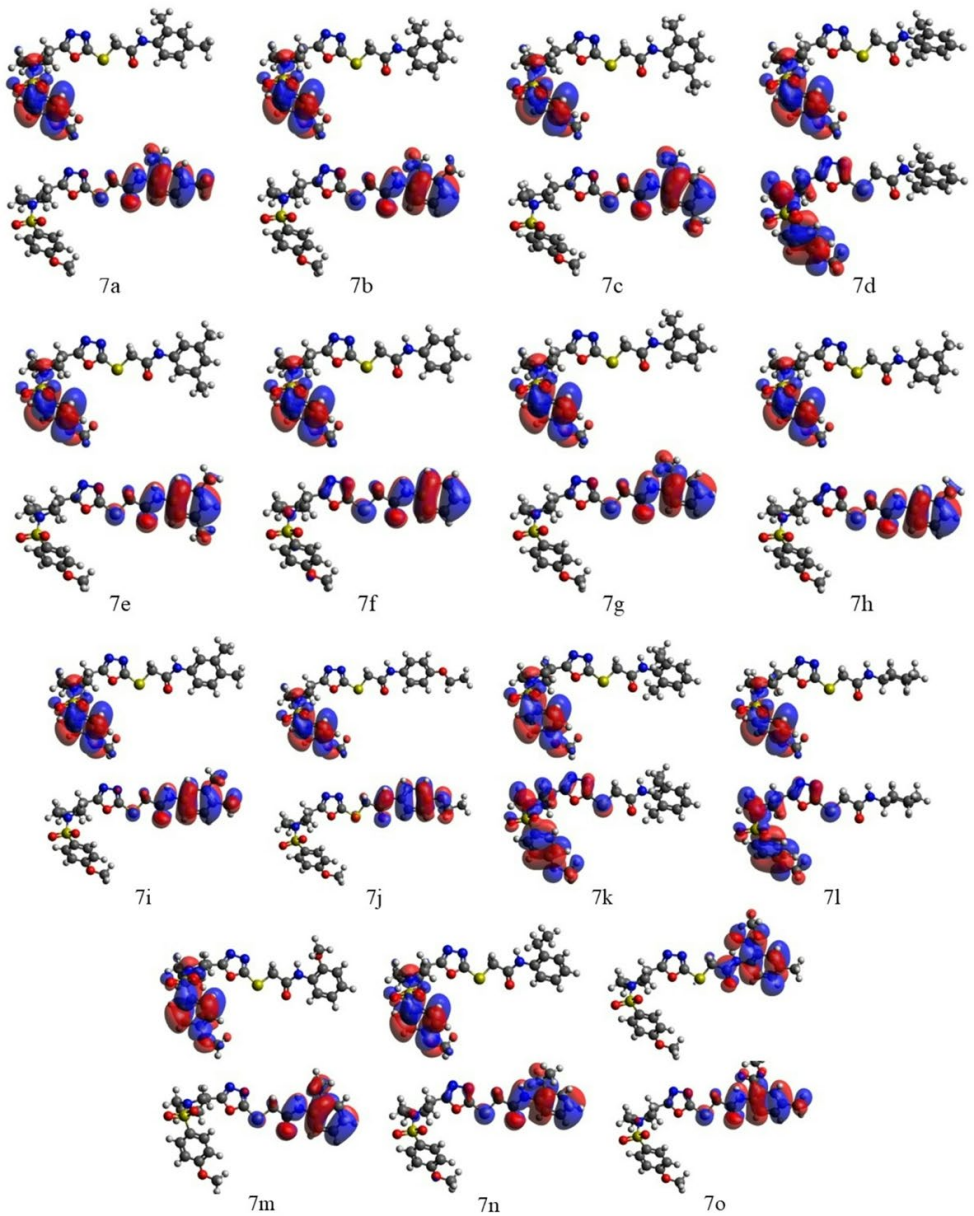
FMOs of **7a-7o** computed with the B3LYP/6-311G(d, p) approach in the implicit DMSO.

Consideration of the **7a-7o** FMOs in Fig. 12 shows that for the most part of the compounds, except for **7d**, **7k**, **7l**, and **7o**, the FMOs have similar topology: the HOMOs are dominated by the substituting group R whereas the LUMOs are dominated by the methoxyphenyl moiety and sulfoxide moiety. This provides certain qualitative support for the suggestions given above that substituting groups would determine inhibitor activity of the compounds studied. It will be necessary to make deeper analysis of the relation between the chemical structure/electronic properties of these compounds and their biological activity.

#### MEP analysis

**Fig. 13:**
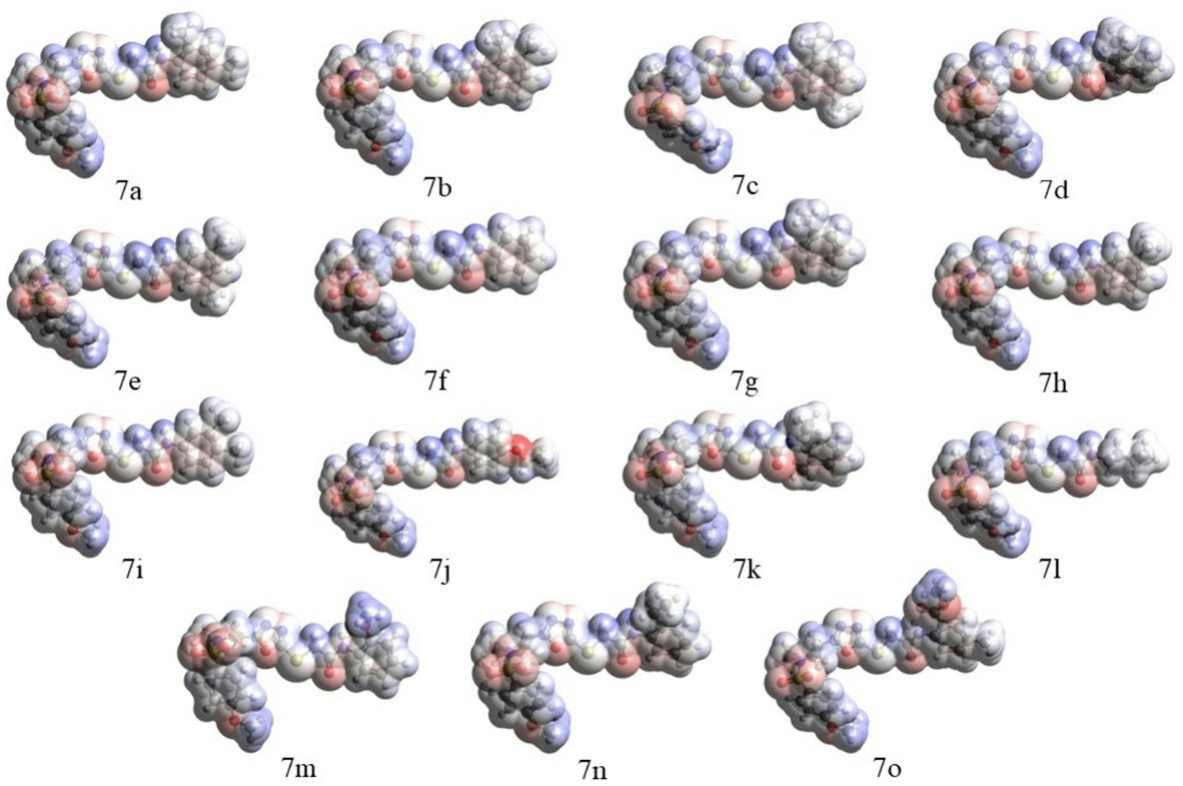
MEP plots of the compounds **7a-7o** calculated using the B3LYP/6-311G(d, p) approach with the implicit effects of DMSO taken into account.

Consideration of the MEP plots of the compounds **7a-7o** presented in Fig. 13 shows that molecules of all compounds have noticeable accumulations of both positive (as indicated by blue color) and negative (as indicated by red color) electrostatic potentials, which implies existence of various interactions of molecules of these compounds with the protein pockets/enzyme active sites as well as with polar molecules of the human body environment. This, in turn, implies that these compounds can effectively interact with the enzyme active sites. The presence of accumulations of both positive and negative potentials (or, in other words, electrophilic and nucleophilic spots) in molecules of the synthesized compounds qualitatively supports the existence of various binding interactions such as carbon-hydrogen bonds, electrostatic interactions, π-cation interactions, multiple hydrophobic alkyl-alkyl and π-alkyl interactions found in the docking studies.

### Molecular docking analysis

**Fig. 14:**
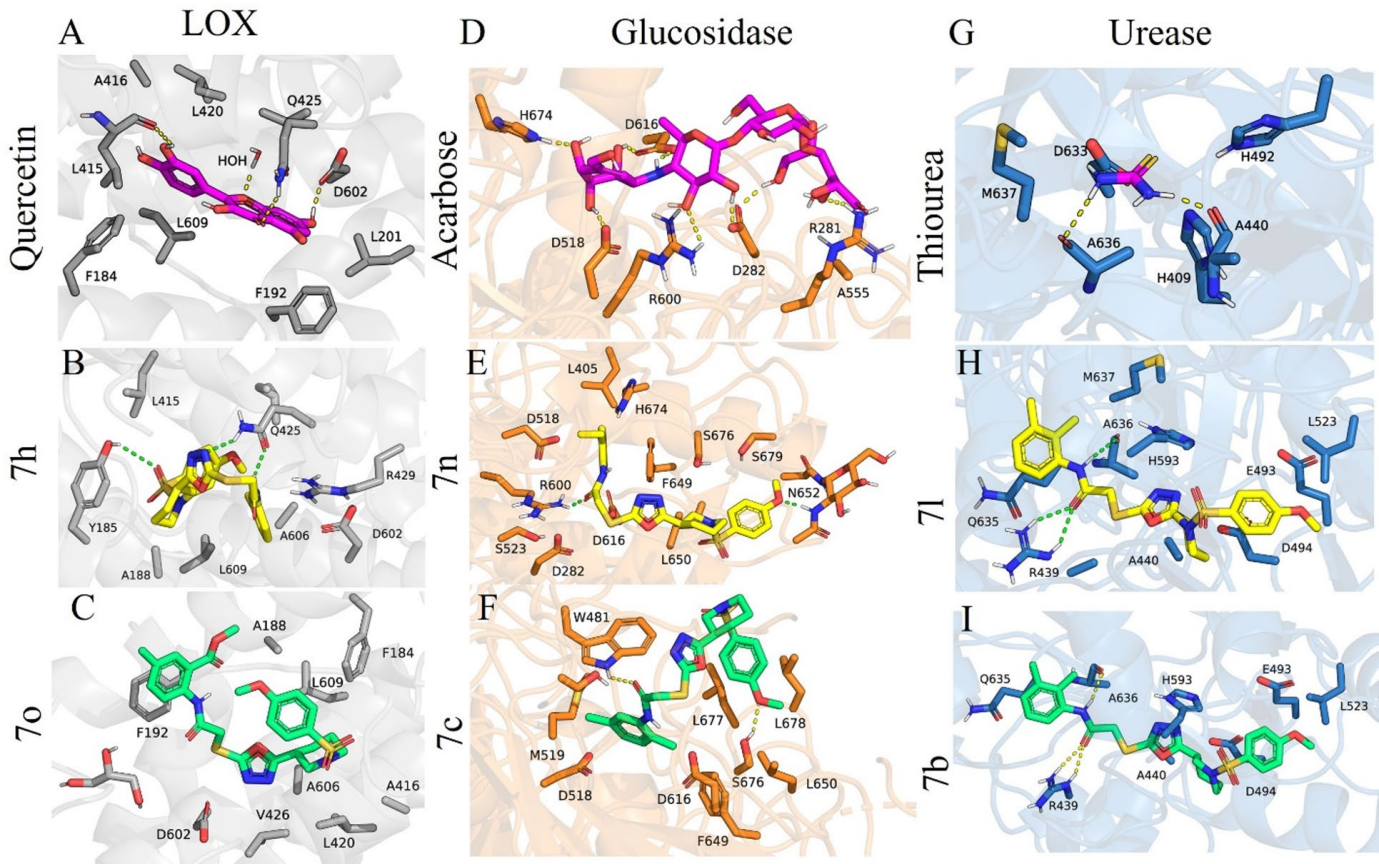
Three-dimensional docking interactions of standard inhibitors and synthesized compounds with all three target enzymes. The LOX binding poses of quercetin (A), compound 7h (B), and compound 7o (C) are shown, followed by the α-glucosidase interactions of Acarbose (D), compound 7n (E), and compound 7c (F). The urease binding conformations of Thiourea (G), compound 7l (H), and compound 7b (I) are presented for comparison.

Molecular docking studies were carried out to investigate the binding orientation of the synthesized compounds within the active sites of the target enzymes, and the resulting three-dimensional interaction diagrams are presented in Fig. 14. To get better insight, the molecular docking conformations of all ligands understudy are visually depicted in Figures S46-S82 of the Supporting Information. Moreover, to elucidate the comprehensive binding patterns and atomic-level interactions, the docked complexes were further analyzed utilizing the Protein–Ligand Interaction Profiler (PLIP). The resulting interaction diagrams, which detail residue-specific hydrogen bonds, hydrophobic contacts, salt bridges, and pi-interactions, are presented in Figure S83 of the Supporting Information. A comprehensive docking scores table has also been provided in the Supporting Information as Table S1, summarizing the binding affinities of all synthesized compounds and the standard drug in kcal/mol. These docking generated conformations of ligands, together with the docking score data, complement the docking results shown in Fig. 14 and strengthen the structural interpretation of the docking data in relation to the biochemical inhibition profiles.

### Docking analysis with Lipoxygenase (LOX)

The docking scores of the synthesized LOX inhibitors ranged from − 6.1 to -8.3 kcal/mol, indicating generally stronger predicted binding than the standard ligand quercetin (-6.486 kcal/mol). As shown in Fig. 14A, the standard ligand quercetin established a stable interaction framework within the LOX binding pocket. It formed hydrogen bonds with Q425, D602, and a conserved water molecule, and hydrophobic interactions with F184, F192, L415, and L609. These interactions explain its inhibitory activity and serve as a reference for evaluating the synthesized compounds.

Compound 7 h (As illustrated in Fig. 14B), the most potent LOX inhibitor in vitro, adopted a binding orientation highly consistent with quercetin. It formed hydrogen bonds with Q425 and Y185, supported by polar contacts with A606 and R429. Hydrophobic interactions with F184, F192, L419, and L609 further stabilized the complex. Importantly, 7 h achieved the most favorable docking score of -8.3 kcal/mol, which is substantially stronger than quercetin (-6.486 kcal/mol) and supports its excellent IC50 value.

Compound 7o (Fig. 14C), the weakest LOX inhibitor, displayed a shifted and suboptimal docking pose. Only weak or poorly oriented hydrogen bonds were present. Hydrophobic interactions with L419, F184, and Y185 were insufficient to compensate for the loss of strong anchoring contacts with Q425 or D602. Correspondingly, its docking score of -7.9 kcal/mol was weaker than the top-performing compounds and did not show a strong advantage over quercetin, explaining its minimal inhibitory activity.

These observations confirm that the presence of strong hydrogen bonding with Q425, together with hydrophobic stabilization, is critical for LOX inhibition. The docking outcomes align with the biochemical data, where 7 h is the most active and 7o is the least active compound, and the incorporated docking score trends further strengthen this interpretation.

### Docking analysis with α-glucosidase

Acarbose (as demonstrated in Fig. 14D), the standard α-glucosidase inhibitor, formed extensive hydrogen bonds with key catalytic residues D282, D518, D616, and H674, along with polar interactions with R600 and R281. This robust interaction network reflects its inhibitory behavior, and its docking score of -5.671 kcal/mol provides a reference point for evaluating the synthesized derivatives. The docking scores of the synthesized compounds ranged from − 6.4 to -8.1 kcal/mol, indicating generally stronger predicted binding than acarbose.

As presented in Fig. 14E, compound 7n, the most potent α-glucosidase inhibitor among the synthesized derivatives, closely replicated the binding pattern of acarbose. It formed multiple hydrogen bonds with D518, D616, S676, S679, and R600. Hydrophobic contacts with F649, L650, and L405 contributed to a highly stabilized complex. Consistent with its excellent in vitro activity, 7n showed the best docking score of -8.1 kcal/mol, clearly outperforming acarbose (-5.671 kcal/mol) and supporting its strong inhibitory effect.

As displayed in Fig. 14F, compound 7c demonstrated weak α-glucosidase inhibition and adopted a correspondingly poor docking pose. Its hydrogen bonding interactions were limited to D518 and D616, and the ligand shifted toward the outer region of the catalytic site. Hydrophobic interactions with W481, W376, and L677 were insufficient to compensate for the loss of direct catalytic engagement. In line with this weak orientation, 7c exhibited a comparatively less favorable docking score of -7.1 kcal/mol, which, although better than acarbose, is significantly weaker than the top-performing compounds such as 7n. This supports its reduced inhibitory performance.

Overall, strong α-glucosidase inhibition requires hydrogen bonding with D518 and D616 and proper orientation toward H674. Compound 7n satisfies these structural requirements while 7c does not, consistent with their experimental IC50 values and supported by their docking scores.

### Docking analysis with urease

The docking scores of the synthesized urease inhibitors ranged from − 6.7 to -7.6 kcal/mol, demonstrating substantially stronger predicted binding compared to the standard inhibitor thiourea, which showed a docking score of -3.214 kcal/mol. The standard inhibitor thiourea (As represented in Fig. 14G) formed signature hydrogen bonds with D633, A636, and H409, establishing the canonical interaction motif required for urease inhibition.

As highlighted in Fig. 14H, compound 7l, one of the most active urease inhibitors in this study, successfully reproduced these key interactions. It formed hydrogen bonds with R439, Q635, A636, and H593, and also displayed polar contacts with E493. Hydrophobic interactions with M637 and A440 further strengthened the binding stability. Consistent with its potent activity, 7l exhibited an excellent docking score of -7.6 kcal/mol, which is markedly stronger than thiourea (-3.214 kcal/mol) and supports its superior inhibitory behavior.

As outlined in Fig. 14I, compound 7b, which displayed poor urease inhibition, adopted a weak and unstable docking pose. It failed to maintain strong hydrogen bonding with catalytic residues and instead relied mainly on dispersed hydrophobic contacts with L523, A440, and E493, together with only weak interactions with R439 and A636. Reflecting this suboptimal binding pattern, 7b showed a comparatively weaker docking score of -7.1 kcal/mol, which, although better than thiourea, is significantly poorer than the top-performing compounds such as 7l, supporting its low biochemical activity.

These findings emphasize the importance of hydrogen bonds with A636, Q635, and H593 for effective urease inhibition. Compound 7l demonstrates this requirement, whereas 7b does not, which aligns with their respective inhibitory outcomes and is clearly supported by their docking score differences.

Taken together, the docking studies provide a clear structural rationale for the observed enzyme inhibition profiles of the synthesized compounds. The most active derivatives in each enzyme assay displayed well-oriented binding poses, characterized by strong hydrogen bonding with key catalytic residues and reinforced by hydrophobic stabilization within the binding pocket. In contrast, the least active compounds lacked these essential interactions and adopted displaced or weakly anchored orientations, which correlated strongly with their poor biochemical activities. The combined evidence from PyMOL-visualized docking poses and PLIP-generated interaction maps confirm that hydrogen bond formation with catalytic residues, complemented by favorable hydrophobic contacts, is the principal determinant of inhibitory potency against LOX, α-glucosidase, and urease. These findings validate the docking workflow and support the proposed structure–activity relationships of this compound series.

## Conclusions

A series of novel compounds containing 1,3,4-oxadiazole and piperidine moieties were designed and synthesized with excellent yields. Various spectral analyses were employed to corroborate the structures and screening of novel compounds against activity of target enzymes: lipoxygenase, urease, and α-glucosidase. Compounds exhibited moderate to high inhibitory potential against the target enzymes. Compounds 7a and 7n revealed higher inhibitory activity against α-glucosidase enzyme, compounds 7a, 7b, 7h, and 7n were found to be the most potent against lipoxygenase enzyme while compounds 7a and 7l revealed excellent inhibitory activity against urease enzyme and subsequently have ability to replace the respective standards after further studies. DFT and docking results suggested that molecules of the compounds studied can easily accommodate themselves to the enzyme active sites, due to high potential flexibility of these compounds, and can quite strongly interact with the enzyme active sites and with polar molecules of the human body environment, due to significant MEP accumulations in the molecules of the compounds studied. It was shown that almost all compounds studied have significant HOMO/LUMO gaps in the implicit DMSO, implying that all these compounds should have quite high thermodynamic stability. Furthermore, incorporation of detailed docking scores and PLIP-assisted interaction mapping confirmed that the most active inhibitors demonstrated well-oriented binding poses with strong hydrogen bonding and hydrophobic stabilization, providing a clear structural rationale for their observed biological activities. These integrated computational insights reinforce the SAR trends discussed and validate the reliability of the docking workflow used in this study. It will be necessary to make deeper analysis of the relation between the chemical structure/electronic properties of these compounds and their biological activity.

This research can be further extended to obtain better outcomes in drug discovery programs and can explore new horizons in pharmaceutical research.

## Experimental part

### Materials and methods

Jasco-320-A was used to record IR spectra. Bruker spectrometer was employed to record ^1^H-NMR (600 MHz frequency) and ^13^C-NMR spectra (150 MHz frequency) with the coupling constant in Hz and chemical shifts in ppm. CDCl_3_/DMSO mixture were used as a solvent. Griffin and George apparatus was used to record melting points (uncorrected) with the help of open capillary tube. Thin-layer chromatography (TLC) performed on aluminum plates pre-coated with silica gel using a solvent system having different ratio of ethyl acetate and n-hexane was used to monitor progress of the reaction and purity of synthesized compounds. UV lamp (λ = 254 nm) was used to visualize the spots during the TLC chromatography.

### Synthesis of ethyl-1-[(3-methoxyphenyl sulfonyl]piperidine-3-carboxylate (1)

4-Methoxy benzene sulfonyl chloride **(a;** 0.06 mol**)** and ethyl nipecotate **(b;** 0.06 mol**)** were reacted in equimolar quantity by stirring for 12 h using distilled water as a solvent. Na_2_CO_3_ solution (5%) was used to maintain pH at 8–10. TLC assured the completion of the reaction. EtOAc and n-hexane mixture (30:70) was utilized to develop chromatograms and visualization of spots was done using UV light. On completion of the reaction, diluted HCl was added dropwise to neutralize the mixture. Products were collected by filtration and washed with cold distilled water.

### Synthesis of 1-[(4-methoxyphenyl)sulfonyl]piperidien-3-carbohydrazide (2)

Compound **1** (0.05 mol) was refluxed with hydrazine hydrate in equimolar amount using methanol as a solvent for 2 h to convert it into 1-[(4-methoxyphenyl)sulfonyl]piperidien-3-carbohydrazide (**2;** 0.05 mol). TLC was run to confirm the reaction completion. After the reaction completion, the excess of a solvent was evaporated, and the product was cooled at room temperature. Filtration was used to quench the product. Methanol was used to wash the product which was further air dried.

### Synthesis of 5-[1-(4-methoxyphenyl)sulfonyl)piperidine-3-yl]-1,3,4-oxadiazole-2-thiol (3)

5-[1-(4-Methoxyphenyl)sulfonyl)piperidine-3-yl]-1,3,4-oxadiazole-2-thiol **(3;** 0.08 mol**)** was prepared by refluxing compound **2 (**0.08 mol) with equimolar amount of CS_2_ and KOH in the methanol solvent for 2 h. TLC was used for assurance of the reaction completion. On completion of the reaction, the reaction contents were cooled down at room temperature and chilled distilled water was added in excess. Reaction contents were acidified by the addition of diluted HCl dropwise to obtain the maximum product yield. The product formed was filtered and cold distilled water was utilized to wash it.

### General method to synthesize *N*-alkyl/aralkyl/aryl acetamides (6a-6o)

0.02 mol of 2-bromoacetyl bromide **(4)** was stirred with 0.02 mol of *N*-alkyl/aralkyl/aryl amines **(**0.03 mol; **5a-5o)** using 5% aqueous solution of Na_2_CO_3_ to maintain pH at 8–10. The product obtained in the form of precipitates was filtered while solvent extraction was used to obtain liquid products. TLC confirmed that reactants have been consumed completely.

### General method to synthesize *N*-alkyl/aralkyl/aryl acetamide analogues of 1,3,4-oxadiazole (7a-7o)

0.5 mmol of *N*-alkyl/aralkyl/aryl amines **6a-6o** and compound **3 (**0.5 mmol) reacted in DMF solvent in the presence of LiH by stirring for 6 h at room temperature. Target compounds **7a-7o** were acquired by simple filtration, washed by using cold distilled water and air dried.

### *N*-(2,4-Dimethylphenyl)-2-((5-(1-((4-methoxyphenyl)sulfonyl)piperidin-3-yl)-1,3,4-oxadiazol-2-yl)thio)acetamide (7a)

Light orange solid; yield: 82%; m.p.: 185–187 °C; HR-MS: [M]^•+^ 516.1501 (Calcd. for C_24_H_28_N_4_O_5_S_2_, 516.6310). *Anal*. Calcd for C_24_H_28_N_4_O_5_S_2_: C, 55.80; H, 5.46; N, 10.84; O, 15.48; S, 12.41. Found: C, 55.72; H, 5.41; N, 10.80; O, 15.41; S, 12.35. IR (KBr, *v*_max_ cm^− 1^): 1348 (-SO_2_ str.), 1515 (Ar C = C str.), 1572 (C = N str.), 3044 (Ar-H), 1234, 1076 (C-O-C str.), 3346 (N-H str.), 1666 (C = O str.). ^1^H-NMR (600 MHz, CDCl_3_, *δ* / ppm): 7.72 (d, *J* = 8.9 Hz, 2H, H-2’’ & H-6’’), 7.67 (d, *J* = 8.2 Hz, 1H, H-6’’’), 7.07 (d, *J* = 8.3 Hz, 1H, H-5’’’), 7.04 (s, 1H, H-3’’’), 7.01 (d, *J* = 8.9 Hz, 2H, H-3’’ & H-5’’), 4.05 (s, 2H, H -1’’’’), 4.01–3.99 (m, 1H, H*e*-2’), 3.89 (s, 3H, H-7’’), 3.71–3.69 (m, 1H, Ha-2’), 3.14–3.09 (m, 1H, H-3’), 2.63–2.59 (m, 1H, H*e*-6’), 2.44–2.40 (m, 1H, H*a*-6’), 2.32 (s, 3H, H-8’’’), 2.31 (s, 3H, H-7’’’), 2.16–2.13 (m, 1H, H*a*-5’), 1.92–1.89 (m, 2H, H*a*-4’), 1.80–1.72 (m, H, H*e-*5’), 1.64–1.57 (m, 1H, H*e*-4). ^13^C-NMR (150 MHz, CDCl_3_, *δ* / ppm): 163.11 (C-1’’’’), 161.56 (C-5), 160.83 (C-2), 160.43 (C-4’’), 133.65 (C-4’’’), 133.29 (C-2’’’), 131.38 (C-1’’), 129.74 (C-2’’ & C-6’’), 127.69 (C-1’’’), 126.97 (C-3’’’), 126.22 (C-5’’’), 119.66 (C-6’’’), 114.34 (C-3’’ & C-5’’), 55.61 (C-7’’), 52.83 (C-2’), 48.61 (C-6’), 46.27 (C-2’’’’), 33.60 (C-3’), 27.28 (C-4’), 23.87 (C-5’), 20.71 (C-8’’’), 17.63 (C-7’’’); EIMS (*m/z*): 509 [M]^+^, 389 [C_17_H_13_N_2_O_5_S_2_]^+^, 348 [C_15_H_12_N_2_O_4_S_2_]^+^, 163 [C_8_H_5_NO_3_]^+^, 289 [C_14_H_11_NO_4_S]^+^, 275 [C_14_H_11_O_4_S]^+^, 274 [C_14_H_12_NO_3_S]^+^, 248 [C_13_H_12_O_3_S]^+^, 162 [C_10_H_12_NO]^+^, 120 [C_8_H_10_N]^+^, 65 [C_5_H_5_]^+^, 52 [C_4_H_4_]^+^.

### *N*-(2,3-Dimethylphenyl)-2-((5-(1-((4-methoxyphenyl)sulfonyl)piperidin-3-yl)-1,3,4-oxadiazol-2-yl)thio)acetamide (7b)

Light pink solid; yield: 87%; m.p.: 174–176 °C; HR-MS: [M]^•+^ 516.1501 (Calcd. for C_24_H_28_N_4_O_5_S_2_, 516.6310). *Anal*. Calcd for C_24_H_28_N_4_O_5_S_2_: C, 55.80; H, 5.46; N, 10.84; O, 15.48; S, 12.41. Found: C, 55.73; H, 5.42; N, 10.82; O, 15.42; S, 12.34. IR (KBr, *v*_max_ cm^− 1^): 1350 (-SO_2_ str.), 1513 (Ar C = C str.), 1573 (C = N str.), 1238, 1078 (C-O-C str.), 3342 (N-H str.), 1664 (C = O str.), 3046 (Ar-H str.). ^1^H-NMR (600 MHz, CDCl_3_, *δ* / ppm): 7.07 (s, 1H, NH), 7.72 (d, *J* = 8.9 Hz, 2H, H-2’’ & H-6’’), 7.60 (d, *J* = 8.0 Hz, 1H, H-6’’’), 7.16 (t, *J* = 7.9 Hz, 1H, H-5’’’), 7.01 (d, *J* = 8.9 Hz, 2H, H-3’’ & H-5’’), 7.00 (d, *J* = 7.6 Hz, 1H, H-4’’’), 4.05 (s, 2H, H -1’’’’), 4.02–3.99 (m, 1H, H*e*-2’), 3.89 (s, 3H, H-7’’), 3.72–3.70 (m, 1H, H*a*-2’), 3.15–3.10 (m, 1H, H-3’), 2.63–2.59 (m, 1H, H*e*-6’), 2.44–2.40 (m, 1H, H*a*-6’), 2.35 (s, 3H, H-8’’’), 2.25 (s, 3H, H-7’’’), 2.16–2.13 (m, 1H, H*a*-5’), 1.92–1.89 (m, 2H, H*a*-4’), 1.78–1.73 (m, 1H, H*e-*5’), 1.64–1.59 (m, 1H, H*e*-4’). ^13^C-NMR (150 MHz, CDCl_3_, *δ* / ppm): 163.11 (C-1’’’’), 161.00 (C-5), 160.83 (C-2), 160.54 (C-4’’), 137.61 (C-1’’’), 135.74 (C-3’’’), 131.38 (C-1’’), 129.74 (C-2’’ & C-6’’), 127.70 (C-2’’’), 126.34 (C-4’’’), 126.25 (C-5’’’), 118.05 (C-6’’’), 114.34 (C-3’’ & C-5’’), 55.61 (C-7’’), 52.83 (C-2’), 48.61 (C-6’), 46.27 (C-2’’’’), 33.62 (C-3’), 27.30 (C-4’), 23.88 (C-5’), 20.65 (C-8’’’), 13.42 (C-7’’’); EIMS (*m/z*): 509 [M]^+^, 389 [C_17_H_13_N_2_O_5_S_2_]^+^, 348 [C_15_H_12_N_2_O_4_S_2_]^+^, 163 [C_8_H_5_NO_3_]^+^, 289 [C_14_H_11_NO_4_S]^+^, 275 [C_14_H_11_O_4_S]^+^, 274 [C_14_H_12_NO_3_S]^+^, 248 [C_13_H_12_O_3_S]^+^, 162 [C_10_H_12_NO]^+^, 120 [C_8_H_10_N]^+^, 65 [C_5_H_5_]^+^, 52 [C_4_H_4_]^+^.

### *N*-(2,5-Dimethylphenyl)-2-((5-(1-((4-methoxyphenyl)sulfonyl)piperidin-3-yl)-1,3,4-oxadiazol-2-yl)thio)acetamide (7c)

Off white solid; yield: 86%; m.p.: 171–173 °C; HR-MS: [M]^•+^ 516.1501 (Calcd. for C_24_H_28_N_4_O_5_S_2_, 516.6310). *Anal*. Calcd for C_24_H_28_N_4_O_5_S_2_: C, 55.80; H, 5.46; N, 10.84; O, 15.48; S, 12.41. Found: C, 55.70; H, 5.39; N, 10.81; O, 15.40; S, 12.35. IR (KBr, *v*_max_ cm^− 1^): 1346 (-SO_2_ str.), 1516 (Ar C = C str.), 1569 (C = N str.), 1232, 1074 (C-O-C str.), 3349 (N-H str.), 1670 (C = O str.), 3047 (Ar-H str.). ^1^H-NMR (600 MHz, CDCl_3_, *δ* / ppm): 7.75 (s, 1H, H-6’’’), 7.73 (d, *J* = 8.9 Hz, 2H, H-2’’ & H-6’’), 7.09 (d, *J* = 7.6 Hz, 1H, H-3’’’), 7.02 (d, *J* = 8.9 Hz, 2H, H-3’’ & H-5’’), 6.87 (d, *J* = 7.5 Hz, 1H, H-4’’’), 4.05 (s, 2H, H -1’’’’), 3.89 (s, 3H, H-7’’), 4.03-4.00 (m, 1H, H*e*-2’), 3.72–3.70 (m, 1H, H*a*-2’), 3.16–3.13 (m, 1H, H-3’), 2.65–2.61 (m, 1H, H*e*-6’), 2.46–2.42 (m, 1H, H*a*-6’), 2.37 (s, 3H, H-8’’’), 2.30 (s, 3H, H-7’’’), 2.18–2.15 (m, 1H, H*a*-5’), 1.94–1.91 (m, 2H, H*a*-4’), 1.80–1.77 (m, 1H, H*e-*5’), 1.66–1.60 (m, 1H, H*e*-4’). ^13^C-NMR (150 MHz, CDCl_3_, *δ* / ppm): 163.12 (C-1’’’’), 161.00 (C-5), 160.52 (C-2), 160.37 (C-4’’), 137.24 (C-1’’’), 135.54 (C-5’’’), 131.38 (C-1’’), 129.74 (C-2’’ & C-6’’), 127.70 (C-3’’’), 124.44 (C-2’’’), 122.86 (C-4’’’), 119.41 (C-6’’’), 114.34 (C-3’’ & C-5’’), 55.61 (C-7’’), 52.83 (C-2’), 48.62 (C-6’), 46.28 (C-2’’’’), 33.58 (C-3’), 27.25 (C-4’), 23.86 (C-5’), 21.29 (C-8’’’), 17.14 (C-7’’’); EIMS (*m/z*): 509 [M]^+^, 389 [C_17_H_13_N_2_O_5_S_2_]^+^, 348 [C_15_H_12_N_2_O_4_S_2_]^+^, 163 [C_8_H_5_NO_3_]^+^, 289 [C_14_H_11_NO_4_S]^+^, 275 [C_14_H_11_O_4_S]^+^, 274 [C_14_H_12_NO_3_S]^+^, 248 [C_13_H_12_O_3_S]^+^, 162 [C_10_H_12_NO]^+^, 120 [C_8_H_10_N]^+^, 65 [C_5_H_5_]^+^, 52 [C_4_H_4_]^+^.

### *N*-(2,6-Dimethylphenyl)-2-((5-(1-((4-methoxyphenyl)sulfonyl)piperidin-3-yl)-1,3,4-oxadiazol-2-yl)thio)acetamide (7d)

Pink white solid; yield: 83%; m.p.: 196–198 °C; HR-MS: [M]^•+^ 516.1501 (Calcd. for C_24_H_28_N_4_O_5_S_2_, 516.6310). *Anal*. Calcd for C_24_H_28_N_4_O_5_S_2_: C, 55.80; H, 5.46; N, 10.84; O, 15.48; S, 12.41. Found: C, 55.74; H, 5.39; N, 10.80; O, 15.41; S, 12.32. IR (KBr, *v*_max_ cm^− 1^): 1342 (-SO_2_ str.), 1520 (Ar C = C str.), 1565 (C = N str.), 1239, 1077 (C-O-C str.), 3348 (N-H str.), 1665 (C = O str.), 3050 (Ar-H str.). ^1^H-NMR (600 MHz, CDCl_3_, *δ* / ppm): 8.01 (s, 1H, NH), 7.69 (d, *J* = 8.9 Hz, 2H, H-2’’ & H-6’’), 7.19 (d, *J* = 8.6 Hz, 2H, H-3’’’ & H-5’’’), 7.17 (t, *J* = 8.4 Hz, 1H, H-4’’’), 7.01 (d, *J* = 8.9 Hz, 2H, H-3’’ & H-5’’), 6.87 (d, *J* = 7.5 Hz, 1H, H-4’’’), 4.05 (s, 2H, H -1’’’’), 4.03-4.00 (m, 1H, H*e*-2’), 3.89 (s, 3H, H-7’’), 3.72–3.70 (m, 1H, H*a*-2’), 3.16–3.13 (m, 1H, H-3’), 2.65–2.61 (m, 1H, H*e*-6’), 2.46–2.42 (m, 1H, H*a*-6’), 2.24 (s, 3H, H-8’’’), 2.23 (s, 3H, H-7’’’), 2.18–2.15 (m, 1H, H*a*-5’), 1.94–1.91 (m, 2H, H*a*-4’), 1.80–1.77 (m, 1H, H*e-*5’), 1.66–1.60 (m, 1H, H*e*-4’). ^13^C-NMR (150 MHz, CDCl_3_, *δ* / ppm): 163.12 (C-1’’’’), 161.00 (C-5), 160.52 (C-2), 160.37 (C-4’’), 136.07 (C-1’’’), 135.95 (C-2’’’), 135.25 (C-6’’’), 132.29 (C-1’’), 129.74 (C-2’’ & C-6’’), 128.75 (C-3’’’), 128.33 (C-5’’’), 127.50 (C-4’’’), 114.36 (C-3’’ & C-5’’), 55.64 (C-7’’), 52.83 (C-2’), 48.06 (C-6’), 46.43 (C-2’’’’), 33.16 (C-3’), 26.86 (C-4’), 23.53 (C-5’), 17.69 (C-7’’’ & C-8’’’); EIMS (*m/z*): 509 [M]^+^, 389 [C_17_H_13_N_2_O_5_S_2_]^+^, 348 [C_15_H_12_N_2_O_4_S_2_]^+^, 163 [C_8_H_5_NO_3_]^+^, 289 [C_14_H_11_NO_4_S]^+^, 275 [C_14_H_11_O_4_S]^+^, 274 [C_14_H_12_NO_3_S]^+^, 248 [C_13_H_12_O_3_S]^+^, 162 [C_10_H_12_NO]^+^, 120 [C_8_H_10_N]^+^, 65 [C_5_H_5_]^+^, 52 [C_4_H_4_]^+^.

### *N*-(3,5-Dimethylphenyl)-2-((5-(1-((4-methoxyphenyl)sulfonyl)piperidin-3-yl)-1,3,4-oxadiazol-2-yl)thio)acetamide (7e)

Off white solid; yield: 85%; m.p.: 178–180 °C; HR-MS: [M]^•+^ 516.1501 (Calcd. for C_24_H_28_N_4_O_5_S_2_, 516.6310). *Anal*. Calcd for C_24_H_28_N_4_O_5_S_2_: C, 55.80; H, 5.46; N, 10.84; O, 15.48; S, 12.41. Found: C, 55.71; H, 5.37; N, 10.82; O, 15.40; S, 12.35. IR (KBr, *v*_max_ cm^− 1^): 1346 (-SO_2_ str.), 1512 (Ar C = C str.), 1561 (C = N str.), 1242, 1077 (C-O-C str.), 3347 (N-H str.), 1660 (C = O str.), 3058 (Ar-H str.). ^1^H-NMR (600 MHz, CDCl_3_, *δ* / ppm): 7.72(d, *J* = 8.9 Hz, 2H, H-2’’ & H-6’’), 7.10 (s, 2H, H-2’’’ & H-6’’’), 7.01 (d, *J* = 8.9 Hz, 2H, H-3’’ & H-5’’), 6.74 (s,1H, H-4’’’), 4.05 (s, 2H, H -1’’’’), 3.89 (s, 3H, H-7’’), 3.99–3.97 (m, 1H, H*e*-2’), 3.69–3.67 (m, 1H, H*a*-2’), 3.16–3.12 (m, 1H, H-3’), 2.67–2.63 (m, 1H, H*e*-6’), 2.48–2.41 (m, 1H, H*a*-6’), 2.34 (s, 3H, H-8’’’), 2.30 (s, 3H, H-7’’’), 2.16–2.13 (m, 1H, H*a*-5’), 1.94–1.90 (m, 2H, H*a*-4’), 1.78–1.76 (m, 1H, H*e-*5’), 1.67–1.62 (m, 1H, H*e*-4’). ^13^C-NMR (150 MHz, CDCl_3_, *δ* / ppm): 163.13 (C-1’’’’), 161.00 (C-5), 160.35 (C-2), 160.04 (C-4’’), 139.18 (C-3’’’), 137.38 (C-5’’’), 132.29 (C-1’’), 129.74 (C-2’’ & C-6’’), 127.64 (C-1’’’), 124.94 (C-4’’’), 115.35 (C-2’’’ & C-6’’’), 114.35 (C-3’’ & C-5’’), 55.61 (C-7’’), 52.83 (C-2’), 48.62 (C-6’), 46.29 (C-2’’’’), 33.50 (C-3’), 27.20 (C-4’), 23.81 (C-5’), 21.42 (C-7’’’ & C-8’’’); EIMS (*m/z*): 509 [M]^+^, 389 [C_17_H_13_N_2_O_5_S_2_]^+^, 348 [C_15_H_12_N_2_O_4_S_2_]^+^, 163 [C_8_H_5_NO_3_]^+^, 289 [C_14_H_11_NO_4_S]^+^, 275 [C_14_H_11_O_4_S]^+^, 274 [C_14_H_12_NO_3_S]^+^, 248 [C_13_H_12_O_3_S]^+^, 162 [C_10_H_12_NO]^+^, 120 [C_8_H_10_N]^+^, 65 [C_5_H_5_]^+^, 52 [C_4_H_4_]^+^.

### 2-((5-(1-((4-Methoxyphenyl)sulfonyl)piperidin-3-yl)-1,3,4-oxadiazol-2-yl)thio)-*N*-phenylacetamide (7f)

Orange white solid; yield: 87%; m.p.: 193–195 °C; HR-MS: [M]^•+^ 488.1188 (Calcd. for C_22_H_24_N_4_O_5_S_2_, 458.5770). *Anal*. Calcd for C_22_H_24_N_4_O_5_S_2_: C, 54.08; H, 4.95; N, 11.47; O, 16.37; S, 13.12. Found: C, 54.02; H, 4.91; N, 11.42; O, 16.32; S, 13.10. IR (KBr, *v*_max_ cm^− 1^): 1347 (-SO_2_ str.), 1513 (Ar C = C str.), 1562 (C = N str.), 1244, 1070 (C-O-C str.), 3339 (N-H str.), 1659 (C = O str.), 3054 (Ar-H str.). ^1^H-NMR (600 MHz, CDCl_3_, *δ* / ppm): 7.72 (d, *J* = 8.9 Hz, 2H, H-2’’ & H-6’’), 7.10 (s, 2H, H-2’’’ & H-6’’’), 7.01 (d, *J* = 8.9 Hz, 2H, H-3’’ & H-5’’), 6.74 (s,1H, H-4’’’), 4.05 (s, 2H, H -1’’’’), 3.89 (s, 3H, H-7’’), 3.99–3.97 (m, 1H, H*e*-2’), 3.69–3.67 (m, 1H, H*a*-2’), 3.16–3.12 (m, 1H, H-3’), 2.67–2.63 (m, 1H, H*e*-6’), 2.48–2.41 (m, 1H, H*a*-6’), 2.34 (s, 3H, H-8’’’), 2.30 (s, 3H, H-7’’’), 2.16–2.13 (m, 1H, H*a*-5’), 1.94–1.90 (m, 2H, H*a*-4’), 1.78–1.76 (m, 1H, H*e-*5’), 1.67–1.62 (m, 1H, H*e*-4’). ^13^C-NMR (150 MHz, CDCl_3_, *δ* / ppm): 163.14 (C-1’’’’), 161.00 (C-5), 160.44 (C-2), 160.07 (C-4’’), 137.61 (C-1’’’), 132.29 (C-1’’), 129.74 (C-2’’ & C-6’’), 129.38 (C-3’’’ & C-5’’’), 123.08 (C-4’’’), 117.54 (C-2’’’ & C-6’’’), 114.36 (C-3’’ & C-5’’), 55.62 (C-7’’), 52.83 (C-2’), 48.61 (C-6’), 46.29 (C-2’’’’), 33.53 (C-3’), 27.20 (C-4’), 23.79 (C-5’); EIMS (*m/z*): 481 [M]^+^, 389 [C_17_H_13_N_2_O_5_S_2_]^+^, 348 [C_15_H_12_N_2_O_4_S_2_]^+^, 289 [C_14_H_11_NO_4_S]^+^, 275 [C_14_H_11_O_4_S]^+^, 274 [C_14_H_12_NO_3_S]^+^, 248 [C_13_H_12_O_3_S]^+^, 134 [C_8_H_8_NO]^+^, 92 [C_6_H_6_N]^+^, 65 [C_5_H_5_]^+^, 52 [C_4_H_4_]^+^.

### 2-((5-(1-((4-Methoxyphenyl)sulfonyl)piperidin-3-yl)-1,3,4-oxadiazol-2-yl)thio)-*N*-(o-tolyl)acetamide (7 g)

Light peach solid; yield: 85%; m.p.: 114–116 °C; HR-MS: [M]^•+^ 502.1345 (Calcd. for C_23_H_26_N_4_O_5_S_2_, 502.6040). *Anal*. Calcd for C_23_H_26_N_4_O_5_S_2_: C, 54.96; H, 5.21; N, 11.15; O, 15.92; S, 12.76. Found: C, 54.92; H, 5.20; N, 11.12; O, 15.90; S, 12.72. IR (KBr, *v*_max_ cm^− 1^): 1343 (-SO_2_ str.), 1514 (Ar C = C str.), 1564 (C = N str.), 1242, 1077 (C-O-C str.), 3343 (N-H str.), 1658 (C = O str.), 3056 (Ar-H str.). ^1^H-NMR (600 MHz, CDCl_3_, *δ* / ppm): 7.89 (d, *J* = 8.0 Hz, 1H, H-6’’’), 7.73 (d, *J* = 11.7 Hz, 2H, H-2’’ & H-6’’), 7.22 (d, *J* = 7.3 Hz, 1H, H-3’’’), 7.06 (t, *J* = 7.4 Hz, 1H, H-5’’’ ), 7.02 (d, *J* = 11.9 Hz, 2H, H-3’’ & H-5’’), 7.00- 6.98 (m, 1H, H-4’’’), 4.07-4.00 (m, 1H, H*e*-2’), 4.05 (s, 2H, H -1’’’’), 3.89 (s, 3H, H-7’’), 3.71–3.69 (m, 1H, H*a*-2’), 3.16–3.13 (m, 1H, H-3’), 2.66–2.62 (m, 1H, H*e*-6’), 2.47–2.43 (m, 1H, H*a*-6’), 2.35 (s, 3H, H-7’’’), 2.18–2.15 (m, 1H, H*a*-5’), 1.93–1.91 (m,2H, H*a*-4’), 1.79–1.76 (m, 1H, H*e-*5’), 1.68–1.62 (m, 1H, H*e*-4’). ^13^C-NMR (150 MHz, CDCl_3_, *δ* / ppm): 163.13 (C-1’’’’), 161.00 (C-5), 160.57 (C-2), 160.40 (C-4’’), 135.81 (C-1’’’), 132.29 (C-1’’), 129.74 (C-2’’ & C-6’’), 127.68 (C-2’’’), 127.35 (C-3’’’), 126.03 (C-4’’’), 123.71 (C-5’’’), 118.79 (C-6’’’), 114.34 (C-3’’ & C-5’’), 55.64 (C-7’’), 52.83 (C-2’), 48.61 (C-6’), 46.28 (C-2’’’’), 33.59 (C-3’), 27.25 (C-4’), 23.85 (C-5’), 17.60 (C-7’’’); EIMS (*m/z*): 495 [M]^+^, 389 [C_17_H_13_N_2_O_5_S_2_]^+^, 348 [C_15_H_12_N_2_O_4_S_2_]^+^, 289 [C_14_H_11_NO_4_S]^+^, 275 [C_14_H_11_O_4_S]^+^, 274 [C_14_H_12_NO_3_S]^+^, 248 [C_13_H_12_O_3_S]^+^, 148 [C_9_H_10_NO]^+^, 106 [C_7_H_8_N]^+^, 65 [C_5_H_5_]^+^, 52 [C_4_H_4_]^+^.

### 2-((5-(1-((4-Methoxyphenyl)sulfonyl)piperidin-3-yl)-1,3,4-oxadiazol-2-yl)thio)-*N*-(m-tolyl)acetamide (7 h)

Light grey solid; yield: 87%; m.p.: 170–172 °C; HR-MS: [M]^•+^ 502.1345 (Calcd. for C_23_H_26_N_4_O_5_S_2_, 502.6040). *Anal*. Calcd for C_23_H_26_N_4_O_5_S_2_: C, 54.96; H, 5.21; N, 11.15; O, 15.92; S, 12.76. Found: C, 54.91; H, 5.21; N, 11.10; O, 15.89; S, 12.71. IR (KBr, *v*_max_ cm^− 1^): 1354 (-SO_2_ str.), 1522 (Ar C = C str.), 1563 (C = N str.), 1246, 1077 (C-O-C str.), 3346 (N-H str.), 1663 (C = O str.), 3057 (Ar-H str.). ^1^H-NMR (600 MHz, CDCl_3_, *δ* / ppm): 7.72 (d, *J* = 8.9 Hz, 2H, H-2’’ & H-6’’), 7.44 (s, 1H, NH), 7.33 (s, 1H, H-2’’’), 7.27–7.24 (m, 2H, H-5’’’ & H-6’’’), 7.01 (d, *J* = 8.9 Hz, 2H, H-3’’ & H-5’’), 6.91 (d, *J* = 5.6 Hz, 1H, H-4’’’), 4.00-3.98 (m, 1H, H*e*-2’), 4.05 (s, 2H, H -1’’’’), 3.89 (s, 3H, H-7’’), 3.70–3.68 (m, 1H, H*a*-2’), 3.17–3.12 (m, 1H, H-3’), 2.67–2.63 (m, 1H, H*e*-6’), 2.48–2.44 (m, 1H, H*a*-6’), 2.38 (s, 3H, H-7’’’), 2.17–2.14 (m, 1H, H*a*-5’), 1.94–1.90 (m, 2H, H*a*-4’), 1.80–1.74 (m, 1H, H*e-*5’), 1.68–1.62 (m, 1H, H*e*-4’). ^13^C-NMR (150 MHz, CDCl_3_, *δ* / ppm): 163.14 (C-1’’’’), 161.00 (C-5), 160.39 (C-2), 160.06 (C-4’’), 139.41 (C-3’’’), 137.51 (C-1’’’), 132.29 (C-1’’), 129.74 (C-2’’ & C-6’’), 129.19 (C-5’’’), 127.63 (C-4’’’), 123.95 (C-2’’’), 118.19 (C-6’’’), 114.66 (C-3’’ & C-5’’), 55.62 (C-7’’), 52.83 (C-2’), 48.62 (C-6’), 46.29 (C-2’’’’), 33.53 (C-3’), 27.20 (C-4’), 23.81 (C-5’), 21.54 (C-7’’’); EIMS (*m/z*): 495 [M]^+^, 389 [C_17_H_13_N_2_O_5_S_2_]^+^, 348 [C_15_H_12_N_2_O_4_S_2_]^+^, 289 [C_14_H_11_NO_4_S]^+^, 275 [C_14_H_11_O_4_S]^+^, 274 [C_14_H_12_NO_3_S]^+^, 248 [C_13_H_12_O_3_S]^+^, 148 [C_9_H_10_NO]^+^, 106 [C_7_H_8_N]^+^, 65 [C_5_H_5_]^+^, 52 [C_4_H_4_]^+^.

### *N*-(2-ethylphenyl)-2-((5-(1-((4-methoxyphenyl)sulfonyl)piperidin-3-yl)-1,3,4-oxadiazol-2-yl)thio)acetamide (7i)

Peach solid; yield: 82%; m.p.: 116–118 °C; HR-MS: [M]^•+^ 516.1501 (Calcd. for C_24_H_28_N_4_O_5_S_2_, 516.6310). *Anal*. Calcd for C_24_H_28_N_4_O_5_S_2_: C, 55.80; H, 5.46; N, 10.84; O, 15.48; S, 12.41. Found: C, 55.76; H, 5.40; N, 10.81; O, 15.42; S, 12.40. IR (KBr, *v*_max_ cm^− 1^): 1358 (-SO_2_ str.), 1528 (Ar C = C str.), 1568 (C = N str.), 1228, 1071 (C-O-C str.), 3344 (N-H str.), 1668 (C = O str.), 3058 (Ar-H str.). ^1^H-NMR (600 MHz, CDCl_3_, *δ* / ppm): 7.87 (d, *J* = 8.0 Hz, 1H, H-6’’’), 7.72 (d, *J* = 8.9 Hz, 2H, H-2’’ & H-6’’), 7.30–7.24 (m, 2H, H-3’’’ & H-5’’’), 7.11 (t, *J* = 7.4 Hz, 1H, H-4’’’), 7.01 (d, *J* = 8.9 Hz, 2H, H-3’’ & H-5’’), 6.92 (s, 1H, NH), 4.05 (s, 2H, H -1’’’’), 4.02-4.00 (m, 1H, H*e*-2’), 3.89 (s, 3H, H-7’’), 3.72–3.70 (m, 1H, H*a*-2’), 3.16–3.11 (m, 1H, H-3’), 2.68 (q, 2H, H-7’’’), 2.65–2.61 (m, 1H, H*e*-6’), 2.45–2.41 (m, 1H, H*a*-6’), 2.17–2.14 (m, 1H, H*a*-5’), 1.93–1.90 (m,2H, H*a*-4’), 1.80–1.73 (m, 1H, H*e-*5’), 1.65–1.60 (m, 1H, H*e*-4’), 1.30 (t, *J* = 7.6 Hz, 3H, H-8’’’). ^13^C-NMR (150 MHz, CDCl_3_, *δ* / ppm): 163.12 (C-1’’’’), 161.00 (C-5), 160.64 (C-2), 160.57 (C-4’’), 135.17 (C-1’’’), 132.29 (C-1’’), 129.74 (C-2’’ & C-6’’), 128.69 (C-2’’’), 127.68 (C-5’’’), 127.19 (C-4’’’), 124.12 (C-3’’’), 119.48 (C-6’’’), 114.37 (C-3’’ & C-5’’), 55.62 (C-7’’), 52.63 (C-2’), 48.61 (C-6’), 46.28 (C-2’’’’), 33.61 (C-3’), 27.26 (C-4’), 24.07 (C-7’’’), 23.86 (C-5’), 13.76 (C-8’’’); EIMS (*m/z*): 509 [M]^+^, 389 [C_17_H_13_N_2_O_5_S_2_]^+^, 348 [C_15_H_12_N_2_O_4_S_2_]^+^, 163 [C_8_H_5_NO_3_]^+^, 289 [C_14_H_11_NO_4_S]^+^, 275 [C_14_H_11_O_4_S]^+^, 274 [C_14_H_12_NO_3_S]^+^, 248 [C_13_H_12_O_3_S]^+^, 162 [C_10_H_12_NO]^+^, 120 [C_8_H_10_N]^+^, 65 [C_5_H_5_]^+^, 52 [C_4_H_4_]^+^.

### *N*-(4-ethoxyphenyl)-2-((5-(1-((4-methoxyphenyl)sulfonyl)piperidin-3-yl)-1,3,4-oxadiazol-2-yl)thio)acetamide (7j)

Purple grey solid; yield: 83%; m.p.: 150–152 °C; HR-MS: [M]^•+^ 532.1450 (Calcd. for C_24_H_28_N_4_O_6_S_2_, 532.6300). *Anal*. Calcd for C_24_H_28_N_4_O_6_S_2_: C, 54.12; H, 5.30; N, 10.52; O, 18.02; S, 12.04. Found: C, 54.10; H, 5.25; N, 10.45; O, 18.00; S, 12.02. IR (KBr, *v*_max_ cm^− 1^): 1351 (-SO_2_ str.), 1514 (Ar C = C str.), 1566 (C = N str.), 1239, 1077 (C-O-C str.), 3345 (N-H str.), 1667 (C = O str.), 3060 (Ar-H str.). ^1^H-NMR (600 MHz, CDCl_3_, *δ* / ppm): 7.71 (d, *J* = 8.9 Hz, 2H, H-2’’ & H-6’’), 7.57 (s, 1H, NH), 7.37 (d, *J* = 8.8 Hz, 2H, H-3’’’ & H-5’’’), 7.01 (d, *J* = 8.9 Hz, 2H, H-3’’ & H-5’’), 6.90 (d, 2H, H-2’’’ & H-6’’’), 4.05 (s, 2H, H -1’’’’), 4.03(q, 2H, H-7’’’), 3.98–3.96 (m, 1H, H*e*-2’), 3.89 (s, 3H, H-7’’), 3.68–3.66 (m, 1H, H*a*-2’), 3.13–3.10 (m, 1H, H-3’), 2.65–2.62 (m, 1H, H*e*-6’), 2.47–2.43 (m, 1H, H*a*-6’), 2.15–2.12 (m, 1H, H*a*-5’), 1.92–1.89 (m, 2H, H*a*-4’), 1.79–1.75 (m, 1H, H*e-*5’), 1.74–1.61 (m, 1H, H*e*-4’), 1.42 (t, *J* = 13.9 Hz, 3H, H-8’’’). ^13^C-NMR (150 MHz, CDCl_3_, *δ* / ppm): 163.13 (C-1’’’’), 161.00 (C-5), 160.61 (C-2), 160.22 (C-4’’), 155.17 (C-4’’’), 132.29 (C-1’’), 129.73 (C-2’’ & C-6’’), 127.62 (C-1’’’), 119.68 (C-2’’’ & C-6’’’), 115.26 (C-3’’’ & C-5’’’), 114.35 (C-3’’ & C-5’’), 63.81 (C-7’’’), 55.62 (C-7’’), 52.63 (C-2’), 48.63 (C-6’), 46.29 (C-2’’’’), 33.55 (C-3’), 27.21 (C-4’), 23.81 (C-5’), 14.85 (C-8’’’); EIMS (*m/z*): 525 [M]^+^, 389 [C_17_H_13_N_2_O_5_S_2_]^+^, 348 [C_15_H_12_N_2_O_4_S_2_]^+^, 289 [C_14_H_11_NO_4_S]^+^, 275 [C_14_H_11_O_4_S]^+^, 274 [C_14_H_12_NO_3_S]^+^, 248 [C_13_H_12_O_3_S]^+^, 178 [C_10_H_12_NO_2_]^+^, 136 [C_8_H_10_NO]^+^, 65 [C_5_H_5_]^+^, 52 [C_4_H_4_]^+^.

### *N*-(2-ethyl-6-methylphenyl)-2-((5-(1-((4-methoxyphenyl)sulfonyl)piperidin-3-yl)-1,3,4-oxadiazol-2-yl)thio)acetamide (7k)

Peach solid; yield: 84%; m.p.: 145–147 °C; HR-MS: [M]^•+^ 530.1658 (Calcd. for C_25_H_30_N_4_O_5_S_2_, 530.6580). *Anal*. Calcd for C_25_H_30_N_4_O_5_S_2_: C, 56.59; H, 5.70; N, 10.56; O, 15.07; S, 12.08. Found: C, 56.54; H, 5.64; N, 10.52; O, 15.02; S, 12.04. IR (KBr, *v*_max_ cm^− 1^): 1346 (-SO_2_ str.), 1512 (Ar C = C str.), 1561 (C = N str.), 1226, 1069 (C-O-C str.), 3338 (N-H str.), 1658 (C = O str.), 3058 (Ar-H str.). ^1^H-NMR (600 MHz, CDCl_3_, *δ* / ppm): 7.71 (d, *J* = 8.9 Hz, 2H, H-2’’ & H-6’’), 7.22 (t, *J* = 7.6 Hz, 1H, H-4’’’), 7.15 (dd, *J* = 9.60 Hz, 2H, H-3’’’ & H-5’’’), 7.01 (d, *J* = 8.9 Hz, 2H, H-3’’ & H-5’’), 4.05 (s, 2H, H -1’’’’), 2.28 (s, 3H, H-7’’’), 4.00-3.97 (m, 1H, H*e*-2’), 3.90 (s, 3H, H-7’’), 3.72–3.70 (m, 1H, H*a*-2’), 3.05–3.01 (m, 1H, H-3’), 2.65 (q, 2H, H-8’’’), 2.56–2.53 (m, 1H, H*e*-6’), 2.37–2.33 (m, 1H, H*a*-6’), 2.13–2.08 (m, 1H, H*a*-5’), 1.87–1.84 (m,2H, H*a*-4’), 1.73–1.68 (m, 1H, H*e-*5’), 1.52–1.49 (m, 1H, H*e*-4’), 1.20 (t, *J* = 7.6 Hz, 3H, H-9’’’). ^13^C-NMR (150 MHz, CDCl_3_, *δ* / ppm): 163.09 (C-1’’’’), 161.00 (C-5), 160.70 (C-2), 160.22 (C-4’’), 141.47 (C-1’’’), 135.96 (C-2’’’), 132.29 (C-1’’), 129.73 (C-2’’ & C-6’’), 128.72 (C-6’’’), 127.92 (C-5’’’), 127.71 (C-4’’’), 126.84 (C-3’’’), 114.32 (C-3’’ & C-5’’), 55.62 (C-7’’), 52.63 (C-2’), 48.42 (C-6’), 46.24 (C-2’’’’), 33.67 (C-3’), 27.42 (C-4’), 24.70 (C-8’’’), 23.92 (C-5’), 18.33 (C-7’’’), 14.61 (C-9’’’); EIMS (*m/z*): 523 [M]^+^, 389 [C_17_H_13_N_2_O_5_S_2_]^+^, 348 [C_15_H_12_N_2_O_4_S_2_]^+^, 289 [C_14_H_11_NO_4_S]^+^, 275 [C_14_H_11_O_4_S]^+^, 274 [C_14_H_12_NO_3_S]^+^, 248 [C_13_H_12_O_3_S]^+^, 176 [C_11_H_14_NO]^+^, 134 [C_9_H_12_N]^+^, 65 [C_5_H_5_]^+^, 52 [C_4_H_4_]^+^.

### *N*-cyclohexyl-2-((5-(1-((4-methoxyphenyl)sulfonyl)piperidin-3-yl)-1,3,4-oxadiazol-2-yl)thio)acetamide (7l)

Off white solid; yield: 87%; m.p.: 154–156 °C; HR-MS: [M]^•+^ 494.1658 (Calcd. for C_22_H_30_N_4_O_5_S_2_, 494.6250). *Anal*. Calcd for C_22_H_30_N_4_O_5_S_2_: C, 53.42; H, 6.11; N, 11.33; O, 16.17; S, 12.96. Found: C, 53.40; H, 6.05; N, 11.30; O, 16.12; S, 12.91. IR (KBr, *v*_max_ cm^− 1^): 1347 (-SO_2_ str.), 1513 (Ar C = C str.), 1562 (C = N str.), 1227, 1079 (C-O-C str.), 3339 (N-H str.), 1657 (C = O str.), 3059 (Ar-H str.). ^1^H-NMR (600 MHz, CDCl_3_, *δ* / ppm): 7.71 (d, *J* = 8.9 Hz, 2H, H-2’’ & H-6’’), 7.01 (d, *J* = 8.9 Hz, 2H, H-3’’ & H-5’’), 4.05 (s, 2H, H -1’’’’), 4.00-3.97 (m, 1H, H*e*-2’), 3.89 (s, 3H, H-7’’), 3.72–3.70 (m, 1H, H*a*-2’), 3.05–3.01 (m, 1H, H-3’), 2.56–2.53 (m, 1H, H*e*-6’), 2.37–2.33 (m, 1H, H*a*-6’), 2.13–2.08 (m, 1H, H*a*-5’), 1.87–1.84 (m,2H, H*a*-4’), 1.75–1.74 (m, 2H, H*e*-2’’’ & H*e*-6’’’), 1.73–1.68 (m, 1H, H*e-*5’), 1.52–1.49 (m, 1H, H*e*-4’), 1.49–1.48 (m, 2H, H*a*-2’’’ & H*a*-6’’’), 1.48–1.47 (m, 1H, H*e*-4’’’), 1.42–1.36 (m, 1H, H*a*-4’’’), 1.28–1.21 (m, 2H, H*e*-3’’’ & H*e*-5’’’ ), 1.11–1.10 (m, 2H, H*e*-3’’’ & H*e*-5’’’). ^13^C-NMR (150 MHz, CDCl_3_, *δ* / ppm): 163.09 (C-1’’’’), 161.00 (C-5), 160.70 (C-2), 160.08 (C-4’’), 132.29 (C-1’’), 129.73 (C-2’’ & C-6’’), 114.32 (C-3’’ & C-5’’), 55.66 (C-7’’), 52.53 (C-2’), 48.70 (C-6’), 48.09 (C-1’’’), 46.28 (C-2’’’ & C-6’’’), 46.24 (C-2’’’’), 33.64 (C-3’), 27.42 (C-4’), 25.92 (C-4’’’), 24.97 (C-3’’’ & C-5’’’), 23.31 (C-5’); EIMS (*m/z*): 487 [M]^+^, 389 [C_17_H_13_N_2_O_5_S_2_]^+^, 348 [C_15_H_12_N_2_O_4_S_2_]^+^, 289 [C_14_H_11_NO_4_S]^+^, 275 [C_14_H_11_O_4_S]^+^, 274 [C_14_H_12_NO_3_S]^+^, 248 [C_13_H_12_O_3_S]^+^, 140 [C_8_H_14_NO]^+^, 98 [C_6_H_12_N]^+^, 70 [C_5_H_10_]^+^, 52 [C_4_H_4_]^+^.

### *N*-(2-methoxyphenyl)-2-((5-(1-((4-methoxyphenyl)sulfonyl)piperidin-3-yl)-1,3,4-oxadiazol-2-yl)thio)acetamide (7 m)

Brown solid; yield: 82%; m.p.: 159–161 °C; HR-MS: [M]^•+^ 518.1294 (Calcd. for C_23_H_26_N_4_O_6_S_2_, 518.6030). *Anal*. Calcd for C_23_H_26_N_4_O_6_S_2_: C, 53.27; H, 5.05; N, 10.80; O, 18.51; S, 12.36. Found: C, 53.22; H, 5.01; N, 10.80; O, 18.46; S, 12.32. IR (KBr, *v*_max_ cm^− 1^): 1342 (-SO_2_ str.), 1508 (Ar C = C str.), 1557 (C = N str.), 1237, 1076 (C-O-C str.), 3347 (N-H str.), 1668 (C = O str.), 3054 (Ar-H str.). ^1^H-NMR (600 MHz, CDCl_3_, *δ* / ppm): 8.12 (d, *J* = 9.5 Hz, 1H, H-6’’’), 7.73 (d, *J* = 8.9 Hz, 2H, H-2’’ & H-6’’), 6.93–6.91 (m, 1H, H-5’’’), 7.05-7.00 (m, 2H, H-3’’’ & H-4’’’), 7.01 (d, *J* = 11.7Hz, 2H, H-3’’ & H-5’’), 4.05 (s, 2H, H -1’’’’), 4.04-4.00 (m, 1H, H*e*-2’), 3.95 (s, 3H, H-7’’’), 3.88 (s, 3H, H-7’’), 3.73–3.71 (m, 1H, H*a*-2’), 3.18–3.13 (m, 1H, H-3’), 2.64–2.60 (m, 1H, H*e*-6’), 2.46–2.41 (m, 1H, H*a*-6’), 2.19–2.16 (m, 1H, H*a*-5’), 1.95–1.90 (m, 2H, H*a*-4’), 1.82–1.74 (m, 1H, H*e-*5’), 1.67–1.59 (m, 1H, H*e*-4’). ^13^C-NMR (150 MHz, CDCl_3_, *δ* / ppm): 163.11 (C-1’’’’), 161.00 (C-5), 160.70 (C-2), 160.22 (C-4’’), 146.96 (C-2’’’), 132.29 (C-1’’), 129.75 (C-2’’ & C-6’’), 127.68 (C-4’’’), 127.15 (C-1’’’), 121.36 (C-5’’’), 116.98 (C-6’’’), 114.37 (C-3’’ & C-5’’), 109.98 (C-3’’’), 55.79 (C-7’’), 52.63 (C-2’), 48.63 (C-6’), 46.28 (C-2’’’’), 33.55 (C-3’), 27.25 (C-4’), 23.88 (C-5’); EIMS (*m/z*): 511 [M]^+^, 389 [C_17_H_13_N_2_O_5_S_2_]^+^, 348 [C_15_H_12_N_2_O_4_S_2_]^+^, 289 [C_14_H_11_NO_4_S]^+^, 275 [C_14_H_11_O_4_S]^+^, 274 [C_14_H_12_NO_3_S]^+^, 248 [C_13_H_12_O_3_S]^+^, 164 [C_9_H_10_NO_2_]^+^, 122 [C_7_H_8_NO]^+^, 65 [C_5_H_5_]^+^, 52 [C_4_H_4_]^+^.

### *N*-benzyl-2-((5-(1-((4-methoxyphenyl)sulfonyl)piperidin-3-yl)-1,3,4-oxadiazol-2-yl)thio)acetamide (7n)

Orange sticky; yield: 76%; m.p.: 174–176 °C; HR-MS: [M]^•+^ 502.1345 (Calcd. for C_23_H_26_N_4_O_5_S_2_, 502.6040). *Anal*. Calcd for C_23_H_26_N_4_O_5_S_2_: C, 54.96; H, 5.21; N, 11.15; O, 15.92; S, 12.76. Found: C, 54.91; H, 5.16; N, 11.10; O, 15.90; S, 12.70. IR (KBr, *v*_max_ cm^− 1^): 1345 (-SO_2_ str.), 1511 (Ar C = C str.), 1560 (C = N str.), 1240, 1077 (C-O-C str.), 3352 (N-H str.), 1672 (C = O str.), 3057 (Ar-H str.). ^1^H-NMR (600 MHz, CDCl_3_, *δ* / ppm): 7.62 (d, *J* = 9.2 Hz, 2H, H-2’’ & H-6’’), 6.93 (d, *J* = 8.6 Hz, 2H, H-3’’ & H-5’’), 4.05 (s, 2H, H -1’’’’), 3.92–3.91 (m, 1H, H*e*-2’), 3.91 (s, 3H, H-7’’), 3.81–3.77 (m, 1H, H*a*-2’), 3.61–3.54 (m, 1H, H-3’), 3.01–2.99 (m, 1H, H*e*-6’), 2.82 (s, 3H, H-7’’’), 2.63–2.56 (m, 1H, H*a*-6’), 2.40–2.36 (m, 1H, H*a*-5’), 1.79–1.76 (m, 2H, H*a*-4’), 1.66–1.59 (m, 1H, H*e-*5’), 1.24–1.18 (m, 1H, H*e*-4’). ^13^C-NMR (150 MHz, CDCl_3_, *δ* / ppm): 163.18 (C-1’’’’), 161.00 (C-5), 160.70 (C-2), 160.22 (C-4’’), 152.93 (C-1’’’), 132.29 (C-1’’), 129.74 (C-2’’ & C-6’’), 128.98 (C-2’’’ & C-6’’’), 128.50 (C-3’’’ & C-5’’’), 126.76 (C-4’’’), 116.98 (C-6’’’), 114.37 (C-3’’ & C-5’’), 55.64 (C-7’’), 52.64 (C-2’), 48.41 (C-6’), 46.25 (C-7’’’), 46.19 (C-2’’’’), 33.55 (C-3’), 27.48 (C-4’), 23.64 (C-5’); EIMS (*m/z*): 509 [M]^+^, 389 [C_17_H_13_N_2_O_5_S_2_]^+^, 348 [C_15_H_12_N_2_O_4_S_2_]^+^, 163 [C_8_H_5_NO_3_]^+^, 289 [C_14_H_11_NO_4_S]^+^, 275 [C_14_H_11_O_4_S]^+^, 274 [C_14_H_12_NO_3_S]^+^, 248 [C_13_H_12_O_3_S]^+^, 162 [C_10_H_12_NO]^+^, 120 [C_8_H_10_N]^+^, 65 [C_5_H_5_]^+^, 52 [C_4_H_4_]^+^.

### Methyl-2-(2-((5-(1-((4-methoxyphenyl)sulfonyl)piperidin-3-yl)-1,3,4-oxadiazol-2-yl)thio)acetamido)-5-methylbenzoate (7o)

Brown sticky; yield: 73%; m.p.: 170–172 °C; HR-MS: [M]^•+^ 560.1399 (Calcd. for C_25_H_28_N_4_O_7_S_2_, 560.6400). *Anal*. Calcd for C_25_H_28_N_4_O_7_S_2_: C, 53.56; H, 5.03; N, 9.99; O, 19.98; S, 11.44. Found: C, 53.53; H, 5.01; N, 9.94; O, 19.93; S, 11.41. IR (KBr, *v*_max_ cm^− 1^): 1341 (-SO_2_ str.), 1515 (Ar C = C str.), 1556 (C = N str.), 1243, 1070 (C-O-C str.), 3354 (N-H str.), 1675 (C = O str.), 3055 (Ar-H str.). ^1^H-NMR (600 MHz, CDCl_3_, *δ* / ppm): 7.94 (s, 1H, H-3’’’), 7.71 (d, *J* = 8.9 Hz, 2H, H-2’’ & H-6’’), 6.97–6.8 3 (m, 2H, H-5’’’ & H-6’’’), 7.01 (d, *J* = 8.9 Hz, 2H, H-3’’ & H-5’’), 4.05 (s, 2H, H -1’’’’), 2.28 (s, 1H, H-7’’’), 4.00-3.97 (m, 1H, H*e*-2’), 3.91 (s, 3H, H-7’’), 3.72–3.70 (m, 1H, H*a*-2’), 3.05–3.01 (m, 1H, H-3’), 2.88 (s, 3H, H-8’’’), 2.80 (s, 3H, H-7’’’), 2.56–2.53 (m, 1H, H*e*-6’), 2.37–2.33 (m, 1H, H*a*-6’), 2.13–2.08 (m, 1H, H*a*-5’), 1.87–1.84 (m,2H, H*a*-4’), 1.73–1.68 (m, 1H, H*e-*5’), 1.52–1.49 (m, 1H, H*e*-4’). ^13^C-NMR (150 MHz, CDCl_3_, *δ* / ppm): 167.12 (C-8’’’), 163.18 (C-1’’’’), 161.00 (C-5), 160.70 (C-2), 160.22 (C-4’’), 138.82 (C-1’’’), 133.93 (C-4’’’), 133.74 (C-6’’’), 133.61 (C-3’’’), 133.55 (C-5’’’), 132.29 (C-1’’), 129.74 (C-2’’ & C-6’’), 115.34 (C-2’’’), 114.37 (C-3’’ & C-5’’), 55.64 (C-7’’), 52.64 (C-2’), 51.55 (C-9’’’), 48.41 (C-6’), 46.19 (C-2’’’’), 33.55 (C-3’), 27.48 (C-4’), 23.64 (C-5’), 21.33 (C-7’’’); EIMS (*m/z*): 553 [M]^+^, 389 [C_17_H_13_N_2_O_5_S_2_]^+^, 348 [C_15_H_12_N_2_O_4_S_2_]^+^, 289 [C_14_H_11_NO_4_S]^+^, 275 [C_14_H_11_O_4_S]^+^, 274 [C_14_H_12_NO_3_S]^+^, 248 [C_13_H_12_O_3_S]^+^, 206 [C_11_H_12_NO_3_]^+^, 150 [C_8_H_8_N_2_]^+^, 65 [C_5_H_5_]^+^, 52 [C_4_H_4_]^+^.

### Lipoxygenase Inhibition assay

Reported method^46,47^ with minor modifications was employed to perform the lipoxygenase inhibition assay. A reaction mixture of 200 µL containing 10 µL of a tested compound, 15 µL of lipoxygenase enzyme (600 units well^− 1^), and 150 µL of 100 mM of sodium phosphate buffer of pH 8 was prepared. The reactants were mixed thoroughly and pre-read at 234 nm. Pre-incubation was performed at 25 °C for 10 min. 25 µL of substrate were added as a starter to initiate the reaction. After 6 min, change in absorbance was recorded at 234 nm with the help of a microplate reader, i.e., Synergy, Biotek, USA. Experiments were performed thrice. Positive control used was quercetin. The following formula was used to calculate the inhibition %:

$${\mathrm{Inhibition~}}\left( {{\% }} \right)=\frac{{{\mathrm{Control}} - {\mathrm{Test}}}}{{{\mathrm{Control}}}} \times 100$$

Control is absorbance with reference and test is absorbance without reference.

### α-Glucosidase inhibition assay

Inspection of α-glucosidase inhibition was made by using protocol mentioned in literature^48^. 10 µl (0.057 units) of purified enzyme, 10 µl (0.5 mM) of the test compound and 70 µl (50 mM) of sodium phosphate buffer (pH = 6.8) were collected in a 96-well plate. Pre-incubation for 10 min at 37 °C was performed. Pre-reading was done at 400 nm. 10 µl (0.5 mM) of p-nitrophenyl glucopyranoside were added as a substrate. The standard used for positive control was acarbose. After incubation at 37 °C for half an hour, reading was noted at 400 nm by using synergy HT microplate reader. The collection of all data was made thrice. The formula given above allowed to calculate % of inhibition.

### Urease inhibition assay

The phenol hypochlorite method reported by Weatherburn^49^ was used in the customized form to screen activity of novel compounds in inhibition of the urease enzyme. A reaction mixture containing 5 µl of a test compound, 55 µl of phosphate buffer, 25 µl of urease enzyme, and 15 µl of urea was incubated at 37 °C for 15 min by using a 96-well plate. 100 µl of phenol-hypochlorite mixture was poured in every well (to get colour) and then incubation for 30 min at 37 °C was done. The change in absorbance was measured at 620 nm, and % of inhibition was calculated by using the above formula. Thiourea was a standard drug used in this assay.

### Computational details

#### Density functional theory studies (DFT)

DFT studies were performed with the Gaussian 16 software^51^. We optimized the **7a-7o** molecules without any symmetry constraints and then performed frequency calculations to verify that the optimized structures are true energy minima. All calculations were performed with the hybrid density functional B3LYP^52^ and the triple-zeta split-valence polarized basis set 6-311G(d, p)^53,54^ (two sets of polarization functions, on heavier atoms and on hydrogens). This approach is furthermore referred to as B3LYP/6-311G(d, p). We did the computational investigation and all analyses listed below using the B3LYP/6-311G(d, p) approach with the implicit effects from dimethyl sulfoxide (DMSO) (dielectric constant ε = 46.826) taken into account. The self-reliable IEF-PCM approach^53^ with the UFF default model as implemented in the Gaussian 16 software was used, with the electrostatic scaling factor α = 1.0. Below we consider the calculated structures, frontier molecular orbitals (FMOs), and molecular electrostatic potential (MEP) plots for all compounds. Avogadro, version 1.1.1, was used to visualize the structures, MOs, and MEP maps^56,57^.

#### Molecular Docking methodology

The binding modes of synthesized compound with active site of enzyme were determined by molecular docking studies. Crystal structures of four proteins of four enzymes were downloaded from protein data bank (PDB)^58^. The four enzymes were AChE, urease, alpha-glucosidase and BChE with the ID of proteins as 1C20, 3LA4, 5NN6 and 6ESJ^59,60^. In downloaded structures, water molecules and ligands were removed by cleaning in Biovia Discovery Studio 2021^61^. The structures of synthesized compounds were drawn in ChemDraw^62^. The files were cleaned with root center and grid designing which were performed with spacing to target the whole proteins and their active sites. Protein files were saved as quotient files (pdbqt file) and continued docking using Autodock and Autodock Vina under standard protocols^63,64^. The representative three-dimensional docked poses for the standards and selected synthesized compounds (most active and least active) were visualized and rendered using PyMOL and are presented in Fig. 14. To further examine residue-level interactions, the docked complexes were evaluated using the Protein–Ligand Interaction Profiler (PLIP). The PLIP-generated diagrams, which detail hydrogen bonds, hydrophobic interactions, π contacts, and salt bridges, are provided as Supporting Information in Figure S46. Together, the PyMOL-rendered 3D poses and PLIP diagrams provide a complete structural interpretation of ligand–protein interactions across all three enzyme systems.

## Supplementary Information

Below is the link to the electronic supplementary material.

Supplementary Material 1

Supplementary Material 2

## Data Availability

The data that support the findings of this study are available in the supplementary material of this article.

## References

[1] Banerjee, R., Roy, D. & Banerjee, M. Synthesis wound healing and diuretic activity of some new 1, 3, 4 thiadiazole derivatives. *World J. Pharm. Pharm. Sci.* **4** (11), 1769–1778 (2015).

[2] Tabatabai, S. A., Lashkari, S. B., Zarrindast, M. R., Gholibeikian, M. & Shafiee, A. Design synthesis and anticonvulsant activity of 2-(2-phenoxy) phenyl- 1,3,4-oxadiazole derivatives. *Iran. J. Pharm. Res.* **12**, 105–111 (2013). 24250678 PMC3813377

[3] Almasirad, A., Abbas Shafiee, A. & Abdollahi, M. Synthesis and analgesic activity of new 1,3,4-oxadiazoles and 1,2,4-triazoles. *J. Med. Chem.* **20** (4), 435–442 (2011).

[4] Shafi, S. S. & Radhakrishnan, T. R. Studies on biologicall active heterocycles. Part I. Synthesis and antibacterial activity of some 2,5-di-substituted-1,3,4-oxadiazole, 1,3,4thiadiazole, 1,2,4-triazole and 4-thiazolidinone. *Indian J. Heterocy Chem.* **5**, 133–138 (1995).

[5] Ma, L. et al. Synthesis and antioxidant activity of novel Mannich base of 1,3,4-oxadiazole derivatives possessing 1,4-benzodioxan. *Bioorg. Med. Chem.* **21** (21), 6763–6770 (2013). 23993673 10.1016/j.bmc.2013.08.002

[6] Almasirad, A., Vousooghi, N., Tabatabai, S. A., Kebriaeezadeh, A. & Shafee, A. Synthesis, anticonvulsant and muscle relaxant activities of substituted 1,3,4-oxadiazole, 1,3,4-thiadiazole and 1,2,4-triazole. *Acta Chim. Slov.* **54**, 317–324 (2007).

[7] Zareef, M. et al. Enzyme Inhib. Synthesis and antimalarial activity of novel chiral and achiral benzenesulfonamides bearing 1, 3, 4-oxadiazole moieties. *Med. Chem.* **22** (3), 301–308 (2007). 10.1080/14756360601114569 17674812

[8] Burbuliene, M. M. et al. Synthesis and anti-inflammatory activity of derivatives of 5-((2-disubstitutedamino-6-methyl-pyrimidin-4-yl)sulfanylmethyl)-3H-1,3,4-oxadiazole-2-thiones. *Farmaco* **59**, 767–774 (2004). 15474053 10.1016/j.farmac.2004.05.007

[9] Duan, W. G., Li, X. R., Mo, Q. J. & Huang, J. X. Synthesis and herbicidal activity of 5-dehydroabietyl-1,3,4-oxadiazole derivatives. *Holzforschung*. **65**(2), 191–197 (2011).

[10] Hajimahdi, Z., Zarghi, A., Zabihollahi, R. & Aghasadeghi, M. R. Synthesis, biological evaluation, and molecular modeling studies of new 1, 3, 4-oxadiazole and 1, 3, 4-thiadiazole-substituted 4-oxo-4H-pyrido[1,2-a] pyrimidines as anti-HIV-1 agents. *Med. Chem. Res.* **22** (5), 2467–2475 (2013).

[11] Joshi, S. D., Vagdevi, H. M., Vaidya, V. P. & Gadaginamatha, G. S. Synthesis of new 4-pyrrol-1-yl-benzoic acid Hydrazide analogs and some derived oxadiazole, Triazole and pyrrole ring system: a novel class of potential anti- bacterial and anti- tubercular agents. *Eur. J. Med. Chem.* **43**, 1989–1996 (2008). 18207286 10.1016/j.ejmech.2007.11.016

[12] Cao, S., Qian, X., Song, G. & Huang, Q. Synthesis and insecticidal activity of new 2-(5-(trifluoromethyl)pyridyloxymethyl)-1,3,4-oxadiazoles. *J. Fluor. Chem.* **117** (1), 63–66 (2002).

[13] Maslat, Ahmed, O., Abussaud, M., Tashtoush, H. & Mahmoud, T. Synthesis antibacterial, antifungal and genotoxic activity of bis-1,3,4-oxadiazole derivatives. *Pol. J. Pharmacol.* **54**, 55–59 (2002). 12020044

[14] Wu, W., Chen, Q., Tai, A., Jiang, G. & Ouyang, G. Synthesis and antiviral activity of 2-substituted methylthio-5-(4-amino-2-methylpyrimidin-5-yl)-1,3,4-oxadiazole derivatives. *Bioorg. Med. Chem. Lett.* **25** (10), 2243–2246 (2015). 25900217 10.1016/j.bmcl.2015.02.069

[15] Kosikowskaand, P. & Berlicki, L. Urease inhibitors as potential drugs for gastric and urinary tract infections: a patent review. *Expert Opin. Ther. Pat.* **21**, 945–957 (2011). 21457123 10.1517/13543776.2011.574615

[16] Krajewska, B. & Ureases, I. Functional, catalytic and kinetic properties: a review. *J. Mol. Catal. B: Enzym*. **59**, 9–21 (2009).

[17] Beraldoand, H. & Gambinob, D. The wide Pharmacological versatility of semi-carbazones, thiosemicarbazones and their metal complexes. *Mini Rev. Med. Chem.* **4**, 31–39 (2004). 14754441 10.2174/1389557043487484

[18] Kim, S. α-Glucosidase inhibitor from *Buthus martensi* Karsch. *Food Chem.* **136**, 297–300 (2013). 23122061 10.1016/j.foodchem.2012.08.063

[19] Lebovitz, H. E. Alpha-glucosidase inhibitors, endocrinology and metabolism. *Clin. N Am.* **26**, 539–551 (1997). 10.1016/s0889-8529(05)70266-8 9314014

[20] Alitonou, G. A. et al. Investigations on the essential oil of cymbopogon giganteus from Benin for its potential use as an anti-inflammatory agent. *Int. J. Aromather.* **16**, 37–41 (2006).

[21] Abbasi, M. A. et al. Benzoylsalireposide an anti-oxidant, lipoxygenase and chymotrypsin inhibitor. *Proc. Pakistan Acad. Sci.* **42**, 121–124 (2005).

[22] Steinhilber, D. A target for anti-inflammatory drugs revisited. *Curr. Med. Chem.* **6**, 71–85 (1999). 9873115

[23] Kemal, C., Louis-Flemberg, P., Krupinski-Olsen, R. & Shorter, A. L. Reproductive inactivation of soybean Lipoxygenase activity. *J. Biochem.* **26**, 7064–7072 (1987). 10.1021/bi00396a031 3122826

[24] Jensen, E. C., Ogg, C. & Nickerson, K. Lipoxygenase inhibitors shift the yeast/mycelium dimorphism in ceratocystis Ulmi. *Appl. Environ. Microb.* **58**, 25052508 (1992). 10.1128/aem.58.8.2505-2508.1992 PMC195812 1514797

[25] Clapp, H. C., Banerjee, A. & Rotenberg, S. A. Inhibition of soybean Lipoxygenase by n- alkylhydroxylamines. *J. Biochem.* **24**, 1826–1830 (1985). 10.1021/bi00329a004 2990543

[26] Byrum, R. S., Goulet, J. L., Griffiths, R. J. & Koller, B. H. Role of the 5-lipoxygenase-activating protein (FLAP) in murine acute inflammatory responses. *J. Exp. Med.* **185**, 1065–1076 (1997). 9091580 10.1084/jem.185.6.1065 PMC2196234

[27] Bishayee, A., Karmaker, R., Mandal, A., Kundu, S. N. & Chaterjee, M. Vanadium-mediated chemoprotection against chemical hepatocarcinogenesis in rats: haematological and histological characteristics. *Eur. J. Cancer Prev.* **6**, 58–70 (1997). 9161814 10.1097/00008469-199702000-00010

[28] Nesaragi, A. R. et al. Green synthesis of therapeutically active 1,3,4-oxadiazoles as antioxidants, selective COX-2 inhibitors and their in Silico studies. *Bioorg. Med. Chem. Lett.* **43**, 128112. 10.1016/j.bmcl.2021.128112 (2021). 33991632 10.1016/j.bmcl.2021.128112

[29] Musharraf, S. G. et al. Benzimidazole, coumrindione and flavone derivatives as alternate UV laser desorption ionization (LDI) matrices for peptides analysis. *Chem. Cent. J.* **7** (1), 1–13. 10.1186/1752-153X-7-77 (2013). 23621998 10.1186/1752-153X-7-77 PMC3680071

[30] Mohammed Khan, K. et al. Synthesis and β-glucuronidase inhibitory potential of benzimidazole derivatives. *Med. Chem.* **8** (3), 421–427. 10.2174/1573406411208030421 (2012). 22530898 10.2174/1573406411208030421

[31] Ahmad, S. et al. Novel flurbiprofen clubbed oxadiazole derivatives as potential urease inhibitors and their molecular Docking study. *RSC Adv.* **13** (37), 25717–25728. 10.1039/d3ra03841f (2023). PMID: 37649663; PMCID: PMC10464598. 37649663 10.1039/d3ra03841f PMC10464598

[32] Bondock., S., Adel, S., Etman, H. A. & Badria, F. A. Synthesis and antitumor evaluation of some new 1,3,4-oxadiazole-based heterocycles. *Eur. J. Med. Chem.* **48**, 192–199 (2012). 22204901 10.1016/j.ejmech.2011.12.013

[33] Abdu Musad, E., Mohamed, R., Saeed, B. A., Viswanath, B. S. & Rai, K. M. Synthesis and evaluation of antioxidant and antibacterial activities of new substituted bis(1,3,4-oxadiazoles),3,5-bis(substituted) pyrazoles and isoxazoles. *Bioorg. Med. Chem. Lett.* **21**, 3536–3540 (2011). 21612921 10.1016/j.bmcl.2011.04.142

[34] Somani, R. R. & Bhanushali, U. V. Synthesis and evaluation of anti-inflammatory, analgesic and ulcerogenic potential of NSAIDs bearing 1,3,4-oxadiazole scaffold. *Indian J. Pharm. Sci.* **73** (6), 634–640 (2011). 23112397 10.4103/0250-474X.100237 PMC3480748

[35] Zareef, M. et al. Synthesis and antimalarial activity of novel chiral and achiral benzenesulfonamides bearing 1, 3, 4-oxadiazole moieties. *J. Enzyme Inhib. Med. Chem.* **22** (3), 301–308 (2007). 17674812 10.1080/14756360601114569

[36] Zarghi, A. et al. Synthesis and anticonvulsant activity of new 2-substituted-5-(2-benzyloxyphenyl)-1,3,4-oxadiazoles. *Bioorg. Med. Chem. Lett.* **15** (7), 1863–1865 (2005). 15780622 10.1016/j.bmcl.2005.02.014

[37] Manikandan, V. et al. Synthesis and antimicrobial activities of some (E)-N′-1-(substituted benzylidene)benzohydrazides. *J. Adv. Chem.* **5** (1), 17–24 (2017).

[38] Khalid, H. et al. Synthesis, spectral analysis and anti-bacterial study of *N*-substituted derivatives of 2-(5-(1-(phenylsulfonyl)piperidin-4-yl)-1,3,4-Oxadiazol-2-ylthio)acetamide. *J. Saudi Chem. Soc.* **20**, 615–623 (2016).

[39] Taha, M., Imran, S., Rahim, F., Wadood, A. & Khan, K. M. Oxindole based oxadiazole hybrid analogs: novel a-glucosidase inhibitors. *Bioorg. Chem.* **76**, 273–280 (2018). 29223804 10.1016/j.bioorg.2017.12.001

[40] Taha, M. et al. Synthesis and molecular modelling studies of phenyl linked oxadiazole-phenylhydrazone hybrids as potent antileishmanial agents. *Eur. J. Med. Chem.* **126**, 1021–1033 (2017). 28012342 10.1016/j.ejmech.2016.12.019

[41] Biju, C. R., Ilango, K., Prathap, M. & Rekha, K. Design and microwave-assisted synthesis of 1,3,4-oxadiazole derivatives for analgesic and anti-inflammatory activity. *J. Young Pharm.* **4**, 33–37 (2012). 22523458 10.4103/0975-1483.93576 PMC3326780

[42] Abbasi, M. A., Aziz ur-Rehman, Siddiqui, S. Z., Sheeza, A., Nazir, S. & Ahmad, I. Synthesis, antibacterial and Lipoxygenase Inhibition studies of *N*-(Alkyl/aralkyl)-*N*-(2,3-dihydro-1,4-benzodioxin-6-yl)-4-methylbenzenesulfonamides. *Turk. J. Pharm. Sci.* **14** (1), 49–55 (2017). 32454594 10.4274/tjps.84756 PMC7228002

[43] Haris, H. K. et al. Synthesis of some new biologically active *N*-substituted-2’’- [(phenylsulfonyl)(piperidin-1-yl)amino]acetamide derivatives. *Pak J. Pharm. Sci.* **27** (3), 517–524 (2014). 24811811

[44] Ahmad, S. et al. Novel flurbiprofen clubbed oxadiazole derivatives as potential urease inhibitors and their molecular Docking study. *RSC Adv.* **13**, 25717 (2023). 37649663 10.1039/d3ra03841f PMC10464598

[45] Khan, Y. et al. Identification of novel oxadiazole-based benzothiazole derivatives as potent inhibitors of a-glucosidase and urease: Synthesis, in vitro bio-evaluation and their in Silico molecular Docking study. *J. Saudi Chem. Soc.* **27** (4), 101682 (2023).

[46] Evans, A. T. Actions of cannabis constituents on enzymes of arachidonate metabolism: anti-inflammatory potential. *Bio Pharm.* **36**, 2035–2037 (1987). 10.1016/0006-2952(87)90505-3 3109435

[47] Tappel, A. L. The mechanism of the oxidation of unsaturated fatty acid catalyzed by hematin compounds. *Arch. Biochem. Biophys.* **44** (2), 378–395 (1953). 13058395 10.1016/0003-9861(53)90056-3

[48] Pierre, C., Roland, R. & Tremblay, D. J. Y. p-Nitrophenol-α-D-Glucopyranoside as substrate for measurement of Maltase activity in human semen. *Clin. Chem.* **24**, 208–211 (1978). 23909

[49] Naureen, S. et al. Discovery of indolebased tetraarylimidazoles as potent inhibitors of urease with low antilipoxygenase activity. *Eur. J. Med. Chem.* **102**, 464–470 (2015). 26310891 10.1016/j.ejmech.2015.08.011

[50] Liu, J. et al. Biological evaluation of coumarin derivatives as mushroom tyrosinase inhibitors. *Food Chem.* **135**, 2872–2878 (2012). 22980884 10.1016/j.foodchem.2012.07.055

[51] Frisch, M. J. et al. (2016). J. V., Cioslowski, J., Fox, D. J. Gaussian, Inc. Gaussian 16, Revision B.01, Wallingford CT.

[52] Axel, D. B. Density-functional thermochemistry. III. The role of exact exchange. *J. Chem. Phys.* **98**, 5648–5652 (1993).

[53] McLean, A. D. & Chandler, G. S. Contracted Gaussian-basis sets for molecular calculations. 1. 2nd row atoms, Z = 11–18. *J. Chem. Phys.* **72**, 5639–5648. 10.1063/1.438980 (1980).

[54] Raghavachari, K., Binkley, J. S., Seeger, R. & People, J. A. Self-Consistent molecular orbital Methods. 20. Basis set for correlated wave-functions. *J. Chem. Phys.* **72**, 650–654. 10.1063/1.438955 (1980).

[55] Tomasi, J., Mennucci, B. & Cammi, R. Quantum mechanical continuum solvation models. *Chem. Rev.* **105**, 2999–3093 (2005). 16092826 10.1021/cr9904009

[56] Hanwell, M. D. et al. Avogadro: an advanced semantic chemical editor, visualization, and analysis platform. *J. Cheminformatics*. **41**, 1–17. 10.1186/1758-2946-4-17 (2012). 10.1186/1758-2946-4-17 PMC3542060 22889332

[57] Hanwell, M. D. et al. Avogadro: an open-source molecular builder and visualization tool. *J. Cheminform*. **4** (17), 22889332 (2012). 10.1186/1758-2946-4-17 PMC3542060 22889332

[58] Giessel, J. M., Serbian, I., Loesche, A. & Csuk, R. Substituted cinnamic anhydrides act as selective inhibitors of acetylcholinesterase. *Bioorg. Chem.* **90**, 103058 (2019). 31212181 10.1016/j.bioorg.2019.103058

[59] Rosenberry, T. L. et al. Comparison of the binding of reversible inhibitors to human butyrylcholinesterase and acetylcholinesterase: a crystallographic, kinetic and calorimetric study. *Molecules* **22**, 2098 (2017). 29186056 10.3390/molecules22122098 PMC6149722

[60] Carlini, C. R. & Ligabue-Braun, R. Ureases as multifunctional toxic proteins: a review. *Toxicon* **110**, 90–109 (2016). 26690979 10.1016/j.toxicon.2015.11.020

[61] Dassault Systèmes BIOVIA. *Biovia Discovery Studio Visualizer, v16.1.0* (Dassault Systèmes, 2016).

[62] Taha, M. et al. Synthesis of new urease enzyme inhibitors as antiulcer drug and computational study. *J. Biomol. Struct. Dyn.* **40**, 8232–8247 (2022). 33860726 10.1080/07391102.2021.1910072

[63] Morris, G. M. et al. AutoDock4 and AutoDockTools4: automated docking with selective receptor flexibility. *J. Comput. Chem.* **30**, 2785–2791 (2009). 19399780 10.1002/jcc.21256 PMC2760638

[64] Trott, O. & Olson, A. J. AutoDock vina: improving the speed and accuracy of Docking with a new scoring function, efficient optimization, and multithreading. *J. Comput. Chem.* **31**, 455–461 (2010). 19499576 10.1002/jcc.21334 PMC3041641

