# Supplementary material for: Structural and computational supported development of 2,5-disubstituted-1,3,4-oxadiazole analogues as active LOX, urease, and α-glucosidase inhibitors
Source: Sci Rep. 2026 Jan 21;16:5866. doi: 10.1038/s41598-026-35499-1 (PMC12895000; doi:10.1038/s41598-026-35499-1)
Supplement: Supplementary file 2 — Supplementary Material 2 [file 41598_2026_35499_MOESM2_ESM.docx]

**Structural and computational supported development of 2,5-disubstituted-1,3,4-oxadiazole analogues as active LOX, urease, and α-glucosidase inhibitors**

|  |
| --- |

Jamila Javid^a^, Aziz-ur-Rehman^a^******, Javed Iqbal^b^*****, Ijaz Ahmed^c^, Nadia Bhatti^b^, Aleksey Kuznetsov^d^, Fatiqa Zafar^b^, Muhammad Adnan Ayub^b^, Osama A. Mohammed^e^, Samiah H. Al-Mijalli^f^, Munawar Iqbal^g,h^, Syed A. Ali Shah^i,j^

^a^*Department of Chemistry, Government College University, Lahore-54000, Pakistan.*

^b^*Department of Chemistry, University of Sahiwal, Sahiwal-57000, Pakistan.*

*^c^School of Chemistry, University of the Punjab Lahore, 54590, Pakistan.*

*^d^Departamento de Química, Campus Santiago Vitacura, Universidad Técnica Federico Santa María, Vitacura 7660251, Santiago, Chile.*

*^e^ Department of Pharmacology, College of Medicine, University of Bisha, Bisha, 61922, Saudi Arabia*

*^f^Department of Biology, College of Sciences, Princess Nourah bint Abdulrahman University, P.O. Box 84428, Riyadh 11671, Saudi Arabia.*

*^g^ School of Chemistry, University of the Punjab, Lahore 54000, Punjab, Pakistan.*

*^h^Renewable Energy and Environmental Technology Center, University of Tabuk, Tabuk, 47913, Saudi Arabia*

*^i^Faculty of Pharmacy, Universiti Teknologi MARA Cawangan Selangor Kampus Puncak Alam, Bandar Puncak Alam 42300, Selangor, Malaysia.*

*^j^Atta-ur-Rahman Institute for Natural Product Discovery (AuRIns), Universiti Teknologi MARA Cawangan Selangor Kampus Puncak Alam, Bandar Puncak Alam 42300, Selangor, Malaysia.*

*Corresponding Author: Dr. Javed Iqbal, E-mail: [javediqbal.chemist@gmail.com](mailto:javediqbal.chemist@gmail.com);

[javediqbal@uosahiwal.edu.pk](mailto:javediqbal@uosahiwal.edu.pk); Tel: (+92)-3457593602 Ext. 449.

**DETAILED SUPPLEMENTARY INFORMATION**

**^1^H and ^13^C-NMR SPECTRA OF ALL SYNTHESIZED COMPOUNDS**


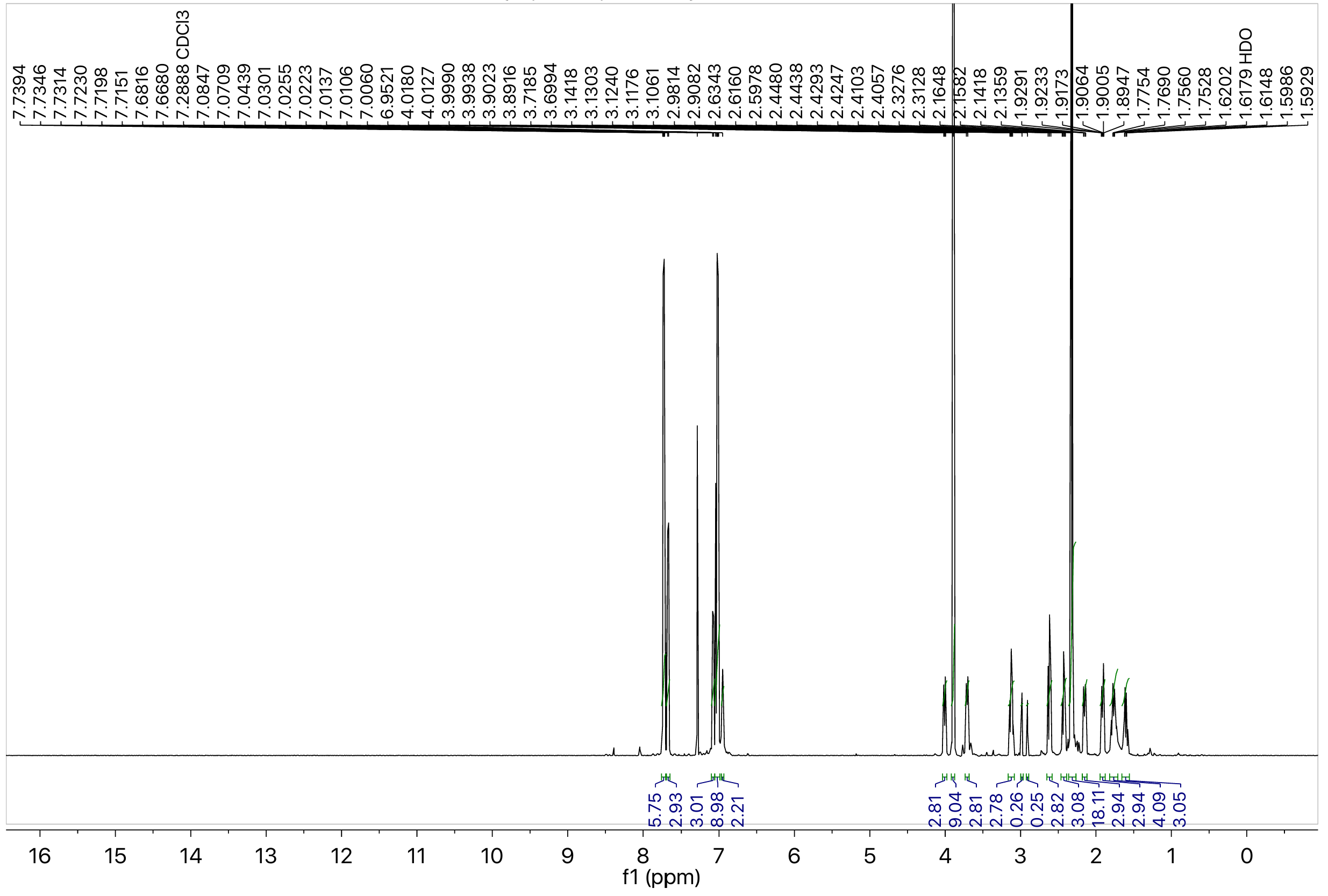


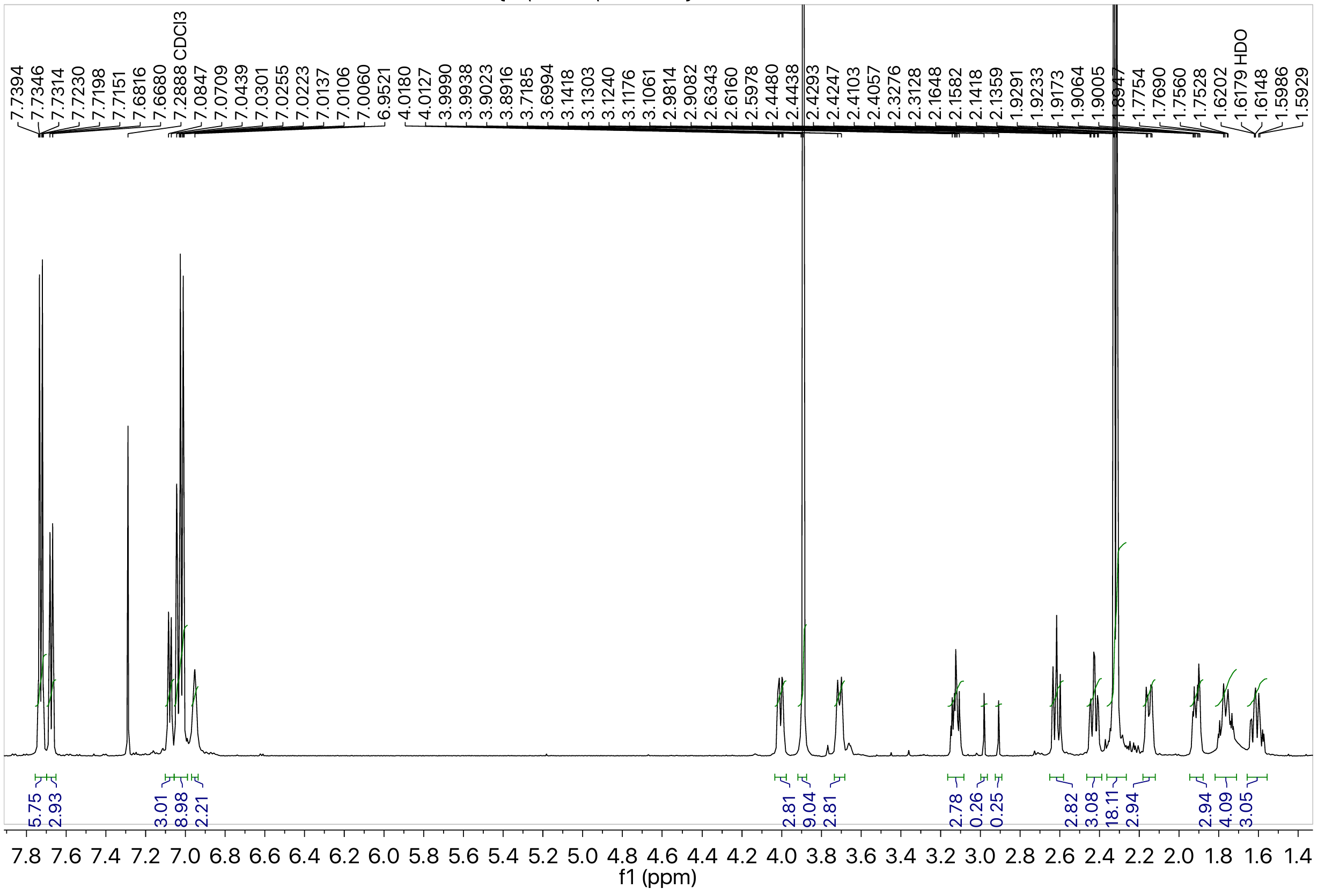


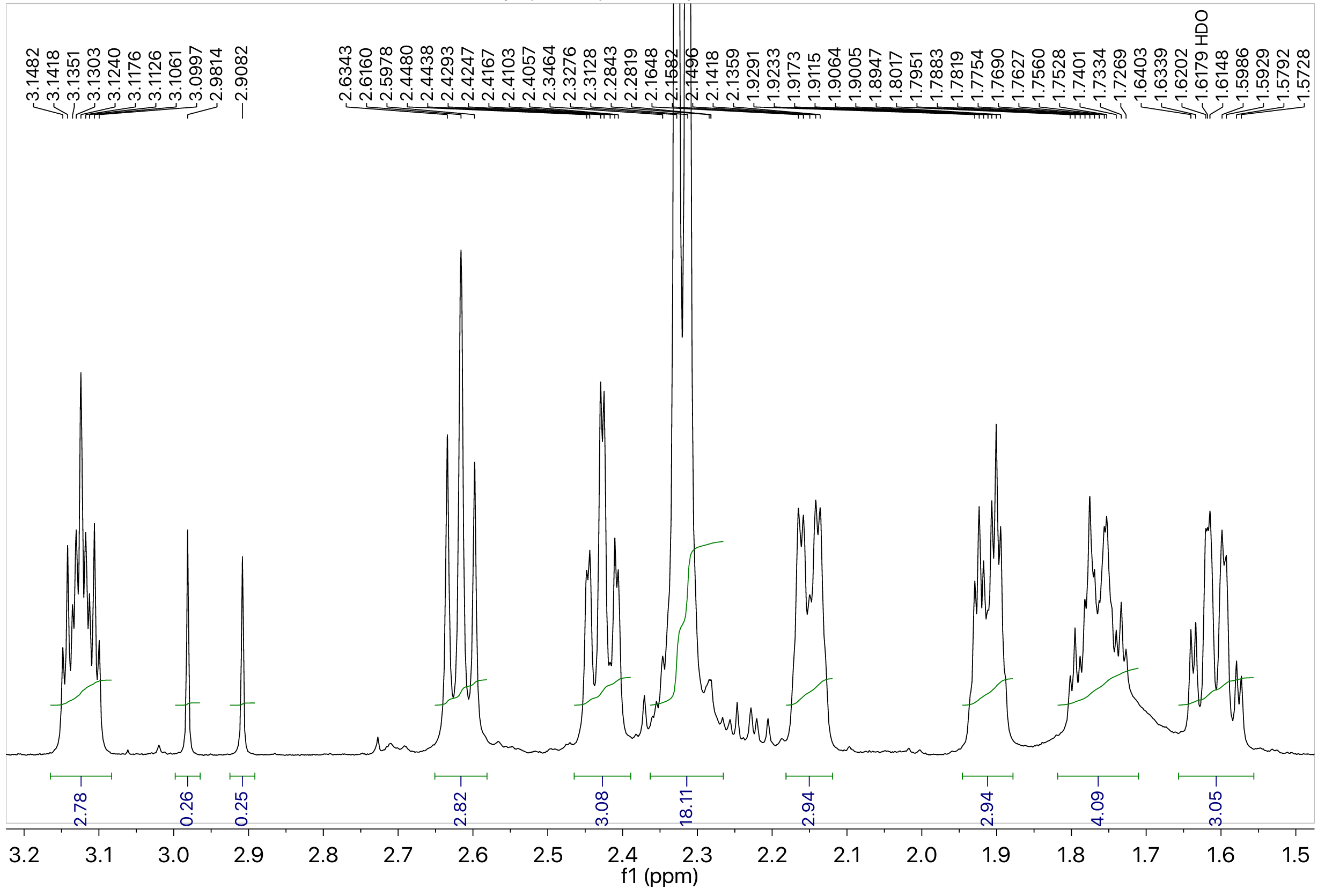


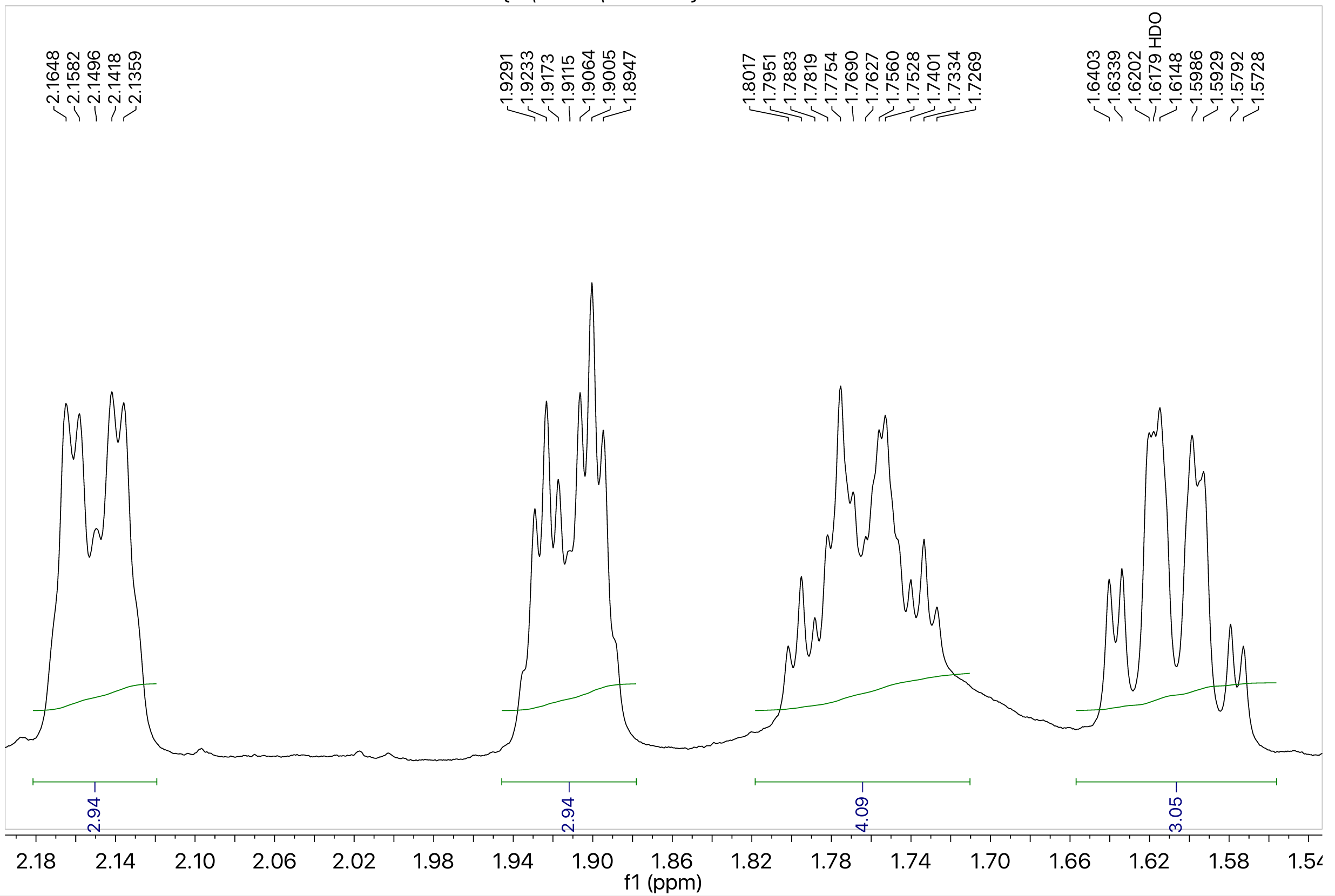


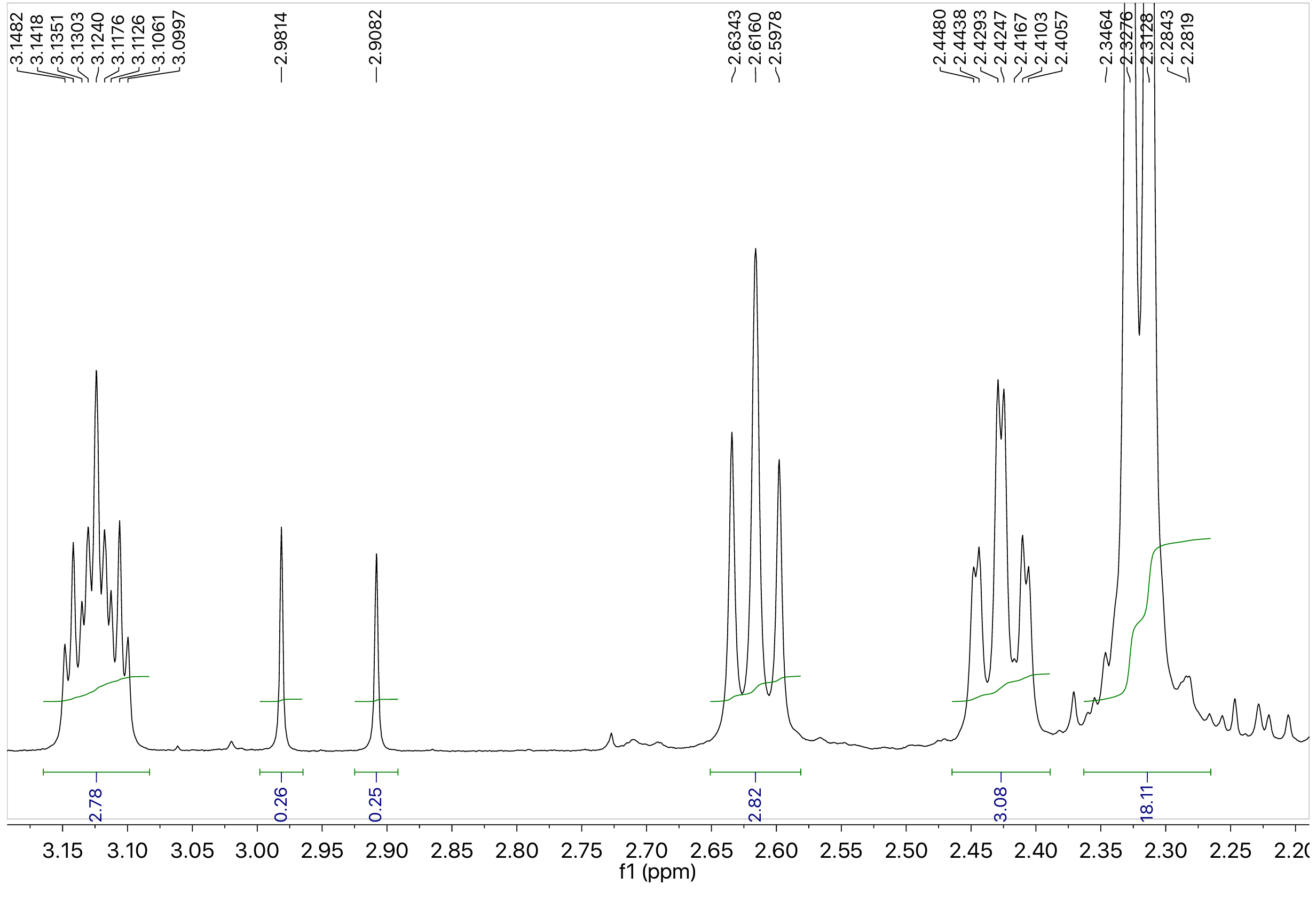


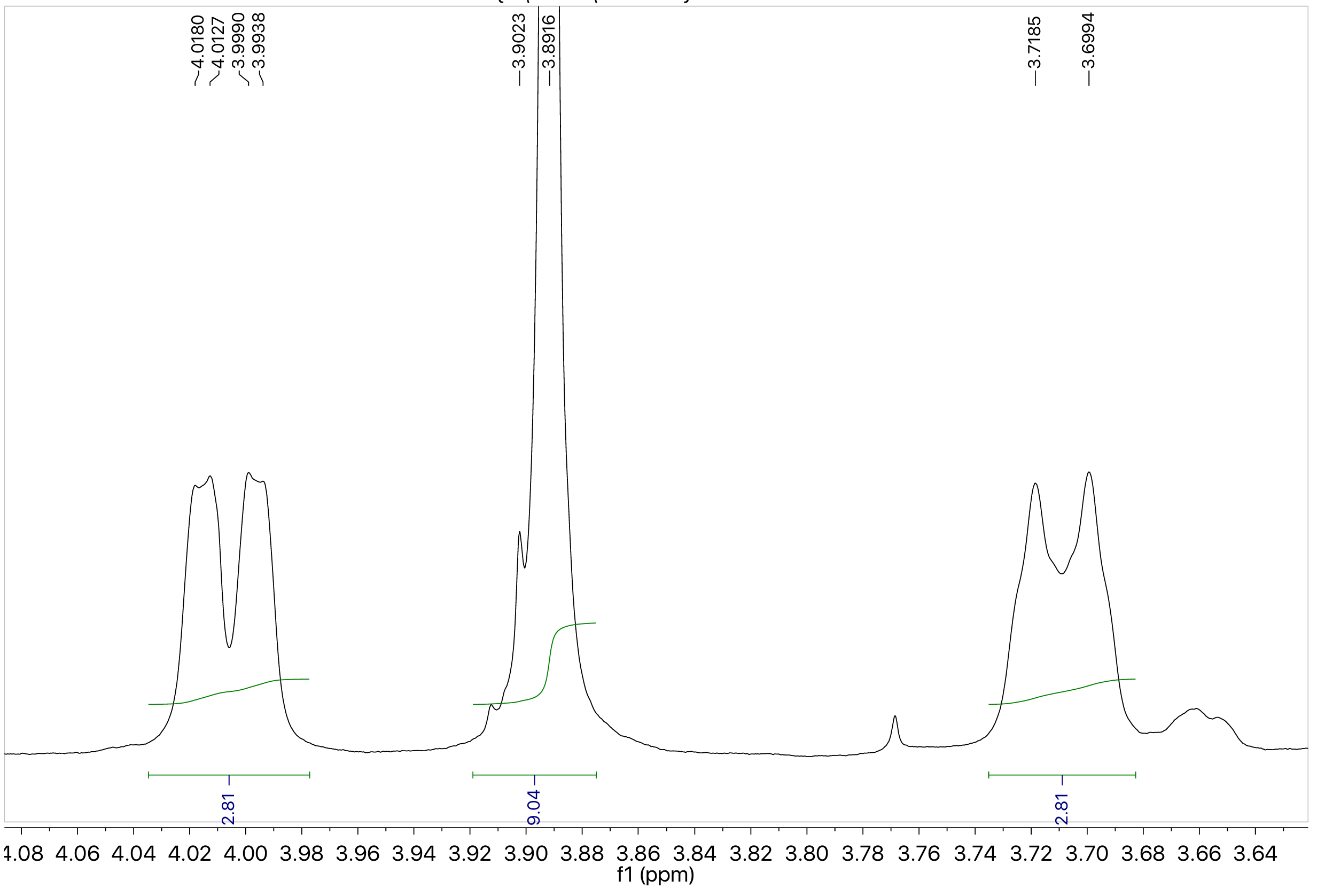


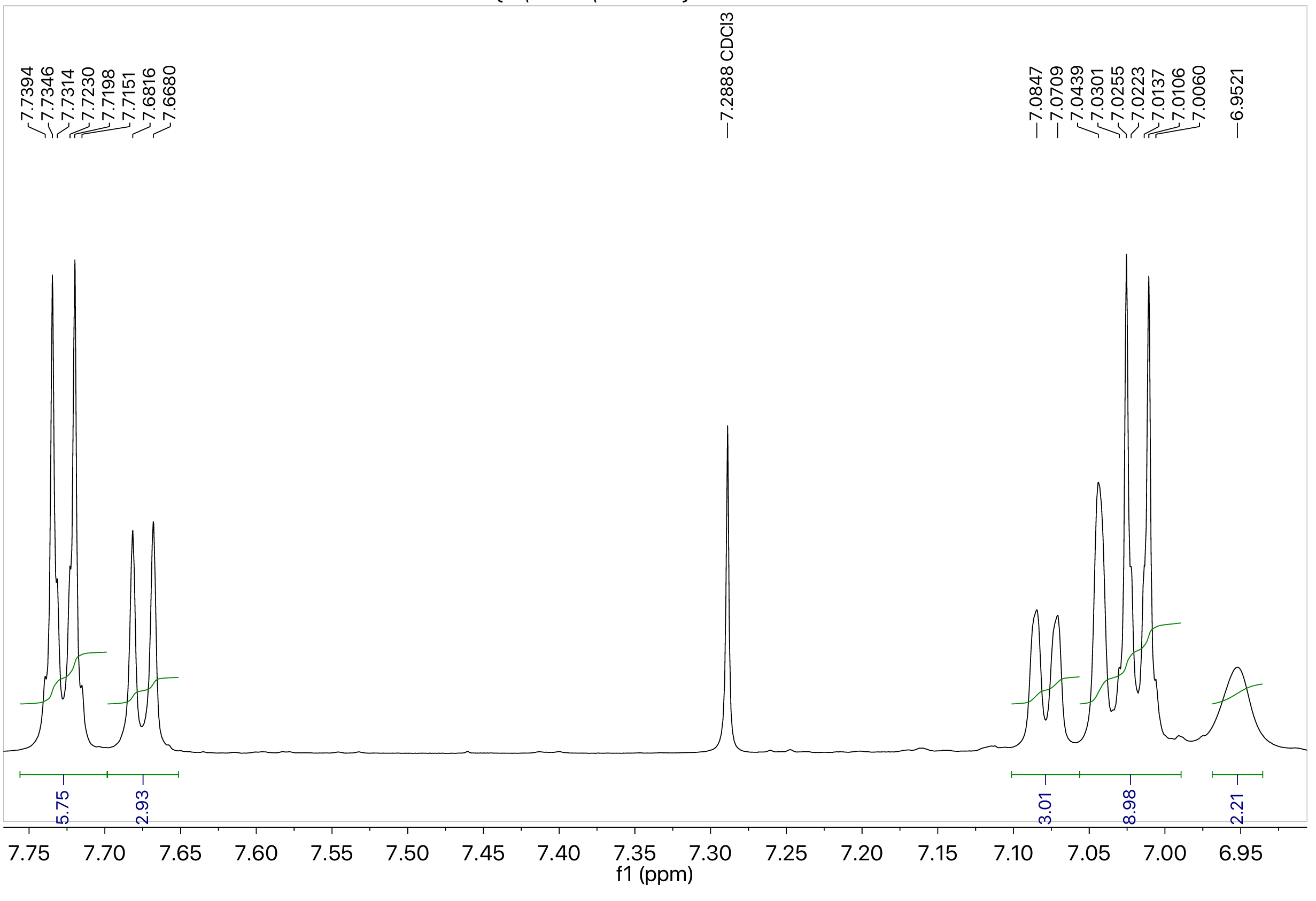


**Figure S1:** HNMR spectrum of synthesized compound **H-NMR of compound 7a**

**
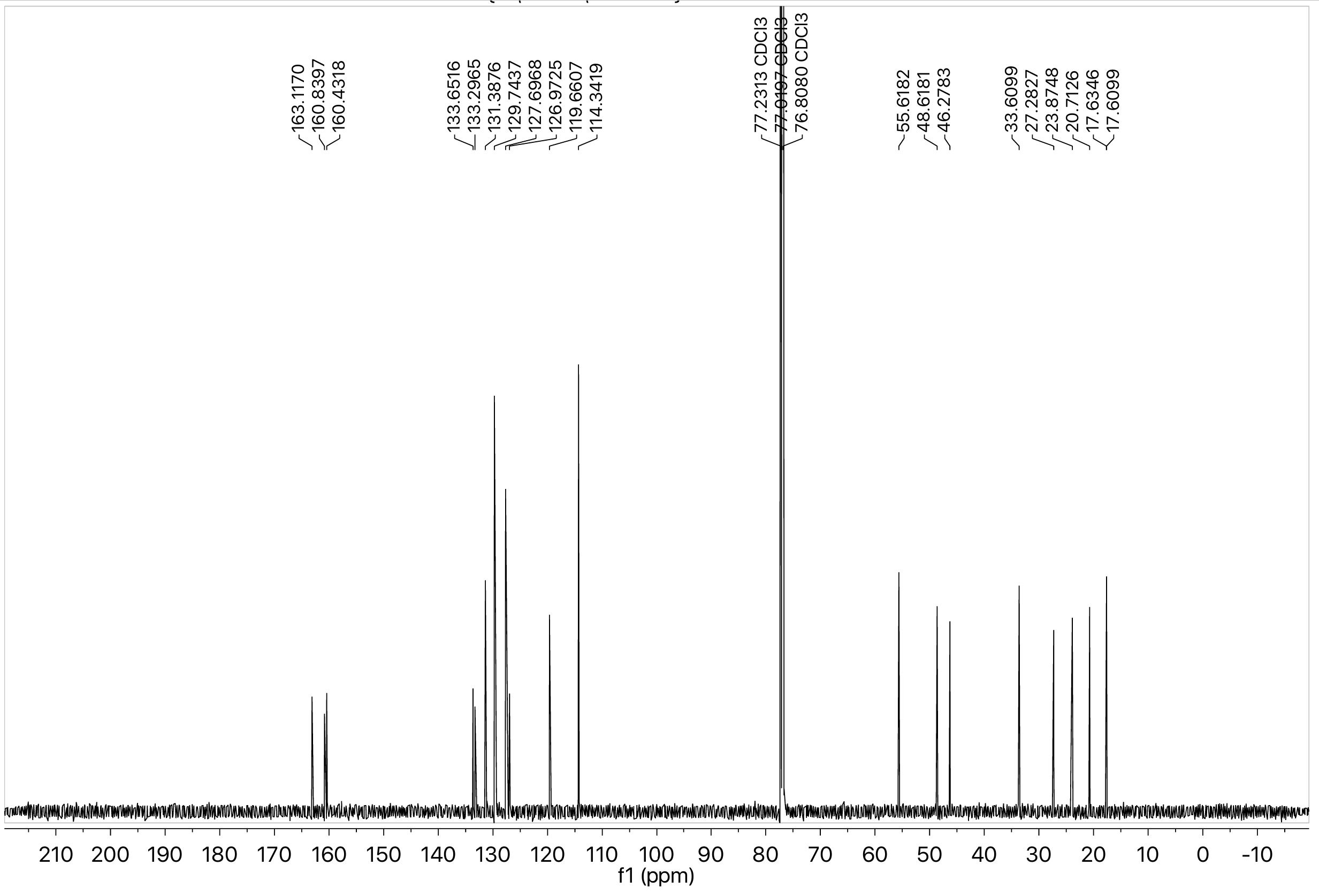
**

**
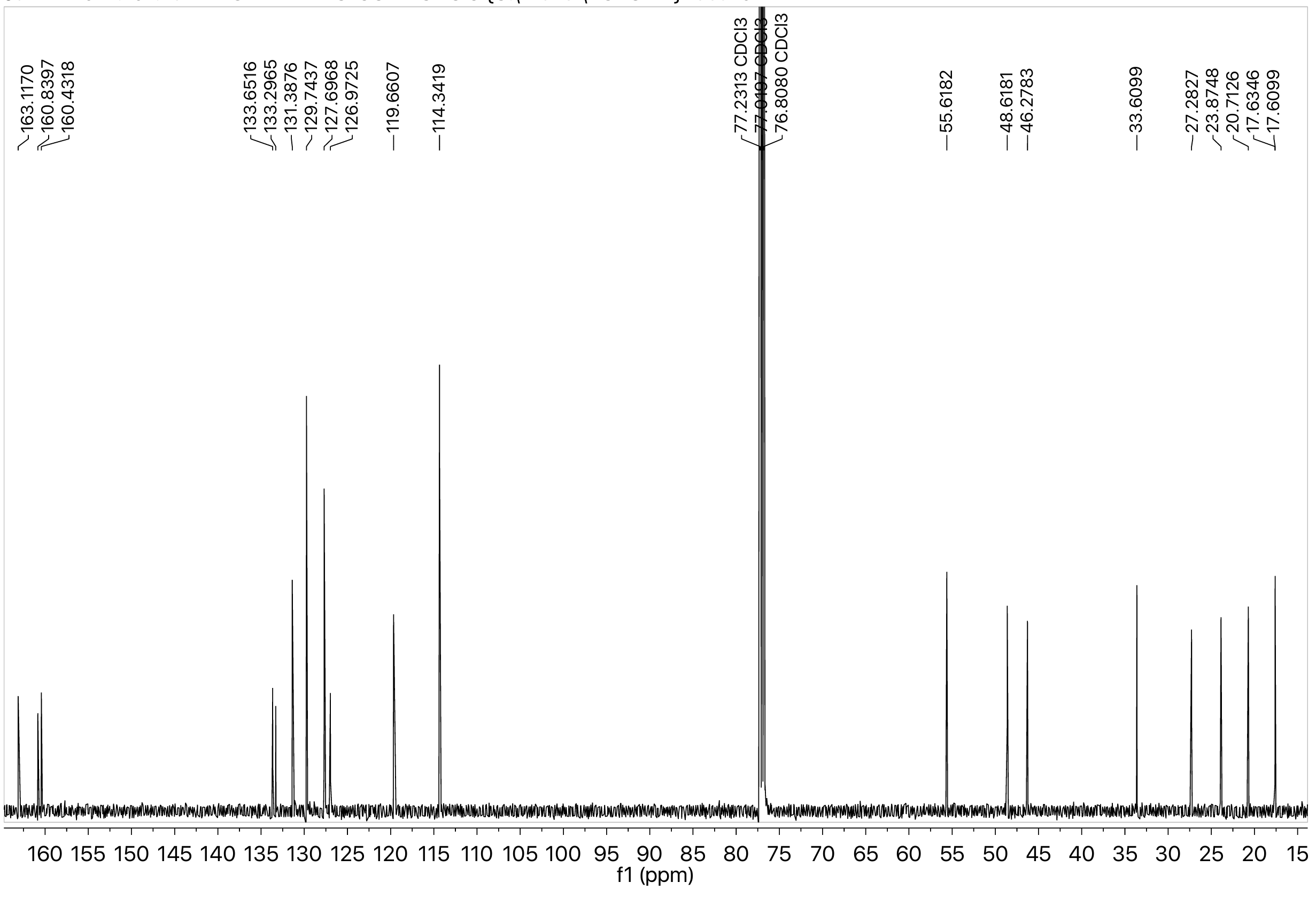
**

**
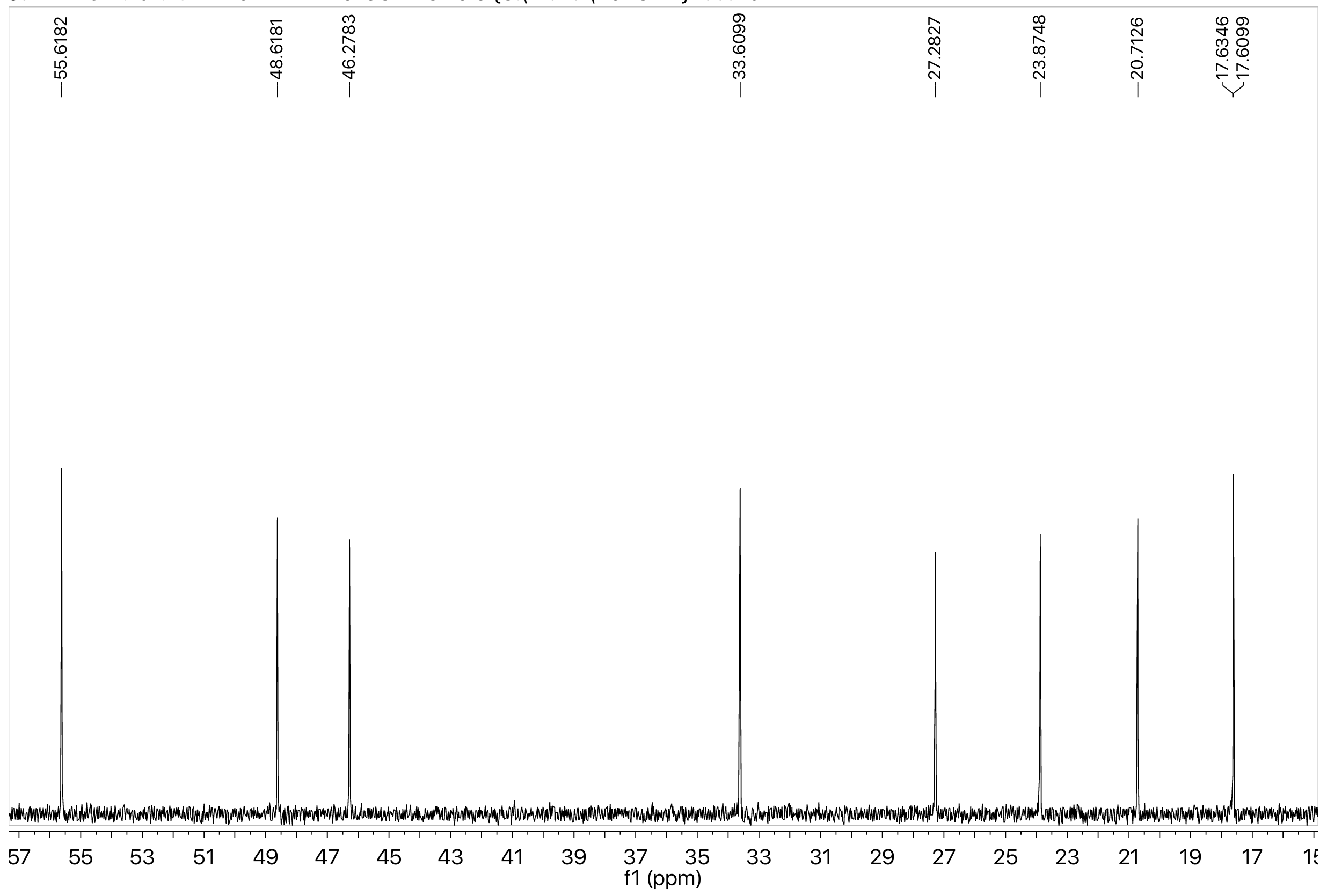
**

**
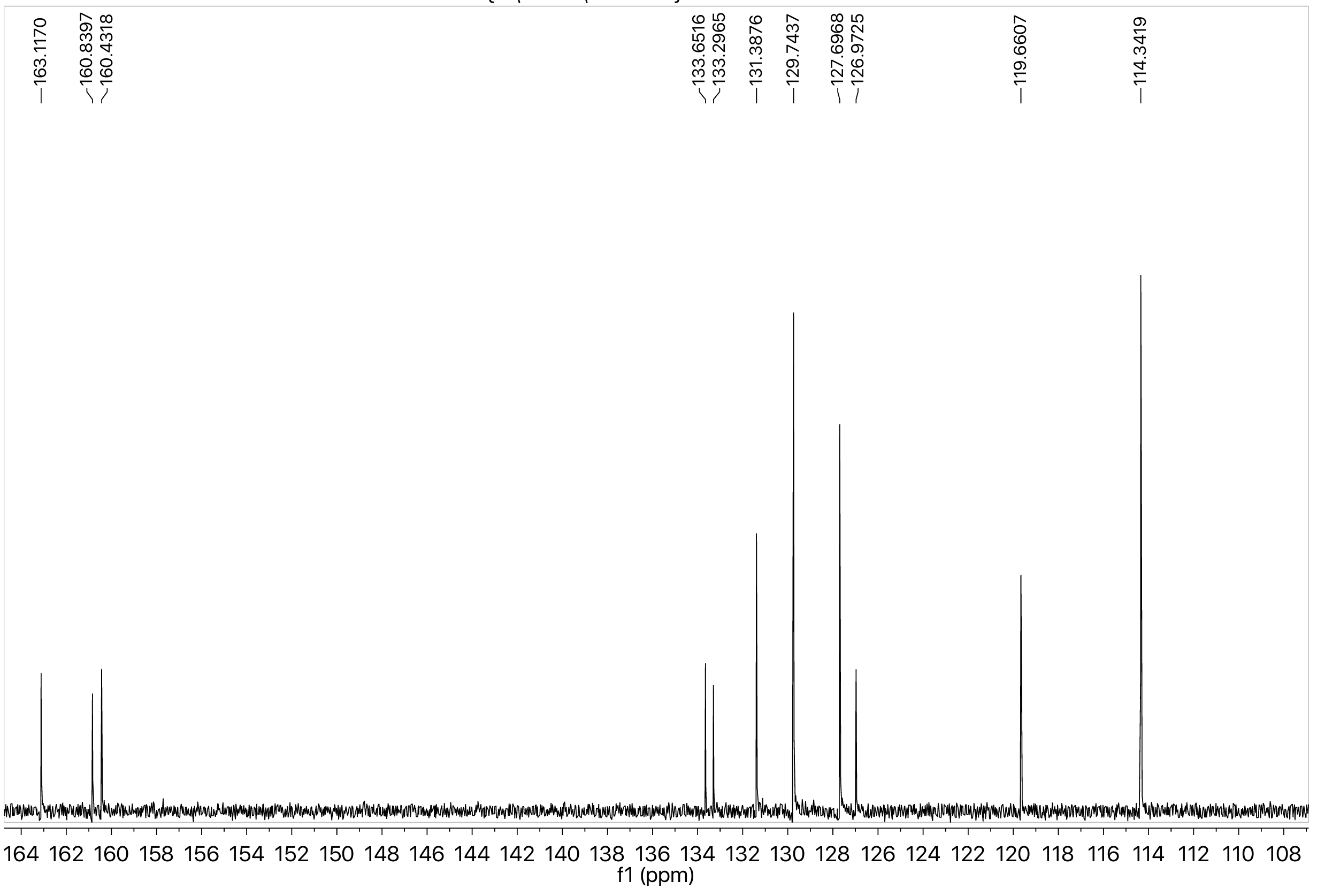
**

**
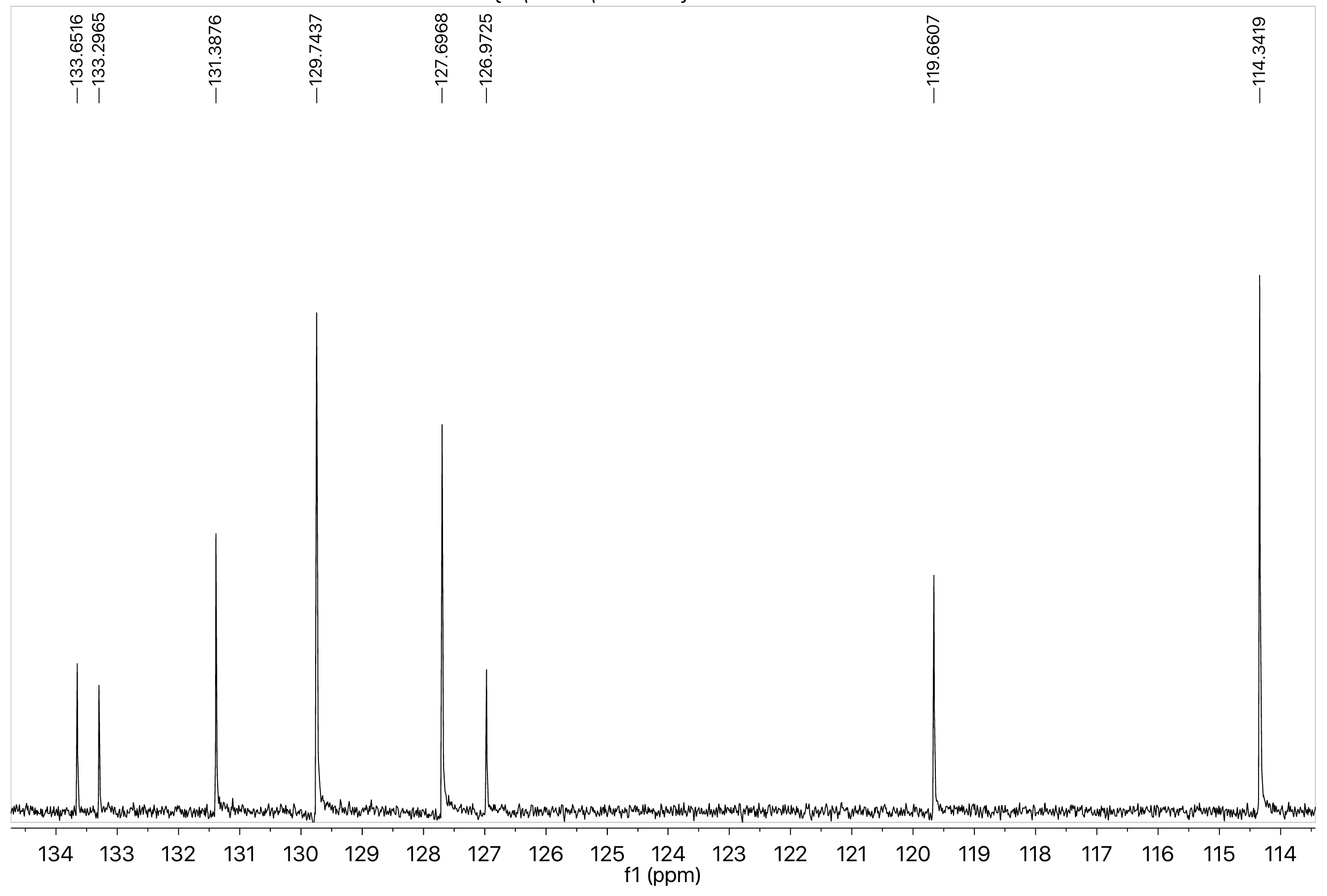
**

**
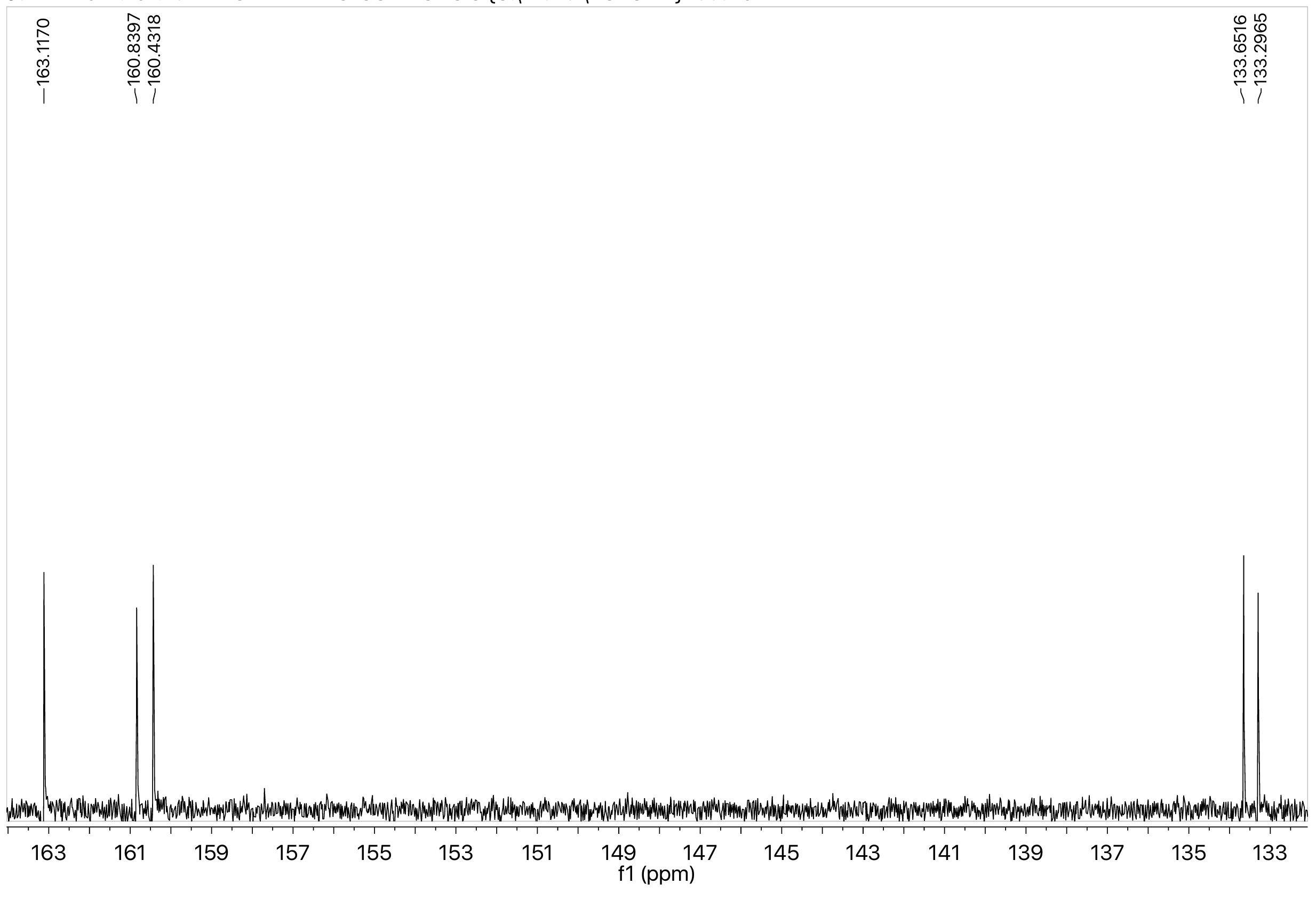
**

**Figure S2:** 13C-NMR spectrum of synthesized compound **7a**


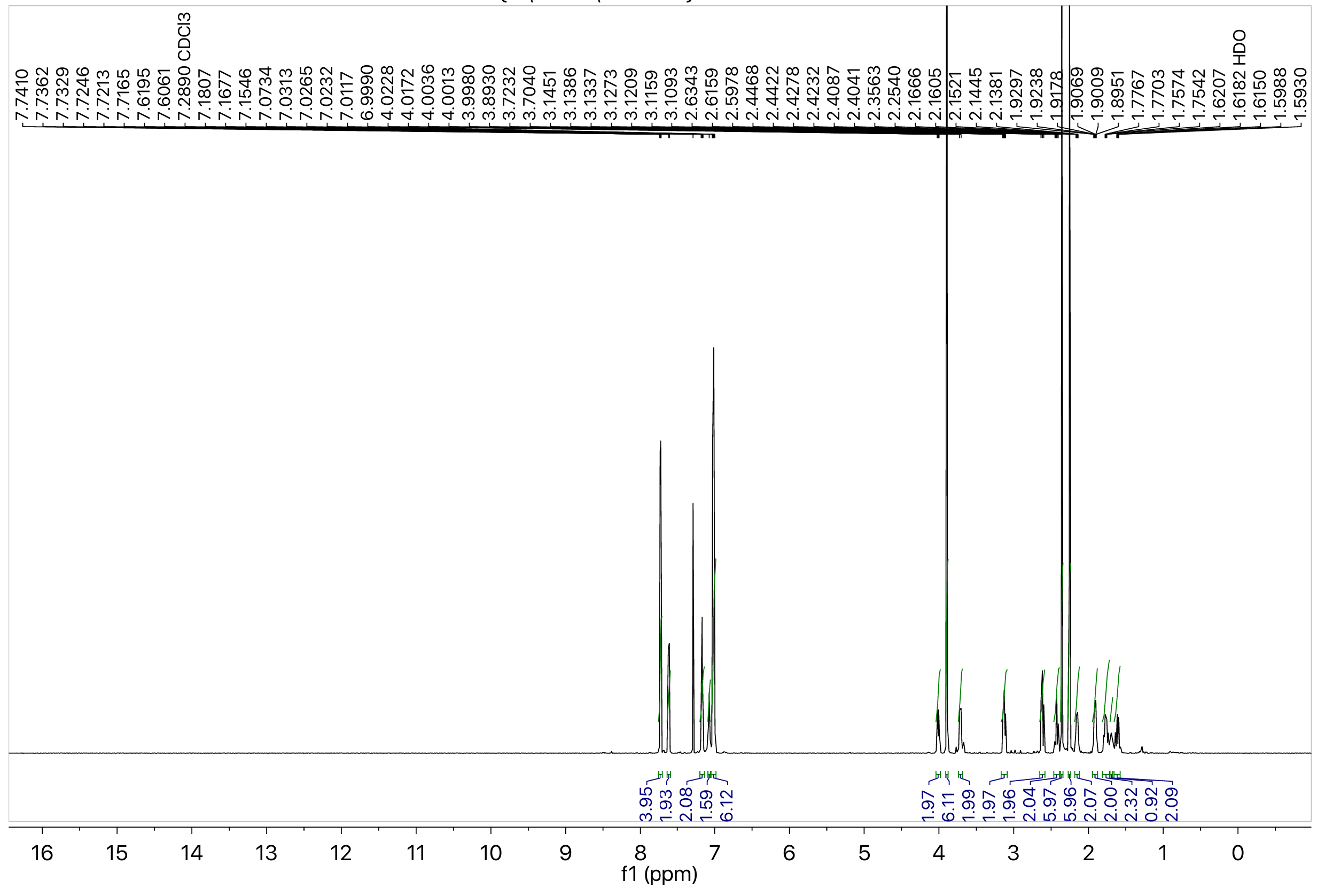


**Figure S3: 1H-NMR spectrum of compound 7b**

**
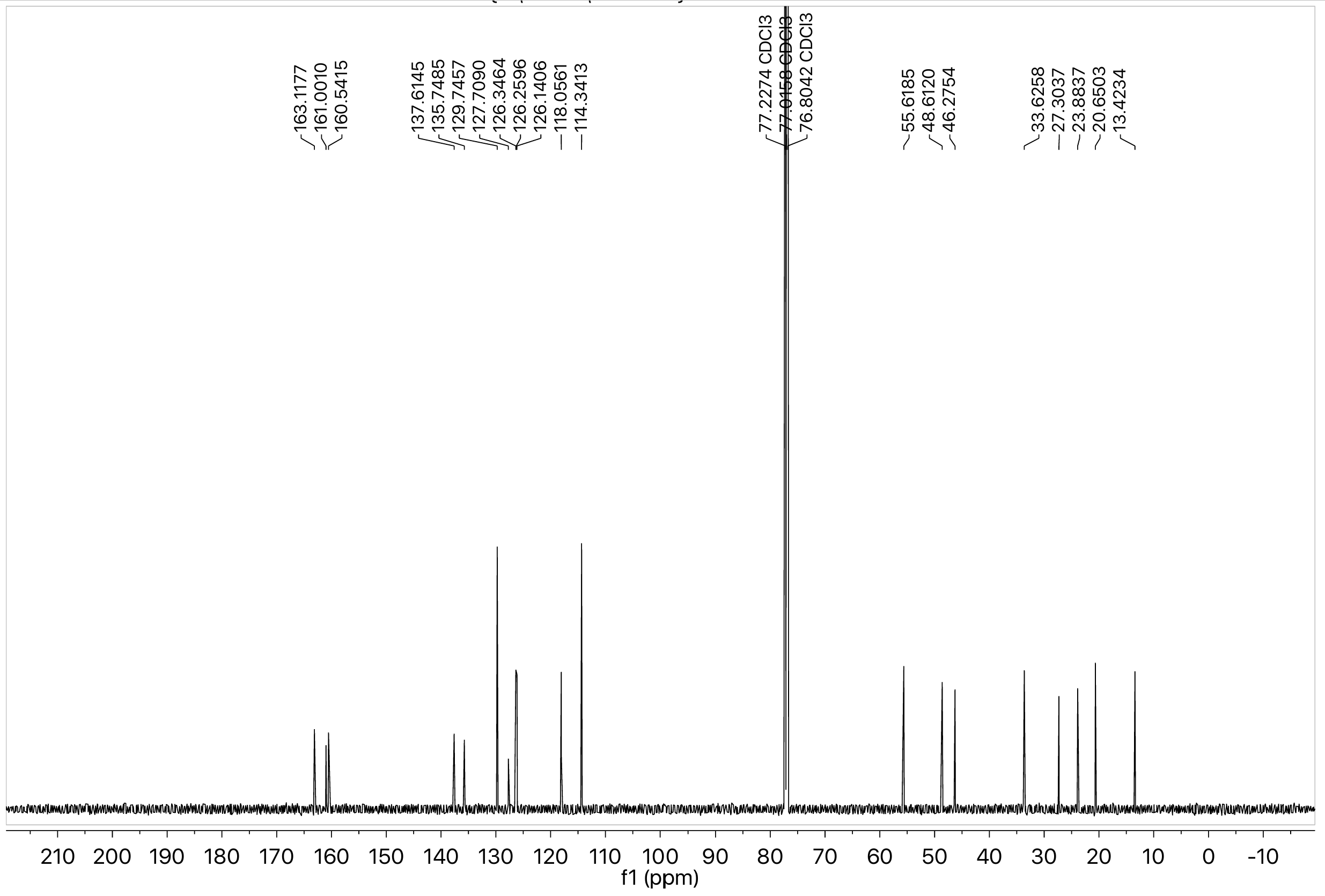
**

**Figure S4: 13C-NMR spectrum of compound 7b**


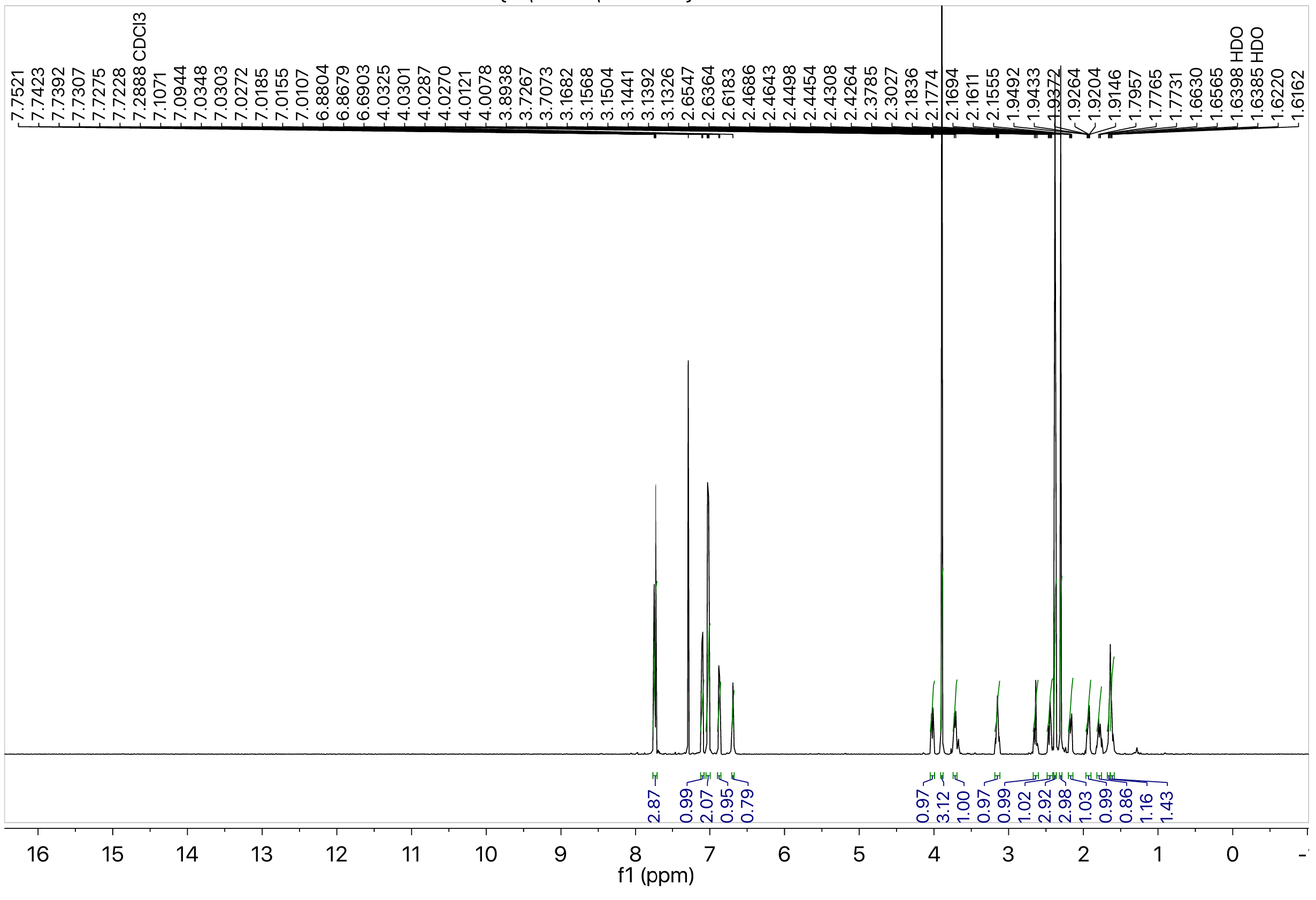


**Figure S5: 1H-NMR spectrum of compound 7c**


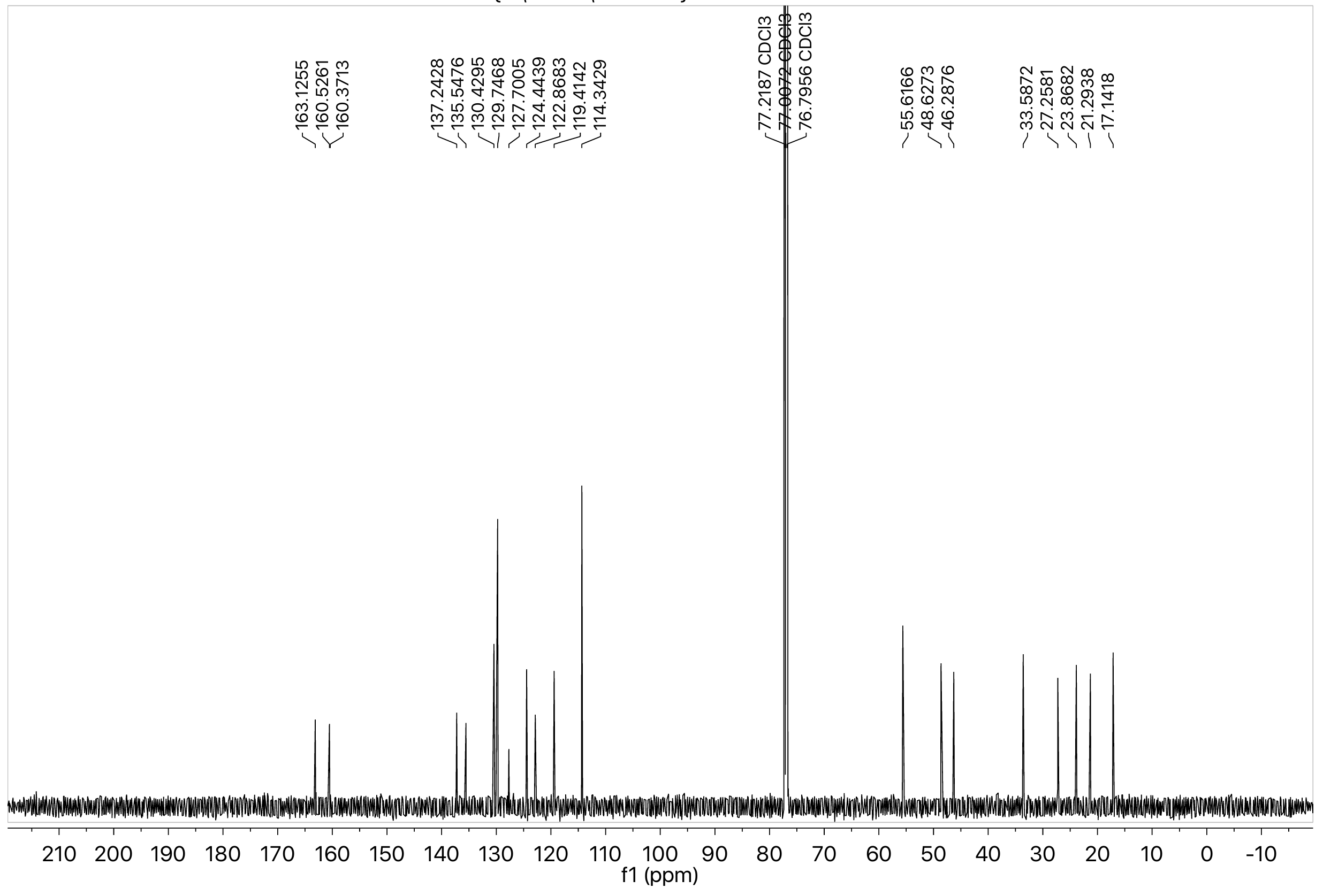


**Figure S6: 13C-NMR spectrum of compound 7c**


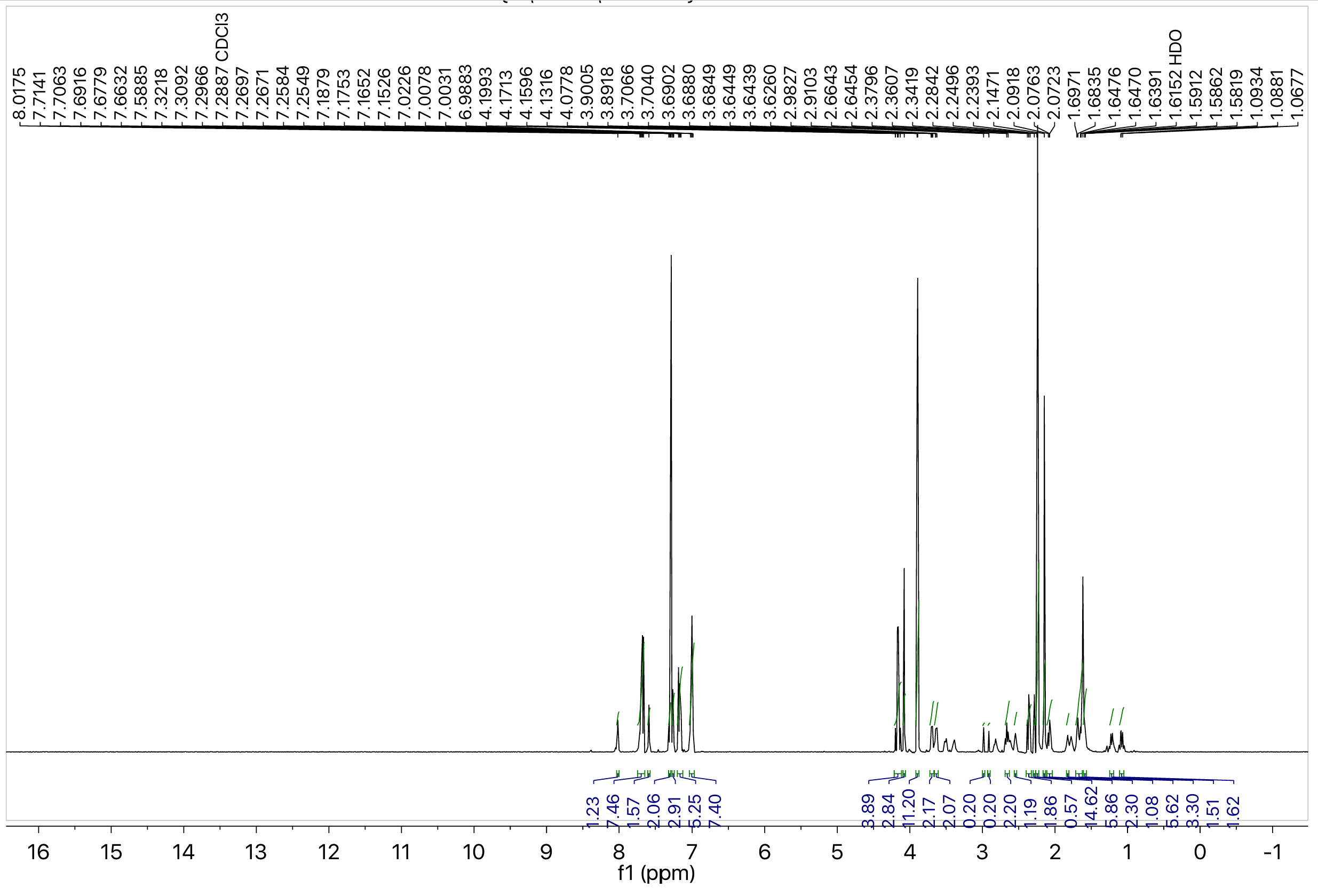


**Figure S7: 1H-NMR spectrum of compound 7d**


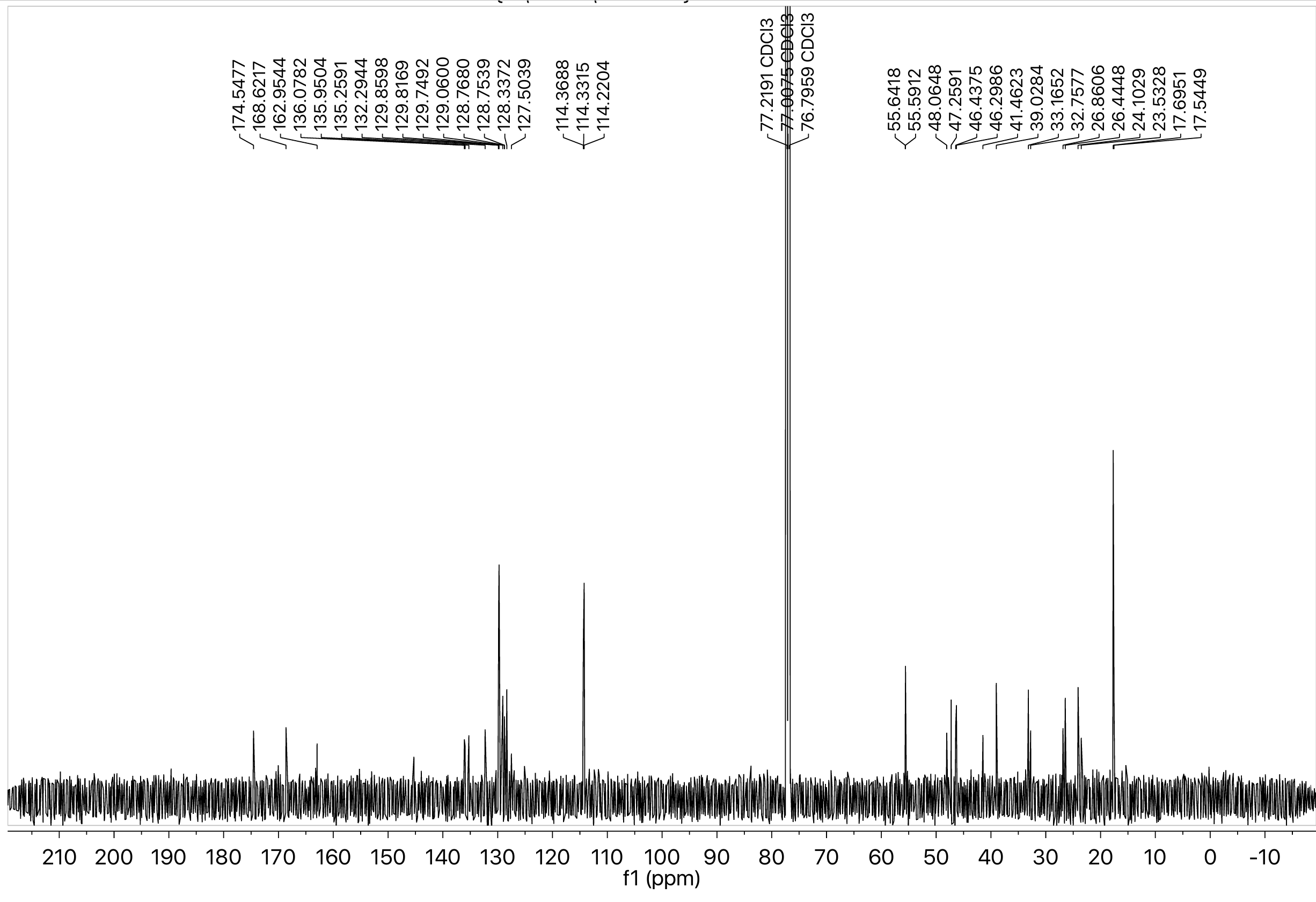


**Figure S8: 13C-NMR spectrum of compound 7d**


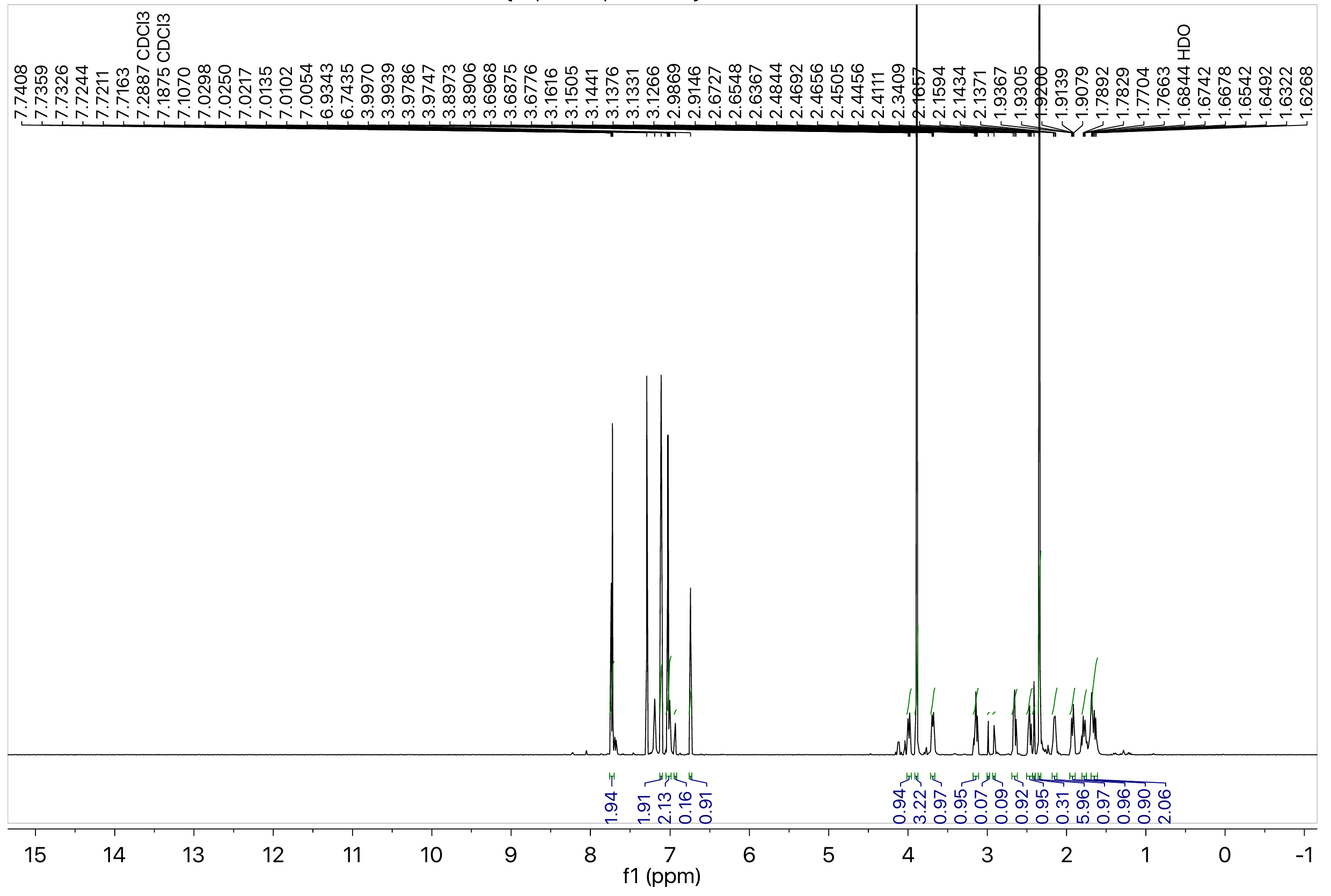


**Figure S9: 1H-NMR spectrum of compound 7e**


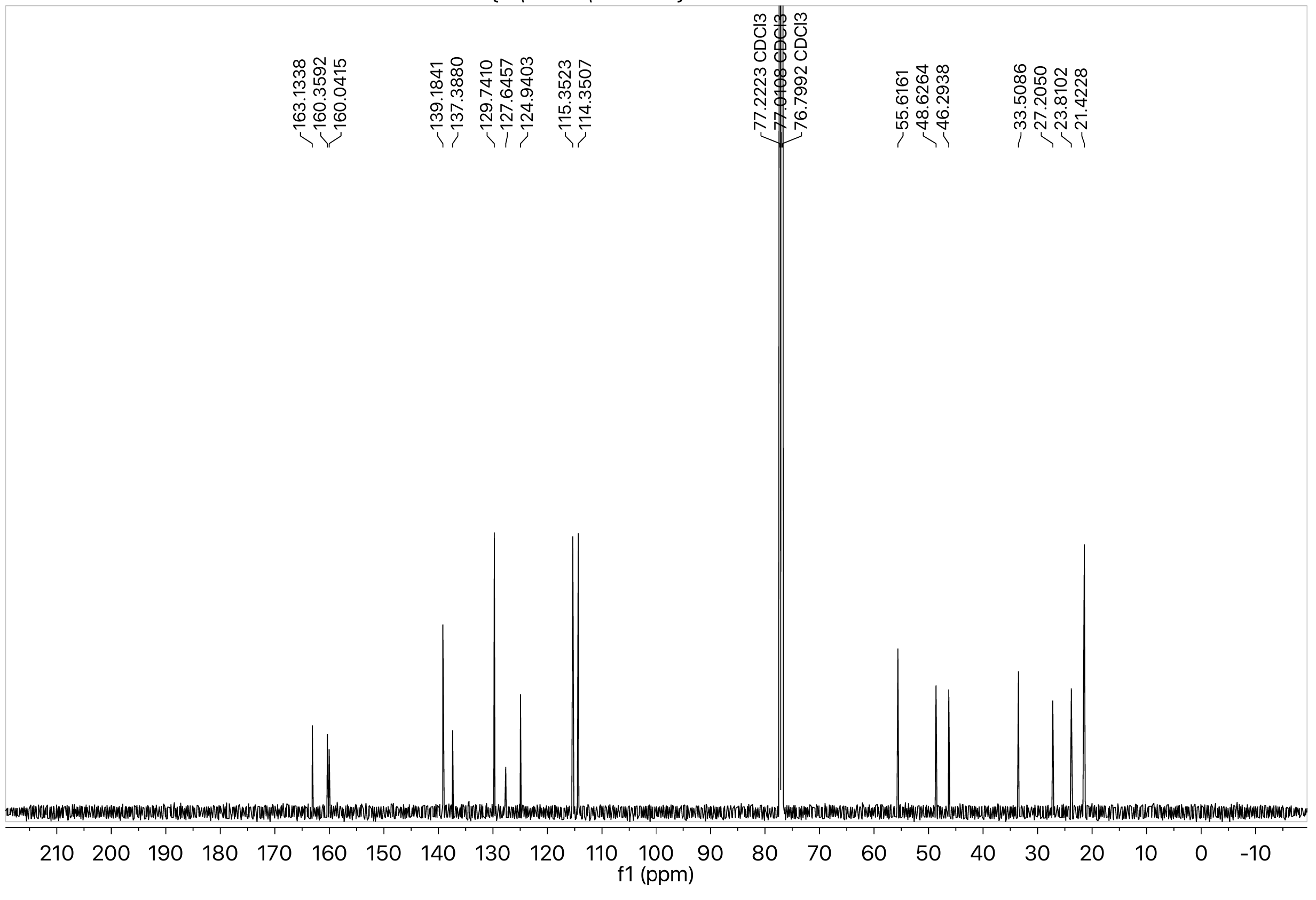


**Figure S10: 13C-NMR spectrum of compound 7e**


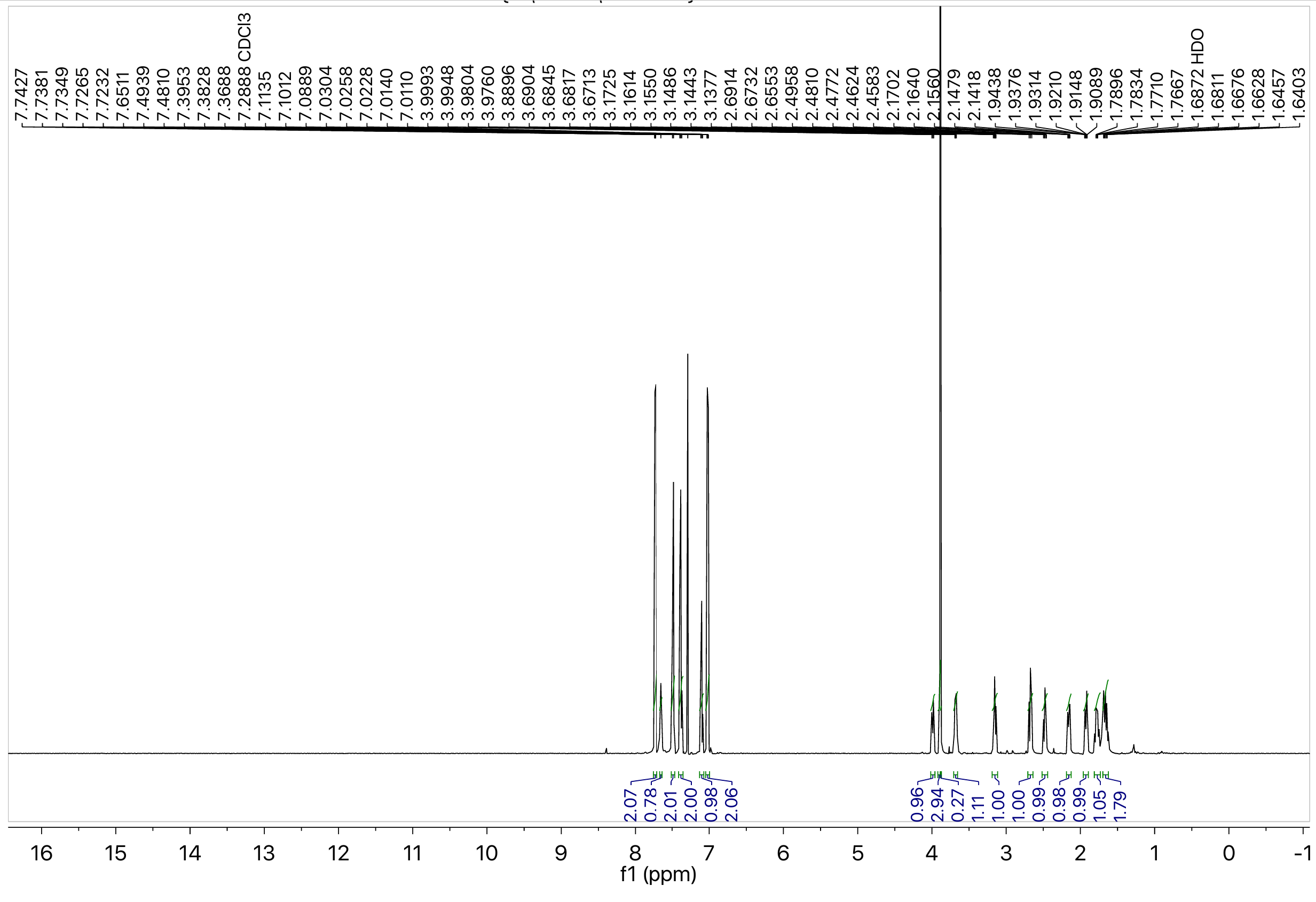


**Figure S11: 1H-NMR spectrum of compound 7f**


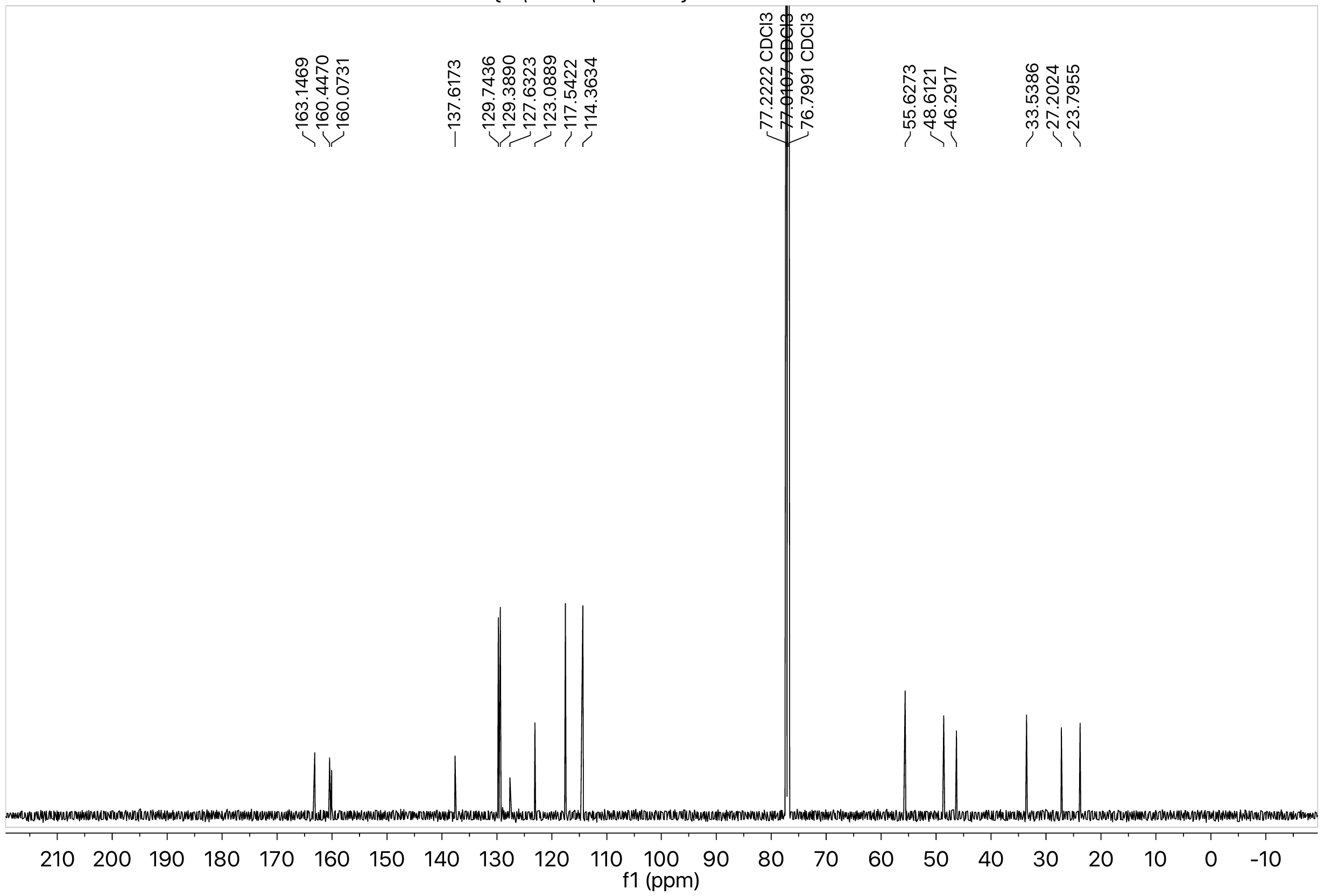


**Figure S12: 13C-NMR spectrum of compound 7f**


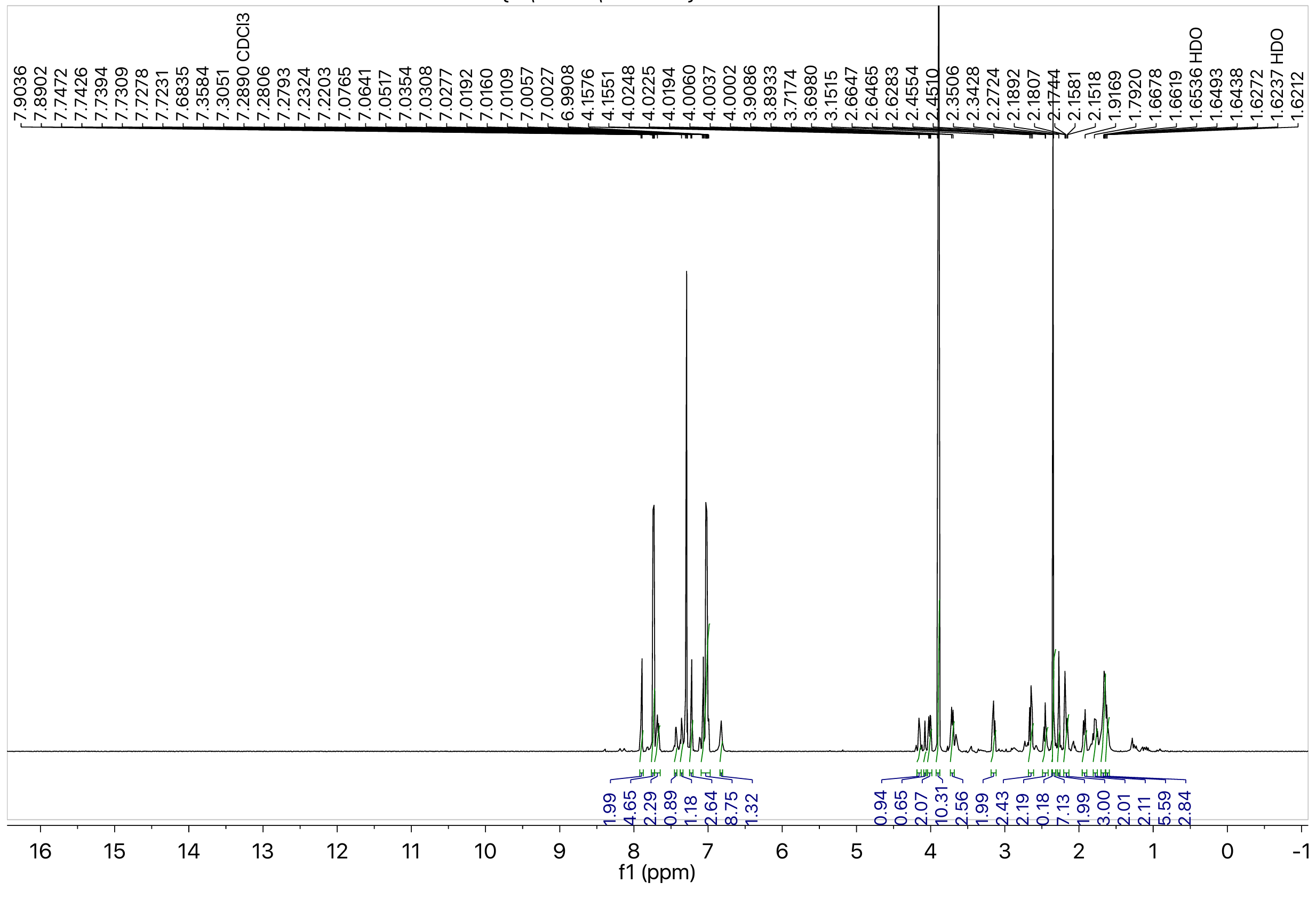


**Figure S13: 1H-NMR spectrum of compound 7g**


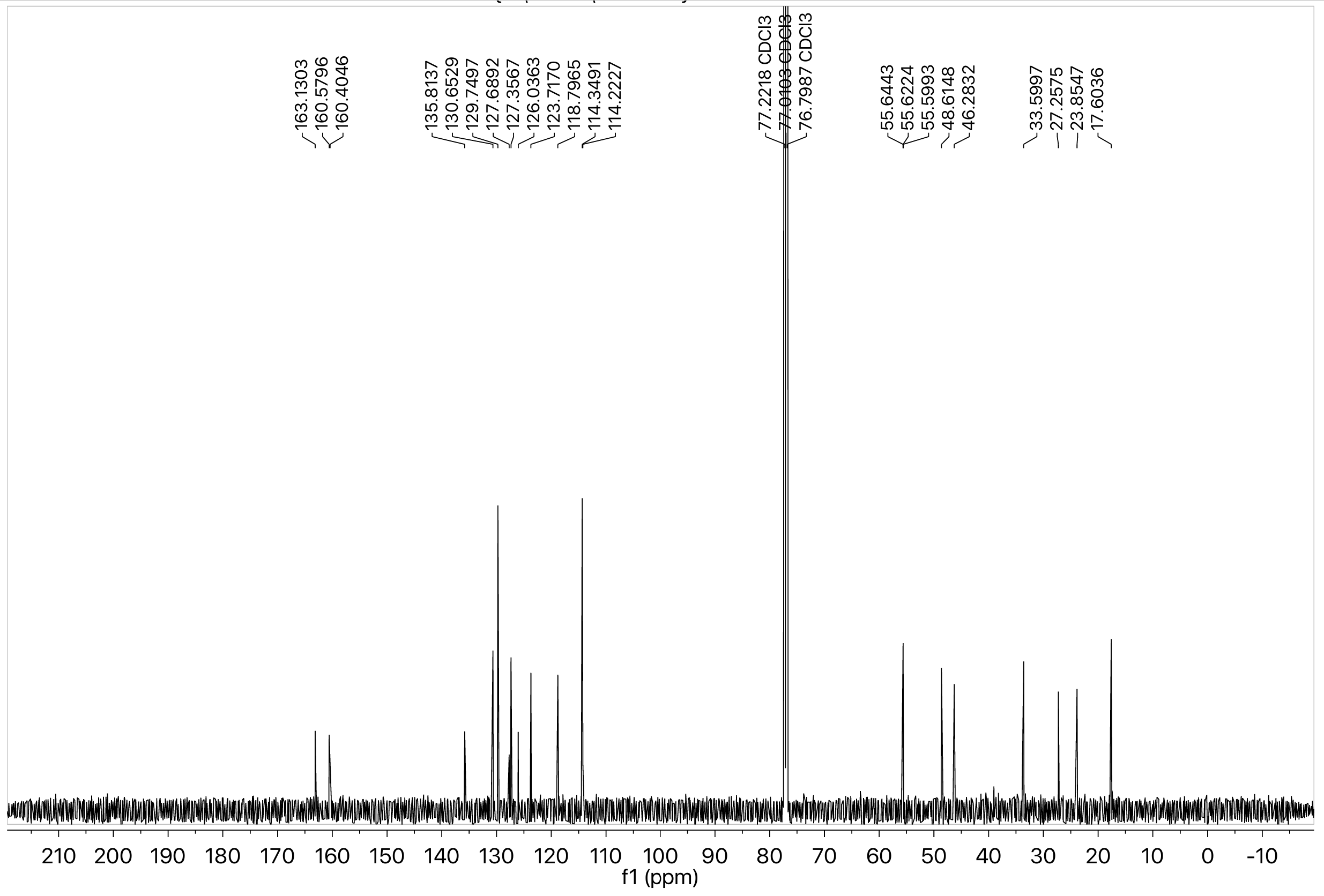


**Figure S14: 13C-NMR spectrum of compound 7g**


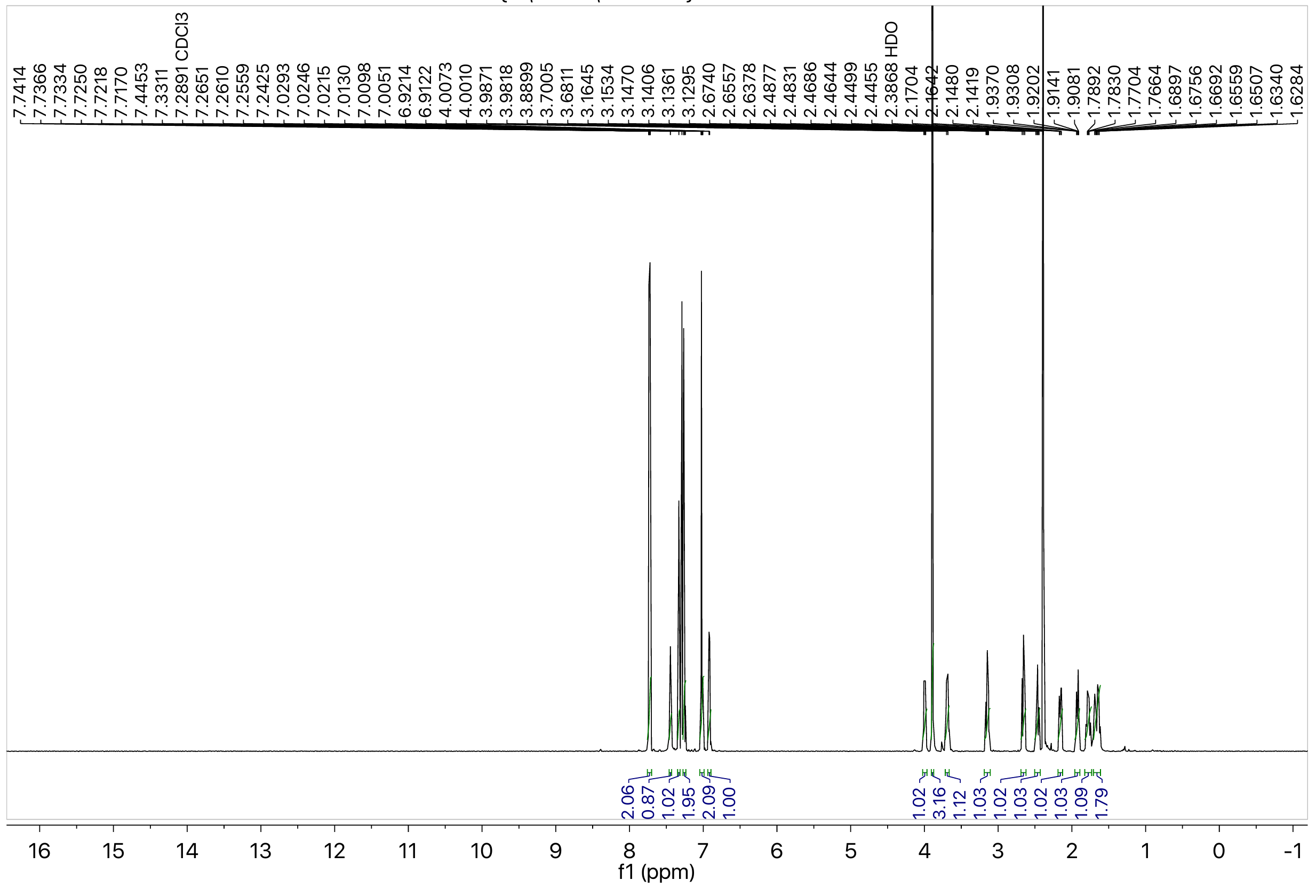


**Figure S15: 1H-NMR spectrum of compound 7h**


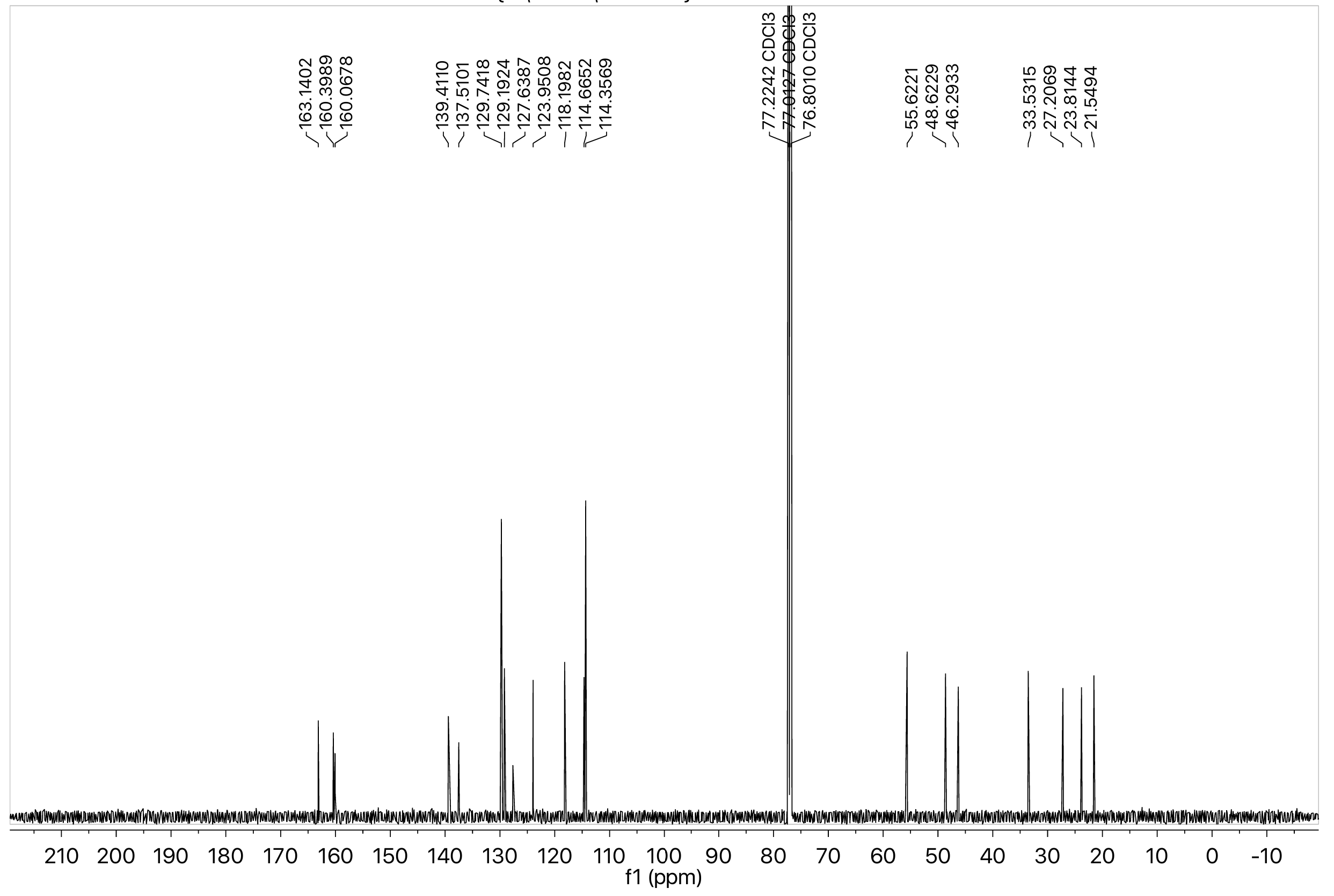


**Figure S16: 13C-NMR spectrum of compound 7h**


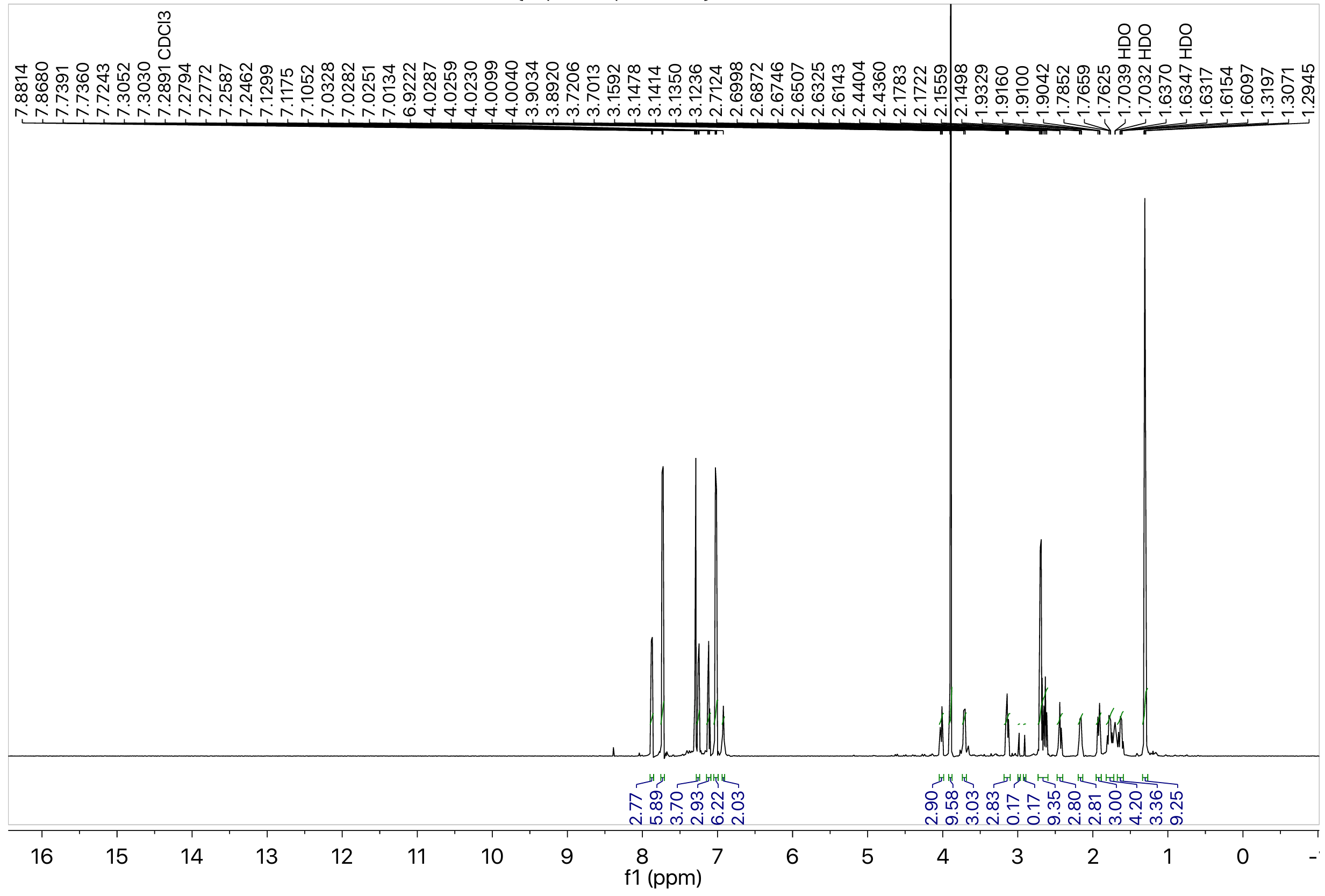


**Figure S17: 1H-NMR spectrum of compound 7i**


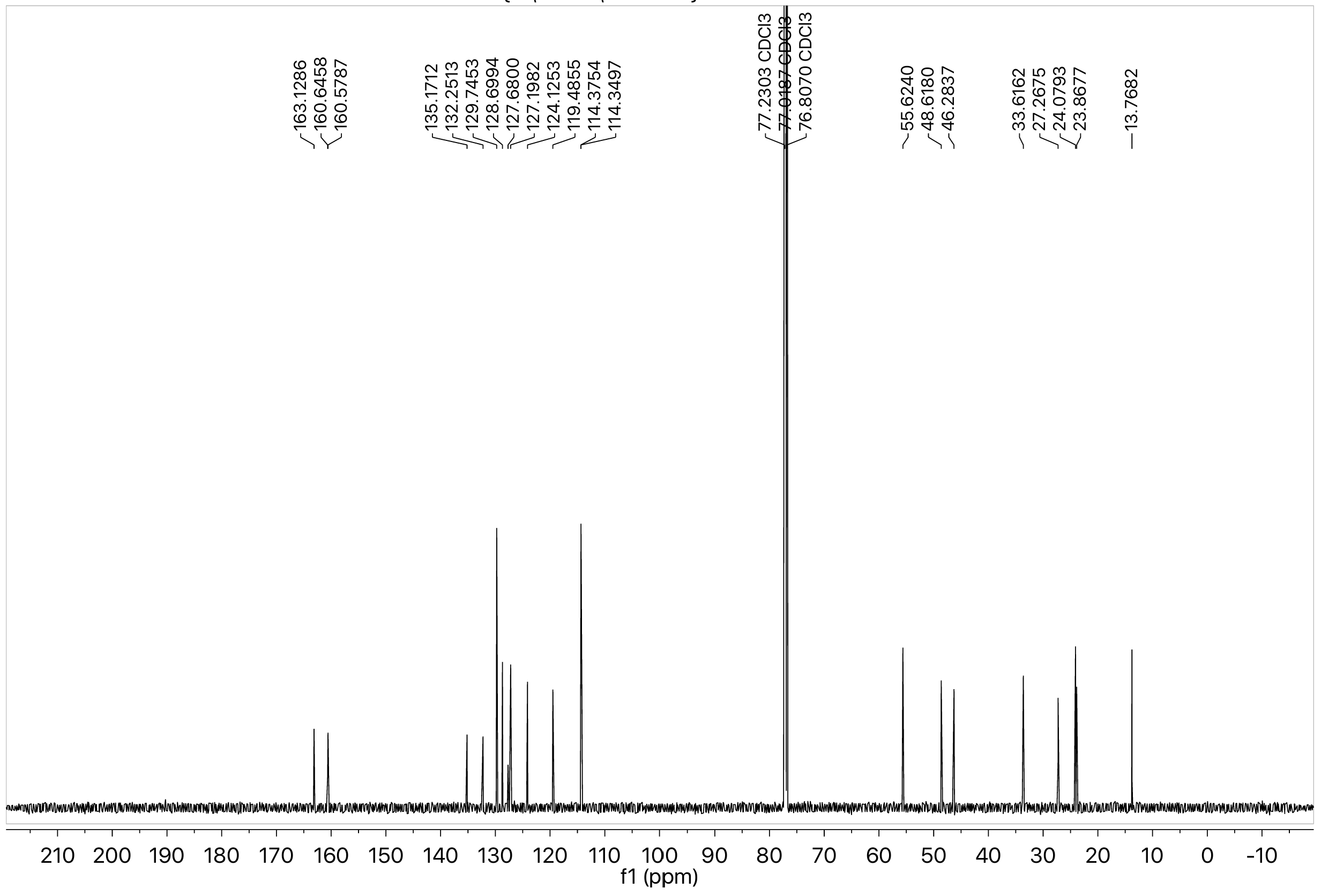


**Figure S18: 13C-NMR spectrum of compound 7i**


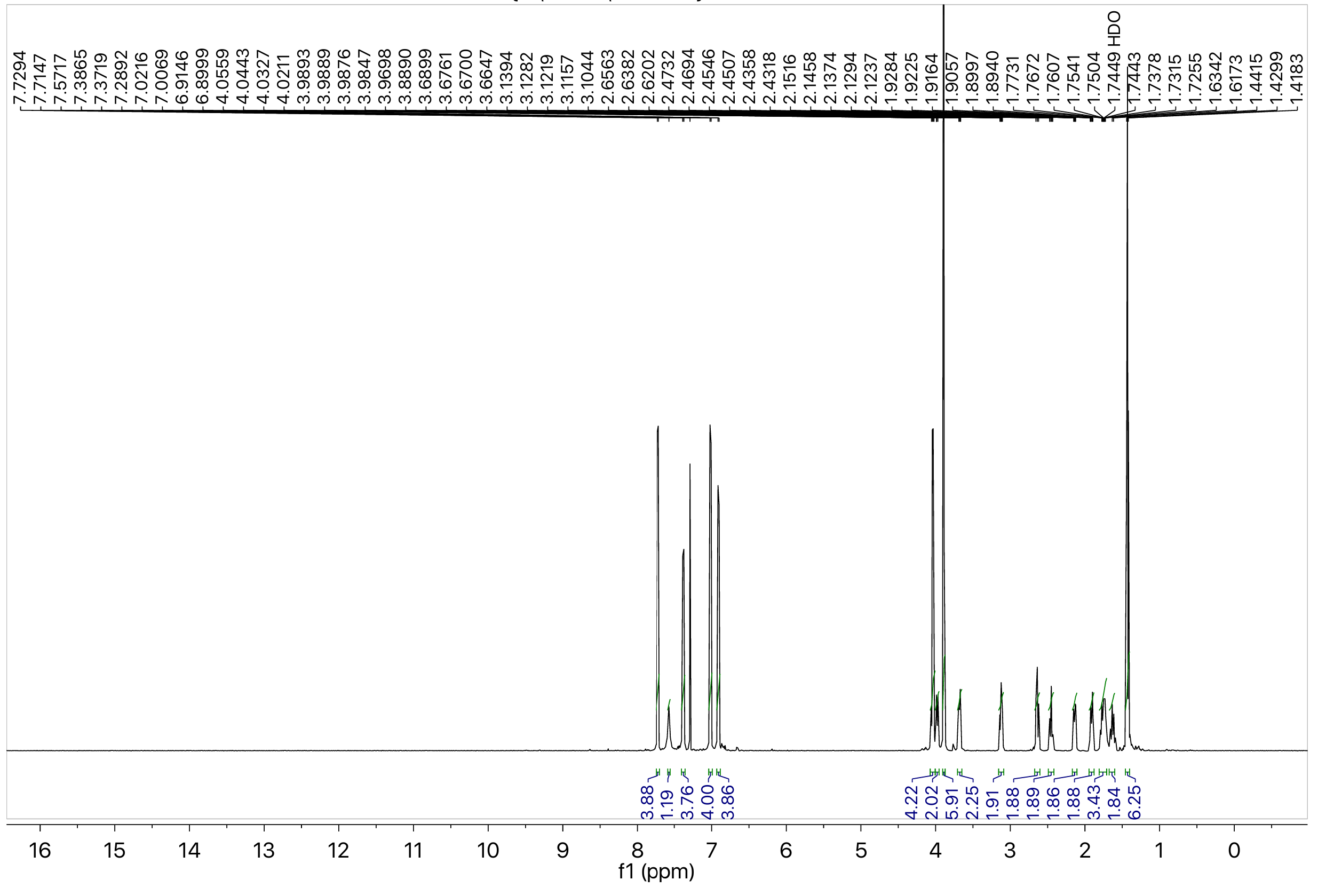


**Figure S19: 1H-NMR spectrum of compound 7j**


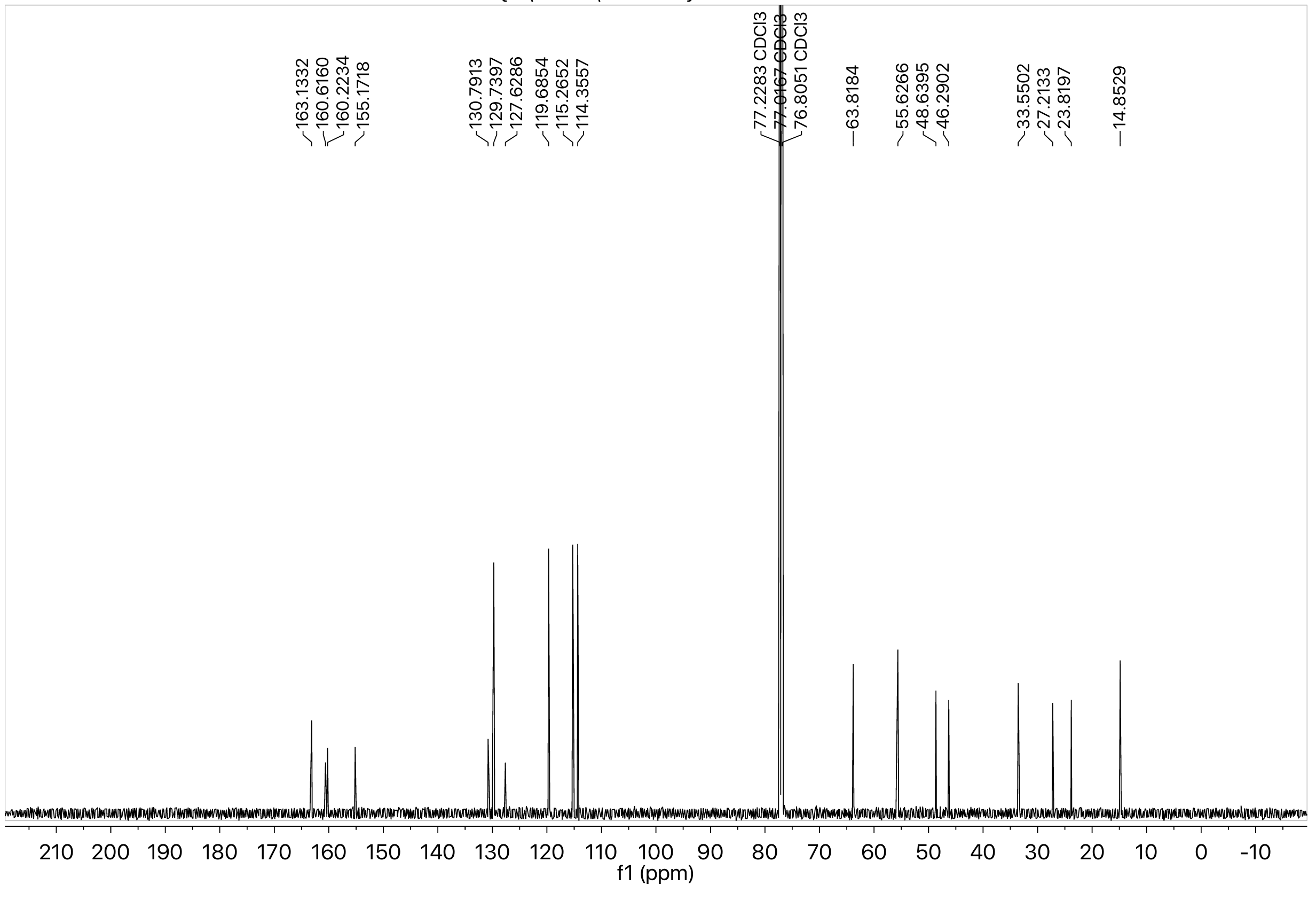


**Figure S20: 13C-NMR spectrum of compound 7j**


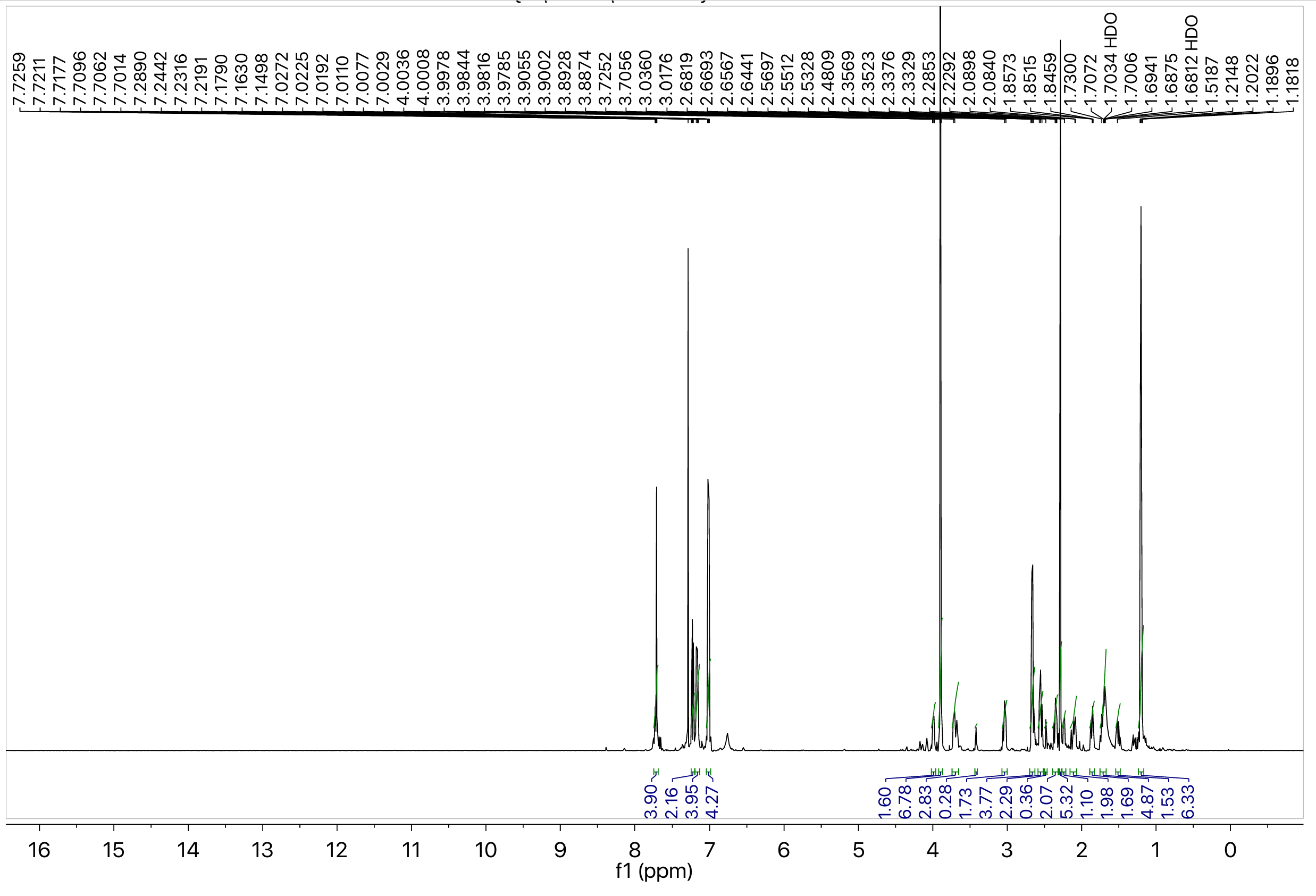


**Figure S21: 1H-NMR spectrum of compound 7k**


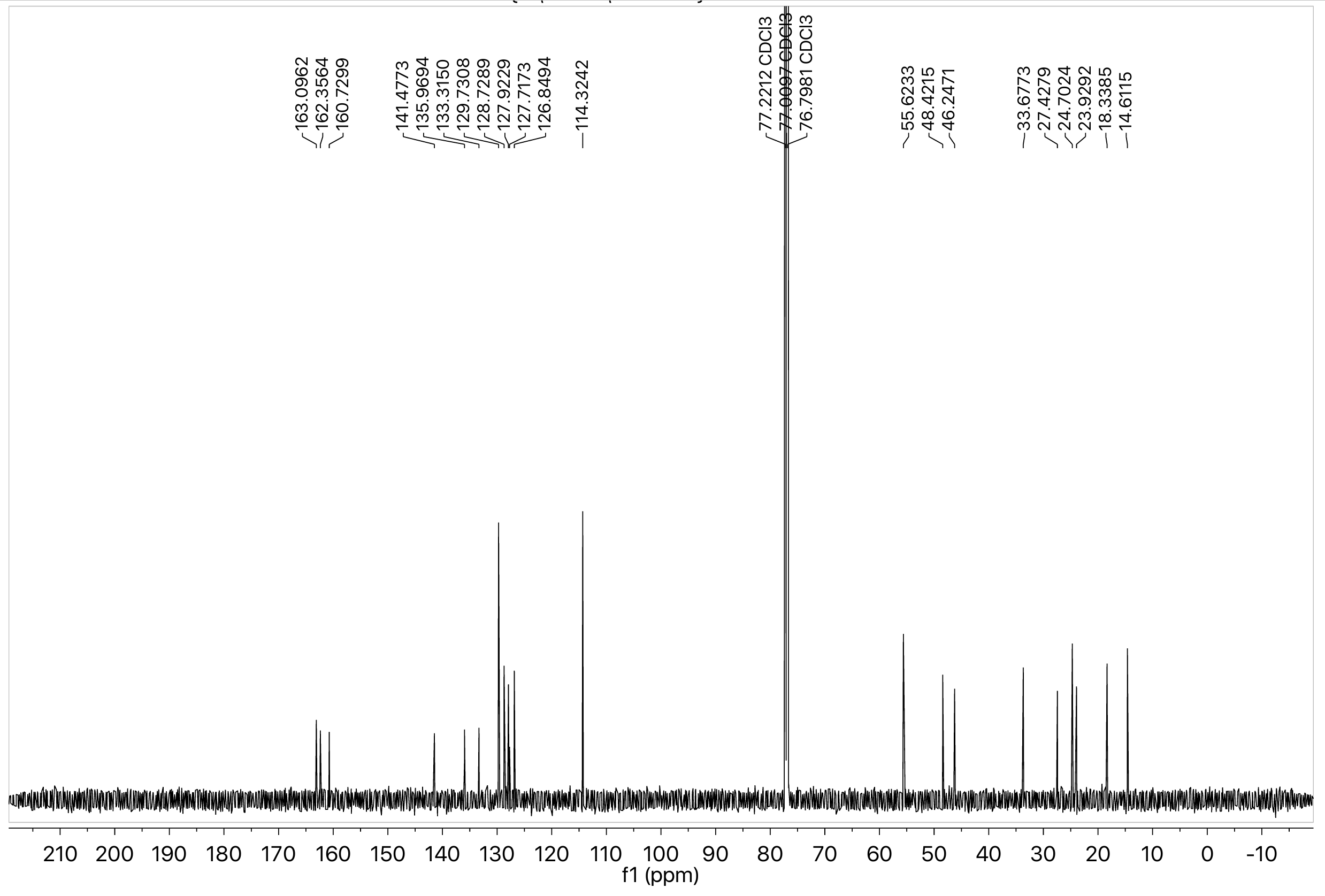


**Figure S22: 13C-NMR spectrum of compound 7k**


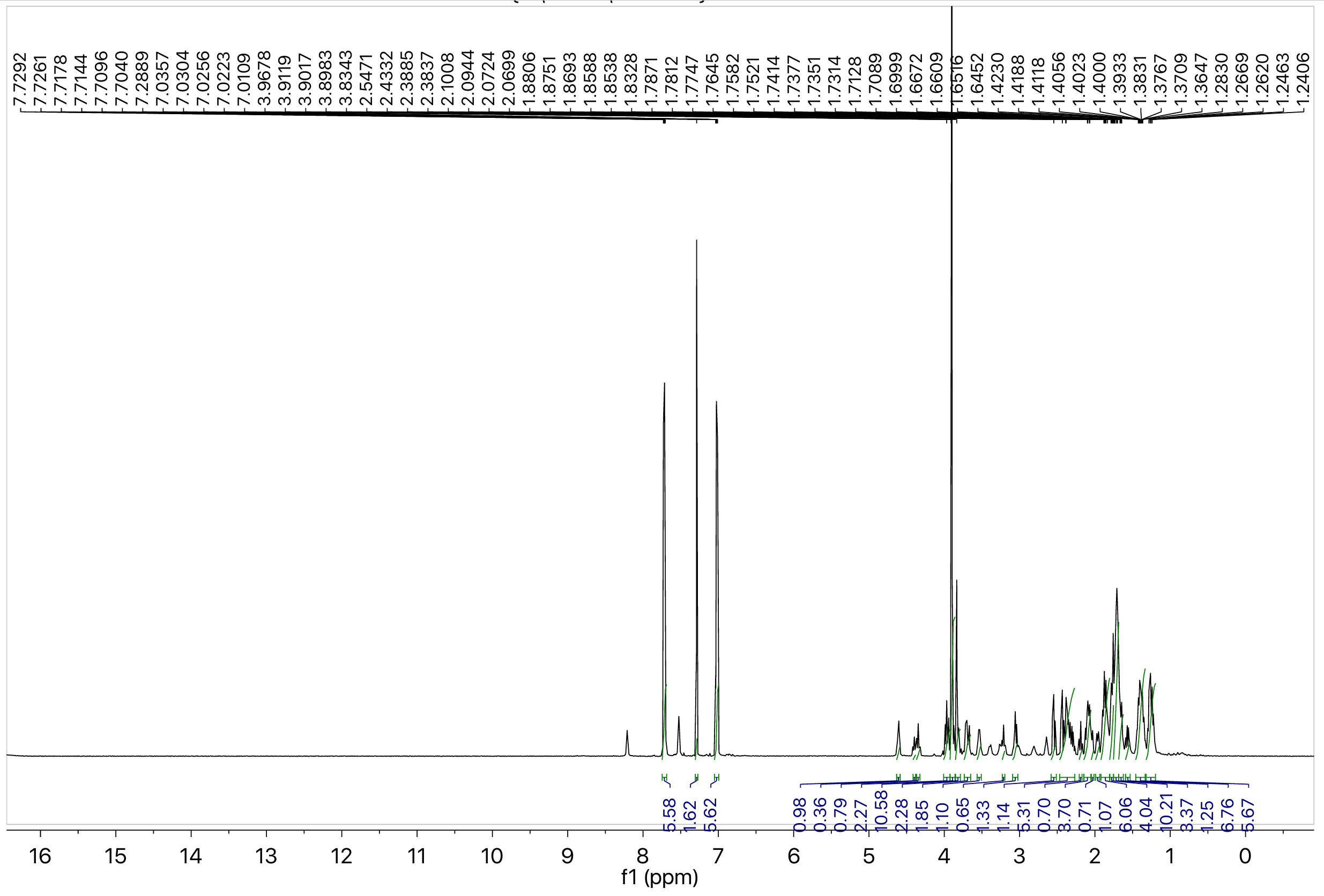


**Figure S23: 1H-NMR spectrum of compound 7l**


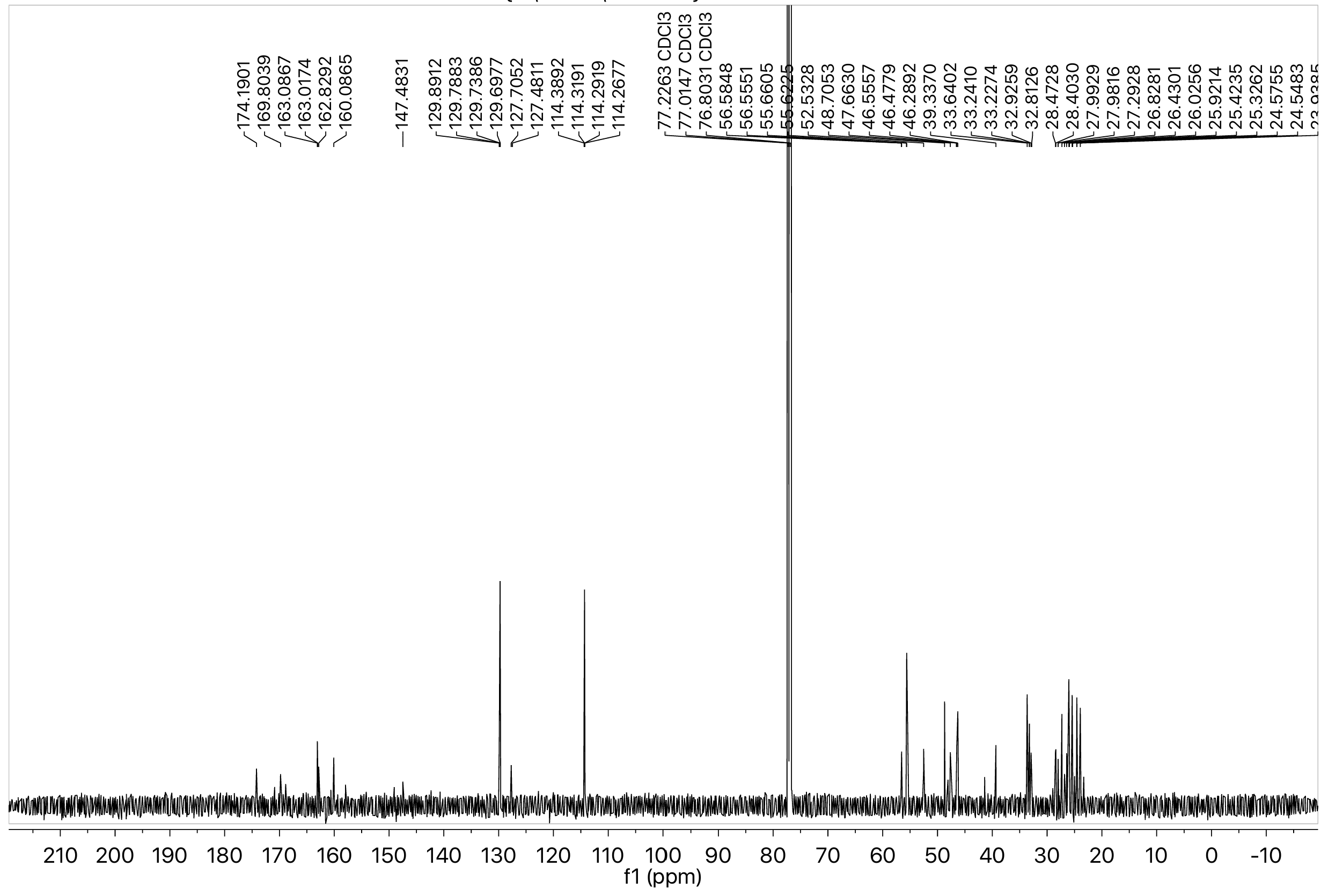


**Figure S24: 13C-NMR spectrum of compound 7l**


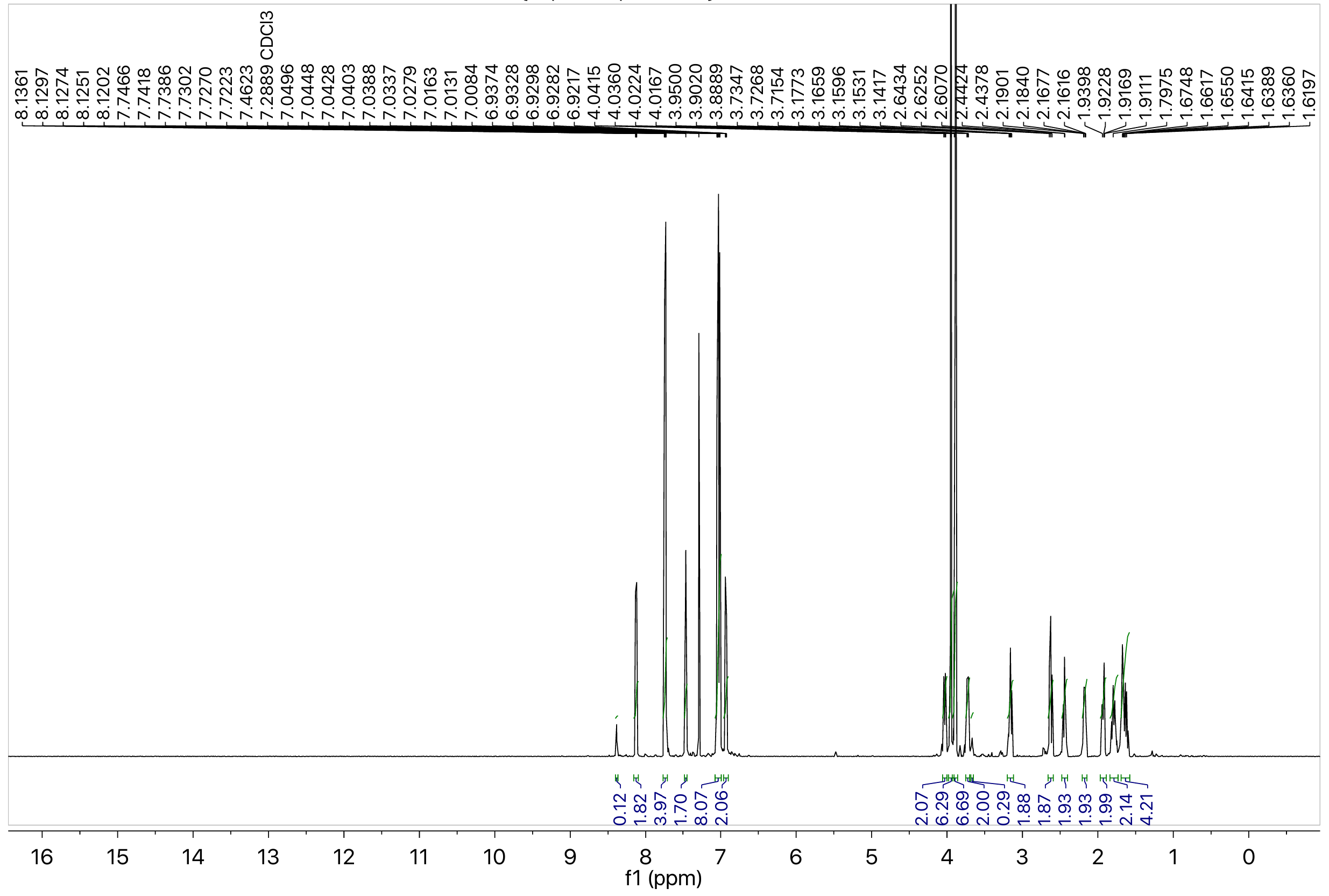


**Figure S25: 1H-NMR spectrum of compound 7m**


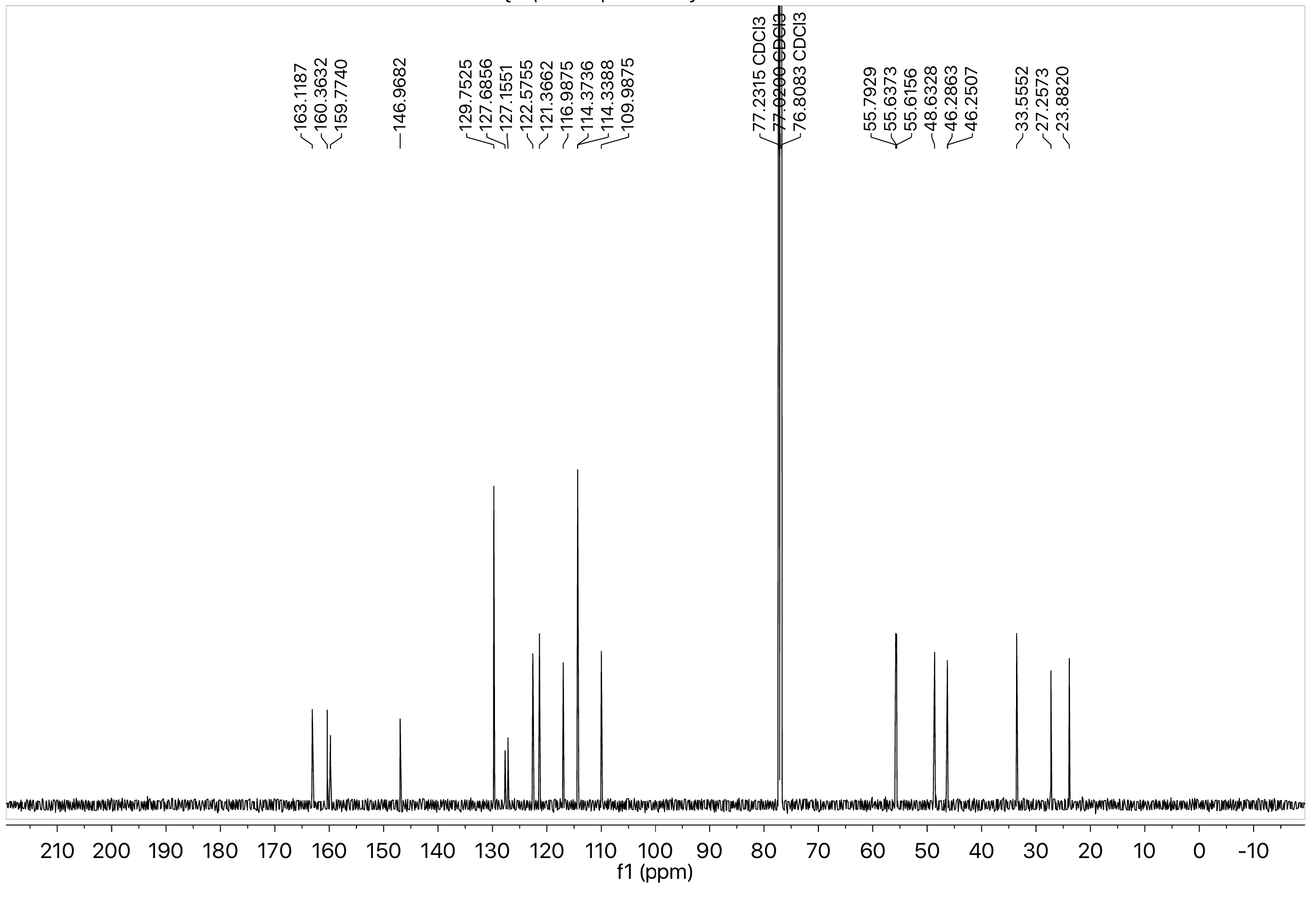


**Figure S26: 13C-NMR spectrum of compound 7m**


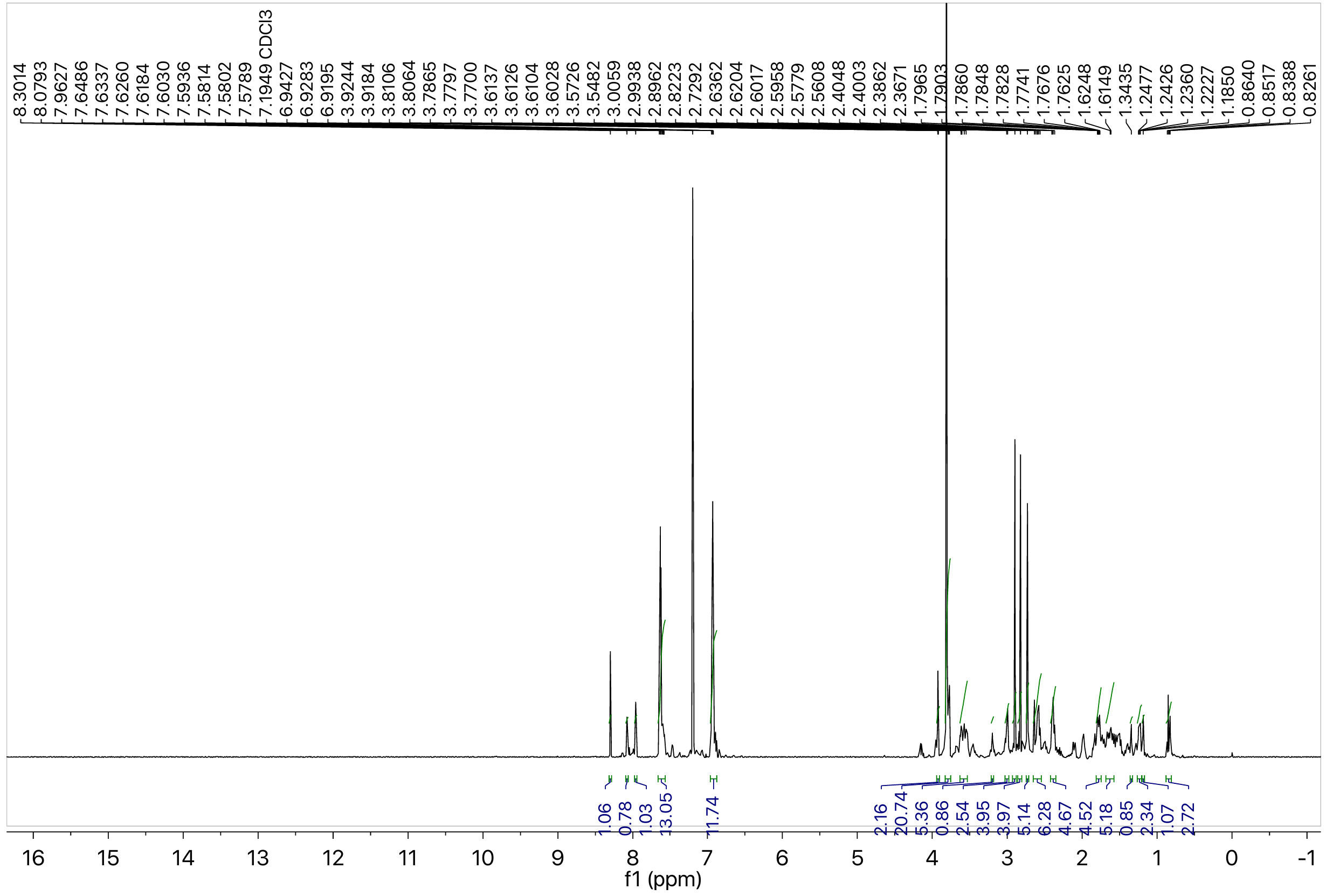


**Figure S27: 1H-NMR spectrum of compound 7n**


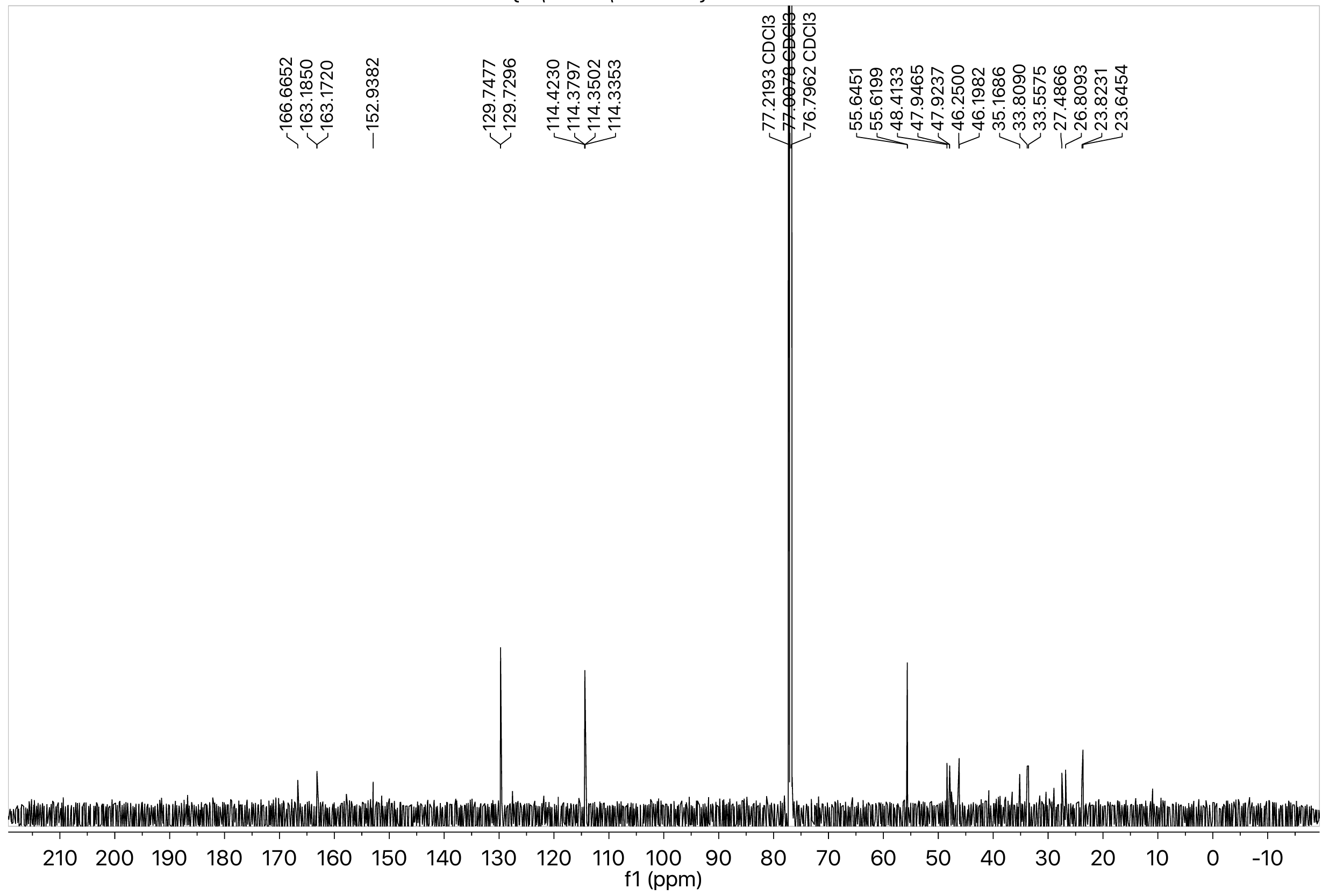


**Figure S28: 13C-NMR spectrum of compound 7n**


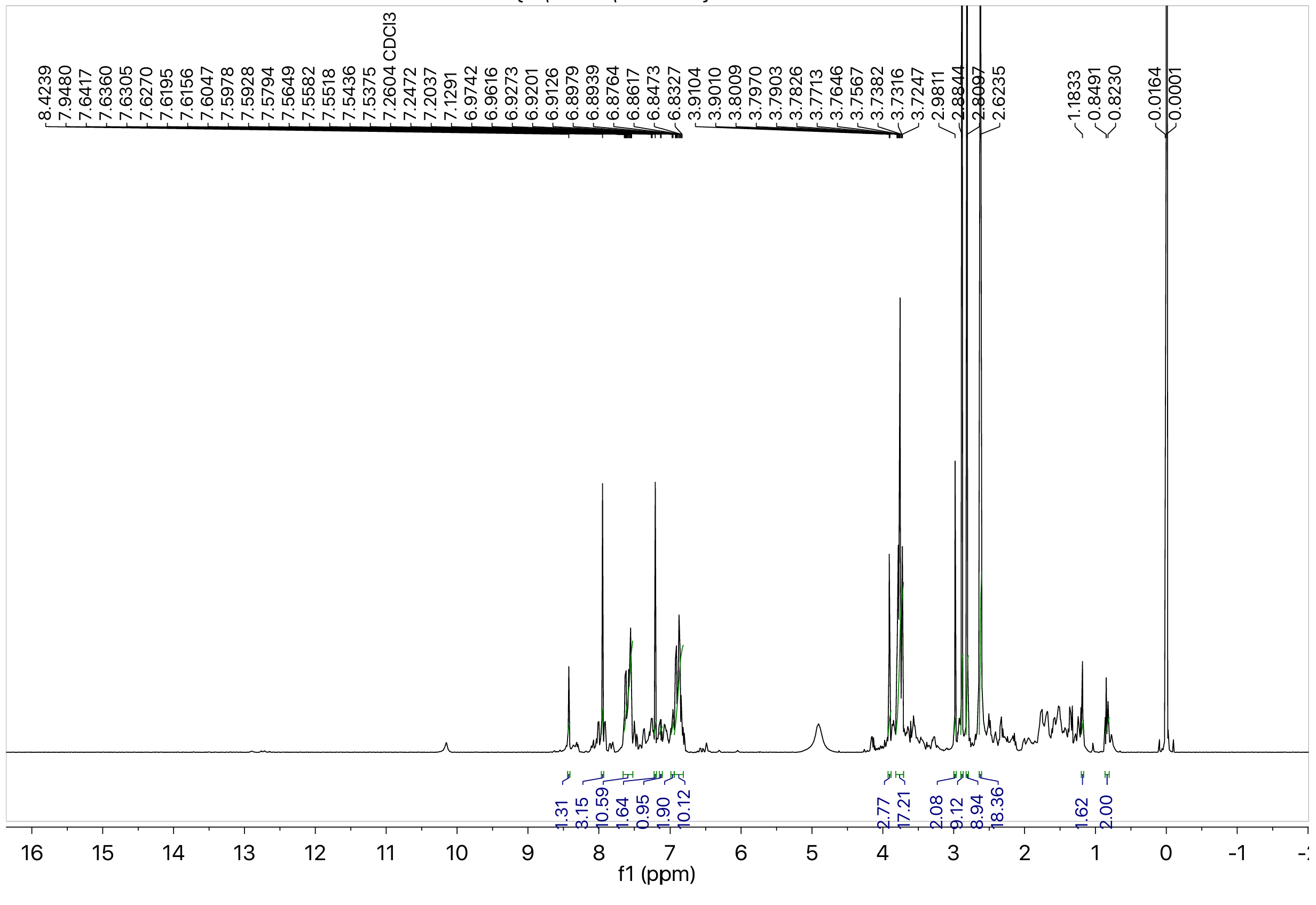


**Figure S29: 1H-NMR spectrum of compound 7o**


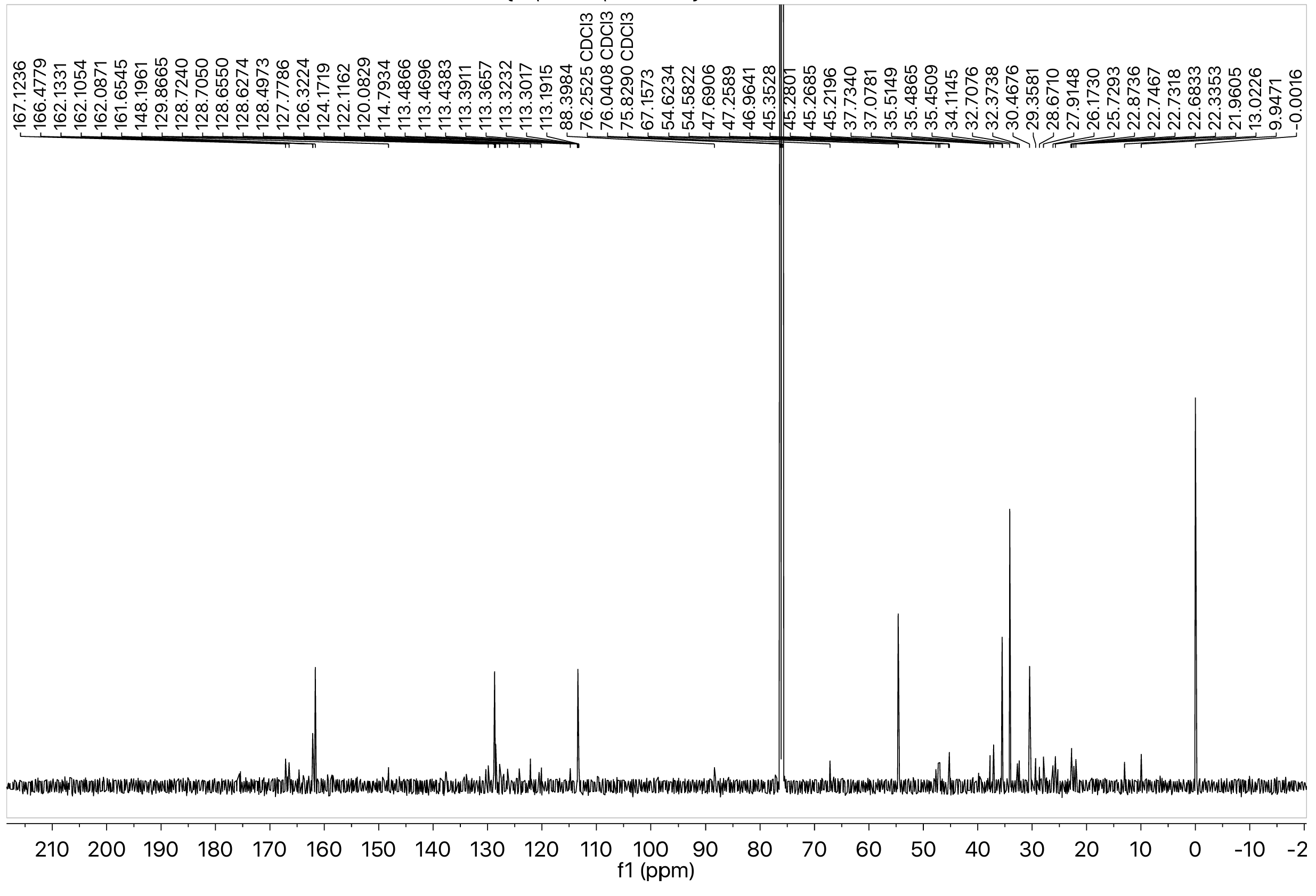


**Figure S30: 13C-NMR spectrum of compound 7o**

**EI-MS SPECTRA OF ALL COMPOUNDS**

**Figure S31: EI-MS Spectra of (7a)**

**Figure S32: EI-MS Spectra of (7b)**

**Figure S33: EI-MS Spectra of (7c)**

**Figure S34: EI-MS Spectra of (7d)**

**Figure S35: EI-MS Spectra of (7e)**

**Figure S36: EI-MS Spectra of (7f)**

**Figure S37: EI-MS Spectra of (7g)**

**Figure S38: EI-MS Spectra of (7h)**

**Figure S39: EI-MS Spectra of (7i)**

**Figure S40: EI-MS Spectra of (7j)**

**Figure S41: EI-MS Spectra of (7k)**

**Figure S42: EI-MS Spectra of (7l)**

**Figure S43: EI-MS Spectra of (7m)**

**Figure S44: EI-MS Spectra of (7n)**

**Figure S45: EI-MS Spectra of (7o)**

**DOCKING STUDIES OF ALL COMPOUNDS AGAINST LIPOXYGENASE**

Compound **7a** was docked against lipoxygenase with the PDB ID: 3V99. Their interactions are shown in Figure S46. Their interaction involves hydrogen bond and hydrophobic. Conventional hydrogen bond interaction observed with GLN363 and THR364 at the bond distance of 3.36Å and 3.25 Å. Carbon hydrogen bond revealed with ILE673 at bond distance of 3.45 Å. Pi-sigma interaction observed with LEU607 at the bond distance of 3.62 Å. Pi-pi T shaped interaction observed with HIS432 at the bond distance of 4.71 Å. Many alkyl interactions were observed with ALA419, LEU368, LEU607, LEU368 and LEU373 at the bond distance of 4.34 Å, 5.25 Å, 4.33 Å, 4.24 Å and 4.64 Å. Pi-alkyl interactions observed with HIS367, HIS372, HIS372, HIS432, LEU368, LEU368, LEU373, ALA410, and ARG411 at the bond distance of 4.59 Å, 4.54 Å, 4.79 Å, 4.97 Å, 5.31 Å, 4.67 Å, 4.88 Å, 5.26 Å and 5.23 Å. Its binding affinity is -8.4kcal/mol.


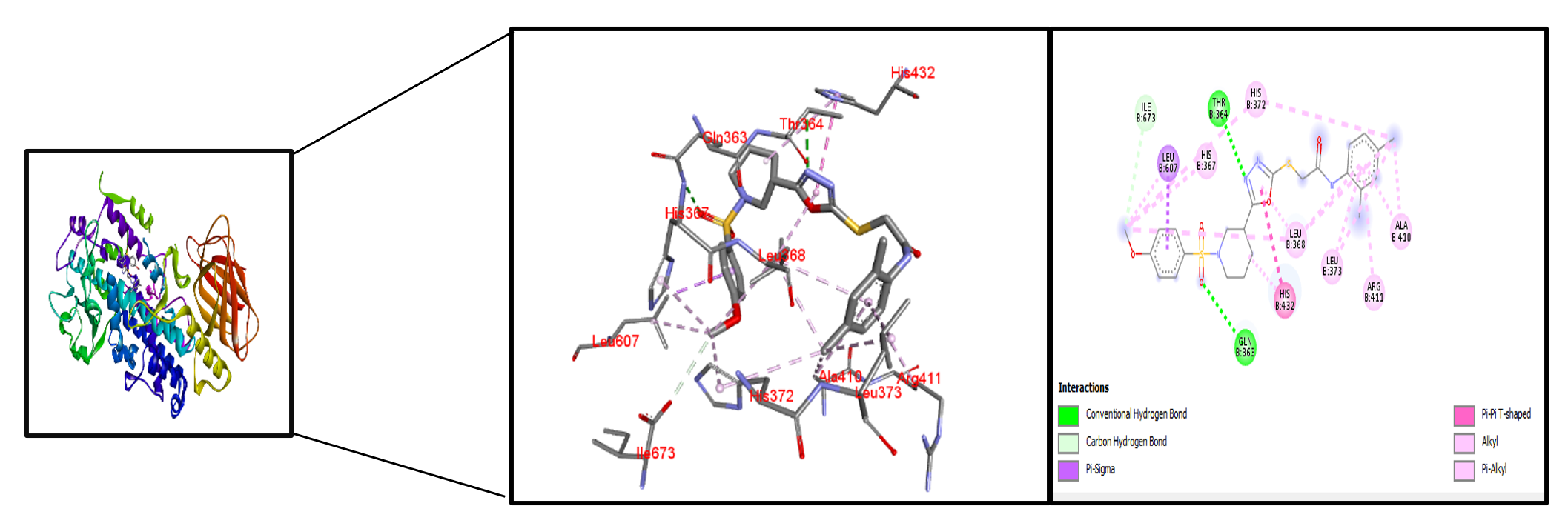


**Figure S46: Docking generated complexes of compound** **7a against lipoxygenase**

Compound **7b** docked against lipoxygenase with the PDB ID: 3V99. Their interaction is shown in Figure S47. Their interaction involves hydrogen bond and hydrophobic. Conventional hydrogen bond interaction observed with THR364 at the bond distance of 3.25 Å. Carbon hydrogen bond revealed with ILE673 at bond distance of 3.43 Å. Pi-sigma interaction observed with LEU607 at the bond distance of 3.70 Å. Pi-pi stacked interaction observed with HIS432 at the bond distance of 4.22 Å. Alkyl interactions were observed with LEU607, LEU368 and LEU373 at the bond distance of 4.63 Å, 3.96 Å and 4.34 Å. Pi-alkyl interactions observed with HIS372, HIS432, LEU368, LEU368, LEU368, ALA410, and ARG411 at the bond distance of 5.12 Å, 4.41 Å, 5.05 Å, 5.11 Å, 5.01 Å and 4.89 Å. Its binding affinity is -8.1 kcal/mol.


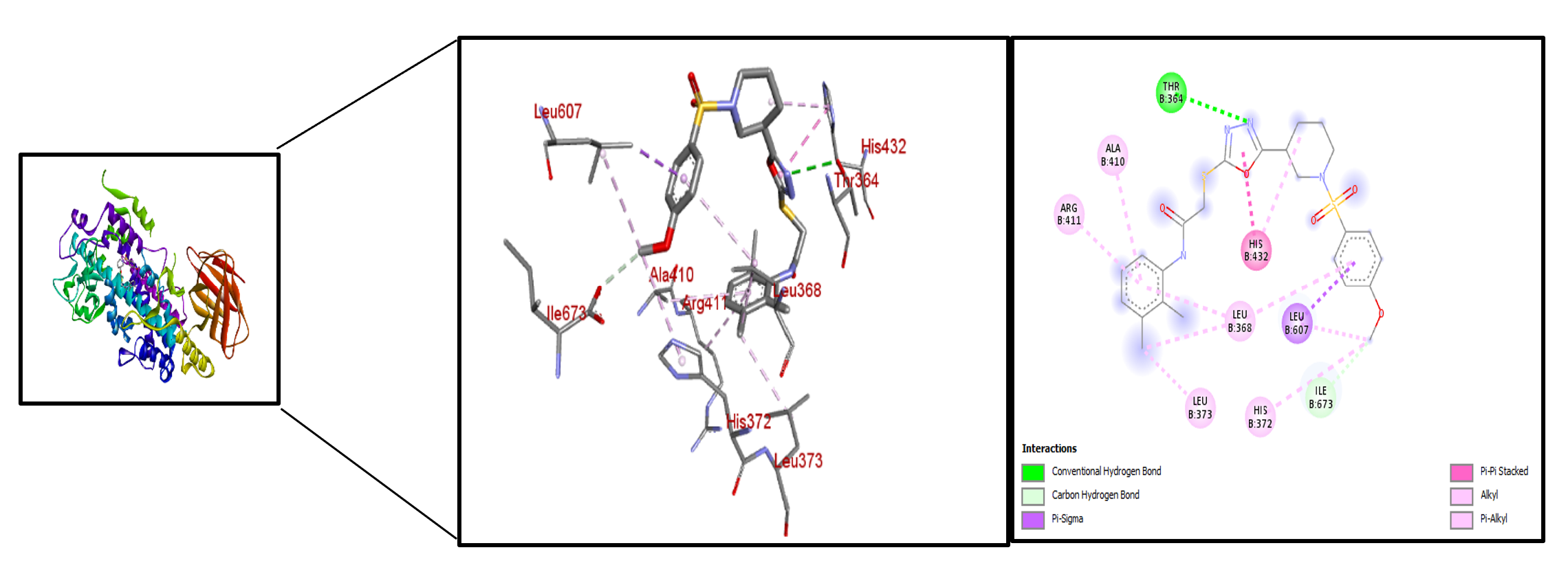


**Figure S47: Docking generated complexes of compound** **7b against lipoxygenase**

Compound **7c** docked against lipoxygenase with PDB ID 3v99. Their interactions are shown in Figure S48. Their interactions involve electrostatic and hydrophobic. A Pi-Cation interaction observed with ARG596 at a bond distance of 4.90 Aͦ. At a bond distance of 3.77Aͦ, Pi-Pi Stacked interactions observed with HIS432. The four Alkyl interactions revealed with CYS300, ARG572, LEU368 and LEU373 at 4.15, 4.67, 3.94 and 4.39 Aͦ respectively. The four Pi-Alkyl interactions are observed with ARG596, LEU368, LEU373 and ARG411 at 4.73, 5.31, 5.42 and 5.22 Aͦ respectively. It has binding affinity of -8.2 Kcal/mol.


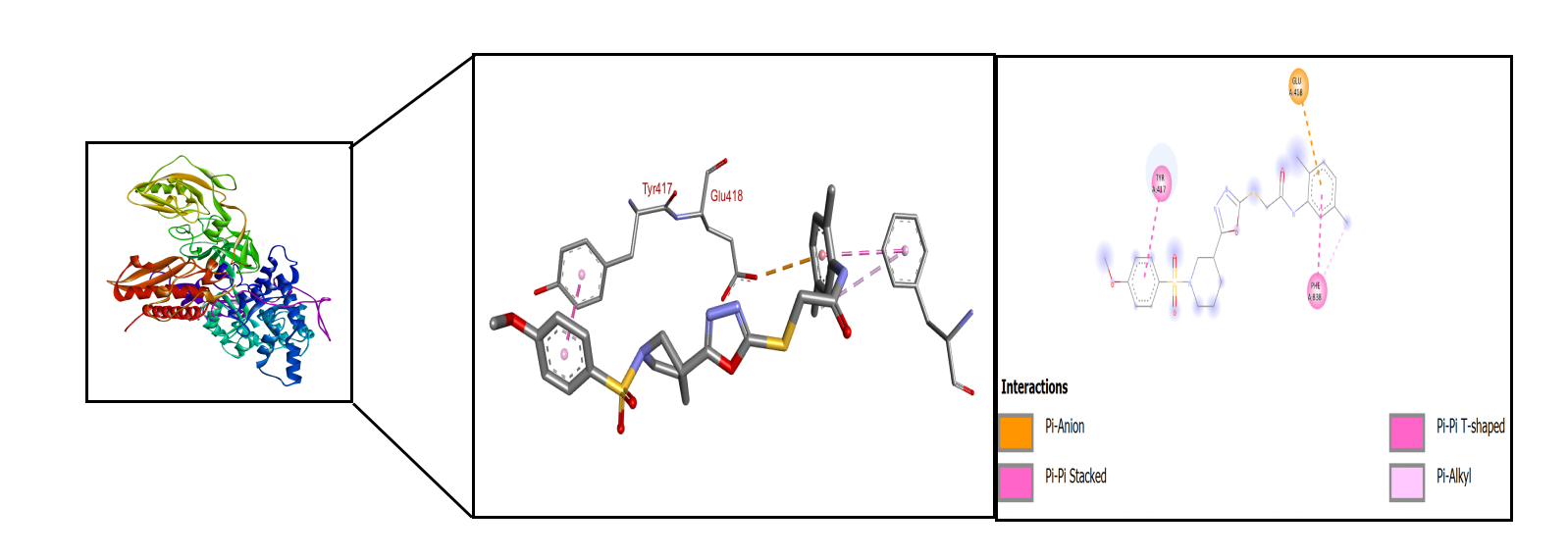


**Figure S48: Docking-generated complexes of compound 7c against lipoxygenase**

The interactions of compound **7d** with lipoxygenase protein are shown in Figure S49. Their docking results revealed the Pi-Donor Hydrogen interactions with GLN611 at distance of 3.59 Aͦ. The two hydrophobic interactions are observed including Pi-Pi Stacked and Alkyl interactions with PHE169 and PRO621 at 3.65 and 4.22 Aͦ respectively. It has binding affinity of -8.0 Kcal/mol.


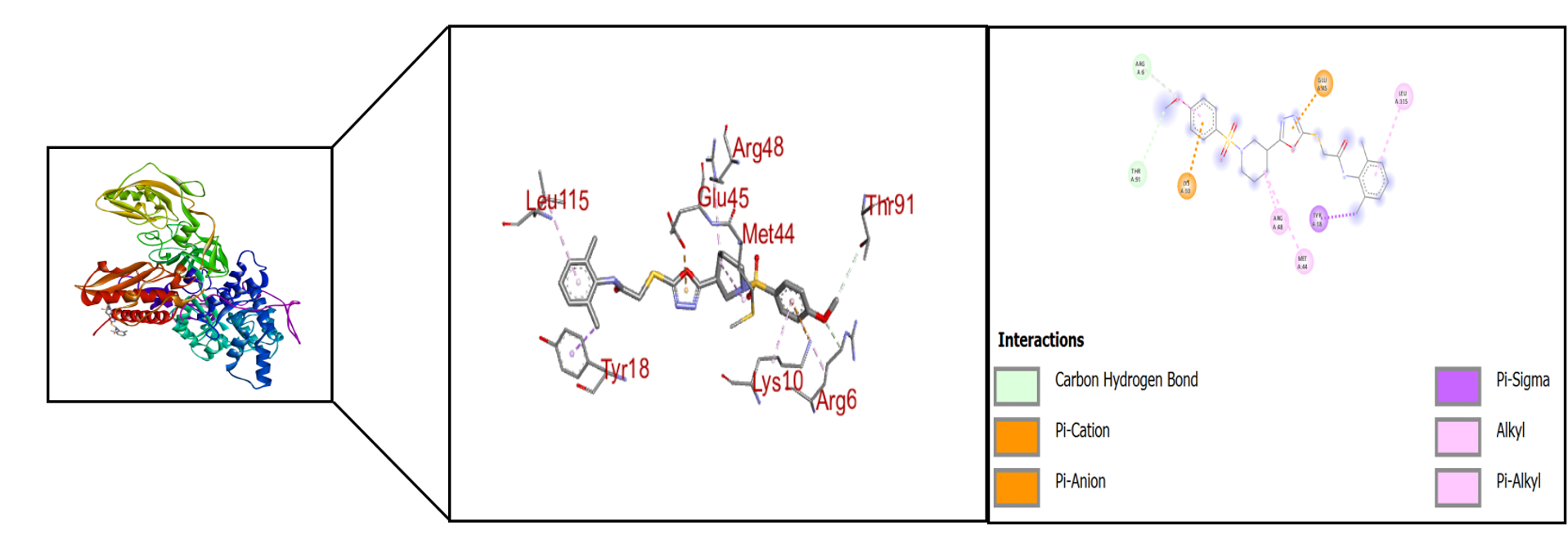


**Figure S49: Docking-generated complexes of compound 7d against lipoxygenase**

Synthesized compounds such as **7e** showed maximum lipoxygenase inhibition activity so they are docked with lipoxygenase confirm different interaction and binding capabilities making it more potent against lipoxygenase (Figure S50). These bindings of **7e** were confirmed with lipoxygenase protein with (PDB ID 3v99). The docking results revealed key interactions stabilizing the ligand within the binding site. Two Hydrogen Bonds were Observed with (UNKO: N24) and ARG411 at a bond distance of 3.39 Å and 3.69 Å respectively. A π–π interaction with LEU607 and a π–π stacking interaction with HIS432 were Observed at 3.58 Å and 4.95 Å respectively indicate aromatic stabilization. Many hydrophobic alkyl interactions present with ALA410, ILE409, VAL408, LEU409, and LEU607 at the bond distance 3.67 Å, 4.40 Å, 4.58 Å, 4.67 Å, 4.64 Å respectively. A π–alkyl interaction was observed with PHE151, HIS372, LEU409, and ALA410 at distances ranging from 4.10 to 5.22 Å, indicating a strong hydrophobic environment that supports ligand binding. It shows binding affinity of -8.1 Kcal/mol.


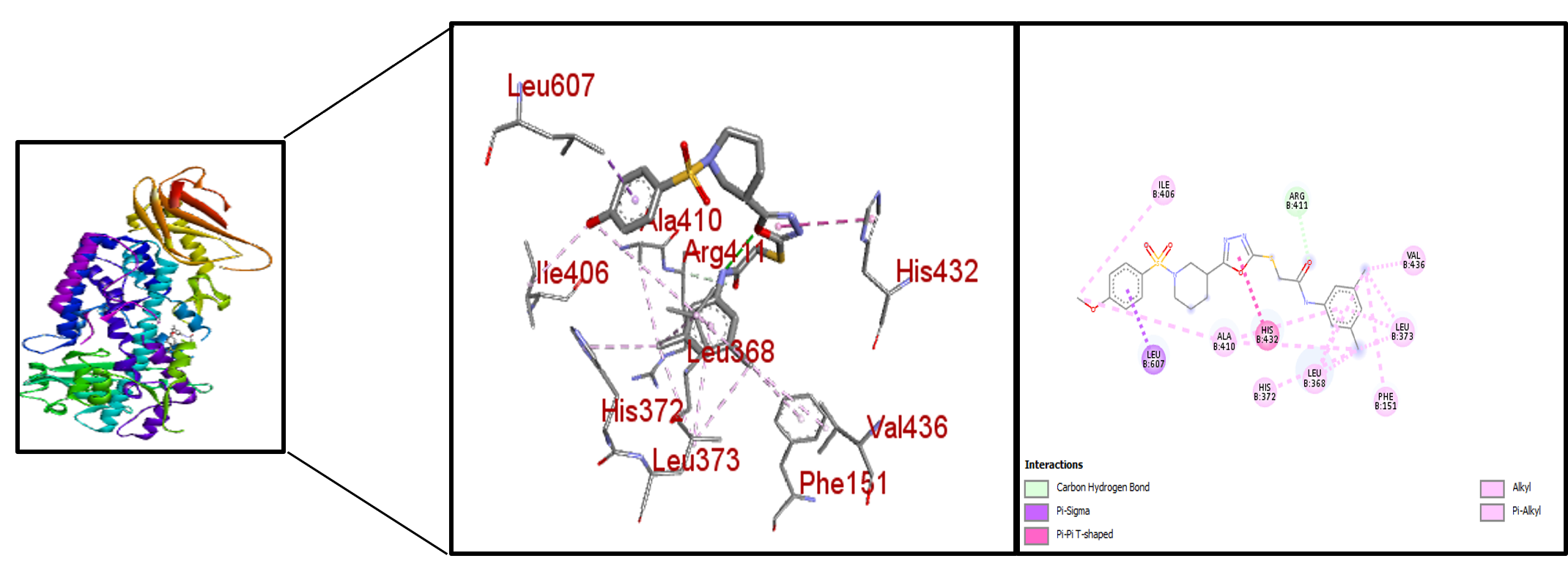


**Figure S50: Docking-generated complexes of compound 7e against lipoxygenase**

Compound **7f** showed minimum lipoxygenase inhibition activity so it is also docked with same protein of (PDB ID 3v99) Figure S51. Two hydrogen bonds were observed, with SER412 and ASP411 at the bond distances of 3.17 Å and 3.05 Å respectively stabilizing the complex through conventional and carbon-hydrogen bonding. A π–π interaction was observed between the ligand and HIS432 with 4.93 Å. A π–alkyl interaction was also observed with HIS372 at a bond distance of 5.16 Å. These interactions collectively create a strong binding network that reinforces ligand affinity and enhances molecular stability within the active site. It shows binding affinity of -6.1 Kcal/mol.


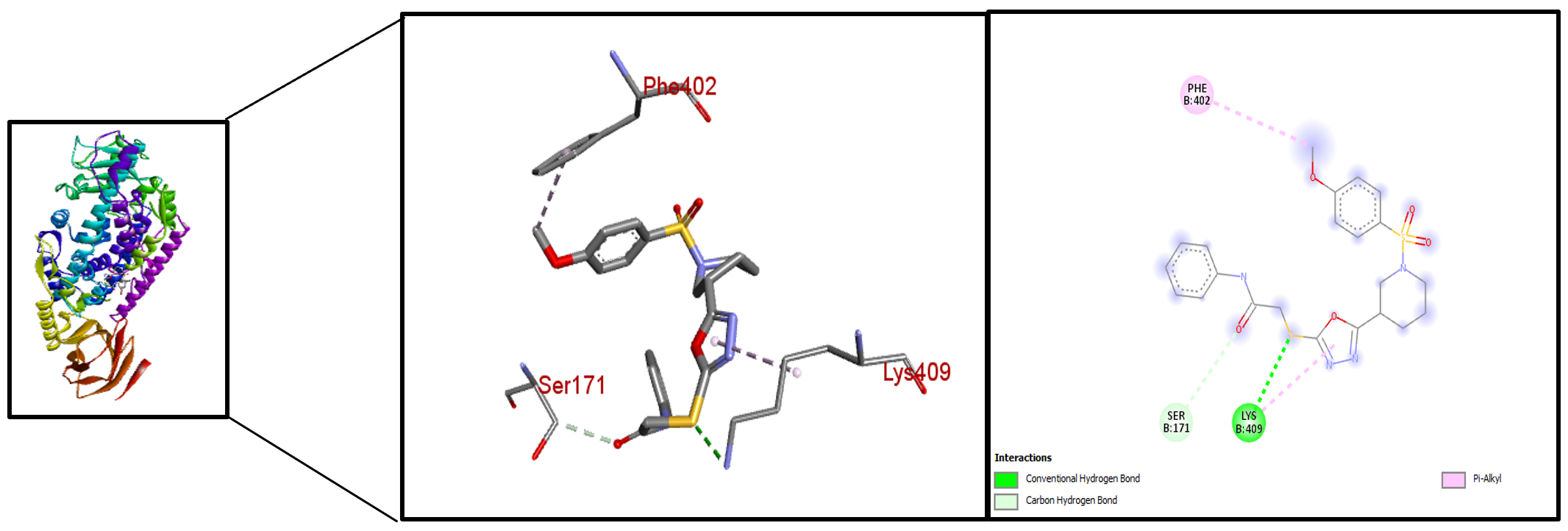


**Figure S51: Docking-generated complexes of compound** **7f against lipoxygenase**

In the molecular docking study, the ligand **7g** binds to the protein's active site through a combination of hydrogen bonding, electrostatic, and hydrophobic interactions. Specifically, the ligand forms two conventional hydrogen bonds with SER670, involving the ligand's acceptor atoms and the donor hydrogen from SER670, at distances of 3.18 Å and 3.22 Å, with angles of 160.5° and 136.3°, respectively. An electrostatic Pi-Anion interaction is also present between the negatively charged GLU614 and the ligand's pi-orbitals at 4.09 Å. Furthermore, the ligand engages in multiple hydrophobic interactions, including a Pi-Sigma interaction with LEU615 at 3.28 Å, two Pi-Pi T-shaped interactions with PHE169 at 4.88 Å and 5.62 Å, alkyl interactions with LEU615, LEU438, and PRO621 at distances ranging from 3.37 Å to 4.49 Å, and Pi-Alkyl interactions with LEU438, ALA672, and ILE406 at distances between 4.84 Å and 5.16 Å. Its binding affinity was -8.2 kcal/mol.


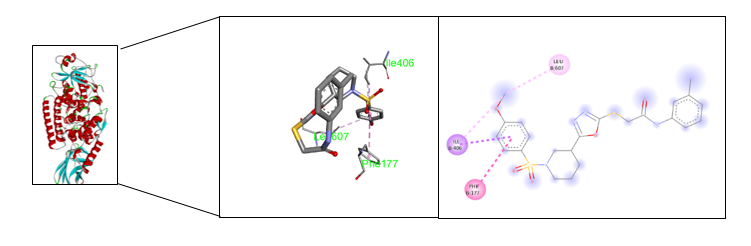


**Figure S52: Docking-generated complexes of compound** **7g against lipoxygenase**

Among the synthesized Compound **7i** exhibited the lipoxygenase (LOX) inhibitory activity. Their interactions are shown in Figure 1. One Carbon Hydrogen bond interaction observed with HIS432 at a bond distance 3.71 Å. A Pi-Cation interaction observed with ARG596 at a bond distance of 4.36 Å. One Pi-Hydrogen Bond shows interaction with ARG596 at bond distance 3.53 Å. At a bond distance of 4.39 Å, Pi-Pi T-shaped interactions observed with HIS432. The three Alkyl interactions are observed with CYS300, LEU373 and ARG411 at 4.63 Å, 4.41 Å, and 4.50 Å respectively. The seven Pi-Alkyl interactions revealed with TRP147, PHE151, PRO299, LEU373, ALA410, ALG411 and LEU368 at 4.40 Å, 4.94 Å, 5.15 Å, 4.87 Å, 4.90 Å, 4.01 Å, and 4.83 Å respectively. Its binding affinity is -8.0 kcal/mol.


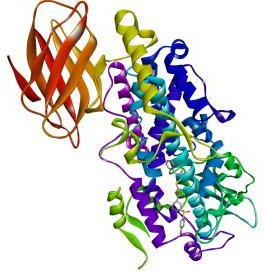

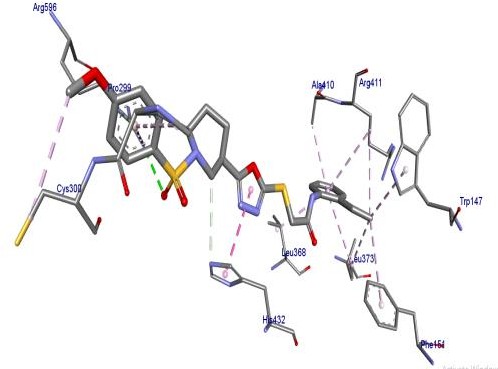

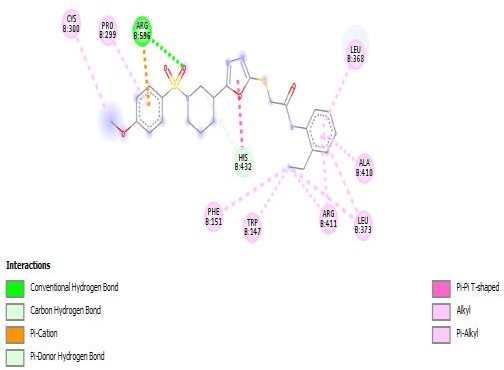


**Figure S53: Docking-generated complexes of compound** **7i against lipoxygenase**

Compound **7j** docking against lipoxygenase protein. The interactions of compound 7J with protein binding site are shown in Figure S55. These interactions involve one Pi-Sigma interaction LEU368 at a bond distance of 3.86 Å. The multiple hydrophobic interactions including Pi-Pi Stacked, Pi sulfur and Pi-Alkyl with PHE177, TYR181 and ALA603 at 5.90 Å, 4.04 Å and 5.38 Å are observed. Its binding affinity is -7.9 kcal/mol.


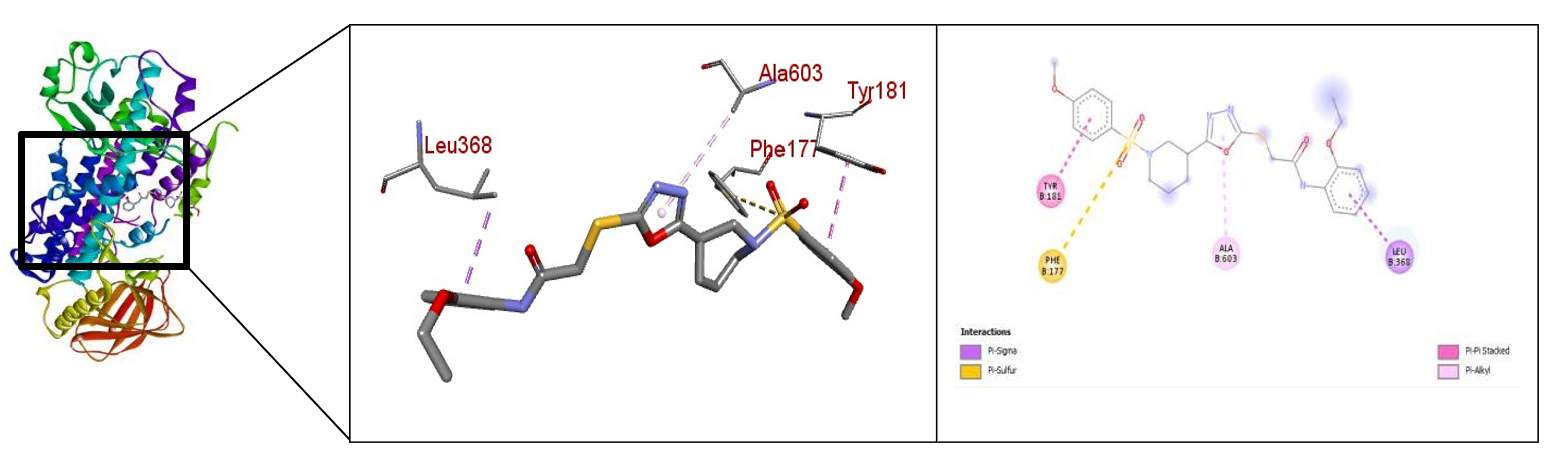


**Figure S55: Docking-generated complexes of compound** **7j against lipoxygenase**

Compound **7k** showed several interactions with 3v99 protein PDB ID in Figure S56. The ligand is represented by a stick model with variously colored atoms, and it interacts with several amino acid residues of the protein, including Asn148, Leu368, Ala410, Ile406, and Leu607. The interactions are represented by dashed lines, indicating potential hydrogen bonds or other non-covalent interactions between the ligand and the protein residues. The distances range from 3.28 to 5.41 Ångströms, indicating a variety of interaction strengths. Notably, the "Types" column categorizes these interactions into "Conventional", "Pi-Sigma", "Alkyl", and "Pi-Alkyl", which correspond to different types of non-covalent interactions. For instance, the conventional hydrogen bond (row 1) has a distance of 3.28 Ångströms, while Pi-Sigma and Pi-Alkyl interactions (rows 2, 3, 6, 7, 8) show distances ranging from 3.66593 to 5.41504 Ångströms, and Alkyl interactions (rows 4, 5) have distances of 4.97376 and 5.33 Ångströms, respectively. This suggests that the nature of the interaction (e.g., hydrogen bonding vs. hydrophobic interactions like Pi-Sigma or Alkyl) influences the distance between the interacting molecules, with hydrogen bonds generally being shorter and thus potentially stronger than the hydrophobic interactions listed.


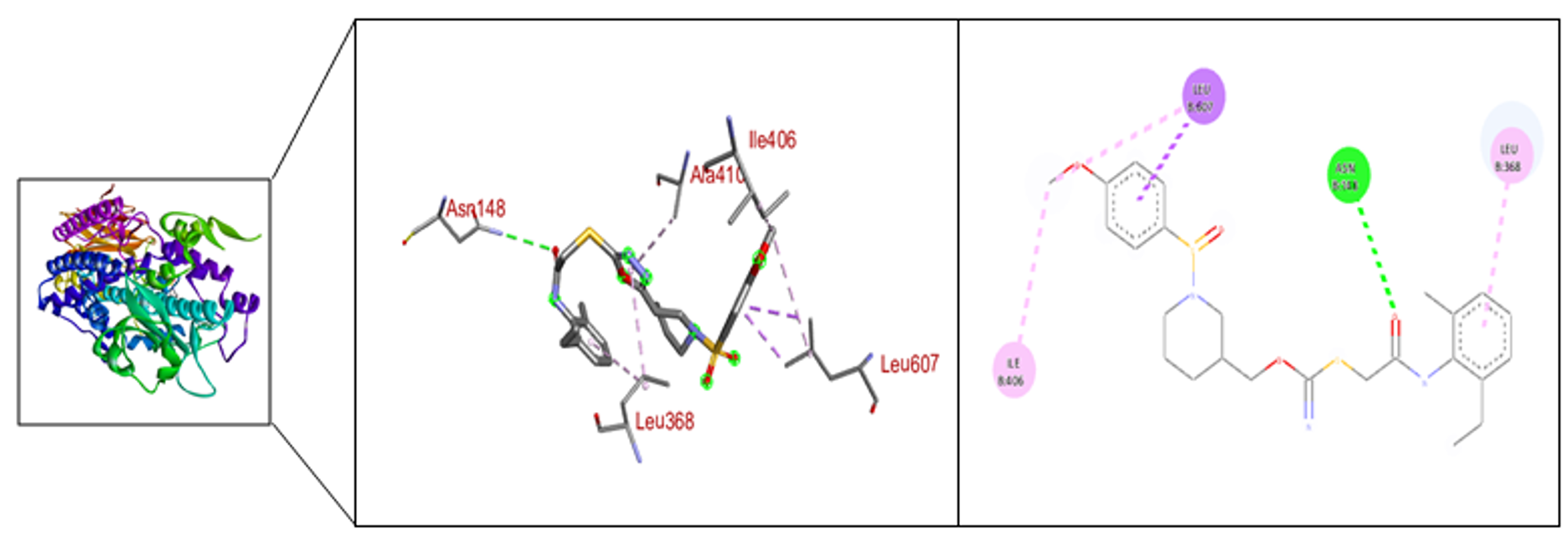


**Figure S54: Docking-generated complexes of compound** **7k against lipoxygenase**

Compound **7l** also dock with 5nn6 protein and their interactions are shown in Figure colours indicating various atoms. Several protein residues (TRP376, LEU678, SER679, GLY651) are labelled, indicating their involvement in the interaction with the ligand and suggesting their significance for understanding the binding mode or affinity. The image highlights various interactions, including hydrogen bonds (green dashed lines) and possibly π-π interactions or other non-covalent interactions (purple dashed lines), crucial for the stability and specificity of the ligand-protein complex. For 'Hydrogen Bond' interactions (rows 1 and 2), the bond distances are 3.38 and 4.11142, respectively. In contrast, 'Hydrophobic' interactions (rows 3-7) exhibit a range of distances from 3.98 to 5.94. Notably, the 'Pi-Alkyl' subtype within 'Hydrophobic' interactions (rows 5-7) displays a relatively narrower range of distances, from 4.21 to 5.34. This suggests that while 'Hydrogen Bond' interactions tend to have shorter distances, 'Hydrophobic' interactions, particularly 'Pi-Alkyl' subtypes, can vary in their bond distances.


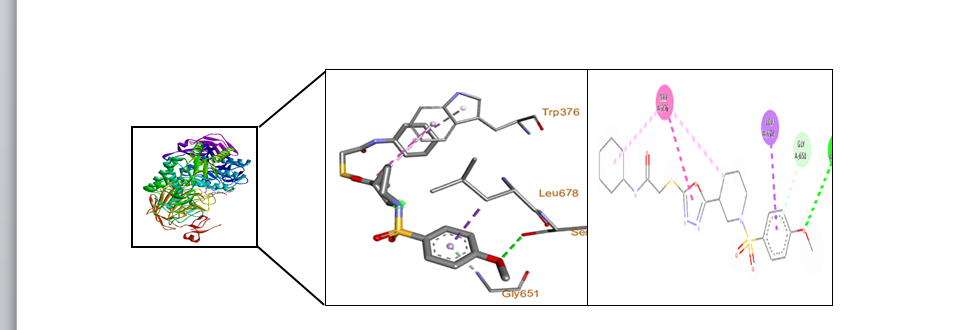


**Figure S55: Docking-generated complexes of compound** **7l against lipoxygenase**

The molecular interactions table for ligand **7m** offers a detailed overview of its hydrophobic contacts with various amino acid residues in chain B of a protein structure. All ten interactions are classified as hydrophobic, further divided into Pi-Sigma, Alkyl, and Pi-Alkyl types. The Pi-Sigma interaction, uniquely marked with a purple square and an angle deviation of 11.695°, occurs between LEU368 and the ligand’s pi-orbitals, suggesting a distinct spatial orientation. The remaining interactions—highlighted in pink—include four Alkyl interactions involving ligand atoms C33 and C36 with residues ILE406, ALA410, LEU373, and ARG4, and five Pi-Alkyl interactions between the ligand’s pi-orbitals and residues ALA410, LEU607, TRP147, and PHE151. Interaction distances range from 3.59 Å to 4.97 Å. Its binding affinity was 7.1 kcal/mol.

**
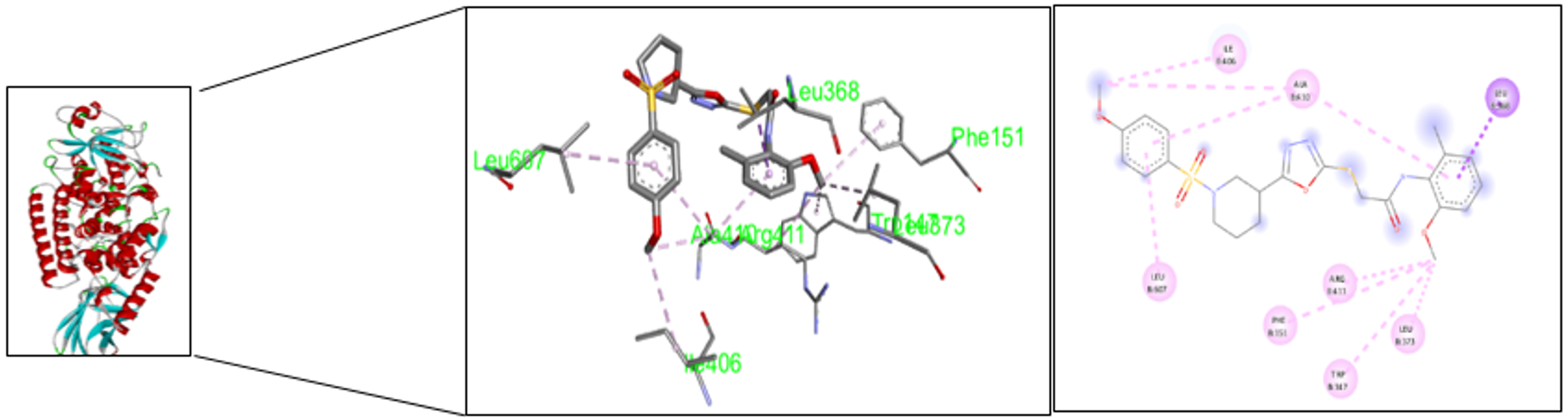
**

**Figure S56: Docking-generated complexes of compound** **7m against lipoxygenase**

The compound **7n** docking with same protein showed interactions in Figure S59. The Hydrogen bond interactions include carbon-hydrogen and Pi-Donor hydrogen are observed with GLN15 and ASN613 at 3.63 and 3.89 Aͦ respectively. The hydrophobic interactions in Pi-Sigma and Pi-Alkyl with SER171 and PHE402 at 3.99 and 4.96 Aͦ are revealed. At a bond distance of 4.02 Aͦ, one Pi-Sulfur interaction with PHE169 observed. It has binding affinity of -8.7 Kcal/mol.


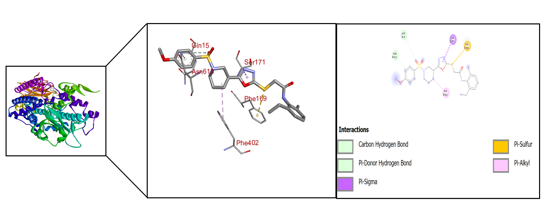


**Figure S57: Docking-generated complexes of compound** **7n against lipoxygenase**

**DOCKING STUDIES OF ALL COMPOUNDS AGAINST UREASE**

Compound **7a** was docked against urease with the PDB ID: 3LA4. Their interaction involves hydrogen bond, electrostatic and hydrophobic. Carbon hydrogen bond interaction observed with LYS745, THR33, and GLU742 at the bond distance of 3.60 Å, 3.72 Å, and 3.68 Å respectively. Pi-cation, pi-donor hydrogen bond observed with LYS716 at the bond distance of 4.12 Å. Pi-donor hydrogen bond observed with TYR32 at the bond distance of 3.57 Å. Pi-sigma interaction observed with VAL744 at the bond distance of 3.89 Å. Alkyl interaction revealed with LYS745 and VAL744 at the bond distance of 4.07 Å and 4.30 Å. Pi-alkyl interaction observed with LYS745, ALA16 and ALA37 at the bond distance of 5.27 Å, 3.69 Å and 5.10 Å. The binding affinity is -7.5 kcal/mol.


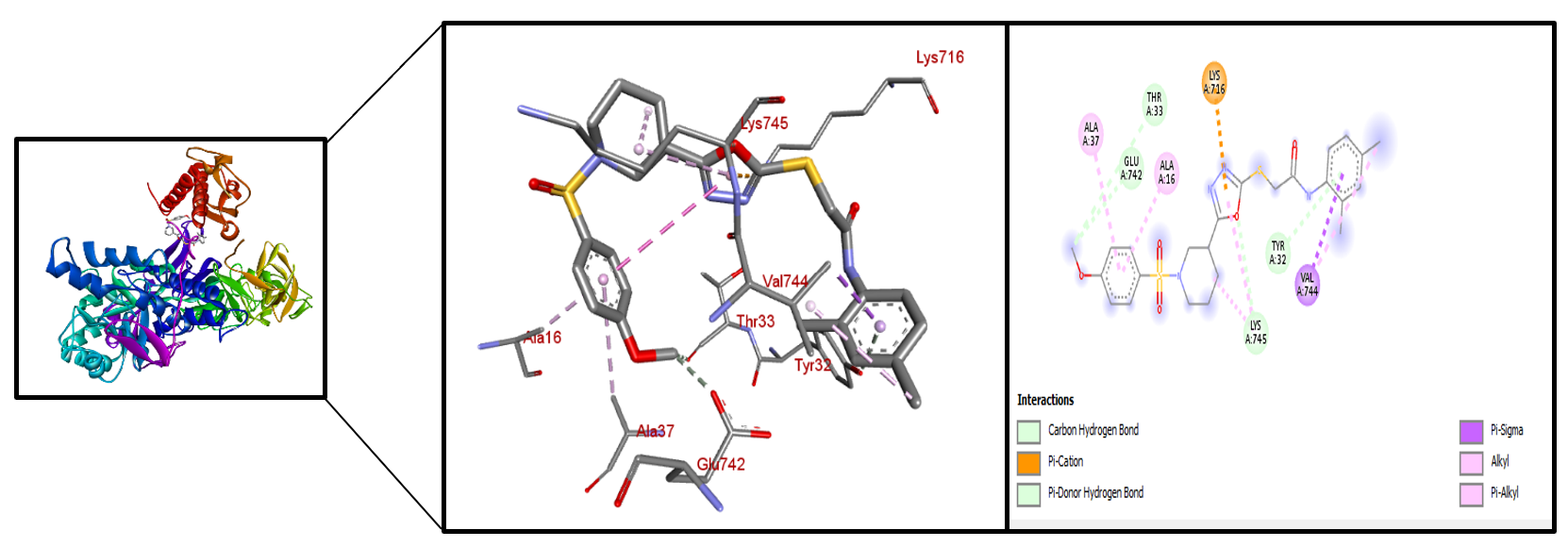


**Figure S58: Docking generated complexes of compound** **7a against urease**

Compound **7c** docking against urease protein having PDB ID 3la4. The interactions of compound **7c** with protein binding site. These interactions involve one electrostatic interaction such as Pi-Anion observed with GLU418 at a bond distance of 3.90 Aͦ. The multiple hydrophobic interactions including Pi-Pi Stacked, Pi-Pi T-Shaped and Pi-Alkyl with TYR417, PHE838 and PHE838 at 3.73, 4.81 and 5.42 Aͦ are observed. It has binding affinity of -7.2 Kcal/mol.


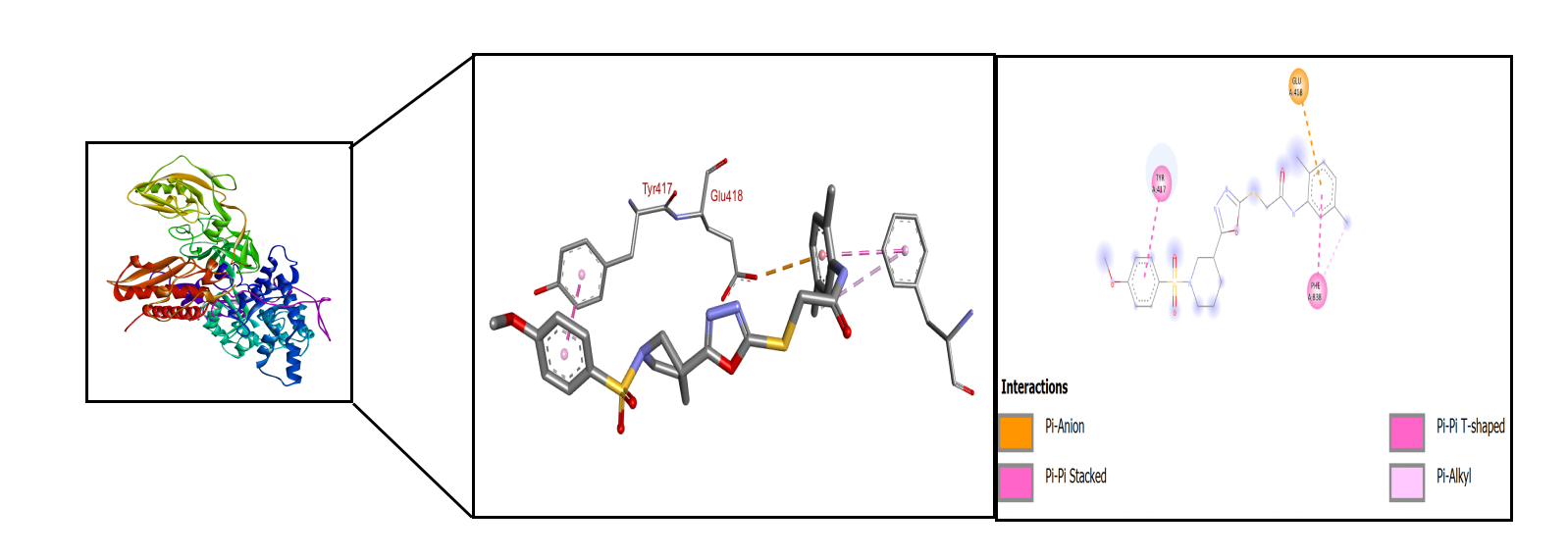


**Figure S59: Docking-generated complexes of compound** **7c against urease**

Compound **7d** showed several interactions with urease protein (PDB ID 3la4) in Figure S64. The docking results showed that two Carbon-Hydrogen bond interactions observed with ARG6 and THR91 at a bond distance of 3.18 and 3.72 Aͦ. A Pi-Donor hydrogen bond involving LYS10 at 4.14 Aͦ. The Pi-Anion interactions with GLU45 at 3.87 Aͦ is noticed. Several hydrophobic interactions include Pi-Sigma, two Alkyl and three Pi-Alkyl contacts with TYR18, ARG48, MET44, ARG6, LYS10 and LEU115 with distance of 3.68, 4.68, 5.31, 5.33, 4.13and 5.48 Aͦ respectively. It has binding affinity of -7.5 Kcal/mol.


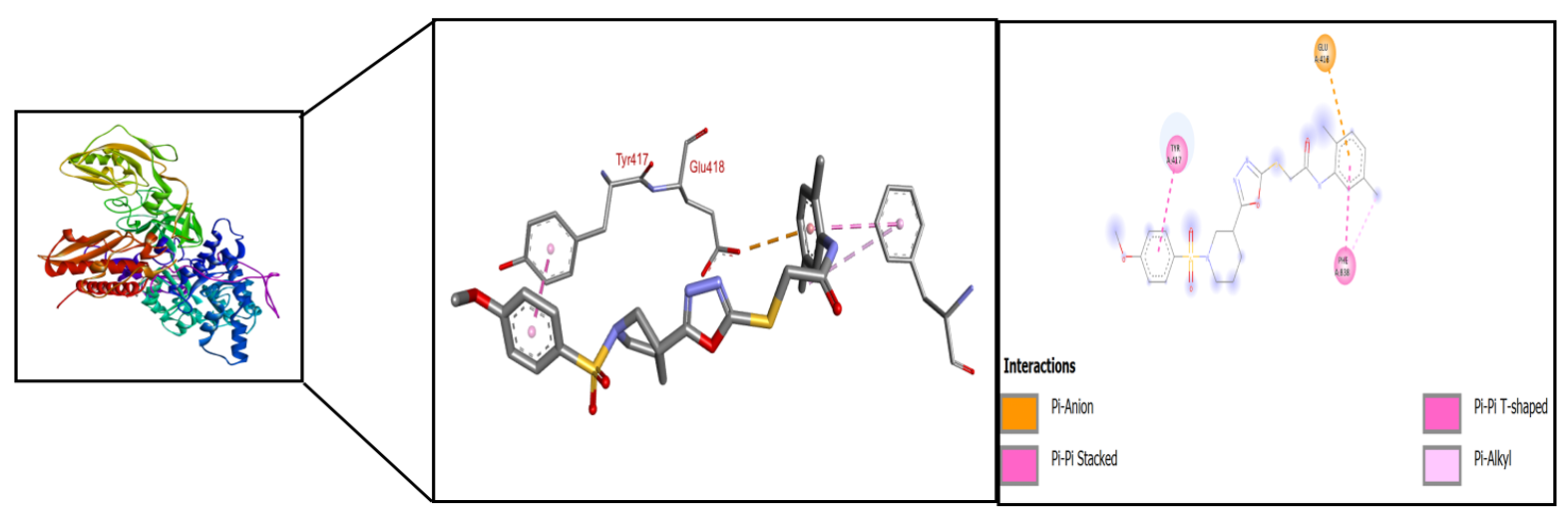


**Figure S60: Docking-generated complexes of compound** **7d against urease**

Synthesized compounds such as **7e** showed maximum Urease inhibition activity so they are docked with Urease confirm different interaction and binding capabilities making it more potent against Urease **(**Figure 65). These bindings of **7e** were confirmed with Urease protein with Urease (PDB ID: 3LA4). Docking studies revealed multiple interactions contributing to the stabilization of the ligand within the binding site. Two hydrogen bonds were observed with UN0:C35 and A: LYS716, at bond distances of 3.432 Å and 3.799 Å respectively. A Pi-sigma interaction observed with A: LEU519: CB at 3.907 Å. Several hydrophobic interactions were noted including π-alkyl and alkyl interactions involving residues such as A: LYS745, A: LEU519, A: LEU516, A: LEU518, A: TYR18, A: PHE740, and A: PHE712. These interactions occurred at distances ranging from 4.019 Å to 5.069 Å. It shows binding affinity of -7.4 Kcal/mol.


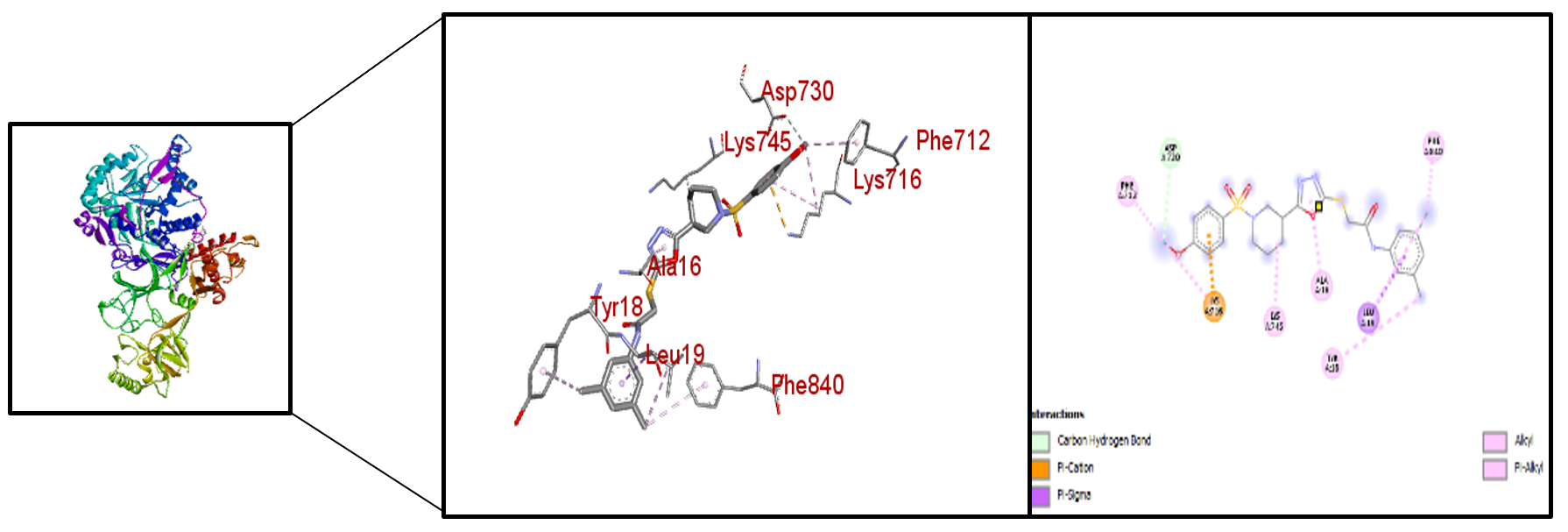


**Figure S61: Docking-generated complexes of compound** **7e against urease**

Docking of compound **7f** with urease protein having PDB ID 3LA4 showed interactions in Figure S66 the docking results showed the Hydrogen bond interactions involving conventional hydrogen bond, carbon hydrogen bond and Pi-Donor hydrogen bond with UNK0:N24 at a bond distance of 3.2 Å. A pi-cation and pi-anion interaction is also observed with A: LYS10:NZ and A: GLU45 at the bond distance of 4.3 Å, 3.5 Å respectively. The multiple hydrophobic interactions include Pi-Pi stacked, two Alkyl and two Pi-Alkyl with A: ARG48, UNK0, UNK0, UNK0 at 3.64, 4.51, 3.98, 4.22, 5.01, 5.35, 5.44, 4.18 Å respectively. It shows binding affinity of -7.5 Kcal/mol.


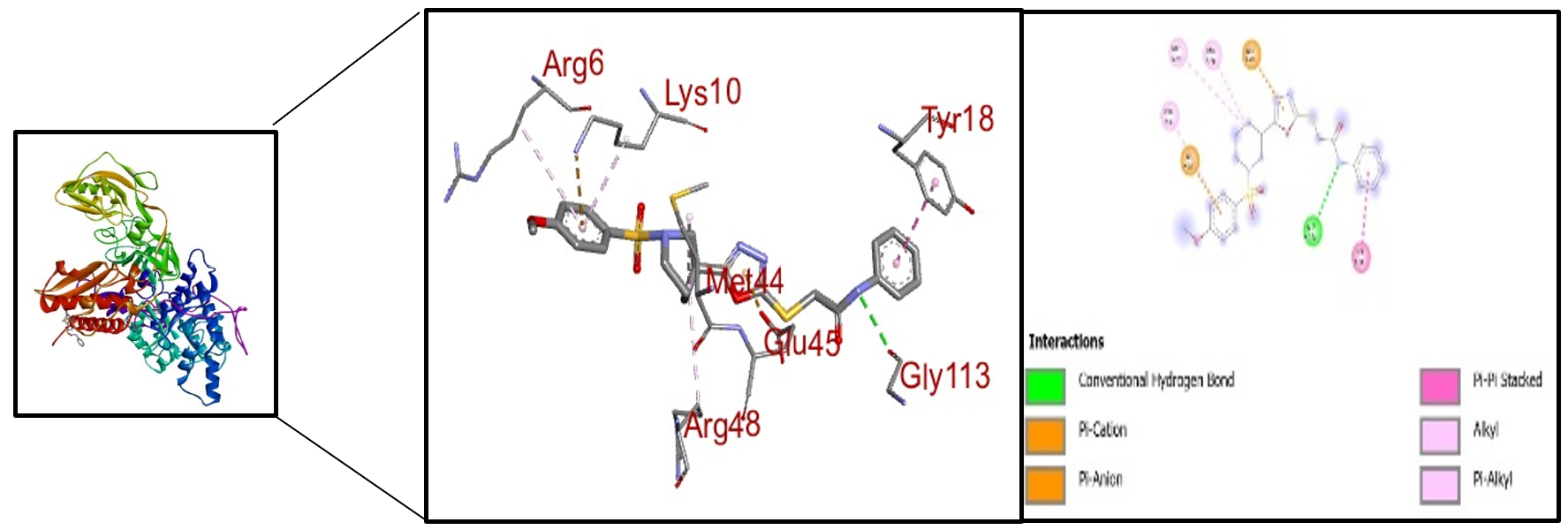


**Figure S62: Docking-generated complexes of compound** **7f against urease**

The compound shows **7g** shows nine interactions involving various chemical entities, each with specific properties. The first interaction, ligand:C15, is visible with a light green color, categorized as a Hydrogen Bond (Carbon Hydrogen) with 3.71426 Å, involving an H-Donor from ligand:C15 to an H-Acceptor at VAL7, with an angle XDA of 101.246°. The second interaction, A: LYS716: N, is visible with an orange color, classified as an Electrostatic (Pi-Cation) interaction with 4.57266 Å, where A: LYS716: N acts as a Positive entity, interacting with LIGAND via Pi-Orbitals. The third interaction, A: LYS716, is visible with a pink color, categorized as Hydrophobic (Alkyl) with 3.68449 Å, involving an Alkyl group from A: LYS716 to LIGAND. The fourth and fifth interactions, ligand:C34, are both visible with pink colors, classified as Hydrophobic (Alkyl) with distances of 4.40038 Å and 4.97151 Å respectively, interacting with A:PRO7 and A: LYS745 via Alkyl groups. The sixth interaction, A: TRP728, is visible with a pink color, categorized as Hydrophobic (Pi-Alkyl) with 4.96909 Å, involving Pi-Orbitals from A: TRP728 to LIGAND. The seventh, eighth, and ninth interactions, all involving ligand, are visible with pink colors, classified as Hydrophobic (Pi-Alkyl) with distances of 5.09512 Å, 3.99814 Å, and 5.36431 Å respectively, interacting with A: VAL44, A: ALA143, and A: LYS745 via Pi- Orbitals to Alkyl groups.


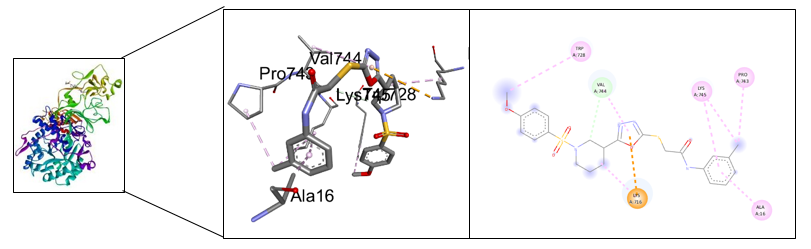


**Figure S63: Docking-generated complexes of compound** **7g against urease**

In compound **7h**, the table details fourteen interactions involving various chemical entities, each with distinct properties. The first interaction, A: THR33:C, is visible with a light green color, categorized as a Hydrogen Bond (Carbon Hydrogen) with 3.17036 Å, involving an H-Donor from A: THR33:CA to an H-Acceptor at ligand, with an angle XDA of 9.264°. The second interaction, ligand:C11, is visible with a light green color, classified as a Hydrogen Bond (Carbon Hydrogen) with 3.77521 Å, where ligand:C11 acts as an H-Donor to an H-Acceptor at A: THR3, with an angle XDA of 107.025°. The third interaction, ligand:C15, is visible with a light green color, categorized as a Hydrogen Bond (Carbon Hydrogen) with 3.33447 Å, involving an H-Donor from ligand:C15 to an H-Acceptor at A: VAL7, with an angle XDA of 93.864°. The fourth interaction, A: LYS716: N, is visible with an orange color, classified as Electrostatic (Pi-Cation) with 4.27555 Å, where A: LYS716: N acts as a Positive entity, interacting with LIGAND via Pi-Orbitals. The fifth interaction, A: TYR32: OH, is visible with an orange color, categorized as a Hydrogen Bond (Pi-Donor H) with 3.42798 Å, involving an H-Donor from A: TYR32: OH, to ligand via Pi-Orbitals. The sixth interaction, A: VAL744:C, is visible with a purple color, classified as Hydrophobic (Pi-Sigma) with 3.89573 Å, where A: VAL744:C acts as a C-H entity, interacting with ligand via Pi-Orbitals. The seventh interaction, A: LEU839:C, is visible with a purple color, categorized as Hydrophobic (Pi-Sigma) with 3.66522 Å, involving a C-H from A: LEU839:C to ligand via Pi-Orbitals. The eighth interaction, A: TYR32, is visible with a pink color, classified as Hydrophobic (Pi-T-shape) with 5.27445 Å, involving Pi-Orbitals from A: TYR32 to ligand. The ninth interaction, A: ALA16, is visible with a pink color, categorized as Hydrophobic (Alkyl) with 4.71661 Å, involving an Alkyl group from A: ALA16 to ligand. The tenth interaction, A: ALA37, is visible with a pink color, classified as Hydrophobic (Alkyl) with 4.37022 Å, involving an Alkyl group from A: ALA37 to ligand. The eleventh interaction, ligand:C33, is visible with a pink color, categorized as Hydrophobic (Alkyl) with 3.70837 Å, involving an Alkyl group from ligand:C33 to A: VAL744. The twelfth interaction, ligand, is visible with a pink color, classified as Hydrophobic (Pi-Alkyl) with 5.38599 Å, involving Pi-Orbitals from ligand to an Alkyl group at A: VAL36. The thirteenth interaction, ligand, is visible with a pink color, categorized as Hydrophobic (Pi-Alkyl) with 3.83764 Å, involving Pi-Orbitals from ligand to an Alkyl group at A: LYS716. The fourteenth interaction, ligand, is visible with a pink color, classified as Hydrophobic (Pi-Alkyl) with 5.28581 Å, involving Pi-Orbitals from ligand to an Alkyl group at A: MET7

**
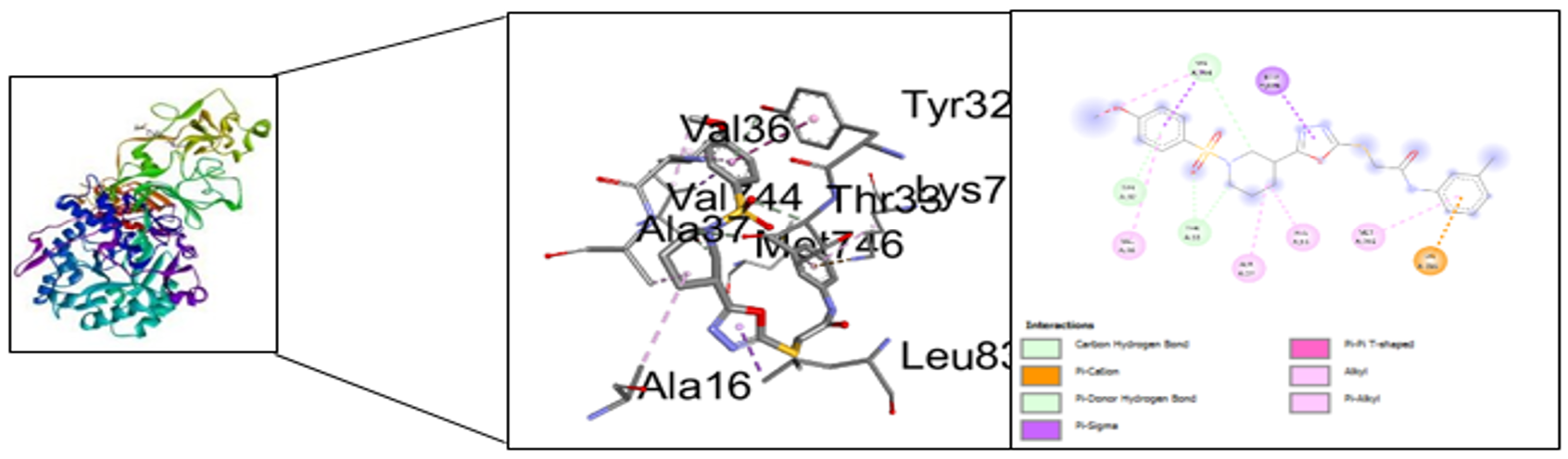
**

**Figure S64: Docking-generated complexes of compound** **7h against urease**

Among the synthesized Compound, **7i** exhibited different interactions with Urease inhibitor. Their interactions are shown in Figure 2. One Pi-Pi stacked bond interaction observed with TYR417 at a bond distance 3.90 Å. A Pi-Pi T Shaped interaction observed with TRP728 at a bond distance of 5.67 Å. One alkyl Bond shows interaction with LEU460 at bond distance 5.29 Å. The five Pi-Alkyl interactions revealed with TYR417, TYP417, PHE838, PRO748, and LYS716 at 5.17 Å, 4.94 Å, 4.16 Å, 5.19 Å, 4.93 Å, and 5.24 Å respectively. Its binding affinity is -7.6 kcal/mol.


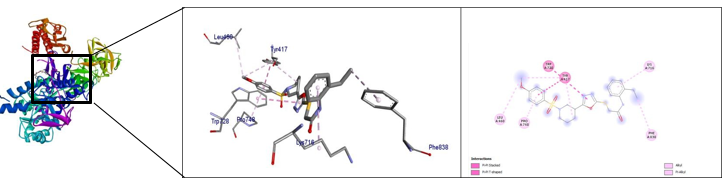


**Figure S65: Docking-generated complexes of compound** **7i against urease**

The docking of compound **7j** with urease protein having PDB ID 3la4 showed interactions. Their interactions involve conventional hydrogen bond with CYS143 at 3.38 Å. Several hydrophobic interactions including Two Pi-Sigma are observed with VAL544, TRP126 at 3.55 Å and 3.76 Å respectively. The two alkyl interactions were observed with ILE148 and CYS143 at 5.44 Å and 4,42 Å. A one Pi- alkyl interaction with PRO266 at 5.34 is observed. Its binding affinity is -7.0 kcal/mol.


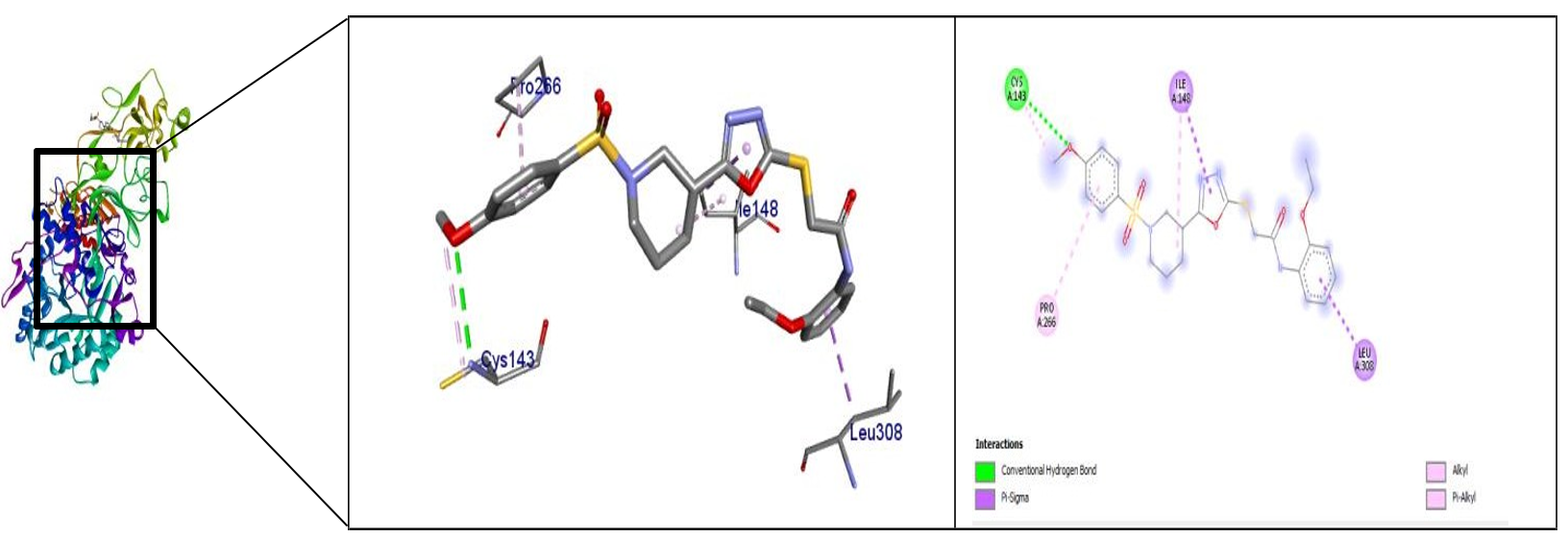


**Figure S66: Docking-generated complexes of compound** **7j against urease**

Compound **7k** docked against urease with PDB ID 3la4. Their interactions are shown in Figure S71. The ligand is shown in a stick representation, with different colors indicating various atoms. Several residues of the protein 3la4 are labelled (TYR32, VAL36, ALA37, ALA16, GLU742, VAL744, ASP730, LYS716, PHE712, LEU839), indicating their involvement in the interaction with the ligand. The image highlights various interactions between the ligand and the protein residues, including hydrogen bonds and possibly π-π interactions or other non-covalent interactions, which are crucial for the stability and specificity of the ligand-protein complex. The software interface is also visible, listing entries with checkboxes for visibility and tagging, allowing users to manage multiple structures or results.

"Hydrophobic" with "Pi-Alkyl" types exhibit a range of bond distances between 4.06 and 5.42074 Å. Notably, the majority of these interactions fall within a narrower range of 4.06 to 5.22634 Å, suggesting a relatively consistent bonding pattern, and ":UNKO:S21 ...", display distinct bond distances and categories, including "Hydrogen Bond" and "Other", with distances of 3.58537 and 3.28075 Å, respectively. Overall, the data suggests that the name type is correlated with the bond distance, with "Pi-Alkyl" interactions tend to have longer bond distances compared to other categories.


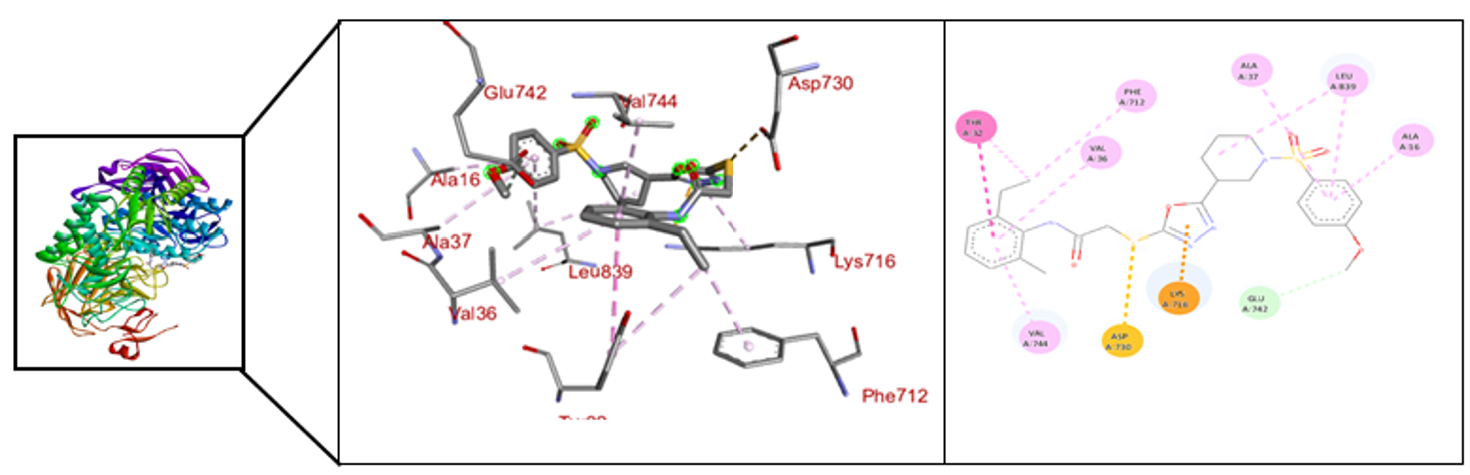


**Figure S67: Docking-generated complexes of compound** **7k** **against urease**

The interactions of compound **7l** with urease protein are shown in Figure S72. the ligand docked into the protein's active site, with different colours representing various atoms and green dashed lines indicating interactions such as hydrogen bonds. The image understanding binding affinity and specificity. hydrogen bond is between a hydrogen donor (H-Donor) and a hydrogen acceptor (H-Acceptor). The "Types" column mentions "Carbon Hyd...", implying the involvement of a carbon-containing group, likely in the context of the ligand. The angles provided, "Angle XDA" at 92.332 degrees and "Angle D" at 90.202 degrees, offer additional geometric context to this interaction, suggesting a nearly perpendicular arrangement. Overall, this data suggests a significant hydrogen bonding interaction between the ligand and the protein, with the specifics of the bond geometry indicating a relatively strong and directed interaction.


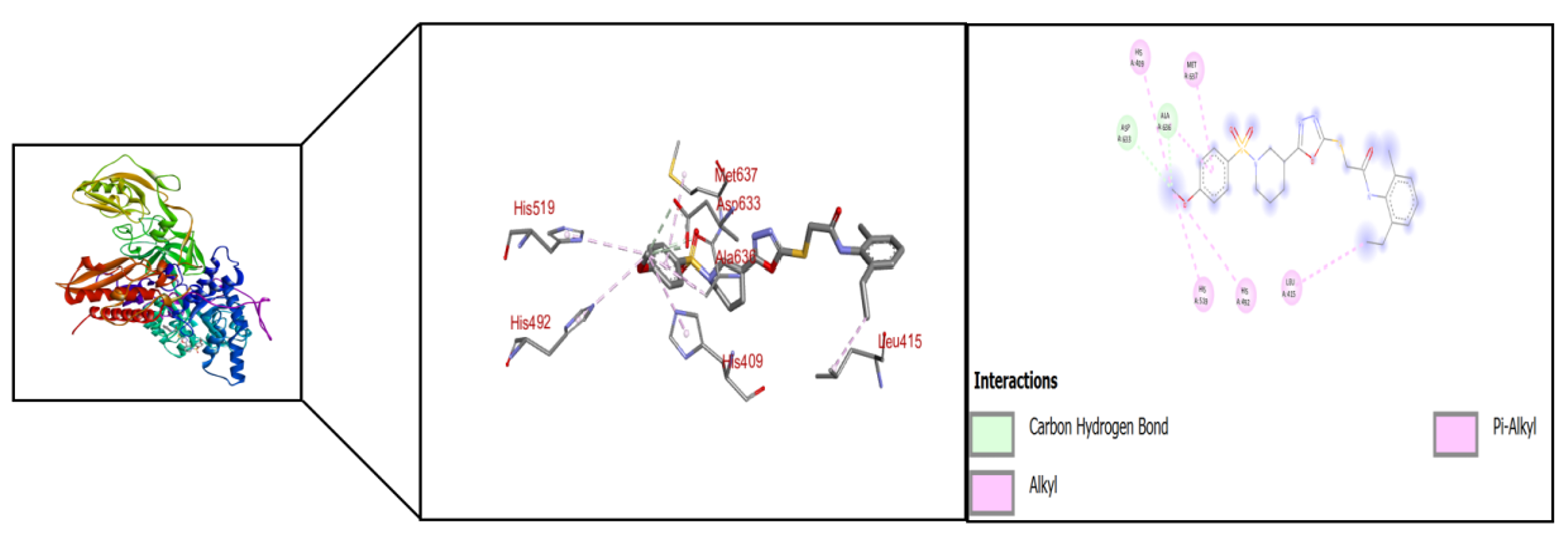


**Figure S68: Docking-generated complexes of compound 7n against urease**

Docking of compound **7o** with urease protein having PDB ID 3LA4 showed interactions in Figure S74 the docking results showed the Hydrogen bond interactions involving conventional hydrogen bond, carbon hydrogen bond and Pi-Donor hydrogen bond with LYS10 and THR86 at a bond distance of 3.36, 3.35 and 4.14 Å. the multiple hydrophobic interactions include Pi-Sigma, two Alkyl and three Pi-Alkyl with TYR18, ARG48, LEU115, LYS10, ALA85 and LEU115 at 3.64, 4.51, 3.98, 4.28, 5.48 and 5.40 Å respectively. It shows binding affinity of -7.6 Kcal/mol.


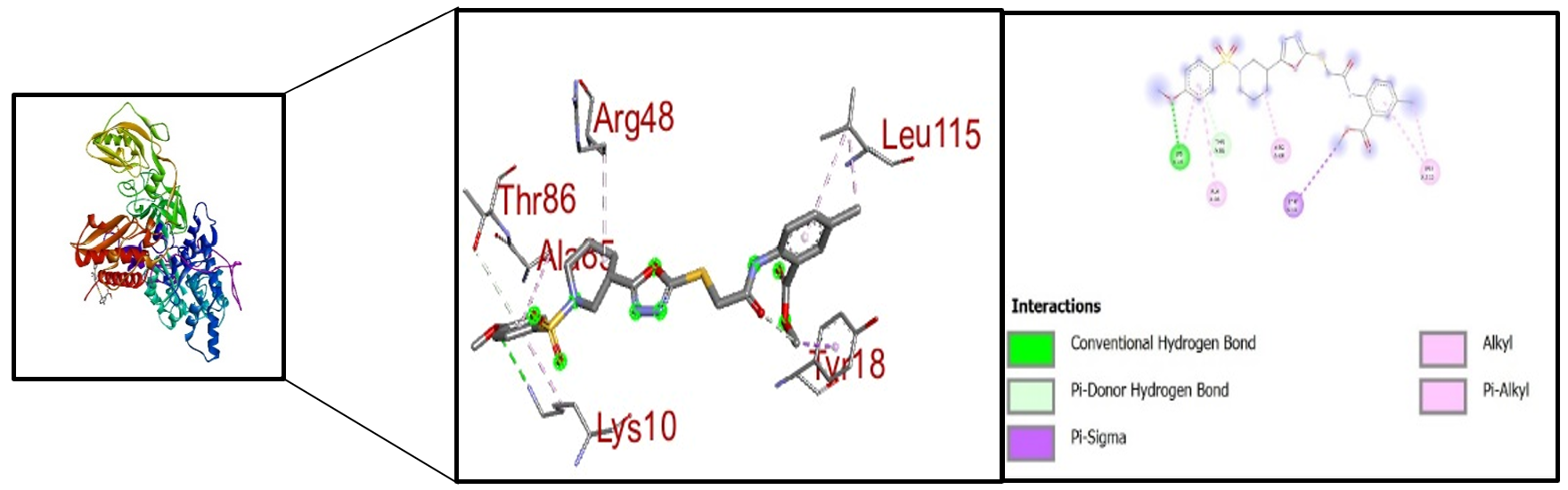


**Figure S69: Docking-generated complexes of compound** **7o against urease**

**DOCKING STUDIES OF ALL COMPOUNDS AGAINST ALPHA GLUCOSIDASE**

Compound **7a** was docked against alpha glucosidase with the PDB ID: 5NN6. Their interactions are shown in Figure S75. Their interaction involves hydrogen bond, electrostatic hydrophobic and others. Carbon hydrogen bond interactions observed with HIS717 and GLU866 at the bond distance of 3.26 Å and 3.63 Å. Pi-cation observed with ARG608 at the bond distance of 4.97 Å. Pi-sigma interaction observed with TYR360 at the bond distance of 3.91 Å. Pi-sulfur interaction observed with HIS717 at the bond distance of 4.95 Å. Pi-pi T shaped interaction observed with HIS717 at the bond distance of 4.65 Å. Alkyl interaction revealed with ARG594 and VAL867 at the bond distance of 5.18 Å and 4.46 Å. Pi-alkyl interaction observed with TYR360, ARG608, LEU868 and ARG594 at the bond distance of 4.55 Å, 5.13 Å, 5.18 Å and 5.47 Å. The binding affinity is **-**7.4 kcal/mol.


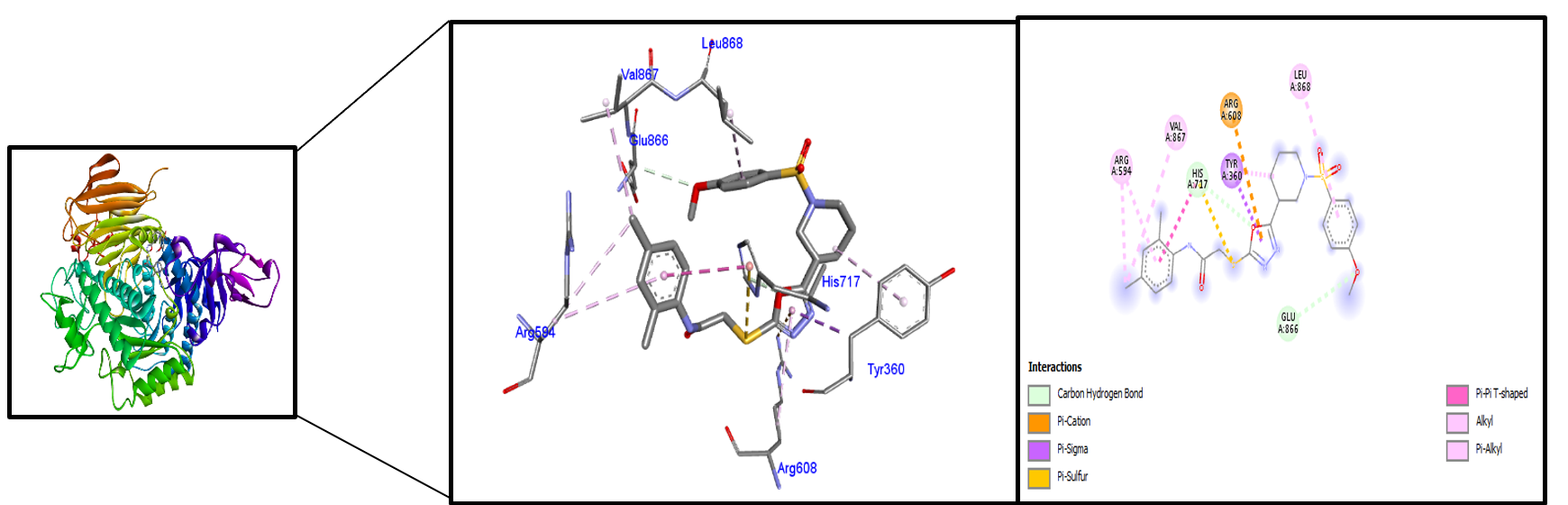


**Figure** **S70: Docking generated complexes of compound** **7a alpha glucosidase**

Compound **7b** was docked against alpha glucosidase with the PDB ID: 5NN6. Their interactions are shown in Figure S75. Their interaction involves hydrogen bond and hydrophobic. Carbon hydrogen bond interactions observed with HIS717 and SER864 at the bond distance of 3.59 Å and 3.61 Å. Pi-Donor hydrogen bond observed with TYR360 at the bond distance of 4.08 Å. Pi-sigma interaction observed with TYR360 at the bond distance of 3.74 Å. Pi-Pi stacked interaction observed with TYR360 at the bond distance of 4.67 Å. Alkyl interaction revealed with VAL867 and LEU868 at the bond distance of 4.49 Å and 4.47 Å. Pi-alkyl interaction observed with HIS717 and LEU868 at the bond distance of 4.81 Å and 4.20 Å. The binding affinity is -8.0 kcal/mol.


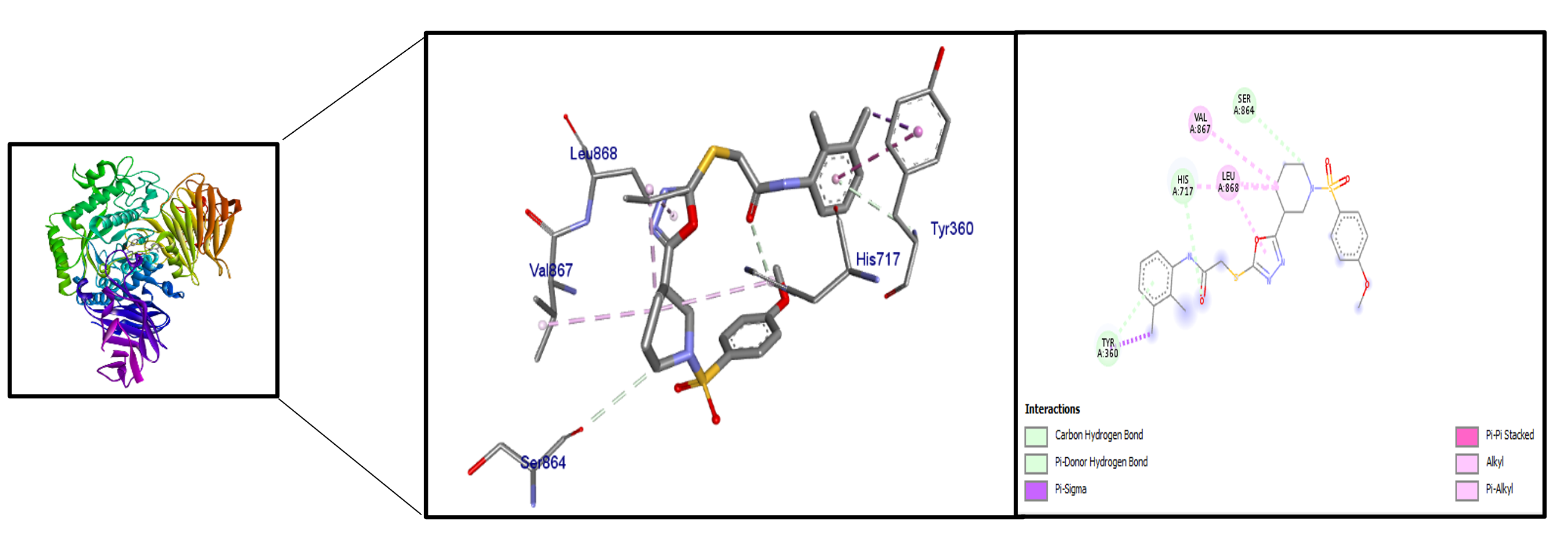


**Figure S71: Docking generated complexes of compound** **7b alpha glucosidase**

The compound **7d** interactions with same protein active sites are shown in Figure S78. The docking study showed the hydrogen bond interactions involve conventional hydrogen bond, carbon-hydrogen bond and Pi-Donor hydrogen bond with LEU165, THR166 and ASN570 at 3.13, 3.73 and 4.00 Aͦ. The electrostatic interactions such as two Pi-Anion revealed with ASP243 at 3.39 and 4.69 Aͦ respectively. A Pi-Pi Stacked interaction observed with TYR569 at 5.54 Aͦ. at a bond distance of 5.47 and 4.70 Aͦ, Alkyl and Pi-Alkyl interactions noticed with ILE164. It has binding affinity of -7.8 Kcal/mol.


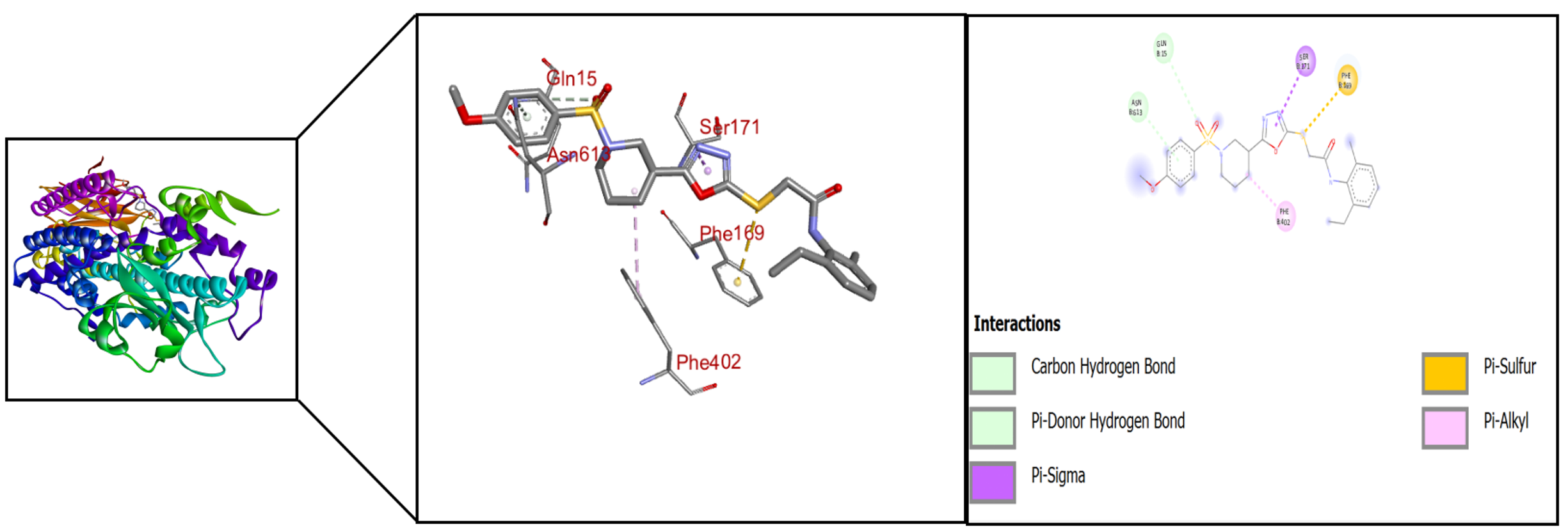


**Figure S72: Docking-generated complexes of compound** **7d alpha glucosidase**

Synthesized compounds such as **7e** showed maximum alpha glucosidase inhibition activity so they are docked with alpha glucosidase (PDB ID 5NN6) confirm different interaction and binding capabilities making it more potent against alpha glucosidase. A carbon hydrogen interaction observed at bond distance of 3.43 Aͦ. Two pi-cation interactions also observed with A: ARG43 and A: ASP513 at the bond distance of 3.45 Aͦ and 3.80 Aͦ respectively. There are two pi-anion interactions also observed with A: AASP513 and A: GLU863 at the bond distance of 4.14 Aͦ and 4.84 Aͦ. A Pi-Pi T- shaped interaction is noted with A: HIS432 at the bond distance of 5.2 Aͦ. Many alkyl interaction is noted at the bond angle 4.6 Aͦ, 4.67 Aͦ, 4.99 Aͦ respectively. Two Pi-alkyl hydrophobic interactions with A: HIS507, A: TRP859 at the bond distance 4.95 Aͦ, 4.82 Aͦ respectively observed. Two Pi-alkyl interactions noted with A: TRP859, at the bond distance of 5.47 Aͦ, 4.97 Aͦ respectively. It shows binding affinity of -7.1 Kcal/mol.


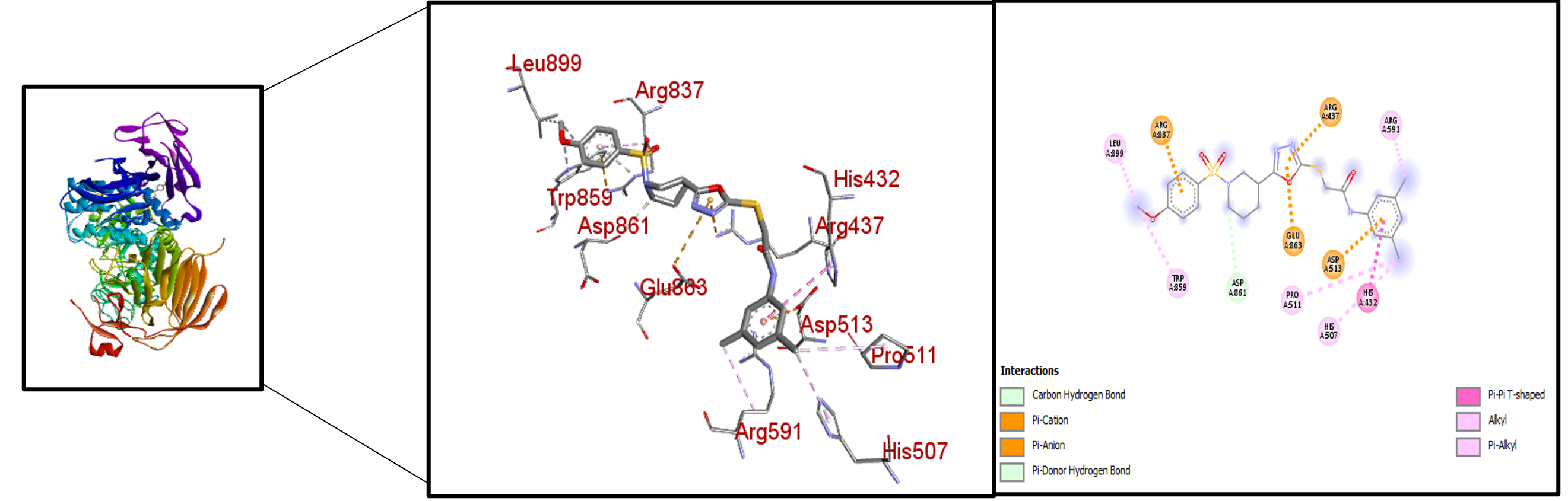


**Figure S73: Docking-generated complexes of compound** **7e alpha glucosidase**

Compound **7f** showed minimum alpha glucosidase inhibition activity so it is also docked with same protein of (PDB ID 5NN6) Figure S80. Three C-H interactions are observed with A: HIS584, A: HIS584, A: HIS589 at the bond distance of 3.18Aͦ, 3.26 Aͦ, 3.63 Aͦ respectively. A Pi-Sigma and Pi-Sulfur is also noted with A: VAL588 at the bond distance of 3.46 Aͦ, 4.90 Aͦ respectively. Many Hydrophobic interactions also observed Pi-Pi stacked with A: TYR360, Pi-Pi T-Shaped with A: HIS717, Alkyl, a pi- alkyl at the distance of 3.77Aͦ, 5.56 Aͦ, 4.92 Aͦ, 5.04 Aͦ respectively. These interactions collectively create a strong binding network that reinforces ligand affinity and enhances molecular stability within the active site. It shows binding affinity of -7.6 Kcal/mol.


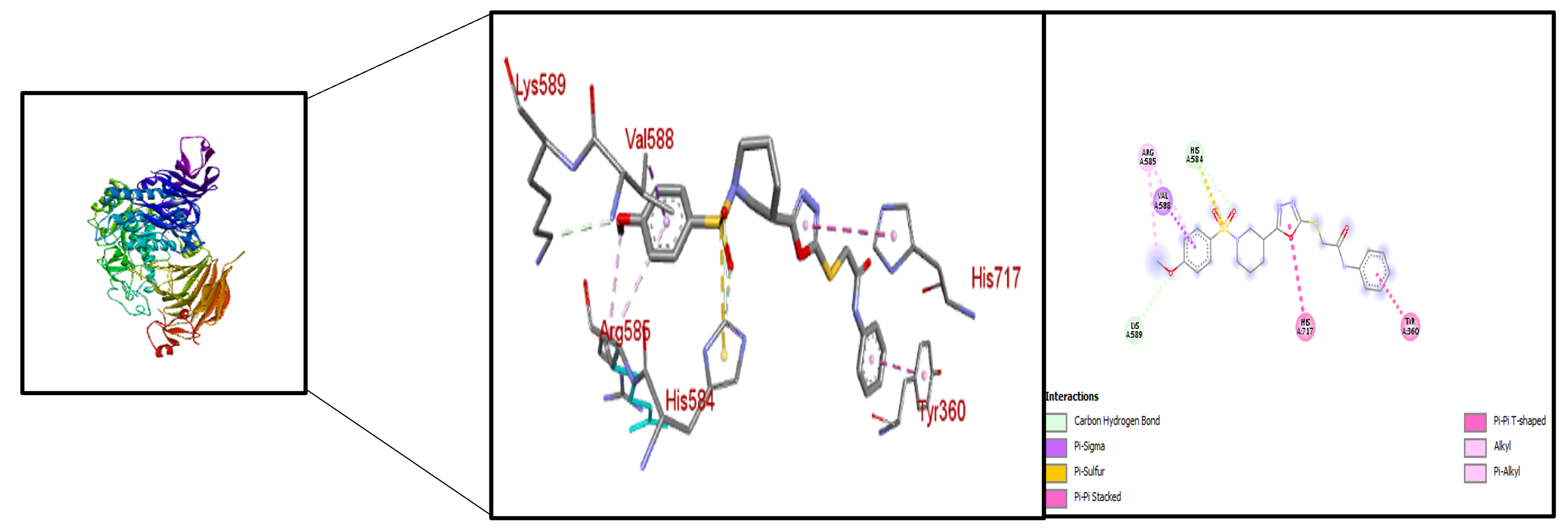


**Figure S74: Docking-generated complexes of compound 7f alpha glucosidase**

The molecular interaction data for ligand **7g** reveals eight distinct interactions with residues in chain A of a protein, encompassing hydrogen bonding, hydrophobic contacts, and other non-covalent interactions. All interactions are marked as visible and vary in type, including Pi-Sulfur, Pi-Pi stacking, Pi-Pi T-shaped, and Pi-Alkyl. The first interaction is a hydrogen bond between PRO595 and ligand, with a donor-acceptor relationship and precise angular measurements (23.421° and 116.45°), indicating strong orientation. A Pi-Sulfur interaction with HIS584 occurs at a longer distance (5.94 Å), suggesting a weaker but relevant contact. Hydrophobic interactions dominate the rest, with aromatic residues like TYR360, HIS717, and PHE362 engaging ligand’s pi-orbitals through Pi-Pi and Pi-Alkyl contacts, ranging from 4.06 Å to 5.01 Å. Its binding energy was -7.4 kcal/mol.

**
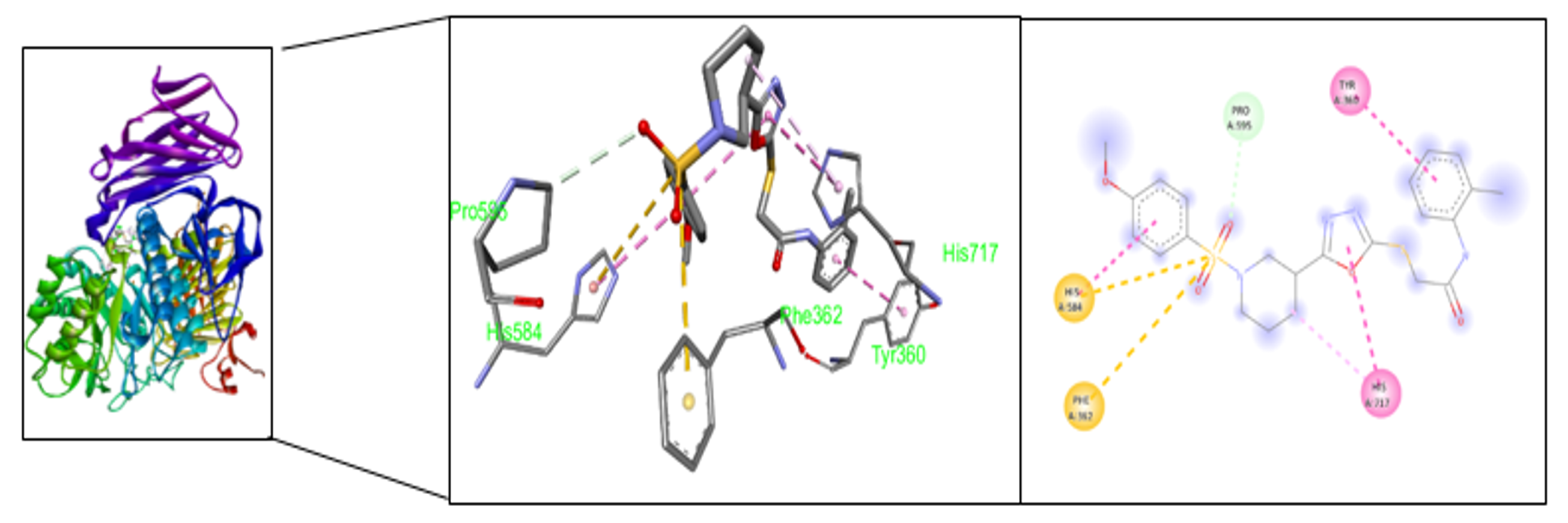
**

**Figure S75:** **Docking generated complex of compound** **7g alpha glucosidase**

The interaction table for ligand **7h** outlines ten molecular contacts with various residues in chain A of a protein, encompassing hydrogen bonding, electrostatic, and hydrophobic interactions. Two hydrogen bonds are formed between the CE atom of LYS903 and LIGAND, with donor-acceptor distances of 3.58 Å and 3.36 Å and angle deviations of 14.061° and 31.343°, respectively, indicating strong directional bonding. An electrostatic Pi-Cation interaction is also observed between the positively charged LYS903 and the ligand’s pi-orbitals at 4.27 Å. Hydrophobic interactions dominate the remaining entries, including Pi-Sigma, Pi-Pi T-shaped, Alkyl, and Pi-Alkyl types. A Pi-Sigma contact occurs between ALA842 and ligand at 3.87 Å, while a Pi-Pi T-shaped interaction is noted between two regions of ligand itself at 4.44 Å, suggesting intramolecular stabilization. Alkyl interactions involve ALA704 and ILE780, as well as LEU826 with ligand’s C33 atom, with distances ranging from 3.99 Å to 4.57 Å. Finally, Pi-Alkyl interactions between ligand’s pi-orbitals and LYS903 occur at longer distances of 4.98 Å and 5.39 Å, reflecting weaker but spatially relevant hydrophobic contacts. Its binding energy was -6.4 kcal/mol.


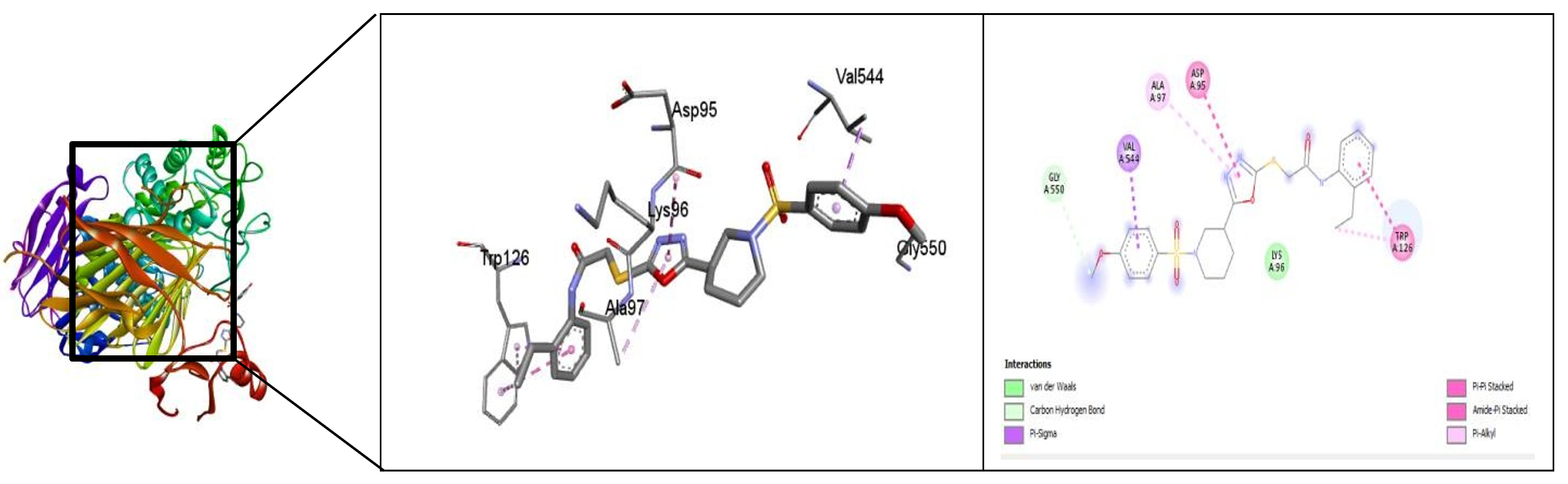


**Figure S76: Docking generated complexes of compound** **7h alpha glucosidase**

Among the synthesized Compound, **7i** Shows the different interactions with Alpha Glucosidase inhibitor. Their interactions are shown in Figure S83. One C-H bond interaction observed with GLY550 at a bond distance 3.64 Å. A Pi-sigma interaction observed with VAL544 at a bond distance of 3.53 Å. Two Pi-Stacked interaction observed withTRP126 and TRP127 at bond distance 3.83 and 4.62 Å respectively. One amid pi-stacked shows interaction with ASP95 AND LYS96 at distance 4.47 Å. The three Alkyl interactions revealed with TRP126, TYP126 AND ALA97 at 4.91 Å, 4.32 Å, 5.24 Å respectively. Its binding affinity is -7.8 kcal/mol.


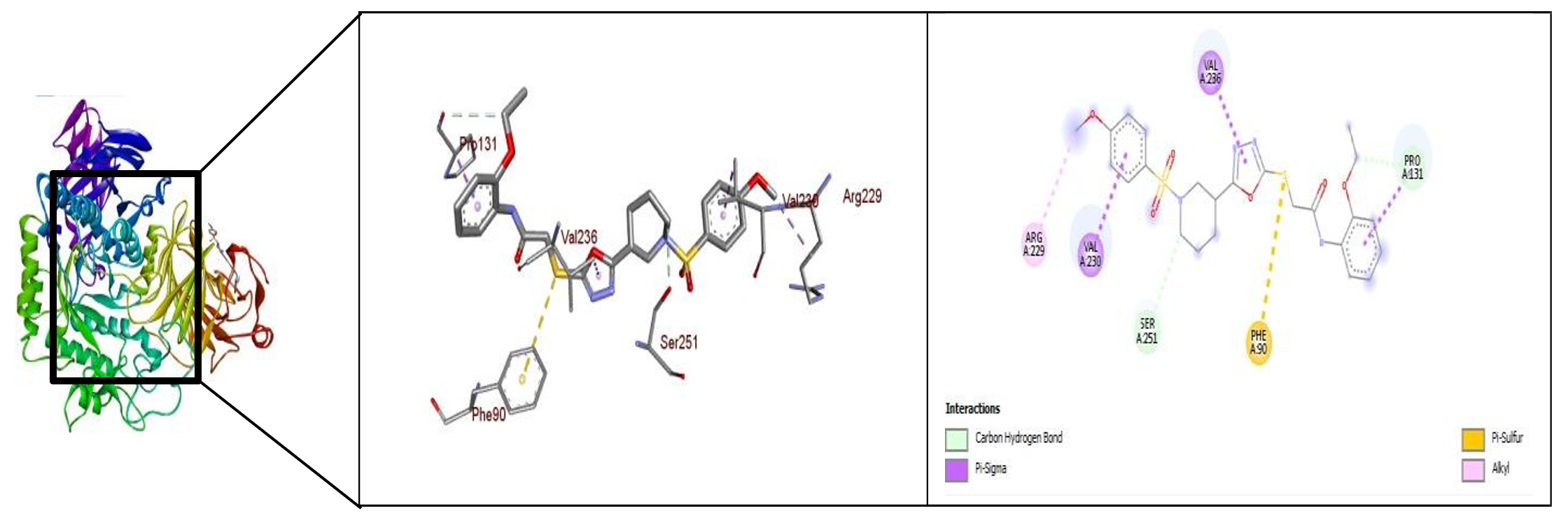


**Figure S77: Docking-generated complexes of compound** **7i alpha glucosidase**

Compound **7j** docking against Alpha Glucosidase protein. The interactions of compound 7j with protein binding site are shown in Figure S82. These interactions involve two C-H bond interactions SER251 and PRO131 at a bond distance of 3.72 Å and 3.72 Å. The multiple hydrophobic interactions including two Pi-sigma, Pi sulfur and Pi-Alkyl with VAL230, VAL236, PHE90 PHE90 and AGR229 at 3.90 Å, 3.79 Å, 5.239 Å and 3.81 Å are observed. Its binding affinity is -7.9 kcal/mol.

**
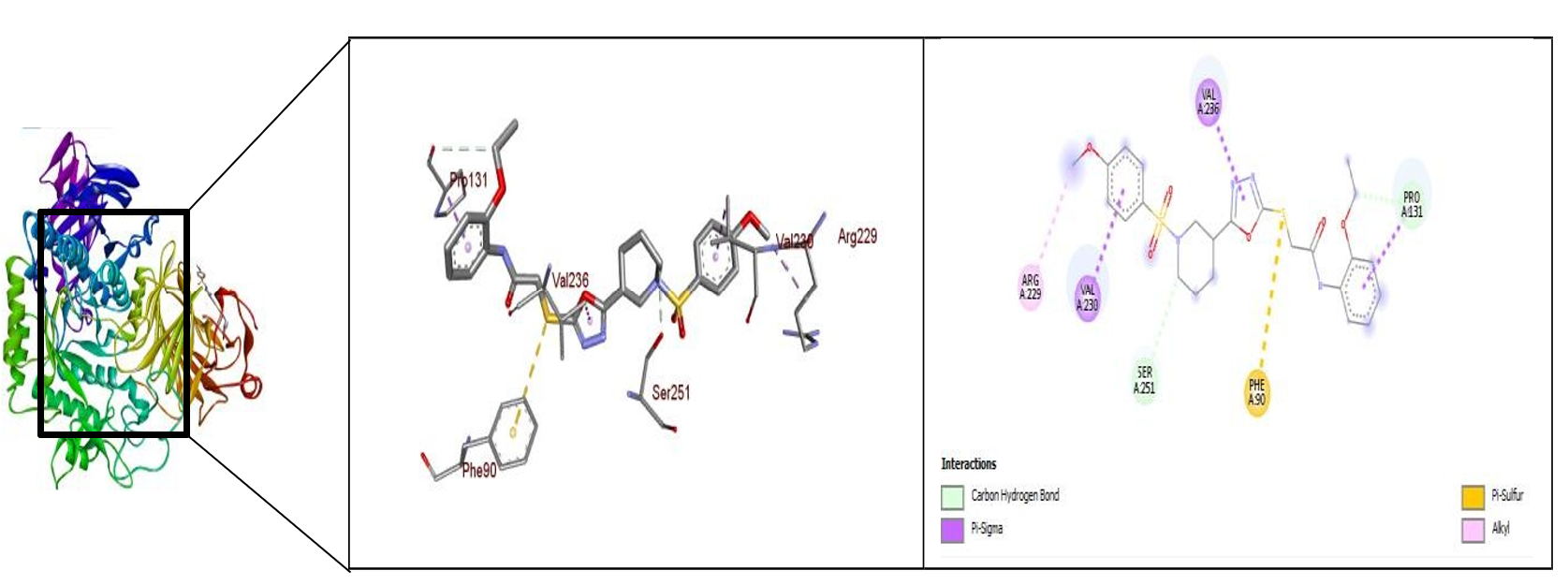
**

**Figure S78: Docking-generated complexes of compound** **7j alpha glucosidase**

The compound **7k** docking with alpha glucosidase protein PDB.ID 5nn6. The protein is represented by various amino acid residues, such as Phe858, Leu668, and Arg585, which are labeled and shown interacting with the ligand. The ligand is positioned within the protein's binding site, forming hydrogen bonds and other non-covalent interactions with the surrounding amino acids. The bond distances for these interactions range from 3.21517 to 5.25458 Å. Notably, the Hydrogen Bond interactions (rows 1 and 3) exhibit relatively shorter distances, with values of 3.21517 and 3.85071 Å, respectively. In contrast, the Hydrophobic interactions (rows 4-13) display a broader range of distances, spanning from 3.63545 to 5.25458 Å. Furthermore, the Pi-Alkyl interactions tend to have longer distances, generally above 4.5 Å. This suggests that the type of interaction and the corresponding bond distance are related, with Hydrogen Bonds typically being shorter and more specific, while Hydrophobic interactions, including Pi-Alkyl, can vary in distance.


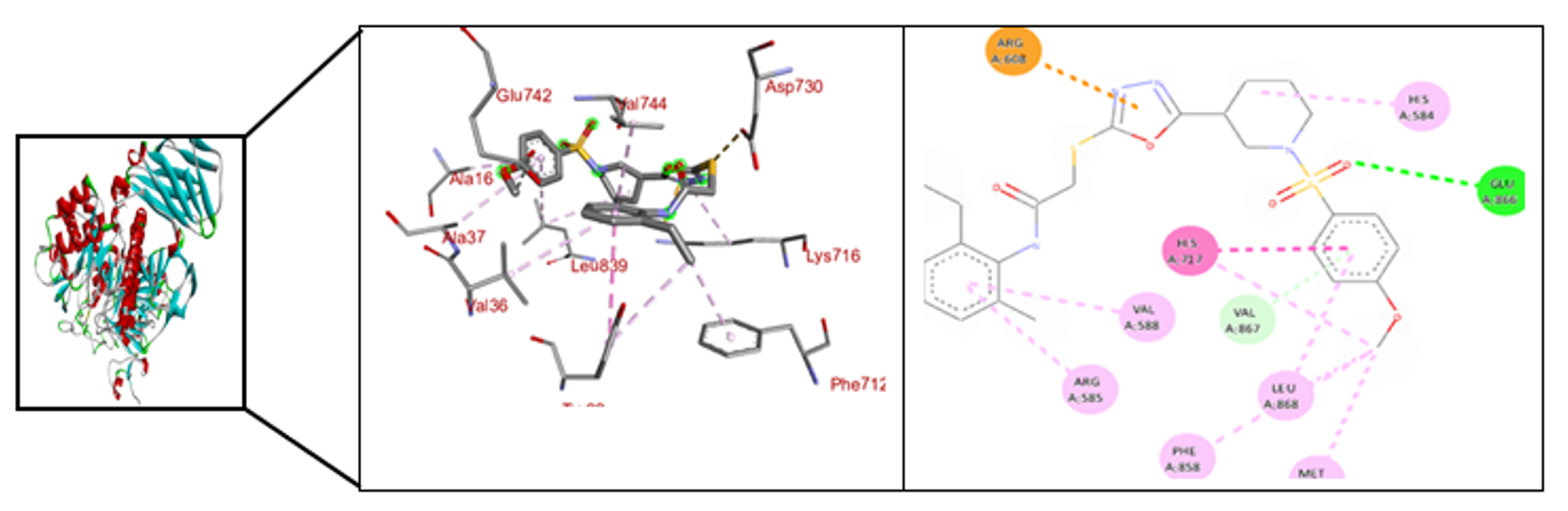


**Figure S79: Docking-generated complexes of compound** **7k alpha glucosidase**

Compound **7l** also dock with 5nn6 protein and their interactions are shown in Figure S86 colours indicating various atoms. Several protein residues (TRP376, LEU678, SER679, GLY651) are labelled, indicating their involvement in the interaction with the ligand and suggesting their significance for understanding the binding mode or affinity. The image highlights various interactions, including hydrogen bonds (green dashed lines) and possibly π-π interactions or other non-covalent interactions (purple dashed lines), crucial for the stability and specificity of the ligand-protein complex. For 'Hydrogen Bond' interactions (rows 1 and 2), the bond distances are 3.38 and 4.11142, respectively. In contrast, 'Hydrophobic' interactions (rows 3-7) exhibit a range of distances from 3.98 to 5.94. Notably, the 'Pi-Alkyl' subtype within 'Hydrophobic' interactions (rows 5-7) displays a relatively narrower range of distances, from 4.21 to 5.34. This suggests that while 'Hydrogen Bond' interactions tend to have shorter distances, 'Hydrophobic' interactions, particularly 'Pi-Alkyl' subtypes, can vary in their bond distances.


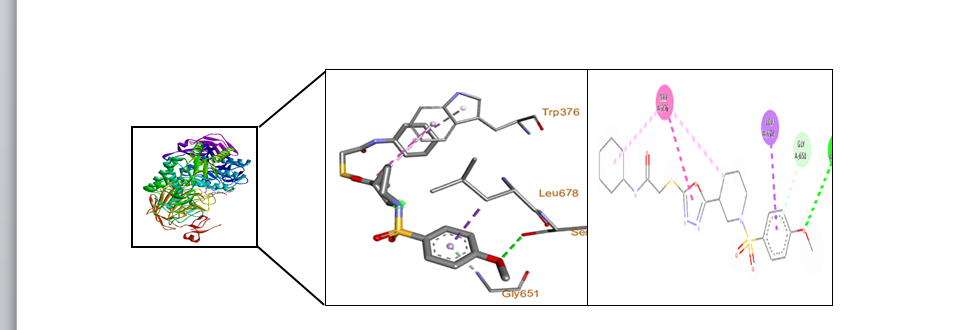


**Figure S80: Docking-generated complexes of compound** **7l alpha glucosidase**

The molecular interaction table for ligand **7m** presents eight distinct interactions with various residues, categorized into hydrogen bonding, electrostatic, hydrophobic, and other non-covalent types. Two hydrogen bonds are identified: one conventional bond between N24 and another atom at a distance of 3.13 Å with an angle of 99.83°, and a carbon hydrogen bond between HIS584 and ligand at 3.69 Å with an angle of 44.12°, both indicating directional bonding. Electrostatic interactions include a Pi-Cation contact between ARG585 and ligand at 4.11 Å, and a Pi-Anion interaction between GLU866 and ligand at 4.50 Å, reflecting charge-based stabilization. Hydrophobic interactions involve a Pi-Sigma contact between ligand:C34 and ligand at 3.70 Å, and two Pi-Alkyl interactions between ligand and ARG5 at 4.81 Å and 5.35 Å, respectively, suggesting spatially favorable non-polar contacts. Additionally, a Pi-Sulfur interaction is observed between ligand: S7 and HIS584 at 5.20 Å, categorized as others highlighting the role of sulfur in ligand binding. These varied interactions ranging in distance from 3.13 Å to 5.35 Å demonstrate ligand’s multifaceted binding behavior, combining directional hydrogen bonds, electrostatic attractions, and hydrophobic contacts. Its binding affinity is -7.7 kcal/mol.


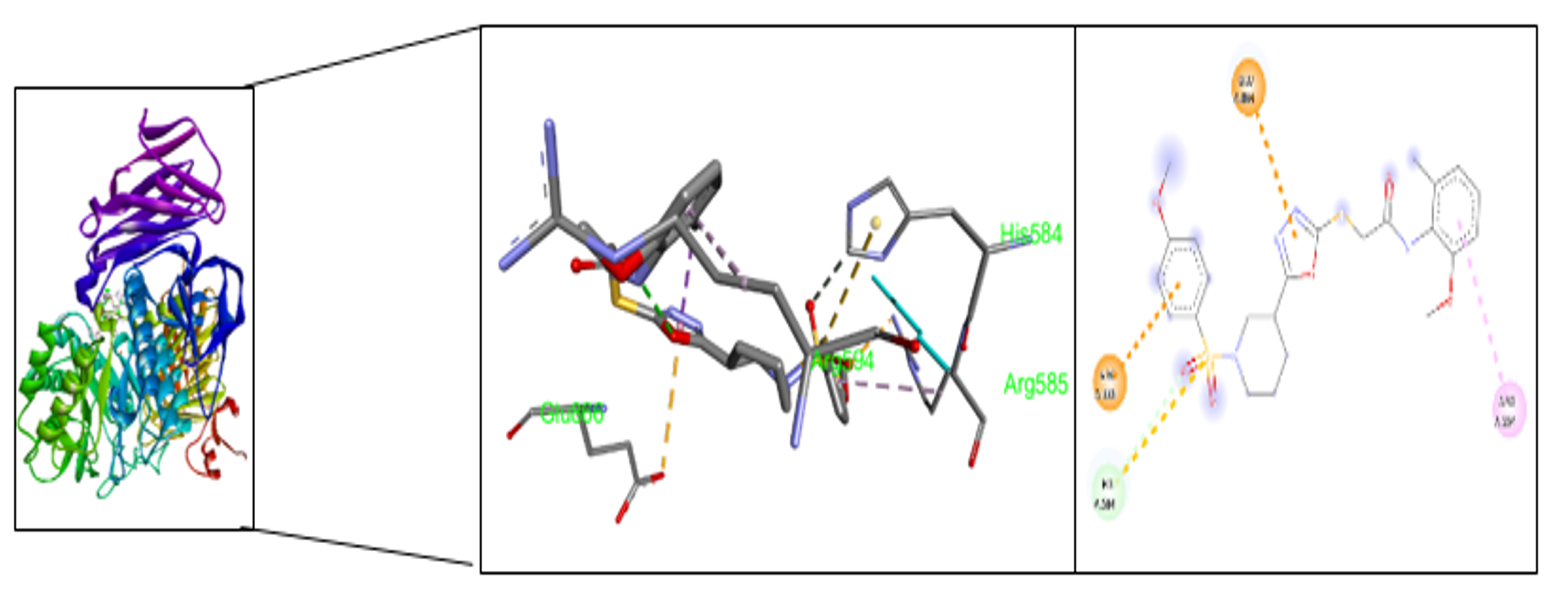


**Figure S81: Docking generated complex compound** **7m alpha glucosidase**

Synthesized compounds such as **7o** showed maximum alpha glucosidase inhibition activity so they are docked with alpha glucosidase confirm different interaction and binding capabilities making it more potent against alpha glucosidase (Figure S89). Many Hydrogen Bond interactions is observed with A: MET363, A: ARG60, A: GLU869, A\:PRO59, at the bond distance of 3.39 Aͦ, 3.37 Aͦ, 3.39 Aͦ, 3.37 Aͦ, 3.70 Aͦ respectively. A pi-sulfur interaction is noted at the bond distance of 5.38 Aͦ. Different Hydrophobic interactions also observed Pi-Pi Stacked with A: TYR360, Pi-Pi T-Shaped with A: HIS584, Alkyl with A: VAL867, three pi-alkyl with A: HIS584, A: HIS717, at the bond distance of 4.37 Aͦ, 4.58 Aͦ, 5.23 Aͦ, 5.02 Aͦ, 5.06 Aͦ and 4.75Aͦ respectively. It shows binding affinity of -7.3 Kcal/mol.


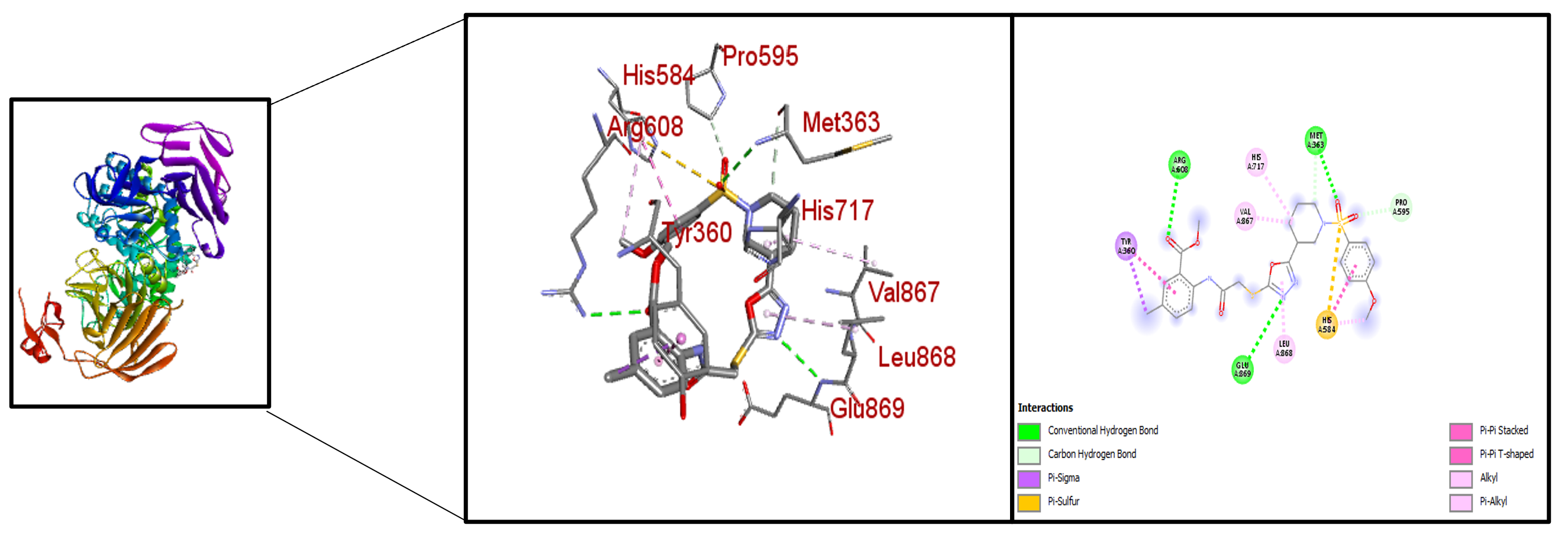


**Figure S82: Docking-generated complexes of compound** **7o alpha glucosidase**


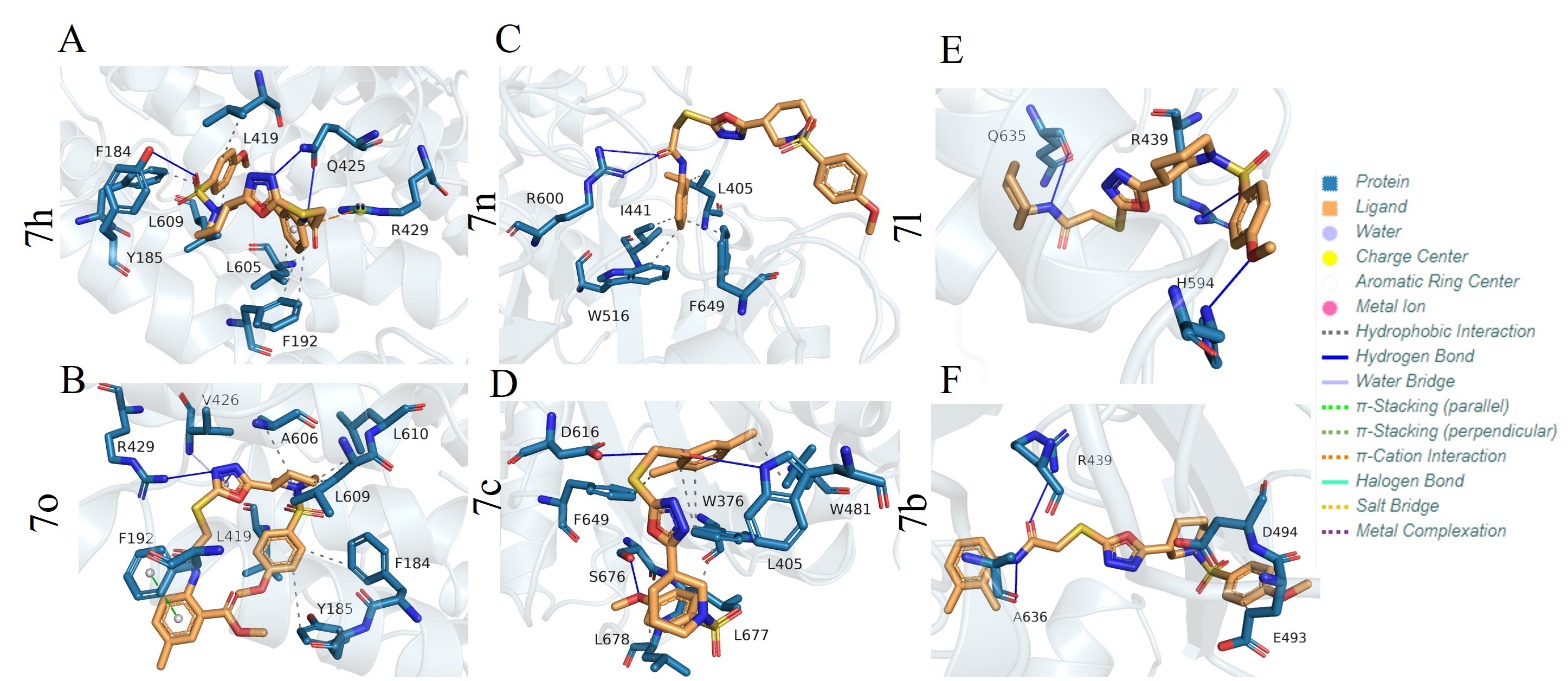


**Figure S83.** PLIP-generated interaction diagrams illustrating residue-level binding profiles of the synthesized compounds within LOX, α-glucosidase, and urease. Panels A and B show the LOX interactions of compounds 7h and 7o, respectively, where 7h forms well-defined hydrogen bonds and hydrophobic contacts consistent with strong inhibition, while 7o displays weaker and poorly oriented interactions. Panels C and D present the α-glucosidase complexes of compounds 7n and 7c, highlighting the extensive hydrogen bonding network of 7n with D518, D616, and R600 in contrast to the limited catalytic engagement observed for 7c. Panels E and F illustrate the urease binding modes of compounds 7l and 7b, demonstrating the strong interaction network of 7l with residues R439, Q635, A636, and H593, compared with the reduced and scattered contacts seen for 7b. Interaction types include hydrogen bonds, hydrophobic contacts, π-stacking, water bridges, salt bridges, halogen contacts, and metal-related interactions as defined in the key.

**Table S1.** **Docking scores of compounds 7a-7o in terms of binding affinity**

| **Species** | **LIPOXYGENASE** Kcal/mol | **UREASE** Kcal/mol | **ALPHA GLUCOSIDASE**  Kcal/mol |
| --- | --- | --- | --- |
| **7a** | -7.4 | -7.5 | **-**7.4 |
| **7b** | -8.1 | -7.1 | **-**8.0 |
| **7c** | -8.2 | -7.2 | -7.1 |
| **7d** | -8.0 | -7.5 | **-**7.8 |
| **7e** | -8.1 | -7.4 | **-**7.1 |
| **7f** | -6.1 | -7.5 | **-**7.6 |
| **7g** | -8.2 | -7.2 | **-**7.4 |
| **7h** | -8.3 | -6.8 | -6.4 |
| **7i** | -8.0 | -7.6 | **-**7.8 |
| **7j** | -7.9 | -7.0 | **-**7.9 |
| **7k** | -7.1 | -7.2 | -6.9 |
| **7l** | -7.4 | -7.6 | -7.2 |
| **7m** | -7.2 | -6.7 | **-**7.7 |
| **7n** | -8.1 | -7.6 | -8.1 |
| **7o** | -7.9 | -7.6 | **-**7.3 |
| **Quercitin** | -6.486 | - | **-** |
| **Thiourea** | - | -3.214 | **-** |
| **Acarbose** | - | - | -5.671 |

**Table S2. B3LYP/6-311G(d,p) results for the compounds 7a-7o, gas phase//DMSO.**

| **Species** | **E_0_, A.U.** | **E_0_+ZPE, A.U.** | **E(HOMO/**  **LUMO), A.U.** | **ΔE(H/L),**  **eV** |
| --- | --- | --- | --- | --- |
| **7a** | -2323.272637//  -2323.301800  3i^a^ | -2322.773081//  -2322.802633 | -0.22940/ -0.03547//  -0.22752/ -0.03827 | 5.28//  5.15 |
| **7b** | -2323.269802//  -2323.299160 | -2322.769662//  -2322.799424 | -0.23501/ -0.03570//  -0.23357/ -0.03863 | 5.42//  5.30 |
| **7c** | -2323.273342//  -2323.302216 | -2322.773818//  -2322.802929 | -0.23267/ -0.03582//  -0.23074/ -0.03880 | 5.36//  5.22 |
| **7d** | -2323.269870  3i//  -2323.299893 | -2322.770328//  -2322.800480 | -0.23820/ -0.03547//  -0.24062/ -0.03860 | 5.52//  5.50 |
| **7e** | -2323.273733//  -2323.303767 | -2322.774886//  -2322.804867 | -0.23333/ -0.03536//  -0.23191/ -0.03838 | 5.39//  5.27 |
| **7f** | -2244.618769//  -2244.648539 | -2244.173960//  -2244.203883 | -0.23887/ -0.03686//  -0.23723/ -0.03844 | 5.50//  5.41 |
| **7g** | -2283.944813  11i//  -2283.974255 | -2283.472370//  -2283.501961 | -0.23777/ -0.03772//  -0.23523/ -0.03850 | 5.44//  5.35 |
| **7h** | -2283.946442//  -2283.975999 | -2283.474347//  -2283.504247 | -0.23755/ -0.03607//  -0.23516/ -0.03844 | 5.48//  5.35 |
| **7i** | -2323.273286//  -2323.302938 | -2322.773736//  -2322.803463 | -0.22939/ -0.03536//  -0.22773/ -0.03847 | 5.28//  5.15 |
| **7j** | -2398.500294//  -2398.532333 | -2397.995376//  -2398.027419 | -0.21885/ -0.03597//  -0.21739/ -0.03837 | 4.98//  4.87 |
| **7k** | -2362.590895//  -2362.621790 | -2362.062666//  -2362.093921 | -0.23842/ -0.03649//  -0.24061/ -0.03910 | 5.49//  5.48 |
| **7l** | -2248.254140//  -2248.282734 | -2247.739887//  -2247.768638 | -0.23712/ -0.03544//  -0.24042/ -0.03880 | 5.49//  5.49 |
| **7m** | -2359.168513//  -2359.199840 | -2358.691947//  -2358.723278 | -0.23753/ -0.03709//  -0.23300/ -0.03854 | 5.45//  5.29 |
| **7n** | -2323.265624//  -2323.295707 | -2322.764725//  -2322.795065 | -0.23815/ -0.03601//  -0.23711/ -0.03860 | 5.50//  5.40 |
| **7o** | -2511.885888//  -2511.920515 | -2511.371498//  -2511.406015 | -0.23685/ -0.06690//  -0.23712/ -0.06598 | 4.62//  4.66 |

^a^These numbers correspond to small imaginary frequencies which cannot be eliminated by further optimization and do not affect the energy of the compound.
