# Supplementary material for: Structural and computational supported development of 2,5-disubstituted-1,3,4-oxadiazole analogues as active LOX, urease, and α-glucosidase inhibitors
Source: Sci Rep. 2026 Jan 21;16:5866. doi: 10.1038/s41598-026-35499-1 (PMC12895000; doi:10.1038/s41598-026-35499-1)
Supplement: Supplementary file 1 — Supplementary Material 1 [file 41598_2026_35499_MOESM1_ESM.docx]

**Structure based development of 2,5-disubstituted-1,3,4-oxadizole analogues as an active enzyme inhibitor**

Jamila Javid^a^, Aziz-ur-Rehman^a^*****, Javed Iqbal^b^******, Nadia Bhatti^b^, Aleksey Kuznetsov^c^, Fatiqa Zafar^b^, Muhammad Adnan Ayub^b^, Osama A. Mohammed^d^, Samiah H. Al-Mijalli^e^, Munawar Iqbal^f^, Syed A. Ali Shah^g,^

***N*-(2,4-dimethylphenyl)-2-((5-(1-((4-methoxyphenyl)sulfonyl)piperidin-3-yl)-1,3,4-oxadiazol-2-yl)thio)acetamide (7a)**

Light orange solid; yield: 82%; m.p.: 185-187 ºC; molecular formula: C_24_H_28_N_4_O_5_S_2_; molecular weight: 516.63 g mol^-1^; 1348 (-SO_2_ str.), 1515 (Ar C=C str.), 1572 (C=N str.), 3044 (Ar-H), 1234, 1076 (C-O-C stretching), 3346 (N-H stretching), 1666 (C=O stretching); ^1^H-NMR (600 MHz, CDCl_3_, *δ* / ppm): 7.72 (d, *J* = 8.9 Hz, 2H, H-2'' & H-6''), 7.67 (d, *J* = 8.2 Hz, 1H, H-6'''), 7.07 (d, *J* = 8.3 Hz, 1H, H-5'''), 7.04 (s, 1H, H-3'''), 7.01 (d, *J* = 8.9 Hz, 2H, H-3'' & H-5''), 4.05 (s, 2H, H -1''''), 4.01-3.99 (m, 1H, H*e*-2'), 3.89 (s, 3H, H-7''), 3.71-3.69 (m, 1H, Ha-2'), 3.14-3.09 (m, 1H, H-3'), 2.63-2.59 (m, 1H, H*e*-6'), 2.44-2.40 (m, 1H, H*a*-6'), 2.32 (s, 3H, H-8'''), 2.31 (s, 3H, H-7'''), 2.16-2.13 (m, 1H, H*a*-5'), 1.92-1.89 (m,2H, H*a*-4'), 1.80-1.72 (m, H, H*e-*5'), 1.64-1.57 (m, 1H, H*e*-4). ^13^C-NMR (150 MHz, CDCl_3_, *δ* / ppm): 163.11 (C-1''''), 161.56 (C-5), 160.83 (C-2), 160.43 (C-4''), 133.65 (C-4'''), 133.29 (C-2'''), 131.38 (C-1''), 129.74 (C-2'' & C-6''), 127.69 (C-1'''), 126.97 (C-3'''), 126.22 (C-5'''), 119.66 (C-6'''), 114.34 (C-3'' & C-5''), 55.61 (C-7''), 52.83 (C-2'), 48.61 (C-6'), 46.27 (C-2''''), 33.60 (C-3'), 27.28 (C-4'), 23.87 (C-5'), 20.71 (C-8'''), 17.63 (C-7''').

**^1^H and ^13^C-NMR SPECTRA OF ALL SYNTHESIZED COMPOUNDS**

**H-NMR of compound 7a**

**C-NMR of compound 7a**

***N*-(2,3-dimethylphenyl)-2-((5-(1-((4-methoxyphenyl)sulfonyl)piperidin-3-yl)-1,3,4-oxadiazol-2-yl)thio)acetamide (6b)**

Light pink solid; yield: 87%; m.p.: 174-176 ºC; molecular formula: C_24_H_28_N_4_O_5_S_2_; molecular weight: 516.63 g mol^-1^; IR (KBr, *v*_max_ cm^−1^): 1350 (-SO_2_ str.), 1513 (Ar C=C str.), 1573 (C=N str.), 1238, 1078 (C-O-C stretching), 3342 (N-H stretching), 1664 (C=O stretching), 3046 (Ar-H); ^1^H-NMR (600 MHz, CDCl_3_, *δ* / ppm): 7.07 (s, 1H, NH), 7.72 (d, *J* = 8.9 Hz, 2H, H-2'' & H-6''), 7.60 (d, *J* = 8.0 Hz, 1H, H-6'''), 7.16 (t, *J* = 7.9 Hz, 1H, H-5'''), 7.01 (d, *J* = 8.9 Hz, 2H, H-3'' & H-5''), 7.00 (d, *J* = 7.6 Hz, 1H, H-4'''), 4.05 (s, 2H, H -1''''), 4.02-3.99 (m, 1H, H*e*-2'), 3.89 (s, 3H, H-7''), 3.72-3.70 (m, 1H, H*a*-2'), 3.15-3.10 (m, 1H, H-3'), 2.63-2.59 (m, 1H, H*e*-6'), 2.44-2.40 (m, 1H, H*a*-6'), 2.35 (s, 3H, H-8'''), 2.25 (s, 3H, H-7'''), 2.16-2.13 (m, 1H, H*a*-5'), 1.92-1.89 (m, 2H, H*a*-4'), 1.78-1.73 (m, 1H, H*e-*5'), 1.64-1.59 (m, 1H, H*e*-4'). ^13^C-NMR (150 MHz,CDCl_3_, *δ* / ppm): 163.11 (C-1''''), 161.00 (C-5), 160.83 (C-2), 160.54 (C-4''), 137.61 (C-1'''), 135.74 (C-3'''), 131.38 (C-1''), 129.74 (C-2'' & C-6''), 127.70 (C-2'''), 126.34 (C-4'''), 126.25 (C-5'''), 118.05 (C-6'''), 114.34 (C-3'' & C-5''), 55.61 (C-7''), 52.83 (C-2'), 48.61 (C-6'), 46.27 (C-2''''), 33.62 (C-3'), 27.30 (C-4'), 23.88 (C-5'), 20.65 (C-8'''), 13.42 (C-7''').

**H-NMR of compound 7b**

**C-NMR of compound 7b**

***N*-(2,5-dimethylphenyl)-2-((5-(1-((4-methoxyphenyl)sulfonyl)piperidin-3-yl)-1,3,4-oxadiazol-2-yl)thio)acetamide (6c)**

Off white solid; yield: 86%; m.p.: 171-173 ºC; molecular formula: C_24_H_28_N_4_O_5_S_2_; molecular weight: 516.63 g mol^-1^; IR (KBr, *v*_max_ cm^−1^): 1346 (-SO_2_ str.), 1516 (Ar C=C str.), 1569 (C=N str.), 1232, 1074 (C-O-C stretching), 3349 (N-H stretching), 1670 (C=O stretching), 3047 (Ar-H); ^1^H-NMR (600 MHz, CDCl_3_, *δ* / ppm): 7.75 (s, 1H, H-6'''), 7.73 (d, *J* = 8.9 Hz, 2H, H-2'' & H-6''), 7.09 (d, *J* = 7.6 Hz, 1H, H-3'''), 7.02 (d, *J* = 8.9 Hz, 2H, H-3'' & H-5''), 6.87 (d, *J* = 7.5 Hz, 1H, H-4'''), 4.05 (s, 2H, H -1''''), 3.89 (s, 3H, H-7''), 4.03-4.00 (m, 1H, H*e*-2'), 3.72-3.70 (m, 1H, H*a*-2'), 3.16-3.13 (m, 1H, H-3'), 2.65-2.61 (m, 1H, H*e*-6'), 2.46-2.42 (m, 1H, H*a*-6'), 2.37 (s, 3H, H-8'''), 2.30 (s, 3H, H-7'''), 2.18-2.15 (m, 1H, H*a*-5'), 1.94-1.91 (m, 2H, H*a*-4'), 1.80-1.77 (m, 1H, H*e-*5'), 1.66-1.60 (m, 1H, H*e*-4'). ^13^C-NMR (150 MHz, CDCl_3_, *δ* / ppm): 163.12 (C-1''''), 161.00 (C-5), 160.52 (C-2), 160.37 (C-4''), 137.24 (C-1'''), 135.54 (C-5'''), 131.38 (C-1''), 129.74 (C-2'' & C-6''), 127.70 (C-3'''), 124.44 (C-2'''), 122.86 (C-4'''), 119.41 (C-6'''), 114.34 (C-3'' & C-5''), 55.61 (C-7''), 52.83 (C-2'), 48.62 (C-6'), 46.28 (C-2''''), 33.58 (C-3'), 27.25 (C-4'), 23.86 (C-5'), 21.29 (C-8'''), 17.14 (C-7''').

**H-NMR of compound 7c**

**C-NMR of compound 7c**

***N*-(2,6-dimethylphenyl)-2-((5-(1-((4-methoxyphenyl)sulfonyl)piperidin-3-yl)-1,3,4-oxadiazol-2-yl)thio)acetamide (6d)**

Pink white solid; yield: 83%; m.p.: 196-198 ºC; molecular formula: C_24_H_28_N_4_O_5_S_2_; molecular weight: 516.63 g mol^-1^; IR (KBr, *v*_max_ cm^−1^): 1342 (-SO_2_ str.), 1520 (Ar C=C str.), 1565 (C=N str.), 1239, 1077 (C-O-C stretching), 3348 (N-H stretching), 1665 (C=O stretching), 3050 (Ar-H); ^1^H-NMR (600 MHz, CDCl_3_, *δ* / ppm): 8.01 (s, 1H, NH), 7.69 (d, *J* = 8.9 Hz, 2H, H-2'' & H-6''), 7.19 (d, *J* = 8.6 Hz, 2H, H-3''' & H-5'''), 7.17 (t, *J* = 8.4 Hz, 1H, H-4'''), 7.01 (d, *J* = 8.9 Hz, 2H, H-3'' & H-5''), 6.87 (d, *J* = 7.5 Hz, 1H, H-4'''), 4.05 (s, 2H, H -1''''), 4.03-4.00 (m, 1H, H*e*-2'), 3.89 (s, 3H, H-7''), 3.72-3.70 (m, 1H, H*a*-2'), 3.16-3.13 (m, 1H, H-3'), 2.65-2.61 (m, 1H, H*e*-6'), 2.46-2.42 (m, 1H, H*a*-6'), 2.24 (s, 3H, H-8'''), 2.23 (s, 3H, H-7'''), 2.18-2.15 (m, 1H, H*a*-5'), 1.94-1.91 (m, 2H, H*a*-4'), 1.80-1.77 (m, 1H, H*e-*5'), 1.66-1.60 (m, 1H, H*e*-4'). ^13^C-NMR (150 MHz, CDCl_3_, *δ* / ppm): 163.12 (C-1''''), 161.00 (C-5), 160.52 (C-2), 160.37 (C-4''), 136.07 (C-1'''), 135.95 (C-2'''), 135.25 (C-6'''), 132.29 (C-1''), 129.74 (C-2'' & C-6''), 128.75 (C-3'''), 128.33 (C-5'''), 127.50 (C-4'''), 114.36 (C-3'' & C-5''), 55.64 (C-7''), 52.83 (C-2'), 48.06 (C-6'), 46.43 (C-2''''), 33.16 (C-3'), 26.86 (C-4'), 23.53 (C-5'), 17.69 (C-7''' & C-8''').

**H-NMR of compound 7d**

**C-NMR of compound 7d**

***N*-(3,5-dimethylphenyl)-2-((5-(1-((4-methoxyphenyl)sulfonyl)piperidin-3-yl)-1,3,4-oxadiazol-2-yl)thio)acetamide (6e)**

Off white solid; yield: 85%; m.p.: 178-180 ºC; molecular formula: C_24_H_28_N_4_O_5_S_2_; molecular weight: 516.63 g mol^-1^; IR (KBr, *v*_max_ cm^−1^): 1346 (-SO_2_ str.), 1512 (Ar C=C str.), 1561 (C=N str.), 1242, 1077 (C-O-C stretching), 3347 (N-H stretching), 1660 (C=O stretching), 3058 (Ar-H); ^1^H-NMR (600 MHz, CDCl_3_, *δ* / ppm): 7.72(d, *J* = 8.9 Hz, 2H, H-2'' & H-6''), 7.10 (s, 2H, H-2''' & H-6'''), 7.01 (d, *J* = 8.9 Hz, 2H, H-3'' & H-5''), 6.74 (s,1H, H-4'''), 4.05 (s, 2H, H -1''''), 3.89 (s, 3H, H-7''), 3.99-3.97 (m, 1H, H*e*-2'), 3.69-3.67 (m, 1H, H*a*-2'), 3.16-3.12 (m, 1H, H-3'), 2.67-2.63 (m, 1H, H*e*-6'), 2.48-2.41 (m, 1H, H*a*-6'), 2.34 (s, 3H, H-8'''), 2.30 (s, 3H, H-7'''), 2.16-2.13 (m, 1H, H*a*-5'), 1.94-1.90 (m, 2H, H*a*-4'), 1.78-1.76 (m, 1H, H*e-*5'), 1.67-1.62 (m, 1H, H*e*-4'). ^13^C-NMR (150 MHz, CDCl_3_, *δ* / ppm): 163.13 (C-1''''), 161.00 (C-5), 160.35 (C-2), 160.04 (C-4''), 139.18 (C-3'''), 137.38 (C-5'''), 132.29 (C-1''), 129.74 (C-2'' & C-6''), 127.64 (C-1'''), 124.94 (C-4'''), 115.35 (C-2''' & C-6'''), 114.35 (C-3'' & C-5''), 55.61 (C-7''), 52.83 (C-2'), 48.62 (C-6'), 46.29 (C-2''''), 33.50 (C-3'), 27.20 (C-4'), 23.81 (C-5'), 21.42 (C-7''' & C-8''').

**H-NMR of compound 7e**

**C-NMR of compound 7e**

**2-((5-(1-((4-methoxyphenyl)sulfonyl)piperidin-3-yl)-1,3,4-oxadiazol-2-yl)thio)-*N*-phenylacetamide (6f)**

Orange white solid; yield: 87%; m.p.: 193-195 ºC; molecular formula: C_22_H_24_N_4_O_5_S_2_; molecular weight: 488.58 g mol^-1^; IR (KBr, *v*_max_ cm^−1^): 1347 (-SO_2_ str.), 1513 (Ar C=C str.), 1562 (C=N str.), 1244, 1070 (C-O-C stretching), 3339 (N-H stretching), 1659 (C=O stretching), 3054 (Ar-H); ^1^H-NMR (600 MHz, CDCl_3_, *δ* / ppm): 7.72 (d, *J* = 8.9 Hz, 2H, H-2'' & H-6''), 7.10 (s, 2H, H-2''' & H-6'''), 7.01 (d, *J* = 8.9 Hz, 2H, H-3'' & H-5''), 6.74 (s,1H, H-4'''), 4.05 (s, 2H, H -1''''), 3.89 (s, 3H, H-7''), 3.99-3.97 (m, 1H, H*e*-2'), 3.69-3.67 (m, 1H, H*a*-2'), 3.16-3.12 (m, 1H, H-3'), 2.67-2.63 (m, 1H, H*e*-6'), 2.48-2.41 (m, 1H, H*a*-6'), 2.34 (s, 3H, H-8'''), 2.30 (s, 3H, H-7'''), 2.16-2.13 (m, 1H, H*a*-5'), 1.94-1.90 (m,2H, H*a*-4'), 1.78-1.76 (m, 1H, H*e-*5'), 1.67-1.62 (m, 1H, H*e*-4'). ^13^C-NMR (150 MHz, CDCl_3_, *δ* / ppm): 163.14 (C-1''''), 161.00 (C-5), 160.44 (C-2), 160.07 (C-4''), 137.61 (C-1'''), 132.29 (C-1''), 129.74 (C-2'' & C-6''), 129.38 (C-3''' & C-5'''), 123.08 (C-4'''), 117.54 (C-2''' & C-6'''), 114.36 (C-3'' & C-5''), 55.62 (C-7''), 52.83 (C-2'), 48.61 (C-6'), 46.29 (C-2''''), 33.53 (C-3'), 27.20 (C-4'), 23.79 (C-5').

**H-NMR of compound 7f**

**C-NMR of compound 7f**

**2-((5-(1-((4-methoxyphenyl)sulfonyl)piperidin-3-yl)-1,3,4-oxadiazol-2-yl)thio)-*N*-(o-tolyl)acetamide (6g)**

Light peach solid; yield: 85%; m.p.: 114-116 ºC; molecular formula: C_23_H_26_N_4_O_5_S_2_; molecular weight: 502.61 g mol^-1^; IR (KBr, *v*_max_ cm^−1^): 1343 (-SO_2_ str.), 1514 (Ar C=C str.), 1564 (C=N str.), 1242, 1077 (C-O-C stretching), 3343 (N-H stretching), 1658 (C=O stretching), 3056 (Ar-H); ^1^H-NMR (600 MHz, CDCl_3_, *δ* / ppm): 7.89 (d, *J* = 8.0 Hz, 1H, H-6'''), 7.73 (d, *J* = 11.7 Hz, 2H, H-2'' & H-6''), 7.22 (d, *J* = 7.3 Hz, 1H, H-3'''), 7.06 (t, *J* = 7.4 Hz, 1H, H-5''' ), 7.02 (d, *J* = 11.9 Hz, 2H, H-3'' & H-5''), 7.00- 6.98 (m,1H, H-4'''), 4.07-4.00 (m, 1H, H*e*-2'), 4.05 (s, 2H, H -1''''), 3.89 (s, 3H, H-7''), 3.71 -3.69 (m, 1H, H*a*-2'), 3.16 -3.13 (m, 1H, H-3'), 2.66-2.62 (m, 1H, H*e*-6'), 2.47-2.43 (m, 1H, H*a*-6'), 2.35 (s, 3H, H-7'''), 2.18-2.15 (m, 1H, H*a*-5'), 1.93-1.91 (m,2H, H*a*-4'), 1.79-1.76 (m, 1H, H*e-*5'), 1.68-1.62 (m, 1H, H*e*-4'). ^13^C-NMR (150 MHz, CDCl_3_, *δ* / ppm): 163.13 (C-1''''), 161.00 (C-5), 160.57 (C-2), 160.40 (C-4''), 135.81 (C-1'''), 132.29 (C-1''), 129.74 (C-2'' & C-6''), 127.68 (C-2'''), 127.35 (C-3'''), 126.03 (C-4'''), 123.71 (C-5'''), 118.79 (C-6'''), 114.34 (C-3'' & C-5''), 55.64 (C-7''), 52.83 (C-2'), 48.61 (C-6'), 46.28 (C-2''''), 33.59 (C-3'), 27.25 (C-4'), 23.85 (C-5'), 17.60 (C-7''').

**H-NMR of compound 7g**

**C-NMR of compound 7g**

**2-((5-(1-((4-methoxyphenyl)sulfonyl)piperidin-3-yl)-1,3,4-oxadiazol-2-yl)thio)-*N*-(m-tolyl)acetamide (6h)**

Light grey solid; yield: 87%; m.p.: 170-172 ºC; molecular formula: C_23_H_26_N_4_O_5_S_2_; molecular weight: 502.61 g mol^-1^; IR (KBr, *v*_max_ cm^−1^): 1354 (-SO_2_ str.), 1522 (Ar C=C str.), 1563 (C=N str.), 1246, 1077 (C-O-C stretching), 3346 (N-H stretching), 1663 (C=O stretching), 3057 (Ar-H); ^1^H-NMR (600 MHz, CDCl_3_, *δ* / ppm): 7.72 (d, *J* = 8.9 Hz, 2H, H-2'' & H-6''), 7.44 (s, 1H, NH), 7.33 (s, 1H, H-2'''), 7.27-7.24 (m, 2H, H-5''' & H-6'''), 7.01 (d, *J* = 8.9 Hz, 2H, H-3'' & H-5''), 6.91 (d, *J* = 5.6 Hz, 1H, H-4'''), 4.00-3.98 (m, 1H, H*e*-2'), 4.05 (s, 2H, H -1''''), 3.89 (s, 3H, H-7''), 3.70 -3.68 (m, 1H, H*a*-2'), 3.17 -3.12 (m, 1H, H-3'), 2.67-2.63 (m, 1H, H*e*-6'), 2.48-2.44 (m, 1H, H*a*-6'), 2.38 (s, 3H, H-7'''), 2.17-2.14 (m, 1H, H*a*-5'), 1.94-1.90 (m,2H, H*a*-4'), 1.80 -1.74 (m, 1H, H*e-*5'), 1.68-1.62 (m, 1H, H*e*-4'). ^13^C-NMR (150 MHz, CDCl_3_, *δ* / ppm): 163.14 (C-1''''), 161.00 (C-5), 160.39 (C-2), 160.06 (C-4''), 139.41 (C-3'''), 137.51 (C-1'''), 132.29 (C-1''), 129.74 (C-2'' & C-6''), 129.19 (C-5'''), 127.63 (C-4'''), 123.95 (C-2'''), 118.19 (C-6'''), 114.66 (C-3'' & C-5''), 55.62 (C-7''), 52.83 (C-2'), 48.62 (C-6'), 46.29 (C-2''''), 33.53 (C-3'), 27.20 (C-4'), 23.81 (C-5'), 21.54 (C-7''').

**H-NMR of compound 7h**

**C-NMR of compound 7h**

***N*-(2-ethylphenyl)-2-((5-(1-((4-methoxyphenyl)sulfonyl)piperidin-3-yl)-1,3,4-oxadiazol-2-yl)thio)acetamide (6i)**

Peach solid; yield: 82%; m.p.: 116-118 ºC; molecular formula: C_24_H_28_N_4_O_5_S_2_; molecular weight: 516.63 g mol^-1^; IR (KBr, *v*_max_ cm^−1^): 1358 (-SO_2_ str.), 1528 (Ar C=C str.), 1568 (C=N str.), 1228, 1071 (C-O-C stretching), 3344 (N-H stretching), 1668 (C=O stretching), 3058 (Ar-H); ^1^H-NMR (600 MHz, CDCl_3_, *δ* / ppm): 7.87 (d, *J* = 8.0 Hz, 1H, H-6'''), 7.72 (d, *J* = 8.9 Hz, 2H, H-2'' & H-6''), 7.30-7.24 (m, 2H, H-3''' & H-5'''), 7.11 (t, *J* = 7.4 Hz, 1H, H-4'''), 7.01 (d, *J* = 8.9 Hz, 2H, H-3'' & H-5''), 6.92 (s, 1H, NH), 4.05 (s, 2H, H -1''''), 4.02-4.00 (m, 1H, H*e*-2'), 3.89 (s, 3H, H-7''), 3.72 -3.70 (m, 1H, H*a*-2'), 3.16 -3.11 (m, 1H, H-3'), 2.68 (q, 2H, H-7'''), 2.65 -2.61 (m, 1H, H*e*-6'), 2.45-2.41 (m, 1H, H*a*-6'), 2.17-2.14 (m, 1H, H*a*-5'), 1.93-1.90 (m,2H, H*a*-4'), 1.80 -1.73 (m, 1H, H*e-*5'), 1.65-1.60 (m, 1H, H*e*-4'), 1.30 (t, *J* = 7.6 Hz, 3H, H-8'''). ^13^C-NMR (150 MHz, CDCl_3_, *δ* / ppm): 163.12 (C-1''''), 161.00 (C-5), 160.64 (C-2), 160.57 (C-4''), 135.17 (C-1'''), 132.29 (C-1''), 129.74 (C-2'' & C-6''), 128.69 (C-2'''), 127.68 (C-5'''), 127.19 (C-4'''), 124.12 (C-3'''), 119.48 (C-6'''), 114.37 (C-3'' & C-5''), 55.62 (C-7''), 52.63 (C-2'), 48.61 (C-6'), 46.28 (C-2''''), 33.61 (C-3'), 27.26 (C-4'), 24.07 (C-7'''), 23.86 (C-5'), 13.76 (C-8''').

**H-NMR of compound 7i**

**C-NMR of compound 7i**

***N*-(4-ethoxyphenyl)-2-((5-(1-((4-methoxyphenyl)sulfonyl)piperidin-3-yl)-1,3,4-oxadiazol-2-yl)thio)acetamide (6j)**

Purple grey solid; yield: 83%; m.p.: 150-152 ºC; molecular formula: C_24_H_28_N_4_O_6_S_2_; molecular weight: 532.63 g mol^-1^; IR (KBr, *v*_max_ cm^−1^): 1351 (-SO_2_ str.), 1514 (Ar C=C str.), 1566 (C=N str.), 1239, 1077 (C-O-C stretching), 3345 (N-H stretching), 1667 (C=O stretching), 3060 (Ar-H); ^1^H-NMR (600 MHz, CDCl_3_, *δ* / ppm): 7.71 (d, *J* = 8.9 Hz, 2H, H-2'' & H-6''), 7.57 (s, 1H, NH), 7.37 (d, *J* = 8.8 Hz, 2H, H-3''' & H-5'''), 7.01 (d, *J* = 8.9 Hz, 2H, H-3'' & H-5''), 6.90 (d, 2H, H-2''' & H-6'''), 4.05 (s, 2H, H -1''''), 4.03(q, 2H, H-7'''), 3.98-3.96 (m, 1H, H*e*-2'), 3.89 (s, 3H, H-7''), 3.68 -3.66 (m, 1H, H*a*-2'), 3.13 -3.10 (m, 1H, H-3'), 2.65 -2.62 (m, 1H, H*e*-6'), 2.47-2.43 (m, 1H, H*a*-6'), 2.15-2.12 (m, 1H, H*a*-5'), 1.92-1.89 (m, 2H, H*a*-4'), 1.79 -1.75 (m, 1H, H*e-*5'), 1.74 -1.61 (m, 1H, H*e*-4'), 1.42 (t, *J* = 13.9 Hz, 3H, H-8'''). ^13^C-NMR (150 MHz, CDCl_3_, *δ* / ppm): 163.13 (C-1''''), 161.00 (C-5), 160.61 (C-2), 160.22 (C-4''), 155.17 (C-4'''), 132.29 (C-1''), 129.73 (C-2'' & C-6''), 127.62 (C-1'''), 119.68 (C-2''' & C-6'''), 115.26 (C-3''' & C-5'''), 114.35 (C-3'' & C-5''), 63.81 (C-7'''), 55.62 (C-7''), 52.63 (C-2'), 48.63 (C-6'), 46.29 (C-2''''), 33.55 (C-3'), 27.21 (C-4'), 23.81 (C-5'), 14.85 (C-8''').

**H-NMR of compound 7j**

**C-NMR of compound 7j**

***N*-(2-ethyl-6-methylphenyl)-2-((5-(1-((4-methoxyphenyl)sulfonyl)piperidin-3-yl)-1,3,4-oxadiazol-2-yl)thio)acetamide (6k)**

Peach solid; yield: 84%; m.p.: 145-147 ºC; molecular formula: C_25_H_30_N_4_O_5_S_2_; molecular weight: 530.66 g mol^-1^;  IR (KBr, *v*_max_ cm^−1^): 1346 (-SO_2_ str.), 1512 (Ar C=C str.), 1561 (C=N str.), 1226, 1069 (C-O-C stretching), 3338 (N-H stretching), 1658 (C=O stretching), 3058 (Ar-H); ^1^H-NMR (600 MHz, CDCl_3_, *δ* / ppm): 7.71 (d, *J* = 8.9 Hz, 2H, H-2'' & H-6''), 7.22 (t, *J* = 7.6 Hz, 1H, H-4'''), 7.15 (dd, *J* = 9.60 Hz, 2H, H-3''' & H-5'''), 7.01 (d, *J* = 8.9 Hz, 2H, H-3'' & H-5''), 4.05 (s, 2H, H -1''''), 2.28 (s, 3H, H-7'''), 4.00-3.97 (m, 1H, H*e*-2'), 3.90 (s, 3H, H-7''), 3.72 -3.70 (m, 1H, H*a*-2'), 3.05 -3.01 (m, 1H, H-3'), 2.65 (q, 2H, H-8'''), 2.56 -2.53 (m, 1H, H*e*-6'), 2.37-2.33 (m, 1H, H*a*-6'), 2.13-2.08 (m, 1H, H*a*-5'), 1.87 -1.84 (m,2H, H*a*-4'), 1.73 -1.68 (m, 1H, H*e-*5'), 1.52 -1.49 (m, 1H, H*e*-4'), 1.20 (t, *J* = 7.6 Hz, 3H, H-9'''). ^13^C-NMR (150 MHz, CDCl_3_, *δ* / ppm): 163.09 (C-1''''), 161.00 (C-5), 160.70 (C-2), 160.22 (C-4''), 141.47 (C-1'''), 135.96 (C-2'''), 132.29 (C-1''), 129.73 (C-2'' & C-6''), 128.72 (C-6'''), 127.92 (C-5'''), 127.71 (C-4'''), 126.84 (C-3'''), 114.32 (C-3'' & C-5''), 55.62 (C-7''), 52.63 (C-2'), 48.42 (C-6'), 46.24 (C-2''''), 33.67 (C-3'), 27.42 (C-4'), 24.70 (C-8'''), 23.92 (C-5'), 18.33 (C-7'''), 14.61 (C-9''').

**H-NMR of compound 7k**

**C-NMR of compound 7k**

***N*-cyclohexyl-2-((5-(1-((4-methoxyphenyl)sulfonyl)piperidin-3-yl)-1,3,4-oxadiazol-2-yl)thio)acetamide (6l) (21)**

Off white solid; yield: 87%; m.p.: 154-156 ºC; molecular formula: C_22_H_30_N_4_O_5_S_2_; molecular weight: 494.63 g mol^-1^; IR (KBr, *v*_max_ cm^−1^): 1347 (-SO_2_ str.), 1513 (Ar C=C str.), 1562 (C=N str.), 1227, 1079 (C-O-C stretching), 3339 (N-H stretching), 1657 (C=O stretching), 3059 (Ar-H); ^1^H-NMR (600 MHz, CDCl_3_, *δ* / ppm): 7.71 (d, *J* = 8.9 Hz, 2H, H-2'' & H-6''), 7.01 (d, *J* = 8.9 Hz, 2H, H-3'' & H-5''), 4.05 (s, 2H, H -1''''), 4.00-3.97 (m, 1H, H*e*-2'), 3.89 (s, 3H, H-7''), 3.72 -3.70 (m, 1H, H*a*-2'), 3.05 -3.01 (m, 1H, H-3'), 2.56 -2.53 (m, 1H, H*e*-6'), 2.37-2.33 (m, 1H, H*a*-6'), 2.13-2.08 (m, 1H, H*a*-5'), 1.87 -1.84 (m,2H, H*a*-4'), 1.75-1.74 (m, 2H, H*e*-2''' & H*e*-6'''), 1.73 -1.68 (m, 1H, H*e-*5'), 1.52 -1.49 (m, 1H, H*e*-4'), 1.49-1.48 (m, 2H, H*a*-2''' & H*a*-6'''), 1.48-1.47 (m, 1H, H*e*-4'''), 1.42-1.36 (m, 1H, H*a*-4'''), 1.28-1.21 (m, 2H, H*e*-3''' & H*e*-5''' ), 1.11-1.10 (m, 2H, H*e*-3''' & H*e*-5'''). ^13^C-NMR (150 MHz, CDCl_3_, *δ* / ppm): 163.09 (C-1''''), 161.00 (C-5), 160.70 (C-2), 160.08 (C-4''), 132.29 (C-1''), 129.73 (C-2'' & C-6''), 114.32 (C-3'' & C-5''), 55.66 (C-7''), 52.53 (C-2'), 48.70 (C-6'), 48.09 (C-1'''), 46.28 (C-2''' & C-6'''), 46.24 (C-2''''), 33.64 (C-3'), 27.42 (C-4'), 25.92 (C-4'''), 24.97 (C-3''' & C-5'''), 23.31 (C-5').

**H-NMR of compound 7l**

**C-NMR of compound 7l**

***N*-(2-methoxyphenyl)-2-((5-(1-((4-methoxyphenyl)sulfonyl)piperidin-3-yl)-1,3,4-oxadiazol-2-yl)thio)acetamide (6m)**

Brown solid; yield: 82%; m.p.: 159-161 ºC; molecular formula: C_23_H_26_N_4_O_6_S_2_; molecular weight: 518.61 g mol^-1^; IR (KBr, *v*_max_ cm^−1^): 1342 (-SO_2_ str.), 1508 (Ar C=C str.), 1557 (C=N str.), 1237, 1076 (C-O-C stretching), 3347 (N-H stretching), 1668 (C=O stretching), 3054 (Ar-H); ^1^H-NMR (600 MHz, CDCl_3_, *δ* / ppm): 8.12 (d, *J* = 9.5 Hz, 1H, H-6'''), 7.73 (d, *J* = 8.9 Hz, 2H, H-2'' & H-6''), 6.93-6.91 (m, 1H, H-5'''), 7.05-7.00 (m, 2H, H-3''' & H-4'''), 7.01 (d, *J* = 11.7Hz, 2H, H-3'' & H-5''), 4.05 (s, 2H, H -1''''), 4.04-4.00 (m, 1H, H*e*-2'), 3.95 (s, 3H, H-7'''), 3.88 (s, 3H, H-7''), 3.73 -3.71 (m, 1H, H*a*-2'), 3.18 -3.13 (m, 1H, H-3'), 2.64 -2.60 (m, 1H, H*e*-6'), 2.46 -2.41 (m, 1H, H*a*-6'), 2.19-2.16 (m, 1H, H*a*-5'), 1.95 -1.90 (m, 2H, H*a*-4'), 1.82 -1.74 (m, 1H, H*e-*5'), 1.67 -1.59 (m, 1H, H*e*-4'). ^13^C-NMR (150 MHz, CDCl_3_, *δ* / ppm): 163.11 (C-1''''), 161.00 (C-5), 160.70 (C-2), 160.22 (C-4''), 146.96 (C-2'''), 132.29 (C-1''), 129.75 (C-2'' & C-6''), 127.68 (C-4'''), 127.15 (C-1'''), 121.36 (C-5'''), 116.98 (C-6'''), 114.37 (C-3'' & C-5''), 109.98 (C-3'''), 55.79 (C-7''), 52.63 (C-2'), 48.63 (C-6'), 46.28 (C-2''''), 33.55 (C-3'), 27.25 (C-4'), 23.88 (C-5').

**H-NMR of compound 7m**

**C-NMR of compound 7m**

***N*-benzyl-2-((5-(1-((4-methoxyphenyl)sulfonyl)piperidin-3-yl)-1,3,4-oxadiazol-2-yl)thio)acetamide (6n)**

Orange sticky; yield: 76%; m.p.: 174-176 ºC; molecular formula: C_23_H_26_N_4_O_5_S_2_; molecular weight: 502.61 g mol^-1^; IR (KBr, *v*_max_ cm^−1^): 1345 (-SO_2_ str.), 1511 (Ar C=C str.), 1560 (C=N str.), 1240, 1077 (C-O-C stretching), 3352 (N-H stretching), 1672 (C=O stretching), 3057 (Ar-H); ^1^H-NMR (600 MHz, CDCl_3_, *δ* / ppm): 7.62 (d, *J* = 9.2 Hz, 2H, H-2'' & H-6''), 6.93 (d, *J* = 8.6 Hz, 2H, H-3'' & H-5''), 4.05 (s, 2H, H -1''''), 3.92-3.91 (m, 1H, H*e*-2'), 3.91 (s, 3H, H-7''), 3.81 -3.77 (m, 1H, H*a*-2'), 3.61 -3.54 (m, 1H, H-3'), 3.01-2.99 (m, 1H, H*e*-6'), 2.82 (s, 3H, H-7'''), 2.63 -2.56 (m, 1H, H*a*-6'), 2.40 -2.36 (m, 1H, H*a*-5'), 1.79 -1.76 (m, 2H, H*a*-4'), 1.66 -1.59 (m, 1H, H*e-*5'), 1.24 -1.18 (m, 1H, H*e*-4'). ^13^C-NMR (150 MHz, CDCl_3_, *δ* / ppm): 163.18 (C-1''''), 161.00 (C-5), 160.70 (C-2), 160.22 (C-4''), 152.93 (C-1'''), 132.29 (C-1''), 129.74 (C-2'' & C-6''), 128.98 (C-2''' & C-6'''), 128.50 (C-3''' & C-5'''), 126.76 (C-4'''), 116.98 (C-6'''), 114.37 (C-3'' & C-5''), 55.64 (C-7''), 52.64 (C-2'), 48.41 (C-6'), 46.25 (C-7'''), 46.19 (C-2''''), 33.55 (C-3'), 27.48 (C-4'), 23.64 (C-5').

**H-NMR of compound 7n**

**C-NMR of compound 7n**

**2.5.15. Methyl 2-(2-((5-(1-((4-methoxyphenyl)sulfonyl)piperidin-3-yl)-1,3,4-oxadiazol-2-yl)thio)acetamido)-5-methylbenzoate (6o)**

Brown sticky; yield: 73%; m.p.: 170-172 ºC; molecular formula: C_25_H_28_N_4_O_7_S_2_; molecular weight: 560.64 g mol^-1^ , IR (KBr, *v*_max_ cm^−1^): 1341 (-SO_2_ str.), 1515 (Ar C=C str.), 1556 (C=N str.), 1243, 1070 (C-O-C stretching), 3354 (N-H stretching), 1675 (C=O stretching), 3055 (Ar-H); ^1^H-NMR (600 MHz, CDCl_3_, *δ* / ppm): 7.94 (s, 1H, H-3'''), 7.71 (d, *J* = 8.9 Hz, 2H, H-2'' & H-6''), 6.97-6.8 3 (m, 2H, H-5''' & H-6'''), 7.01 (d, *J* = 8.9 Hz, 2H, H-3'' & H-5''), 4.05 (s, 2H, H -1''''), 2.28 (s, 1H, H-7'''), 4.00-3.97 (m, 1H, H*e*-2'), 3.91 (s, 3H, H-7''), 3.72 -3.70 (m, 1H, H*a*-2'), 3.05 -3.01 (m, 1H, H-3'), 2.88 (s, 3H, H-8'''), 2.80 (s, 3H, H-7'''), 2.56 -2.53 (m, 1H, H*e*-6'), 2.37-2.33 (m, 1H, H*a*-6'), 2.13-2.08 (m, 1H, H*a*-5'), 1.87 -1.84 (m,2H, H*a*-4'), 1.73 -1.68 (m, 1H, H*e-*5'), 1.52 -1.49 (m, 1H, H*e*-4'). ^13^C-NMR (150 MHz, CDCl_3_, *δ* / ppm): 167.12 (C-8'''), 163.18 (C-1''''), 161.00 (C-5), 160.70 (C-2), 160.22 (C-4''), 138.82 (C-1'''), 133.93 (C-4'''), 133.74 (C-6'''), 133.61 (C-3'''), 133.55 (C-5'''), 132.29 (C-1''), 129.74 (C-2'' & C-6''), 115.34 (C-2'''), 114.37 (C-3'' & C-5''), 55.64 (C-7''), 52.64 (C-2'), 51.55 (C-9'''), 48.41 (C-6'), 46.19 (C-2''''), 33.55 (C-3'), 27.48 (C-4'), 23.64 (C-5'), 21.33 (C-7''').

**H-NMR of compound 7o**

**C-NMR of compound 7o**
